# Regioselective synthesis of heterocyclic *N*-sulfonyl amidines from heteroaromatic thioamides and sulfonyl azides

**DOI:** 10.3762/bjoc.16.243

**Published:** 2020-12-01

**Authors:** Vladimir Ilkin, Vera Berseneva, Tetyana Beryozkina, Tatiana Glukhareva, Lidia Dianova, Wim Dehaen, Eugenia Seliverstova, Vasiliy Bakulev

**Affiliations:** 1 TOS Department, Ural Federal University named after the first President of Russia B.N. Yeltsin, 19 Mira St., 620002 Yekaterinburg, Russia; 2 Molecular Design and Synthesis, Department of Chemistry, KU Leuven, Celestijnenlaan 200F, 3001 Leuven, Belgium

**Keywords:** amidines, Dimroth rearrangement, isoxazoles, sulfonyl thiazoles, thioamides, 1,2,3-triazoles

## Abstract

*N*-Sulfonyl amidines bearing 1,2,3-triazole, isoxazole, thiazole and pyridine substituents were successfully prepared for the first time by reactions of primary, secondary and tertiary heterocyclic thioamides with alkyl- and arylsulfonyl azides. For each type of thioamides a reliable procedure to prepare *N*-sulfonyl amidines in good yields was found. Reactions of 1-aryl-1,2,3-triazole-4-carbothioamides with azides were shown to be accompanied with a Dimroth rearrangement to form 1-unsubstituted 5-arylamino-1,2,3-triazole-4-*N*-sulfonylcarbimidamides. 2,5-Dithiocarbamoylpyridine reacts with sulfonyl azides to form a pyridine bearing two sulfonyl amidine groups.

## Introduction

**Figure 1:**
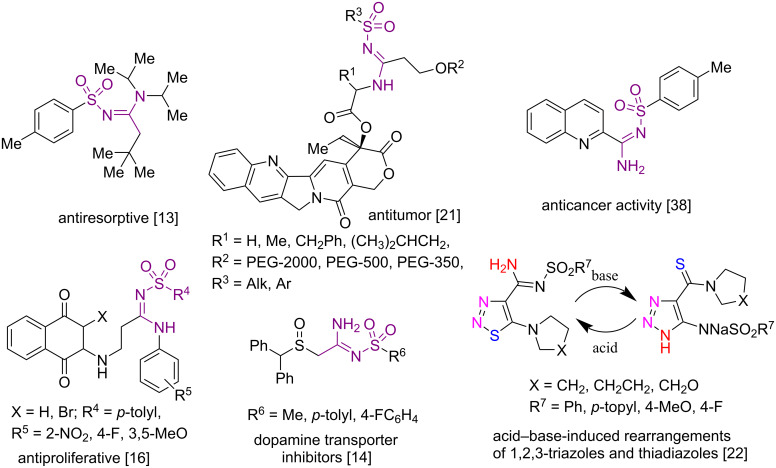
Examples of biological activity and interesting chemical reactivity of *N*-sulfonyl amidines.

The biological activity, rich chemistry and technically useful properties of heterocyclic compounds have made them a focal point of science and industry over the years. Heterocyclic compounds including azoles and azines have been found in natural products, and they are included in the structures of nucleic acids, vitamins, antibiotics and in many types of synthetic drugs [1–12]. *N*-Sulfonyl amidines have received considerable attention because they exhibit various types of pharmaceutical properties and biological activities [13–21] and also have been used as interesting building blocks in organic synthesis [17–20] (Figure 1).

An *N*-sulfonyl amidine was recently found to be a key group in acid–base-induced rearrangements of 1,2,3-triazoles and thiadiazoles [22]. A variety of methods have been developed for the synthesis of *N*-sulfonyl amidines. The most commonly used methods to prepare these compounds include the Cu-catalyzed multicomponent reaction of alkynes, sulfonyl azides and amines [23–31], the reaction of thioacetamide derivatives and cyclic thioamides with sulfonyl azides [22,32–33], the chlorophosphite-mediated Beckmann reaction of oximes with *p*-toluenesulfonyl azide [34], the sulfonyl ynamide rearrangement by treatment with amines [35], the sodium iodide catalyzed reaction of sulfonamide with formamide [36], and the condensation of sulfonamide derivatives with DMF–DMA [37].

**Figure 2:**
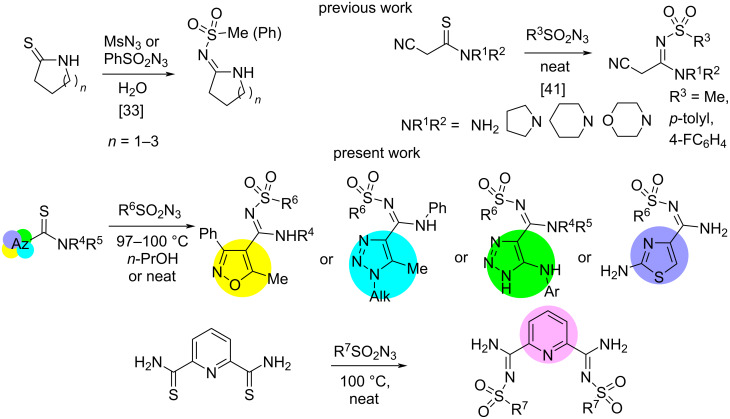
Data on the synthesis of *N′*-sulfonylazole-4-carboximidamides.

A few representatives of *N*-sulfonyl amidines of heteroaromatic acids have been prepared and applied [22,32,38–40]. However, no efficient and general method to prepare a series of heterocyclic *N*-sulfonyl amidines has been elaborated so far. A new approach to *N*-sulfonyl amidines has been published recently, based on the reaction of thioamides with sulfonyl azides [33,41–42] (Figure 2).

This method was used successfully for the synthesis of *N*-sulfonyl amidines of aliphatic acids and benzoic acid, including biologically active compounds. On the other hand, reactions of thioamides with electrophilic reagents have often been used for the synthesis of various types of sulfur containing heterocyclic compounds [43–47]. This gives some promise to the development of a general and efficient method for the synthesis of *N*-sulfonyl amidines of heteroaromatic acids based on the reaction of heterocyclic thioamides with higly electrophilic sulfonyl azides.

With the purpose of the synthesis of heterocyclic *N*-sulfonyl amidines bearing various heteroatoms in the ring, namely nitrogen, sulfur and oxygen atoms, we have studied reactions of thioamides of 1,2,3-triazole-, isoxazole-, thiazolecarboxylic acids and 2,5-dithiocarbamoylpyridine with sulfonyl azides. Due to the high dipole moment, the presence of electronegative heteroatoms bearing electron lone pairs, one could propose alternative reactions which might make it difficult to find a general regioselective procedure for the synthesis of the target molecules in good yields. To the best of our knowledge, there are no examples for the synthesis of *N*-sulfonyl amidines of heteroaromatic acids through this reaction so far.

## Results and Discussion

### 1-Alkyl-1,2,3-triazole-*N*-sulfonyl amidines

**Scheme 1:**
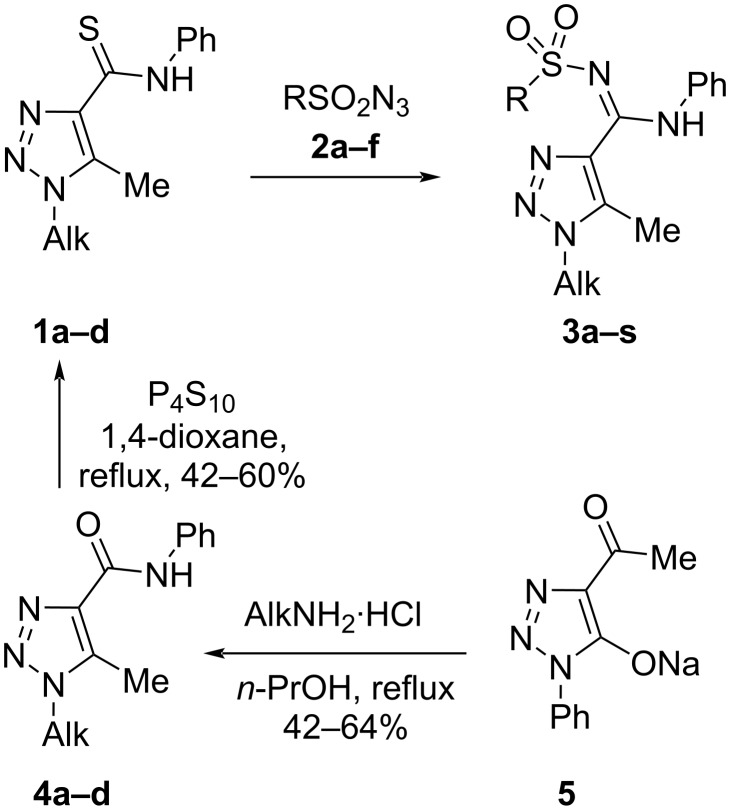
Synthesis of 1-alkyl-*N*-phenyl-*N*'-(sulfonyl)-1*H*-1,2,3-triazole-4-carboximidamides **3**.

Since 1,2,3-triazole derivatives exhibit valuable biological and technical properties, and take part in various ring transformations and rearrangements [48–51], we decided to study reactions of 1-alkyl-1,2,3-triazole-4-carbothioamides **1a**–**d** with aryl- and alkylsulfonyl azides **2a**–**f** with, with the goal of affecting a “iminosulfonylation” (Scheme 1).

**Figure 3:**
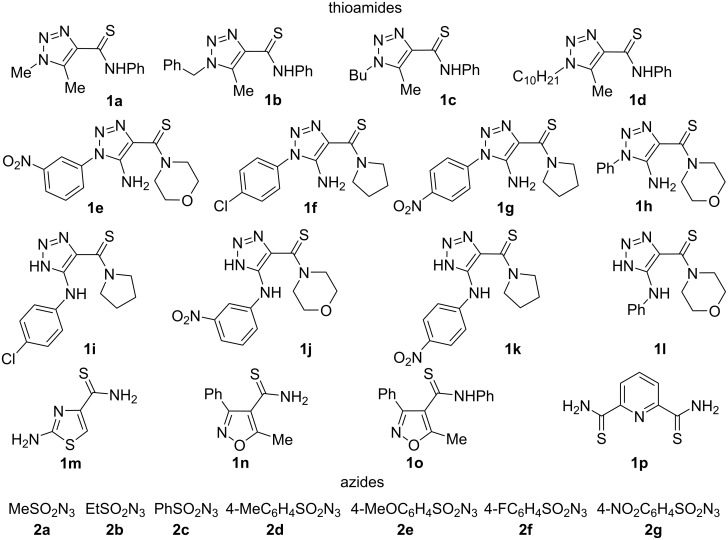
Starting compounds.

The thioamides **1a**–**d** were prepared from the corresponding amides **4a**–**d** by treatment with phosphorus decasulfide (Scheme 1). It is worth noting that amides of 1-alkyl-1,2,3-triazole-4-carboxylic acids are poorly represented in the literature and the methods of their preparation require the addition of alkyl azides to acetylene carboxylic esters and reactions of 2-diazomalonates with aliphatic amines [40,52]. The first approach leads to a mixture of two regioisomers and the second method involves the use of explosive diazo compounds. Therefore, such compounds are better prepared by a recently found method in our laboratory which includes the reaction of 4-acetyl-1,2,3-triazole **5a**–**d** with aniline followed by a Cornforth rearrangement of the 1,2,3-triazole ring [52]. Alkyl- (**2a**,**b**) and arylsulfonyl (**2c**–**g**) azides were prepared, respectively, from the corresponding sulfonyl chlorides and sodium azides according to published methods (Figure 3) [53].

We have found that 1-butyl-1,2,3-triazole-4-carbothioamide (**1c**) reacts well with benzenesulfonyl azide (**2c**) in various solvents to form the desired 1-butyl-1,2,3-*N*-sulfonyl amidine **3n** in diverse solvents such as *n*-butanol, *n*-propanol, toluene, ethanol, water and even under solvent-free conditions (see Table 1 for the yields and other circumstances).

**Table 1:** Optimizations of the reaction conditions for the reaction of thioamide **1с** with phenylsulfonyl azide **2c**^a^.

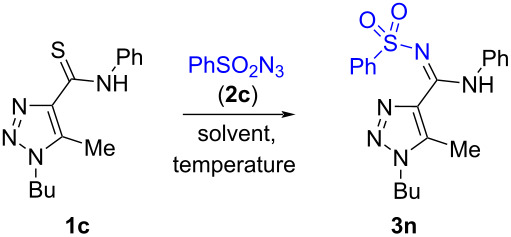					

Entry	Solvent	*T* (°C)	**2c** (equiv)	*t* (h)	Yield^b^ (%)

1	*n*-BuOH	117	5	21	23
2	*n*-BuOH	100	5	12.7	75
3	*n*-PrOH	97.4	5	12.7	80
4	*n*-PrOH	97.4	2.5	12.7	63
5	toluene	105	5	12.7	26
6	water	100	5	12.7	61
7	ethanol	78.4	5	21	74
8	neat	88	1.2	12.7	34
9	neat	88	5	4	86
10	neat	55	5	24	63
**11**	**neat**	**88**	**2.5**	**5**	**87**

^a^Reaction conditions: 0.18 mmol of **1c**, solvent (1 mL); ^b^isolated yield.

From these data we can conclude that the yield of the final product is optimal for the reaction under solvent-free conditions. 1-Butyl-1,2,3-triazole **1с** reacts faster than 1,2,3-triazole-4-carbothioamide **1f** while using a lower amount of a sulfonyl azide (Table 1, entry 11 and Table 2, entry 14). Thus solvent-free conditions, a temperature of 88 °C and a thioamide/azide ratio of 1:2.5 are optimal to prepare *N*-sulfonyl amidine **1c** (entry 11, Table 1).

**Scheme 2:**
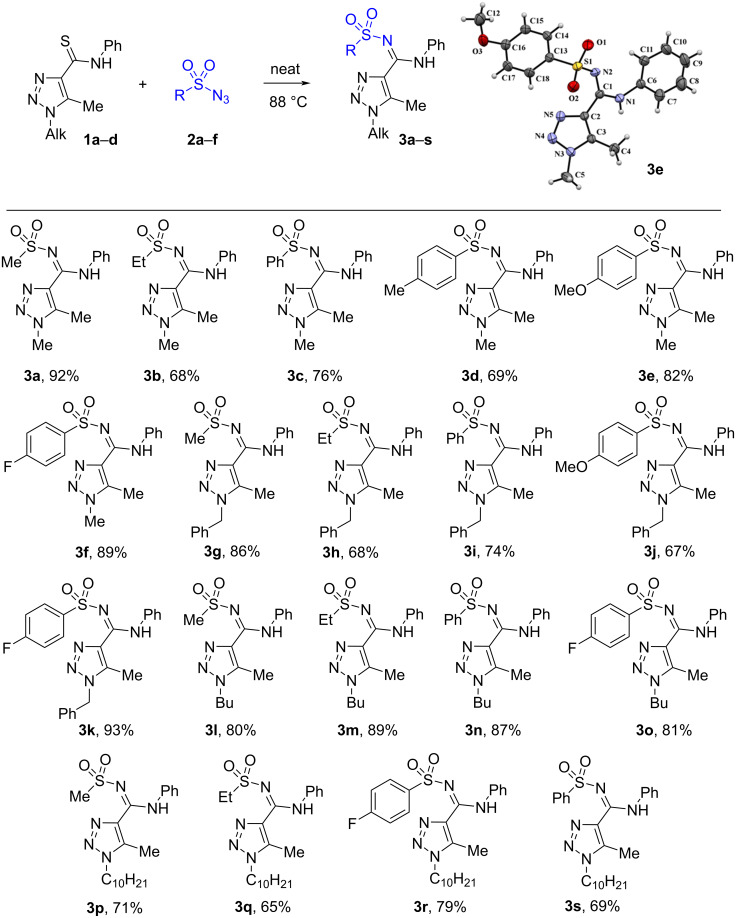
Scope for the reaction of 1-alkyl-1,2,3-triazole-4-carbothioamides **1a**–**d** with azides **2a**–**f**.

Next, these optimized conditions were used for the synthesis of a small library of 1-alkyl-1,2,3-triazoles **3a**–**s** (Scheme 2).

The reaction can be applied without problems to various alkyl substituents in position 1 of the 1,2,3-triazole ring from methyl to decyl and benzyl, goes well with alkylsulfonyl azides and arylsulfonyl azides that were 4-substituted with both electron-withdrawing and electron-donating substituents.

### 5-Arylamino-1,2,3-triazole-*N*-sulfonyl amidines

To further expand the scope of the reaction we continued studying the reaction of 1-aryl-1,2,3-triazole-4-carbothioamides **1e**–**h** with aryl- and alkylsulfonyl azides **2a**,**c**,**f**.

We have found that thioamide **1e** did react with benzenesulfonyl azide (**2c**) neither in water, ethanol nor in the absence of a solvent, conditions that were successfully used in the synthesis of 1-alkyl-1,2,3-triazole-4-*N*-sulfonylimidamides **3a**–**s** (Scheme 2). On the other hand, we have found the formation of a new product **3t** in low yield together with the starting compound **1e** and the product of its rearrangement to 5-(4-nitrophenyl)aminotriazole **1j** [54], when the reaction was carried out in *n*-butanol at 105 °C (Table 2). Therefore, we can conclude that compound **3t** was the product of a tandem reaction involving first the rearrangement of thioamide **1e** to **1j** followed by iminosulfonylation of the latter to form amidine **3t** (Table 2).

**Table 2:** Synthesis and optimization of the reaction conditions for the reaction of thioamide **1j** with phenylsulfonyl azide (**2c**)^a^.

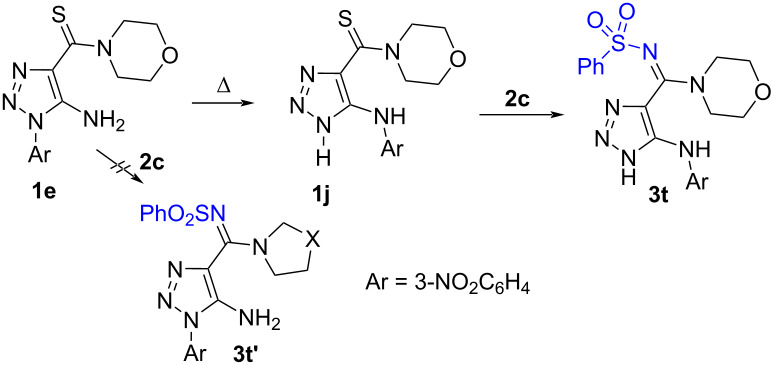					

Entry	Solvent (additive)	*T* (°C)	**2c** (equiv)	*t* (h)	Yield^b^ (%)

1	*n*-BuOH	105	1	17.5	33
2	*n*-BuOH	105	7	17.5	76
3	*n*-BuOH (Cs_2_CO_3_, 10 mol %)	105	5	5	no reaction
4	*n*-BuOH (CuI, 10 mol %)	105	5	5	no reaction
5	*n*-PrOH	88	5	17.5	43
6	*n*-PrOH	97.4	5	17.5	52
7	*n*-PrOH	97.4	7	17.5	78

^a^Reactions conditions: 0.45 mmol of thioamide **1j**, solvent (3 mL); ^b^Isolated yield.

To obtain higher yields of sulfonyl amidines we decided to prepare 5-arylamino-1,2,3-triazole-4-carbothioamide **1j** by rearrangement of triazole **1e** [54] and carried out an optimization with variations of the solvent, temperature and various additives (Table 2). We have shown that optimal conditions include the use of *n*-propanol, a temperature of 97 °C and a ratio of thioamide **1j** and azide **2c** of 1:7 which allowed to prepare the desired compound **3t** in 78% (Table 2).

**Scheme 3:**
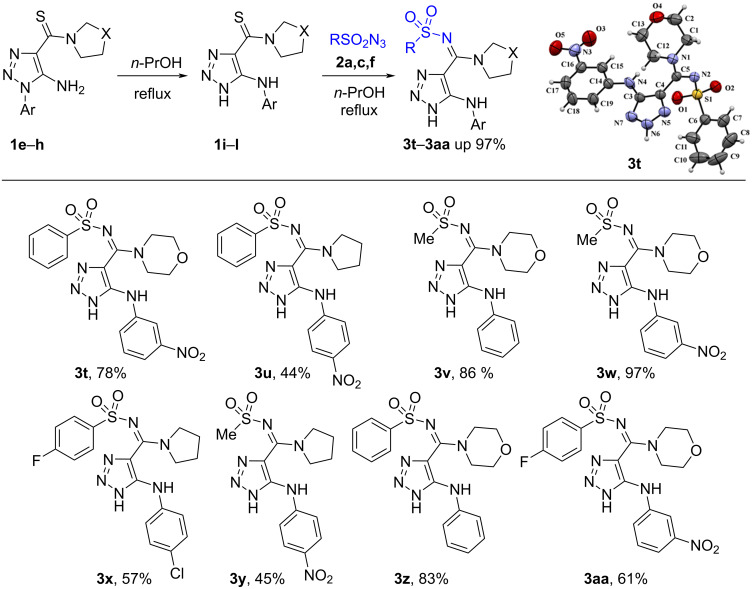
Scope of the reaction of 5-arylamino-1,2,3-triazole-4-carbothioamides **1i**–**l** with azides **2a**,**c**–**f**.

With the optimal conditions in hand we prepared a series of *N*-sulfonyl amidines **3t**–**aa** in good yields (Scheme 3). Thus, a library of *N*-sulfonyl amidines bearing differently substituted 1,2,3-triazoles was successfully prepared. Among them are compounds bearing an NH-unsubstituted 1,2,3-triazole ring which gives extra possibilities for the modification of the molecules by the reaction with electrophilic reagents to prepare new compounds of this series [55] (Scheme 3).

To show the practical convenience of the developed method we tried to synthesize these compounds in a one-pot procedure starting from readily available 1-aryl-1,2,3-triazoles **1f**,**g**,**t** and sulfonyl azides **2c**,**f** (Table 3). Thioamides **1f**,**g**,**t** were converted to 5-arylamino-1,2,3-triazoles **1i**–**k** by heating at reflux in *n*-propanol in the presence of DBU and these rearranged thioamides were then treated with sulfonyl azide **2c**,**f** and kept at the same temperature for 17‒31 h. After flash column chromatography, pure *N*-sulfonyl amidines **3t**,**u**,**x** were isolated in 41‒65 % yield. The data of Table 3 demonstrates that the yields of sulfonyl amidines **3t**,**u**,**x** are higher when we used the one-pot protocol in comparison with the two-step method. Furthermore, the one-pot procedure is obviously more simple and less time consuming.

**Table 3:** Yields of triazoles **3t**,**u**,**x** following a one-pot procedure^a^ compared to the yields involving the isolation of 5-arylamino-1,2,3-triazoles **1i**–**k**.

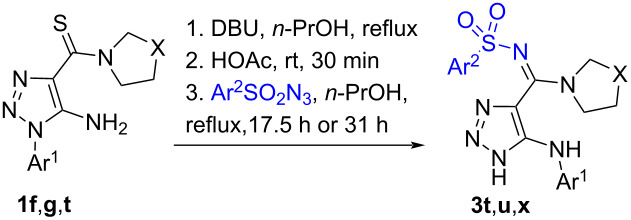					

Entry	Thioamide **1**	Azide **2**	Product **3**	Yield of **3**, % (time)	

				one-pot	with isolation of **1i**–**k**

1	**1t**	**2f**	**3x**	49 (17.5 h)	41 (via **1i**, 27.5 h)
2	**1g**	**2c**	**3u**	41 (31 h)	36 (via **1k**, 41 h)
3	**1f**	**2f**	**3t**	65 (31 h)	60 (via **1j**, 41 h)

^a^**1** (0.60‒0.65 mmol), DBU (0.63‒0.65 mmol), **2** (3.56‒4.0 mmol), HOAc (1 mL).

### 2-Aminothiazole-4-*N*-sulfonyl amidines

In spite of the presence of a nucleophilic amino group capable to react with sulfonyl azide to form an azide group, the reaction of azides **2** occurred selectively to the thioamide group of compound **1m**.

**Scheme 4:**
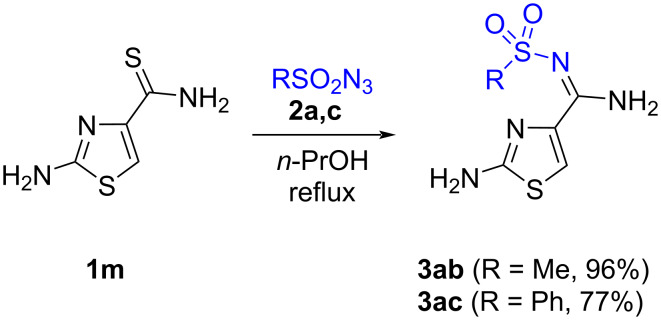
Synthesis of 2-aminothiazole-4-*N*-sulfonyl amidines.

Thus, similar to the reaction of 5-arylamino-1,2,3-triazole-4-carbothioamides **1i**–**l**, the reaction of the primary thioamide of 2-aminothiazole-4-carboxyamide (**1m**) with sulfonyl azides **2a**,**c** is succesful in *n*-propanol at reflux temperature, to afford *N*-sulfonyl amidines **3ab** and **3ac** bearing a 2-aminothiazole ring in very good yields (Scheme 4).

### 3-Methyl-5-phenyl-isoxazole-4-*N*-sulfonyl amidines

**Scheme 5:**
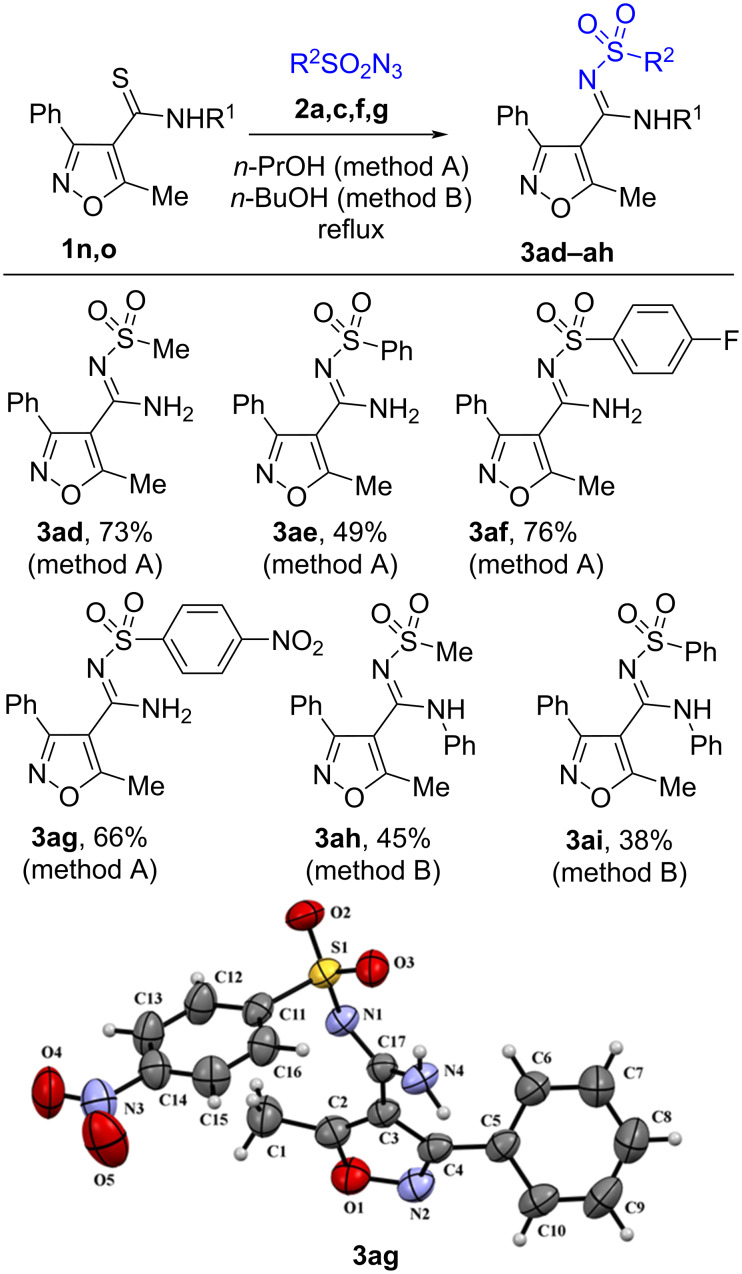
Synthesis of *N*-sulfonyl amidines of isoxazolylcarboxylic acid.

The primary thioamide **1n** containing an isoxazole ring was shown to react with mesyl azide or arylsulfonyl azides in *n*-propanol at reflux temperature to form the *N*-sulfonyl amidines **3ad**–**ag** in 49‒76% yields (Scheme 5).

The reaction takes place also in the absence of a solvent, albeit in lower yields. We have found that secondary thioamide **1o** does not react with sulfonyl azides **2a**,**c** either in *n*-propanol or in the absence of a solvent. On the other hand, we have found that the reaction can occur in *n*-butanol at 118 °C to form compounds **3ah**–**ai** in low yields (38‒45%) accompanied with the formation of tar-like products.

### 2,5-Bis(*N*-sulfonylamidino)pyridines

**Scheme 6:**
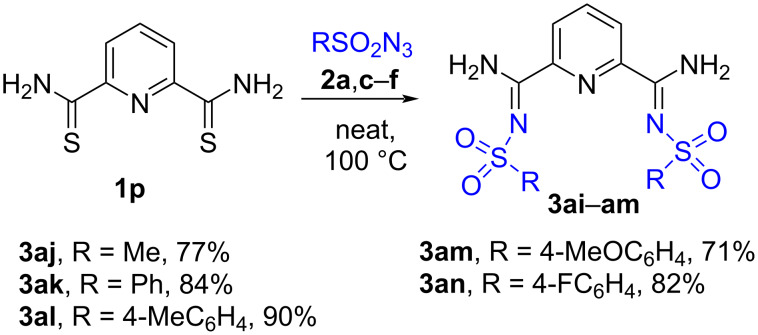
Synthesis of bis(sulfonyl amidines) **3aj**–**an**.

Bis(thioamide) **1p** containing a pyridine ring was found to react with sulfonyl azides **2a**,**c**–**f** either in boiling propanol or in the absence of a solvent to form compounds **3aj**–**an** bearing two *N*-sulfonyl amidine fragments connected to a pyridine linker. The solvent-free protocol includes the use of a lower amount of azide **2d**,**c**,**f** (2.5 equiv) in comparison with the reaction in *n*-propanol (4 equiv of azide) to afford the desired products in the same yield and therefore was selected as the method of choice for the synthesis of **3aj**–**an** (Scheme 6). The synthesis of complexes of bis(sulfonyl amidines) **3aj**–**an** with metals is in progress.

^1^H and ^13^C NMR spectra including 2D HMBC and HSQC experiments of compounds **3a**–**an**, as well as high-resolution mass spectra are consistent with the proposed structures. Carbon signals of the amidine groups of compounds **3** appear at 154.1‒159.7 ppm which is close to 156 ppm which is the value found for *N*-sulfonyl amidines of 1,2,3-thiadiazole-4-carboxylic acid prepared by another method [22] and was clearly different from the thioamide carbon signal at 185‒187 ppm in the ^13^C NMR spectra of starting materials **1**. A final proof of the structures of the prepared compounds comes from the X-ray data for **3e**,**t**,**ag** (Schemes 2, 3, and 5). Moreover, the X-ray data reveal the existence of *N*-sulfonyl amidines **3e**,**t** in the *E*-isomeric form and *N*-sulfonyl amidine **3ag** in *Z*-isomeric form. The existence of the latter in the *Z*-isomeric form can be explained by steric hindrance between the phenyl and the arylsulfonyl groups.

**Scheme 7:**
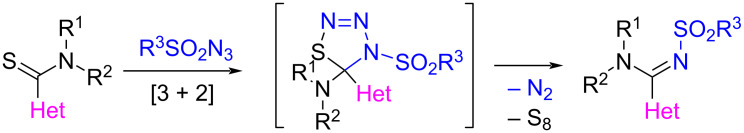
Plausible mechanism for the reaction of heterocyclic thioamides with sulfonyl azides.

Because of the observed evolution of nitrogen and sulfur in every reaction of heterocyclic thioamides and sulfonyl azides it is logic to propose the formation of a thiatriazole ring via [3 + 2] cycloaddition of the azide group and the C=S moiety of the thioamide group (Scheme 7).

The formation of nitrene-like products was excluded because of the high selectivity of the process, where only the thioamide group takes part, even with heterocyclic rings that contain other nucleophilic centers, and in one case, an amino group. Thiatriazoles are known to be unstable compounds that readily evolve nitrogen and sulfur upon heating [56].

## Conclusion

We have shown that the reaction of sulfonyl azides with thioamides can serve as the basis for a general and efficient method for the regioselective synthesis of *N*-sulfonyl amidines of azolyl and pyridine carboxylic acids. The most promising aspect for organic synthesis and green chemistry is a solvent-free process which was successfully applied to prepare sulfonyl amidines containing pyridine and isoxazolyl rings and 1-alkyl-1,2,3-triazole-4-*N*-sulfonylamidino-1,2,3-triazoles. The 1-alkyltriazole thioamides are the most active in the solvent-free method due to their low melting points and good solubility in alkyl- and arylsulfonyl azides. Conversely, thioamides containing 5-arylamino-1,2,3-triazole and 2-aminothiazole rings are not soluble in sulfonyl azides and could be transformed to the corresponding *N*-sulfonyl amidines by reactions in 1-propanol via two- or one-pot procedures. Pyridine-2,6-dithioamide was shown to react with mesyl and arylsulfonyl azides to form pyridine derivatives bearing two *N*-sulfonyl amidine moieties in excellent yield. Depending on the structure of the heterocycle the *N*-sulfonyl amidines exist in either *E*- or *Z*-isomeric forms.

## Experimental

### X-ray diffraction study

X-ray analyses were accomplished on an Xcalibur 3 diffractometer using the standard procedure (graphite-monochromated Mo Kα irradiation, ω-scanning with step 1o, *T* = 295(2) K (see Supporting Information File 1). Using Olex2 [57], the structures were solved with the Superflip [58] structure solution program using charge flipping and refined with the ShelXL [59] refinement package using least squares minimization. Deposition numbers for compounds **3e** (2020829), **3t** (2020831) and **3ag** (2020830), contain the supplementary crystallographic data for this paper. These data can be obtained free of charge from the Cambridge Crystallographic Data Centre via http://www.ccdc.cam.ac.uk/data_request/cif.

## Supporting Information

File 1 Full experimental details and characterization data of all new compounds, crystal data and structure refinement for **3e**, **3t**, and **3ag**.

File 2 Copies of NMR spectra of all new compounds.

File 3 Crystallographic information files for compounds **3e**, **3t** and **3ag**.
