# Supplementary material for: Regioselective synthesis of heterocyclic N-sulfonyl amidines from heteroaromatic thioamides and sulfonyl azides
Source: Beilstein J Org Chem. 2020 Dec 1;16:2937–47. doi: 10.3762/bjoc.16.243 (PMC7722631; doi:10.3762/bjoc.16.243)
Supplement: File 1 — Full experimental details and characterization data of all new compounds, crystal data and structure refinement for 3e, 3t, and 3ag. [file Beilstein_J_Org_Chem-16-2937-s001.pdf]

BEILSTEIN JOURNAL OF ORGANIC CHEMISTRY

Supporting Information

for

Regioselective synthesis of heterocyclic *N*-sulfonyl amidines from heteroaromatic thioamides and sulfonyl azides

Vladimir Ilkin, Vera Berseneva, Tetyana Beryozkina, Tatiana Glukhareva, Lidia Dianova, Wim Dehaen, Eugenia Seliverstova and Vasiliy Bakulev

Beilstein J. Org. Chem. **2020,** *16,* 2937–2947. doi:10.3762/bjoc.16.243

Full experimental details and characterization data of all new compounds, crystal data and structure refinement for 3e, 3t, and 3ag

License and Terms: This is a supporting information file under the terms of the Creative Commons Attribution License (https://creativecommons.org/licenses/by/4.0). Please note that the reuse, redistribution and reproduction in particular requires that the author(s) and source are credited and that individual graphics may be subject to special legal provisions.
The license is subject to the *Beilstein Journal of Organic Chemistry* terms and conditions: (https://www.beilstein-journals.org/bjoc/terms)

Table of Contents

General information S2

Preparation of amides S2

Preparation of thioamides S2

Preparation of azides S2

Full experimental and analytical data for compounds **1a–e,j,m,o** S2

Full experimental and analytical data for compounds **3a–s** S5

Full experimental and analytical data for compounds **3t–3aa** S12

Full experimental and analytical data for compounds **3ab, 3ac** S16

Full experimental and analytical data for compounds **3ad–3af** S17

Full experimental and analytical data for compounds **3ah**,**3ai** S18

Full experimental and analytical data for compounds **3aj–3an** S19

Full experimental and analytical data for compounds **4c,d** S21

Table 1. Crystal data and structure refinement for **3**e,**3t**,**3ag** S23

References S24

Experimental part

General information

Pyridine was dried over KOH and distilled prior to use. 1,4-Dioxane was dried and distilled over Na prior to use. Solvents used for silica gel chromatography (EtOAc, petroleum ether (40–70) (PE)) and crystallization (EtOH) were used without purification or removal of water. ^{1}H and ^{13}C NMR spectra were recorded at 400, 600 and 100, 150 MHz, respectively, in DMSO-d_6, CD_3CN, $CDCl_3$ or CD_3OD-d_4; the chemical shifts (δ) were expressed in ppm, and J values were given in Hz. High-resolution mass spectra (HRMS) were obtained with electrospray ionization (ESI-TOF). The compounds were analyzed in positive ion detection mode. The reactions were monitored by analytical TLC on aluminum foil plates with 0.2 mm silica gel with a fluorescent indicator visualized under UV light. The column chromatography was performed with 60–120 mesh silica gel. Melting points were determined on a melting point apparatus and are uncorrected.

Preparation of amides

5-Methyl-3-phenylisoxazole-4-carboxamide [1], 5-methyl-*N*,3-diphenylisoxazole-4-carboxamide [2], pyridine-2,6-dicarboxamide [3], 2-aminothiazole-4-carboxamide [4], 1,5-dimethyl-*N*-phenyl-1*H*-1,2,3-triazole-4-carboxamide, 1-benzyl-5-methyl-*N*-phenyl-1*H*-1,2,3-triazole-4-carboxamide, 1-butyl-5-methyl-*N*-phenyl-1*H*-1,2,3-triazole-4-carbothioamide, 1-decyl-5-methyl-N-phenyl-1*H*-1,2,3-triazole-4-carbothioamide [5] were synthesized according to previously reported procedures.

Preparation of thioamides

(5-Amino-1-(4-chlorophenyl)-1*H*-1,2,3-triazol-4-yl)(pyrrolidin-1-yl)methanethione (**1f**), (5-amino-1-(4-nitrophenyl)-1*H*-1,2,3-triazol-4-yl)(pyrrolidin-1-yl)methanethione (**1g**), (5-amino-1-phenyl-1*H*-1,2,3-triazol-4-yl)(morpholino)methanethione (**1h**), (5-((4-chlorophenyl)amino)-1*H*-1,2,3-triazol-4-yl)(pyrrolidin-1-yl)methanethione (**1i**), (5-((4-nitrophenyl)amino)-*1H*-1,2,3-triazol-4-yl)(pyrrolidin-1-yl)methanethion (**1k**), morpholino(5-(phenylamino)-1*H*-1,2,3-triazol-4-yl)methanethione (**1l**) [6], 5-methyl-3-phenylisoxazole-4-carbothioamide (**1n**) [7], pyridine-2,6-bis(carbothioamide) (**1p**) [8], 3-(morpholin-4-yl)-3-thioxopropanenitrile [9] were synthesized according to previously reported procedures.

Preparation of azides

Sulfonyl azides **2a-f** [10] and 3-nitrophenyl azide [11] were prepared from the corresponding sulfonyl chlorides following the literature procedures. Warning! Sulfonyl azides are potentially explosive, and all reactions should be carried out behind blast shields. We recommend the use of plastic spatulas for the handling of solid material.

*General procedure for the preparation of carbothioamides **1a–d***

A solution of corresponding amide **4** (1.0 equiv.) in anhydrous 1,4-dioxane (25 mL) was heated to 80 ºC and P_4S_{10} (0.8 equiv) was added portion by portion. The reaction mixture was heated under reflux for 2–4.25 h. The solution was separated from the oily precipitate by decantation, and the 1,4-dioxane was evaporated under reduced pressure. The

residue was purified by flash chromatography on silica gel (60–120) using ethyl acetate/petroleum ether mixtures (1:2, 1:3, 1:4).

1,5-Dimethyl-*N*-phenyl-1*H*-1,2,3-triazole-4-carbothioamide (1a)

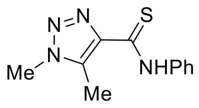

Prepared according to the general procedure from amide 1,5-dimethyl-*N*-phenyl-1*H*-1,2,3-triazole-4-carboxamide (**4a**) (1.582 g, 7.31 mmol) and P_4S_{10} (2.785 g, 6.26 mmol). Bright yellow solid, yield 41% (0.700 g), mp 113–115 °C. ^{1}H NMR (400 MHz, DMSO-*d$_6$*) δ 11.81 (s, 1H, NH), 7.78 (d, J = 7.9 Hz, 2H, CH_{Ar}), 7.42 (t, J = 7.8 Hz, 2H, CH_{Ar}), 7.26 (t, J = 7.4 Hz, 1H, CH_{Ar}), 4.01 (s, 3H, CH_3), 2.68 (s, 3H, CH_3). ^{13}C NMR (100 MHz, DMSO-*d$_6$*): δ 10.1, 34.6, 124.8 (2C), 126.2, 128.3 (2C), 137.8, 138.9, 143.0, 185.1. HRMS (ESI) m/z: $[M+H]^+$ calcd. for $C_{11}H_{13}N_4S$ 233.0855; found 233.0858.

1-Benzyl-5-methyl-*N*-phenyl-1*H*-1,2,3-triazole-4-carbothioamide (1b)

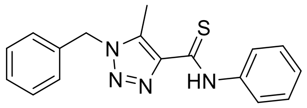

Prepared according to the general procedure from 1-benzyl-5-methyl-*N*-phenyl-1*H*-1,2,3-triazole-4-carbothioamide (**4b**) (2.500 g, 8.55 mmol) and P_4S_{10} (3.230 g, 7.26 mmol). Bright yellow solid, yield 52% (1.382 g), mp 111–113 °C. ^{1}H NMR (400 MHz, DMSO-*d$_6$*) δ 11.87 (s, 1H, NH), 7.77 (d, J = 7.9 Hz, 2H, CH_{Ar}), 7.23–7.44 (m, 8H, CH_{Ar}), 5.70 (s, 2H, CH_2), 2.63 (s, 3H, CH_3). ^{13}C NMR (100 MHz, DMSO-*d$_6$*) δ 10.3, 51.0, 124.7 (2C), 126.2, 127.3 (2C), 128.1, 128.4 (2C), 128.9 (2C), 135.2, 137.4, 138.9, 143.5, 185.0. δ. HRMS (ESI) m/z: $[M+H]^+$ calcd. for $C_{17}H_{17}N_4S$ 309.1168; found 309.1179.

1-Butyl-5-methyl-*N*-phenyl-1*H*-1,2,3-triazole-4-carbothioamide (1c)

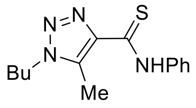

Prepared according to the general procedure from amide **4c** (2.50 g, 9.68 mmol) and P_4S_{10} (3.20 g, 7.20 mmol). Bright yellow solid, yield 60% (1.60 g), mp 92–94 °C. ^{1}H NMR (400 MHz, DMSO-*d*6) δ 11.83 (s, 1H, NH), 7.77 (d, J = 7.6 Hz, 2H, CH_{Ar}), 7.42 (t, J = 7.9 Hz, 2H, CH_{Ar}), 7.26 (t, J = 7.4 Hz, 1H, CH_{Ar}), 4.37 (t, J = 7.2 Hz, 2H, CH_2), 2.70 (s, 3H, CH_3), 1.82–1.76 (m, 2H, CH_2), 1.34–1.28 (m, 2H, CH_2), 0.92 (t, J = 7.4 Hz, 3H). ^{13}C NMR (100 MHz, DMSO-*d*6) δ 10.2, 13.4, 19.2, 31.0, 47.2, 119.2, 124.8 (2C), 126.2, 128.4 (2C), 137.2, 139.0, 143.1, 185.2.HRMS (ESI) m/z: $[M+H]^+$ calcd. for $C_{14}H_{19}N_4S$ 275.1325; found 275.1337.

1-Decyl-5-methyl-*N*-phenyl-1*H*-1,2,3-triazole-4-carbothioamide (1d)

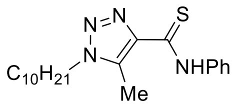

Prepared according to the general procedure from amide **4d** (2.50 g, 7.30 mmol) and P_4S_{10} (2.60 g, 5.85 mmol). Yellow solid, yield 54% (1.422 g), mp 67–69 °C.^{1}H NMR (400 MHz, CD_3CN) δ 10.82 (s, 1H, NH), 7.77 (d, *J* = 7.8 Hz, 2H, CH_{Ar}), 7.44 (t, *J* = 7.8 Hz, 2H, CH_{Ar}), 7.30 (t, *J* = 7.4 Hz, 1H, CH_{Ar}), 4.31 (t, *J* = 7.2 Hz, 2H, CH_2), 2.74 (s, 3H, CH_3), 1.82–1.89 (m, 2H, CH_2), 1.27–1.34 (m, 14H, CH_2), 0.88 (t, *J* = 6.5 Hz, 3H, CH_3). ^{13}C NMR (100 MHz, CD_3CN) δ 11.2, 14.4, 23.4, 27.1, 29.7, 30.0, 30.1, 30.26, 32.0, 49.0, 125.6 (2C), 127.5, 129.6 (2C), 139.0, 139.6, 143.5, 186.6. HRMS (ESI) m/z: $[M+H]^+$ calcd. for $C_{20}H_{31}N_4S$ 359.2264; found 359.2273.

(5-Amino-1-(3-nitrophenyl)-1*H*-1,2,3-triazol-4-yl)(morpholino)methanethione (1e)

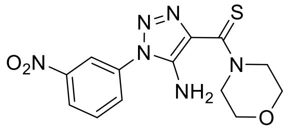

To a suspension of 3-(morpholin-4-yl)-3-thioxopropanenitrile (300 mg, 1.76 mmol, 1.0 equiv) and 3-nitrophenyl azide (304 mg, 1.85 mmol,1.05 equiv) in *n*-PrOH (5 mL) DBU (282 mg, 1.85 mmol, 1.05 equiv) was added at ambient temperature. The obtained solution was stirred for 1.5 h. After the completion of the reaction, the formed precipitate was filtered, washed with ethanol, diethyl ether and dried in a vacuum desiccator. Pale yellow solid, yield 84% (495 mg), mp 148–150 °C. ^{1}H NMR (400 MHz, DMSO-d_6) δ 8.43–8.40 (m, 2H, CH_{Ar}), 8.10 (d, *J* = 7.9 Hz, 1H, CH_{Ar}), 7.92 (d, *J* = 8.1 Hz, 1H, CH_{Ar}), 7.21 (s, 2H, NH_2), 4.35 (br. s, 4H, $2CH_2$), 3.75 (br. s, 4H, $2CH_2$). ^{13}C NMR (100 MHz, DMSO-d_6): δ 49.5, 52.7, 66.1, 119.5, 123.7, 125.9, 130.8, 131.2, 135.4, 146.6, 148.2, 184.2. EIMS (m/z): 334 $[M]^+$ (70), 317 (29), 86 (100). Anal. Calcd for $C_{13}H_{14}N_6O_3S$: C, 46.70; H, 4.22; N, 25.14; found: C, 47.03; H, 4.29; N, 25.27.

Morpholino(5-((3-nitrophenyl)amino)-1*H*-1,2,3-triazol-4-yl)methanethione (1j)

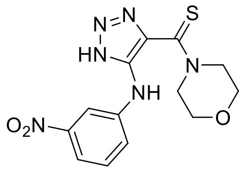

To a suspension of thioamide **1e** (400 mg, 1.2 mmol, 1.0 equiv) in *n*-PrOH (10 mL) DBU (191 mg, 1.25 mmol, 1.05 equiv) was added. The reaction mixture was refluxed for 3 h. After the completion of the rearrangement and cooling of the reaction mixture, glacial acetic acid (0.1 mL) was added, followed by stirring of the reaction mixture for 0.5 h at ambient temperature. The formed precipitate was filtered, washed with ethanol, diethyl ether and dried in a vacuum desiccator. Bright yellow solid, yield 92% (360 mg), mp 254–256 °C. ^{1}H NMR (400 MHz, DMSO-d_6) δ 14.94 (br. s, 1H, NH), 9.12 (s, 1H, NH), 8.46 (s, 1H, CH_{Ar}), 7.76 (d, *J* = 7.9 Hz, 2H, CH_{Ar}), 7.68 (d, *J* = 8.0 Hz, 2H, CH_{Ar}), 7.51 (t, *J* = 8.2 Hz, 2H, CH_{Ar}), 4.31 (s, 2H, CH_2), 3.89 (s, 2H, CH_2), 3.79 (s, 2H, CH_2), 3.64 (s, 2H, CH_2). ^{13}C NMR (100 MHz, DMSO-d_6): δ 49.1, 52.6, 65.7, 66.2, 109.4, 114.1, 122.3, 130.1,

132.1, 143.1, 145.5, 148.5, 185.2. EIMS (m/z): 334 $[M]^+$ (77), 317 (31), 86 (100). Anal. Calcd for $C_{13}H_{14}N_6O_3S$: C, 46.70; H, 4.22; N, 25.14; found: C, 46.45; H, 4.41; N, 25.16.

2-Aminothiazole-4-carbothioamide (1m)

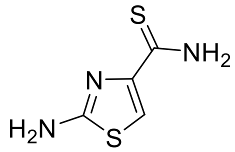

To a solution of 2-aminothiazole-4-carboxamide (1.0 g, 6.98 mmol, 1.0 equiv.) in anhydrous pyridine (20 mL) P_4S_{10} (2.8 g, 6.30 mmol, 0.9 equiv.) was added in small portions at ambient temperature. The reaction mixture was stirred under reflux for 2 h. The obtained solution was cooled and pyridine was evaporated under reduced pressure. The crude mixture was purified by flash chromatography on silica gel (eluent – hexane/EtOAc 4:6 → 2:8) to afford thioamide **1m**. Orange solid, yield 54% (602 mg), mp 192–194 °C. ^1H NMR (400 MHz, DMSO-d_6) δ 9.70 (s, 1H, NH_2), 8.77 (s, 1H, NH_2), 7.46 (s, 1H, $CH_{thiaz.}$), 7.09 (s, 2H, NH_2). ^{13}C NMR (100 MHz, DMSO-d_6): δ 115.5, 149.6, 167.8, 189.4. HRMS (ESI) m/z: $[M+H]^+$ calc. for $C_4H_6N_3S_2$ 159.9998; found 160.0006.

5-Methyl-*N*,3-diphenylisoxazole-4-carbothioamide (1o)

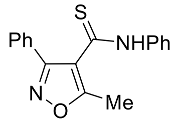

A solution of 5-methyl-3-phenylisoxazole-4-carboxamide (1000 mg, 3.59 mmol, 1.0 equiv.) in adhydrous 1,4-dioxane (20 mL) was heated to 80 ºC, and P_4S_{10} (1440 mg, 3.24 mmol, 0.8 equiv.) was added in portions. The reaction mixture was stirred under reflux for 3 h. The solution was separated from an oily precipitate by decantation, and the solvent was evaporated under reduced pressure. The crude mixture was purified by washing with water and filtered (2 × 25 mL). The collected precipitate was recrystallized from ethanol, filtered, washed with ethanol and dried in a ofvacuum desiccator. Bright yellow solid, yield 66% (700 mg), mp 241–242 °C. ^1H NMR (400 MHz, DMSO-d_6) δ 11.96 (s, 1H, NH), 7.76 (d, J = 8.0 Hz, 2H, CH_{Ar}), 7.69–7.70 (m, 2H, CH_{Ar}), 7.49–7.41 (m, 5H, CH_{Ar}), 7.28 (t, J = 7.2 Hz, 1H, CH_{Ar}), 2.55 (s, 3H, CH_3). ^{13}C NMR (100 MHz, DMSO-d_6): δ 11.4, 120.2, 123.2 (2C), 126.6, 127.6 (2C), 128.0, 128.7 (2C), 128.8 (2C), 129.9, 139.0, 158.8, 167.7, 187.4. HRMS (ESI) m/z: $[M+H]^+$ calc. for $C_{17}H_{15}N_2OS$ 295.0900; found 295.0913.

*General procedure for the preparation of N-sulfonylamidines **3a-s***

A mixture of the corresponding thioamide **1a-d** (1.0 equiv.) and sulfonyl azide **2a-f** (1.2–1.25 equiv.) was heated at 85–95 °C in an oil bath for 6–33.5 h. The reaction mixture was cooled, and petroleum ether was added. The formed precipitate was collected by filtration and washed with petroleum ether. Ethanol or chloroform was added, and the mixture was refluxed for 5–10 min. The precipitate containing S_8 was filtered off and the filtrate was concentrated *in vacuo.* Petroleum ether was added to the residue, the formed precipitate was collected by filtration, and washed with ethanol and dried in a vacuum desiccator over P_4O_{10}.

(*E*)-1,5-Dimethyl-*N'*-(methylsulfonyl)-*N*-phenyl-1*H*-1,2,3-triazole-4-carboximidamide (3a)

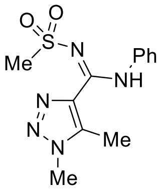

Prepared according to the general procedure from thioamide **1a** (150 mg, 0.64 mmol) and sulfonyl azide **2a** (95 mg, 0.78 mmol) for 16 h. White solid, yield 92% (174 mg), mp 199–201 °C. ^{1}H NMR (400 MHz, DMSO-*d*$_6$) δ 10.48 (s, 1H, NH), 7.71 (d, *J* = 7.5 Hz, 2H, CH_{Ar}), 7.40 (t, *J* = 7.7 Hz, 2H, CH_{Ar}), 7.20 (t, *J* = 7.2 Hz, 1H, CH_{Ar}), 4.01 (s, 3H, CH_3), 3.02 (s, 3 H, CH_3), 2.35 (s, 3H, CH_3). ^{13}C NMR (100 MHz, DMSO-*d*$_6$) δ 8.4, 34.4, 43.1, 122.0 (2C), 125.3, 128.8 (2C), 134.4, 138.0, 138.2, 154.6. HRMS (ESI) m/z: [M+H]$^+$ calcd. for $C_{12}H_{16}N_5O_2S$ 294.1019; found 294.1013.

(*E*)-*N'*-(Ethylsulfonyl)-1,5-dimethyl-*N*-phenyl-1*H*-1,2,3-triazole-4-carboximidamide (3b)

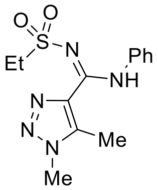

Prepared according to the general procedure from thioamide **1a** (150 mg, 0.64 mmol) and sulfonyl azide **2b** (105 mg, 0.78 mmol) for 9 h. White solid, yield 68% (134 mg), mp 194–196 °C. ^{1}H NMR (400 MHz, DMSO-*d*$_6$) δ 10.47 (s, 1H, NH), 7.70 (d, *J* = 7.7 Hz, 2H, CH_{Ar}), 7.40 (t, *J* = 7.8 Hz, 2H, CH_{Ar}), 7.20 (t, *J* = 7.3 Hz, 1H, CH_{Ar}), 4.00 (s, 3H, CH_3), 3.07 (q, *J* = 7.3 Hz, 2H, CH_2), 2.36 (s, 3H, CH_3), 1.22 (t, *J* = 7.3 Hz, 3H, CH_3). ^{13}C NMR (100 MHz, DMSO-*d*$_6$) δ 8.2, 8.5, 34.4, 48.9, 122.0 (2C), 125.3, 128.8 (2C), 134.4, 138.1, 138.4, 154.7. HRMS (ESI) m/z: [M+H]$^+$ calcd. for $C_{13}H_{18}N_5O_2S$ 308.1176; found 308.1180.

(*E*)-1,5-Dimethyl-*N*-phenyl-*N'*-(phenylsulfonyl)-1*H*-1,2,3-triazole-4-carboximidamide (3c)

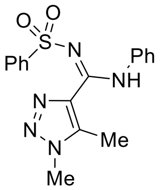

Prepared according to the general procedure from thioamide **1a** (150 mg, 0.64 mmol) and sulfonyl azide **2c** (141 mg, 0.77 mmol) for 9 h. White solid, yield 76% (174 mg), mp 171–173 °C. ^{1}H NMR (400 MHz, DMSO-*d*$_6$) δ 10.68 (s, 1H, NH), 7.70 (d, *J* = 7.2 Hz, 2H, CH_{Ar}), 7.61–7.51 (m, 5H, CH_{Ar}), 7.32 (t, *J* = 7.8 Hz, 2H, CH_{Ar}), 7.17 (t, *J* = 7.3 Hz, 1H, CH_{Ar}), 4.00 (s, 3H, CH_3), 2.32 (s, 3H, CH_3). ^{13}C NMR (100 MHz, DMSO-*d*$_6$) δ 8.5, 34.4, 122.0, 125.6, 125.9 (2C), 128.7, 128.8 (2C), 131.9, 134.8, 137.7, 137.9, 143.0, 154.3. HRMS (ESI) m/z: [M+H]$^+$ calcd. for $C_{17}H_{18}N_5O_2S$ 356.1176; found 356.1174.

(*E*)-1,5-Dimethyl-*N*-phenyl-*N'*-tosyl-1*H*-1,2,3-triazole-4-carboximidamide (3d)

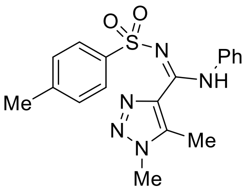

Prepared according to the general procedure from thioamide **1a** (150 mg, 0.64 mmol) and sulfonyl azide **2d** (154 mg, 0.78 mmol) for 18.7 h. White solid, yield 69% (164 mg), mp 151–153 °C. ^{1}H NMR (400 MHz, DMSO-*d*$_6$) *δ* 10.63 (s, 1H, NH), 7.59–7.54 (m, 4H, CH$_{Ar}$), 7.33–7.30 (m, 4H, CH$_{Ar}$), 7.17 (t, *J* = 7.3 Hz, 1H, CH$_{Ar}$), 3.99 (s, 3H, CH$_3$), 2.37 (s, 3H, CH$_3$), 2.31 (s, 3H, CH$_3$). ^{13}C NMR (100 MHz, DMSO-*d*$_6$) *δ* 8.5, 20.9, 34.3, 122.0 (2C), 125.5, 126.0 (2C), 128.7 (2C), 129.2 (2C), 134.7, 137.8, 138.0, 140.3, 142.0, 154.1. HRMS (ESI) m/z: [M+H]$^+$ calcd. for $C_{18}H_{20}N_5O_2S$ 370.1332; found 370.1328.

(*E*)-*N'*-((4-Methoxyphenyl)sulfonyl)-1,5-dimethyl-*N*-phenyl-1*H*-1,2,3-triazole-4-carboximidamide (3e)

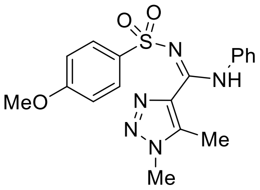

Prepared according to the general procedure from thioamide **1a** (150 mg, 0.64 mmol) and sulfonyl azide **2e** (167 mg, 0.78 mmol) for 16 h. White solid, yield 82% (204 mg), mp 186–188 °C. ^{1}H NMR (400 MHz, DMSO-*d*$_6$) *δ* 10.58 (s, 1H, NH), 7.62 (d, *J* = 8.4 Hz, 2H, CH$_{Ar}$), 7.56 (d, *J* = 7.0 Hz, 2H, CH$_{Ar}$), 7.33 (t, *J* = 7.5 Hz, 2H, CH$_{Ar}$) , 7.17 (t, *J* = 7.1 Hz, 1H, CH$_{Ar}$), 7.04 (d, *J* = 8.4 Hz, 2H, CH$_{Ar}$), 3.99 (s, 3H, OCH$_3$), 3.82 (s, 3H, CH$_3$), 2.31 (s, 3H, CH$_3$). ^{13}C NMR (100 MHz, DMSO-*d*$_6$) *δ* 8.5, 34.3, 55.6, 113.9 (2C), 121.9 (2C), 125.4, 128.1 (2C), 128.7 (2C), 134.6, 135.0, 137.9, 137.9, 153.9, 161.7. HRMS (ESI) m/z: [M+H]$^+$ calcd. for $C_{18}H_{20}N_5O_3S$ 386.1281; found 386.1272.

(*E*)-*N'*-((4-Fluorophenyl)sulfonyl)-1,5-dimethyl-*N*-phenyl-1*H*-1,2,3-triazole-4-carboximidamide (3f)

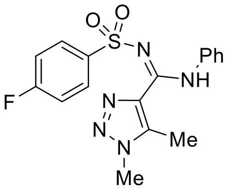

Prepared according to the general procedure from thioamide **1a** (150 mg, 0.64 mmol) and sulfonyl azide **2f** (156 mg, 0.77 mmol) for 11 h. White solid, yield 89% (216 mg), mp 127–129 °C. ^{1}H NMR (400 MHz, DMSO-*d*$_6$) *δ* 10.73 (s, 1H, NH), 7.77–7.73 (m, 2H, CH$_{Ar}$), 7.54 (d, *J* = 7.4 Hz, 2H, CH$_{Ar}$), 7.38–7.32 (m, 4H, CH$_{Ar}$), 7.19 (t, *J* = 7.3 Hz, 1H, CH$_{Ar}$), 4.01 (s, 3H, CH$_3$), 2.34 (s, 3H, CH$_3$). ^{13}C NMR (100 MHz, DMSO-*d*$_6$) *δ* 8.5, 34.4, 115.9 (d, *J* = 22.4 Hz), 122.1, 125.7 (2C), 128.7 (2C), 134.8, 128.95 (d, *J* = 9.5 Hz), 137.6, 137.8, 139.5 (d, *J* = 3.0 Hz), 163.7 (d, *J* = 250.1 Hz), 154.4. HRMS (ESI) m/z: [M+H]$^+$ calcd. for $C_{17}H_{17}FN_5O_2S$ 374.1081; found 374.1081.

(*E*)-1-Benzyl-5-methyl-*N'*-(methylsulfonyl)-*N*-phenyl-1*H*-1,2,3-triazole-4-carboximidamide (3g)

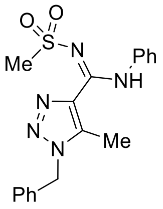

Prepared according to the general procedure from thioamide **1b** (150 mg, 0.49 mmol) and sulfonyl azide **2a** (72 mg, 0.59 mmol) for 6 h. White solid, yield 86% (154 mg), mp 184–186 °C. ^{1}H NMR (400 MHz, DMSO-*d*$_6$) *δ* 10.56 (s, 1H, NH), 7.71 (d, *J* = 7.1 Hz, 2H, CH$_{Ar}$), 7.42–7.32 (m, 5H, CH$_{Ar}$), 7.25–7.19 (m, 3H, CH$_{Ar}$), 5.68 (s, 2H, CH$_2$), 3.03 (s, 3H, CH$_3$), 2.30 (s, 3H, CH$_3$). ^{13}C NMR (100 MHz, DMSO-*d*$_6$) *δ* 8.5, 43.2, 50.6, 122.1 (2C), 125.4, 127.3 (2C), 128.0, 128.8 (4C), 134.2, 135.2, 138.0, 138.5, 154.5. HRMS (ESI) m/z: [M+H]$^+$ calcd. for $C_{18}H_{20}N_5O_2S$ 370.1332; found 370.1331.

(*E*)-1-Benzyl-*N'*-(ethylsulfonyl)-5-methyl-*N*-phenyl-1*H*-1,2,3-triazole-4-carboximidamide (3h)

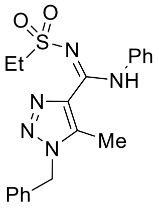

Prepared according to the general procedure from thioamide **1b** (150 mg, 0.49 mmol) and sulfonyl azide **2b** (94 mg, 0.69 mmol) for 23.5 h. White solid, yield 68% (127 mg), mp 155–157 °C. ^{1}H NMR (400 MHz, DMSO-*d*$_6$) *δ* 10.55 (s, 1H, NH), 7.71 (d, *J* = 7.4 Hz, 2H, CH$_{Ar}$), 7.42–7.34 (m, 5H, CH$_{Ar}$), 7.25–7.19 (m, 3H, CH$_{Ar}$), 5.68 (s, 2H, CH$_2$), 3.33 (s, 3H, CH$_3$), 3.07 (q, *J* = 7.3 Hz, 2H, CH$_2$), 2.31 (s, 3H, CH$_3$), 1.22 (t, *J* = 7.3 Hz, 3H, CH$_3$). ^{13}C NMR (100 MHz, DMSO-*d*$_6$) *δ* 8.2, 8.6, 48.9, 50.6, 122.0 (2C), 125.3, 127.3 (2C), 128.0, 128.8 (4C), 134.2, 135.2, 138.0, 138.6, 154.6. HRMS (ESI) m/z: [M+H]$^+$ calcd. for $C_{19}H_{22}N_5O_2S$ 384.1489; found 384.1485.

(*E*)-1-Benzyl-5-methyl-*N*-phenyl-*N'*-(phenylsulfonyl)-1*H*-1,2,3-triazole-4-carboximidamide (3i)

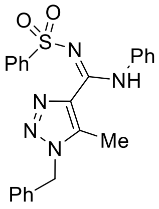

Prepared according to the general procedure from thioamide **1b** (150 mg, 0.49 mmol) and sulfonyl azide **2c** (107 mg, 0.58 mmol) for 21 h. White solid, yield 74% (155 mg), mp 145–147 °C. ^{1}H NMR (400 MHz, DMSO-*d*$_6$) *δ* 10.76 (s, 1H, NH), 7.68 (d, *J* = 7.4 Hz, 2H, CH$_{Ar}$), 7.59–7.28 (m, 12H, CH$_{Ar}$), 7.18 (t, *J* = 7.3 Hz, 1H, CH$_{Ar}$), 5.67 (s, 2H, CH$_2$), 2.28 (s, 3H, CH$_3$). ^{13}C NMR (100 MHz, DMSO-*d*$_6$) *δ* 8.5, 50.7, 122.1 (2C), 125.6, 125.9 (2C), 127.5 (2C), 128.1, 128.7 (2C), 128.8 (4C), 131.9, 134.5, 135.2, 137.7, 138.2, 143.0, 154.4. HRMS (ESI) m/z: [M+H]$^+$ calcd. for $C_{23}H_{22}N_5O_2S$ 432.1489; found 432.1489.

(*E*)-1-Benzyl-*N*'-((4-methoxyphenyl)sulfonyl)-5-methyl-*N*-phenyl-1*H*-1,2,3-triazole-4-carboximid-amide (3j)

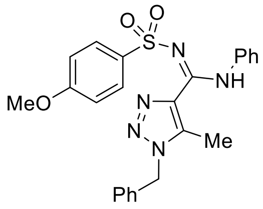

Prepared according to the general procedure from thioamide **1b** (150 mg, 0.49 mmol) and sulfonyl azide **2e** (124 mg, 0.58 mmol) for 27 h. White solid, yield 67% (151 mg), mp 139–141 °C. ^{1}H NMR (400 MHz, DMSO-d_6) δ 10.66 (s, 1H, NH), 7.61–7.56 (m, 4H, CH_{Ar}), 7.44–7.28 (m, 7H, CH_{Ar}), 7.17 (t, J = 7.3 Hz, 1H, CH_{Ar}), 7.0 (d, J = 8.8 Hz, 2H, CH_{Ar}), 5.67 (s, 2H, CH_2), 3.81 (s, 3H, OCH_3), 2.27 (s, 3H, CH_3). ^{13}C NMR (100 MHz, DMSO-d_6) δ 8.5, 50.7, 55.6, 113.9 (2C), 121.9, 125.5, 127.5 (2C), 128.1 (2C), 128.7 (2C), 128.8 (4C), 134.4, 135.0, 135.2, 137.8, 138.2, 154.1,161.7. HRMS (ESI) m/z: $[M+H]^+$ calcd. for $C_{24}H_{24}N_5O_3S$ 462.1594; found 462.1592.

(*E*)-1-Benzyl-*N*'-((4-fluorophenyl)sulfonyl)-5-methyl-*N*-phenyl-1*H*-1,2,3-triazole-4-carboximid-amide (3k)

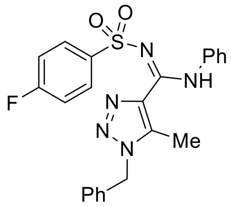

Prepared according to the general procedure from thioamide **1b** (150 mg, 0.49 mmol) and sulfonyl azide **2f** (117 mg, 0.58 mmol) for 6 h. White solid, yield 93% (204 mg), mp 185–187 °C. ^{1}H NMR (400 MHz, DMSO-d_6) δ 10.80 (s, 1H, NH), 7.75–7.71 (m, 2H, CH_{Ar}), 7.56–7.55 (m, 2H, CH_{Ar}), 7.44–7.28 (m, 9H, CH_{Ar}), 7.19 (t, J = 7.3 Hz, 1H, CH_{Ar}), 5.67 (s, 2H, CH_2), 2.30 (s, 3H, CH_3). ^{13}C NMR (100 MHz, DMSO-d_6) δ 8.5, 50.7, 115.9 (d, J = 22.5 Hz), 122.1, 125.7, 127.5 (2C), 128.1 (2C), 128.8 (4C), 128.9 (d, J = 9.4 Hz), 134.5, 135.2, 137.6, 138.1, 139.5 (d, J = 2.9 Hz), 154.5, 163.7 (d, J = 250.1 Hz). HRMS (ESI) m/z: $[M+H]^+$ calcd. for $C_{23}H_{21}FN_5O_2S$ 450.1394; found 450.1394.

(*E*)-1-Butyl-5-methyl-*N*'-(methylsulfonyl)-*N*-phenyl-1*H*-1,2,3-triazole-4-carboximidamide (3l)

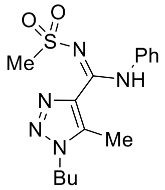

Prepared according to the general procedure from thioamide **1c** (150 mg, 0.55 mmol) and sulfonyl azide **2a** (80 mg, 0.66 mmol) for 13 h. White solid, yield 80% (147 mg), mp 156–158 °C. ^{1}H NMR (400 MHz, DMSO-d_6) δ 10.51 (s, 1H, NH), 7.72 (d, J = 7.2 Hz, 2H, CH_{Ar}), 7.40 (t, J = 7.7 Hz, 2H, CH_{Ar}), 7.21 (t, J = 7.3 Hz, 1H, CH_{Ar}), 4.35 (t, J = 7.0 Hz, 2H, CH_2), 3.01 (s, 3H, CH_3), 2.35 (s, 3H, CH_3), 1.81–1.74 (m, 2H, CH_2), 1.35–1.26 (m, 2H, CH_2), 0.93 (t, J = 7.3 Hz, 3H, CH_3). ^{13}C NMR (100 MHz, DMSO-d_6) δ 8.4, 13.4, 18.9, 31.2, 43.1, 47.0, 122.0 (2C), 125.3, 128.8 (2C), 133.9, 138.0, 138.1, 154.7. HRMS (ESI) m/z: $[M+H]^+$ calcd. for $C_{15}H_{22}N_5O_2S$ 336.1489; found 336.1489.

(*E*)-1-Butyl-*N'*-(ethylsulfonyl)-5-methyl-*N*-phenyl-1*H*-1,2,3-triazole-4-carboximidamide (3m)

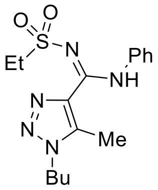

Prepared according to the general procedure from thioamide **1c** (150 mg, 0.55 mmol) and sulfonyl azide **2b** (88 mg, 0.65 mmol) for 23 h. White solid, yield 89% (171 mg), mp 118–120 °C. ^{1}H NMR (400 MHz, DMSO-d_6) δ 10.49 (s, 1H, NH), 7.70 (d, J = 7.7 Hz, 2H, CH_{Ar}), 7.40 (t, J = 7.8 Hz, 2H, CH_{Ar}), 7.20 (t, J = 7.3 Hz, 1H, CH_{Ar}), 4.34 (t, J = 7.0 Hz, 2H, CH_2), 3.06 (q, J = 7.3 Hz, 2H, CH_2), 2.35 (s, 3H), 1.81–1.74 (m, 2H, CH_2), 1.35–1.26 (m, 2H, CH_2), 1.21 (t, J = 7.3 Hz, 3H, CH_3), 0.92 (t, J = 7.3 Hz, 3H, CH_3). ^{13}C NMR (100 MHz, DMSO-d_6) δ 8.2. 8.5, 13.4, 18.9, 31.3, 47.0, 48.9, 122.0 (2C), 125.3, 128.8 (2C), 133.9, 138.1, 138.3, 154.8.HRMS (ESI) m/z: $[M+H]^+$ calcd. for $C_{16}H_{24}N_5O_2S$ 350.1645; found 350.1647.

(*E*)-1-Butyl-5-methyl-*N*-phenyl-*N'*-(phenylsulfonyl)-1*H*-1,2,3-triazole-4-carboximidamide (3n)

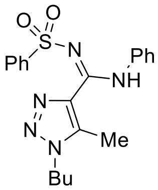

Prepared according to the general procedure from thioamide **1c** (49 mg, 0.18 mmol) and sulfonyl azide **2c** (82 mg, 0.45 mmol) for 5 h. White solid, yield 87% (62 mg), mp 103–105 °C. ^{1}H NMR (400 MHz, DMSO-d_6) δ 10.70 (s, 1H, NH), 7.70 (d, J = 7.3 Hz, 2H, CH_{Ar}), 7.60–7.51 (m, 5H, CH_{Ar}), 7.32 (t, J = 7.5 Hz, 2H, CH_{Ar}), 7.18 (t, J = 7.2 Hz, 1H, CH_{Ar}), 4.33 (t, J = 6.8 Hz, 2H, CH_2), 2.33 (s, 3H, CH_3), 1.82–1.75 (m, 2H, CH_2), 1.38–1.29 (m, 2H, CH_2), 0.94 (t, J = 7.3 Hz, 3H, CH_3). ^{13}C NMR (100 MHz, DMSO-d_6) δ 8.5, 13.4, 19.0, 31.2, 47.0, 122.0 (2C), 125.6, 125.9 (2C), 128.7 (2C), 128.8 (2C), 131.8, 134.2, 137.7, 137.9, 143.1, 154.5. HRMS (ESI) m/z: $[M+H]^+$ calcd. for $C_{20}H_{24}N_5O_2S$ 398.1645; found 398.1648.

(*E*)-1-Butyl-*N'*-((4-fluorophenyl)sulfonyl)-5-methyl-*N*-phenyl-1*H*-1,2,3-triazole-4-carboximidamide (3o)

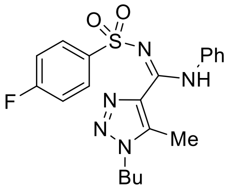

Prepared according to the general procedure from thioamide **1c** (150 mg, 0.55 mmol) and sulfonyl azide **2f** (132 mg, 0.66 mmol) for 14 h. White solid, yield 81% (185 mg), mp 145–147 °C. ^{1}H NMR (400 MHz, DMSO-d_6) δ 10.74 (s, 1H, NH), 7.76–7.72 (m, 2H, CH_{Ar}), 7.55 (d, J = 7.0 Hz, 2H, CH_{Ar}), 7.37–7.32 (m, 4H, CH_{Ar}), 7.19 (t, J = 7.3 Hz, 1H, CH_{Ar}), 4.34 (t, J = 7.0 Hz, 2H, CH_2), 2.33 (s, 3H, CH_3), 1.81–1.74 (m, 2H, CH_2), 1.38–1.28 (m, 2H, CH_2), 0.94 (t, J = 7.3 Hz, 3H, CH_3). ^{13}C NMR (100 MHz, DMSO-d_6) δ 8.4, 13.4, 19.0, 31.3, 47.0, 115.9 (d, J = 22.5, Hz), 122.1, 125.7, 128.7, 128.9 (d, J = 9.4, Hz), 134.2, 137.6, 137.8,

139.5 (d, J = 2.9 Hz), 154.6, 163.7 (d, J = 250.1 Hz). HRMS (ESI) m/z: $[M+H]^+$ calcd. for $C_{20}H_{23}FN_5O_2S$ 416.1551; found 416.1549.

(*E*)-1-Decyl-5-methyl-*N'*-(methylsulfonyl)-*N*-phenyl-1*H*-1,2,3-triazole-4-carboximidamide (3p)

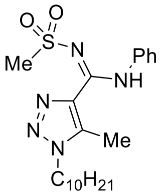

Prepared according to the general procedure from thioamide **1d** (150 mg, 0.42 mmol) and sulfonyl azide **2a** (62 mg, 0.51 mmol) for 17 h. White solid, yield 71% (124 mg), mp 158–160 °C. ^{1}H NMR (400 MHz, CD_3CN) δ 8.96 (s, 1H, NH), 7.42 (br. s, 2H, CH_{Ar}), 7.35 (t, J = 7.7 Hz, 2H, CH_{Ar}), 7.22 (t, J = 7.4 Hz, 2H, CH_{Ar}), 4.26 (t, J = 7.2 Hz, 2H, CH_2), 3.02 (s, 3H, CH_3), 2.37 (s, 3H, CH_3), 1.86–1.78 (m, 2H, CH_2), 1.28 (br. s, 14H, CH_2), 0.89 (t, J = 6.5 Hz, 3H, CH_3). ^{13}C NMR (100 MHz, CD_3CN) δ 9.4, 14.4, 23.5, 27.1, 29.8, 30.1, 30.2, 30.3, 30.4, 32.7, 43.4, 48.9, 124.5, 127.1, 129.9 (2C), 136.4, 139.1, 156.6. HRMS (ESI) m/z: $[M+H]^+$ calcd. for $C_{21}H_{34}N_5O_2S$ 420.2427; found 420.2427.

(*E*)-1-Decyl-*N'*-(ethylsulfonyl)-5-methyl-*N*-phenyl-1*H*-1,2,3-triazole-4-carboximidamide (3q)

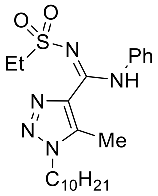

Prepared according to the general procedure from thioamide **1d** (150 mg, 0.42 mmol) and sulfonyl azide **2b** (68 mg, 0.50 mmol) for 33.5 h. White solid, yield 65% (119 mg), mp 115–117 °C. ^{1}H NMR (400 MHz, CD_3CN) δ 9.01 (s br, 1H, NH), 7.40 (br. s, 2H, CH_{Ar}), 7.35 (d, J = 7.7 Hz, 2H, CH_{Ar}), 7.22 (t, J = 7.3 Hz, 1H, CH_{Ar}), 4.26 (t, J = 7.2 Hz, 2H, CH_2), 3.10 (q, J = 7.4 Hz, 2H, CH_2), 2.38 (s, 3H, CH_3), 1.84–1.77 (m, 2H, CH_2), 1.33–1.28 (m, 17H, CH_2+CH_3), 0.89 (t, J = 6.6 Hz, 3H, CH_3). ^{13}C NMR (100 MHz, CD_3CN) δ 8.7, 9.4, 14.4, 23.4, 26.9, 29.7, 30.0, 30.1, 30.2, 30.3, 32.6, 48.7, 50.0, 124.2, 126.9, 129.8 (2C), 136.3, 139.0, 139.1, 156.6. HRMS (ESI) m/z: $[M+H]^+$ calcd. for $C_{22}H_{36}N_5O_2S$ 434.2584; found 434.2592.

(*E*)-1-Decyl-*N'*-((4-fluorophenyl)sulfonyl)-5-methyl-*N*-phenyl-1*H*-1,2,3-triazole-4-carboximidamide (3r)

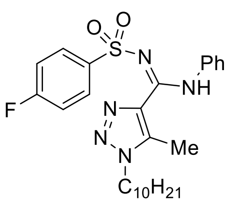

Prepared according to the general procedure from thioamide **1d** (150 mg, 0.42 mmol) and sulfonyl azide **2f** (101 mg, 0.50 mmol) for 14.5 h. White solid, yield 79% (166 mg), mp 127–129 °C. ^{1}H NMR (400 MHz, CD_3CN) δ 9.11 (br. s, 1H, NH), 7.86 (t, J = 6.8 Hz, 2H, CH_{Ar}), 7.31–7.20 (m, 7H, CH_{Ar}), 4.24 (t, J = 7.2 Hz, 2H, CH_2), 2.33 (s, 3H, CH_3), 1.83–1.76 (m, 2H, CH_2), 1.27 (br. s, 14H, CH_2), 0.87 (t, J = 6.4 Hz, 3H, CH_3). ^{13}C NMR (100

MHz, CD_3CN) δ 9.5, 14.4, 23.4, 26.9, 29.7, 30.0, 30.1, 30.2, 30.3, 32.6, 48.8, 116.8 (d, 2C, J = 22.8 Hz), 124.3, 127.2, 129.8 (2C), 130.1 (d, 2C, J = 9.4, Hz), 136.6, 138.6, 138.7, 140.4 (d, J = 2.9 Hz), 156.4, 165.6 (d, J = 250.9 Hz). HRMS (ESI) m/z: $[M+H]^+$ calcd. for $C_{26}H_{35}FN_5O_2S$ 500.2490; found 500.2495.

(*E*)-1-Decyl-5-methyl-*N*-phenyl-*N'*-(phenylsulfonyl)-1*H*-1,2,3-triazole-4-carboximidamide (3s)

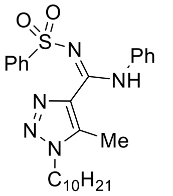

Prepared according to the general procedure from thioamide **1d** (150 mg, 0.42 mmol) and sulfonyl azide **2c** (92 mg, 0.50 mmol) for 20 h. White solid, yield 69% (140 mg), mp 109–111 °C. ^1H NMR (400 MHz, CD_3CN) δ 9.15 (br s, 1H, NH), 7.84 (d, J = 7.3 Hz, 2H, CH_{Ar}), 7.59 (t, J = 7.3 Hz, 1H, CH_{Ar}), 7.52 (t, J = 7.3 Hz, 2H, CH_{Ar}), 7.30–7.20 (m, 5H, CH_{Ar}), 4.24 (t, J = 7.1 Hz, 2H, CH_2), 2.33 (s, 3H, CH_3), 1.84–1.77 (m, 2H, CH_2), 1.28 (s, 14H, CH_2), 0.88 (t, J = 6.6 Hz, 3H, CH_3). ^{13}C NMR (100 MHz, CD_3CN) δ 9.4, 14.4, 23.4, 26.9, 29.7, 30.0, 30.1, 30.2, 30.2, 32.6, 48.7, 124.3, 127.2 (4C), 129.8 (2C), 129.9 (2C), 133.1, 136.6, 138.7, 138.8, 144.0, 156.4. HRMS (ESI) m/z: $[M+H]^+$ calcd. for $C_{26}H_{36}N_5O_2S$ 482.2584; found 482.2585.

General procedure for the preparation of N-sulfonylamidines ***3t-3aa***

To a solution of the corresponding thioamide **1i-l** (1.0 equiv) in 1-propanol (5 mL) sulfonyl azide **2a,c,f** (7.0 equiv) was added and the resulting solution was stirred at reflux for 17.5–31 h. The crude mixture was concentrated under reduced pressure,and the obtained residue was separated by flash chromatography on silica gel (eluent – hexane/EtOAc 1:1 → EtOAc or hexane/EtOAc 1:1 → EtOAc → DCM + 5% EtOH) to afford *N*-sulfonylamidines **3t-3aa**. In order to remove traces of solvents the resulting *N*-sulfonylamidines were suspended in CCl_4 and stirred at reflux for 0.5 h. The solvent was evaporated (the procedure was repeated three times).

(*E*)-*N*-(Morpholino(5-((3-nitrophenyl)amino)-1*H*-1,2,3-triazol-4-yl)methylene)benzenesulfonamide (3t)

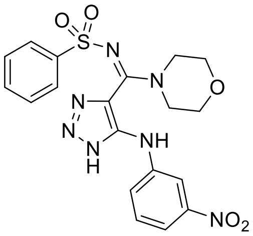

Prepared according to the general procedure from thioamide **1j** (150 mg, 0.45 mmol) and sulfonyl azide **2c** (575 mg, 3.14 mmol) for 17.5 h. Bright yellow solid, yield 78% (160 mg), mp 225–227 °C. ^1H NMR (400 MHz, CD_3CN) δ 12.68 (br. s, 1H, NH), 8.36 (s, 1H, NH), 7.74 (d, J = 7.2 Hz, 1H, H_{Ar}), 7.67 (d, J = 7.3 Hz, 2H, H_{Ar}), 7.62 (d, J = 7.2 Hz, 1H, H_{Ar}), 7.50–7.40 (m, 5H, H_{Ar}), 3.30–3.60 (m, 8H, CH_2). ^{13}C NMR (100 MHz, DMSO-d_6): δ 46.0 (2C), 65.3 (2C), 109.5, 113.6, 121.7, 125.0, 125.7 (2C), 128.3 (2C), 129.8, 131.2, 143.5,

143.6, 145.1, 148.4, 156.3. HRMS (ESI) m/z: $[M+H]^+$ calcd. for $C_{19}H_{20}N_7O_5S$ 458.1241; found 458.1246.

(*E*)-*N*-((5-((4-Nitrophenyl)amino)-1*H*-1,2,3-triazol-4-yl)(pyrrolidin-1-yl)methylene)benzenesulfonamide (3u)

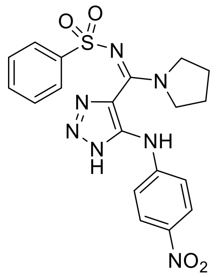

Prepared according to the general procedure from thioamide **1k** (100 mg, 0.31 mmol) and sulfonyl azide **2c** (403 mg, 2.20 mmol) for 31 h. Yellow solid, yield 44% (61 mg), mp 245–247 °C. ^1H NMR (400 MHz, DMSO-d_6) δ 14.94 (br. s, 1H, NH), 9.51 (s, 1H, NH), 8.16 (d, J = 8.8 Hz, 2H, CH_{Ar}), 7.61 (d, J = 7.4 Hz, 2H, CH_{Ar}), 7.53–7.40 (m, 5H, CH_{Ar}), 3.58 (t, J = 5.8 Hz, 2H, CH_2), 3.14 (t, J = 5.7 Hz, 2H, CH_2), 1.92–1.80 (m, 4H, CH_2). ^{13}C NMR (100 MHz, DMSO-d_6): δ 23.9, 24.9, 48.7, 48.9, 114.9, 125.5 (2C), 125.9 (2C), 127.9, 128.5 (2C), 131.3 (2C), 139.0, 143.6, 143.9, 148.8, 154.9. HRMS (ESI) m/z: $[M+H]^+$ calcd. for $C_{19}H_{20}N_7O_4S$ 442.1292; found 442.1299.

(*E*)-*N*-(Morpholino(5-(phenylamino)-1*H*-1,2,3-triazol-4-yl)methylene)methanesulfonamide (3v)

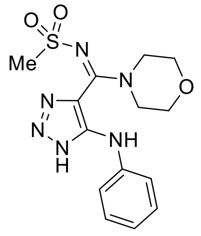

Prepared according to the general procedure from thioamide **1l** (100 mg, 0.34 mmol) and sulfonyl azide **2a** (297 mg, 2.45 mmol) for 26 h. White solid, yield 86% (104 mg), mp 194–196 °C, mp 194–196 °C. ^1H NMR (400 MHz, CD_3CN) δ 12.60 (br. s, 1H, NH), 7.31–7.25 (m, 4H, CH_{Ar}), 7.06 (br. s, 1H, NH), 6.91 (t, J = 6.9 Hz, 1H, CH_{Ar}), 3.61 (br. s, 4H, CH_2), 3.52 (br. s, 4H, CH_2), 2.91 (s, 3H, CH_3). ^{13}C NMR (100 MHz, CD_3CN): δ 43.9, 47.9, 67.1 (2C), 117.6 (2C), 121.9, 126.0, 130.2, 131.2 (2C), 143.7, 157.9. HRMS (ESI) m/z: $[M+H]^+$ calc. for $C_{14}H_{19}N_6O_3S$ 351.1234; found 351.1241.

(*E*)-*N*-(Morpholino(5-((3-nitrophenyl)amino)-1*H*-1,2,3-triazol-4-yl)methylene)methanesulfonamide (3w)

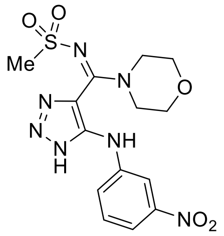

Prepared according to the general procedure from thioamide **1j** (100 mg, 0.30 mmol) and sulfonyl azide **2a** (257 mg, 2.12 mmol) for 17.5 h. Bright yellow solid, yield 97% (115 mg), mp 229–213 °C. ^{1}H NMR (400 MHz, CD_3CN) *δ* 12.74 (s, 1H, NH), 8.30 (s, 1H, NH), 7.72 (d, *J* = 8.0 Hz, 1H, CH_{Ar}), 7.61 (d, *J* = 8.0 Hz, 1H, CH_{Ar}), 7.49–7.45 (m, 2H, CH_{Ar}), 3.65 (br. s, 4H, CH_2), 3.56 (br. s, 4H, CH_2), 2.92 (s, 3H, CH_3). ^{13}C NMR (100 MHz, DMSO-d_6): *δ* 42.9, 46.1 (2C), 66.3 (2C), 109.4, 113.7, 121.7, 125.4, 129.8, 143.6, 145.0, 148.4, 156.5. HRMS (ESI) m/z: $[M+H]^+$ calc. for $C_{14}H_{18}N_7O_5S$ 396.1085; found 396.1093.

(*E*)-*N*-((5-((4-Chlorophenyl)amino)-1*H*-1,2,3-triazol-4-yl)(pyrrolidin-1-yl)methylene)-4-fluorobenzenesulfonamide (3x)

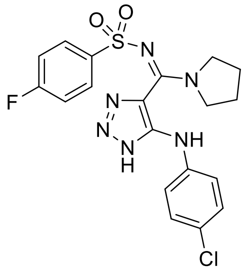

Prepared according to the general procedure from thioamide **1i** (100 mg, 0.32 mmol) and sulfonyl azide **2f** (460 mg, 2.29 mmol) for 31 h. White solid, yield (57%) 84 mg, mp 253–255 °C ^{1}H NMR (400 MHz, DMSO-d_6) *δ* 14.62 (s, 1H, NH), 8.63 (s, 1H, NH), 7.66 (dd, *J* = 8.4, 5.3 Hz, 2H, CH_{Ar}), 7.42 (d, *J* = 8.4 Hz, 2H, CH_{Ar}), 7.21–7.27 (m, 4H, CH_{Ar}), 3.56 (t, *J* = 7.0 Hz, 2H, CH_2), 3.13 (t, *J* = 6.7 Hz, 2H, CH_2), 1.93–1.77 (m, 4H, CH_2). ^{13}C NMR (100 MHz, DMSO-d_6) *δ* 23.9, 24.8, 48.6, 48.7, 115.4 (d, *J* = 22.4 Hz, 2C), 117.3 (2C), 122.8, 126.3, 128.5 (2C), 128.8 (d, *J* = 9.3 Hz, 2C), 140.5 (d, *J* = 2.7 Hz), 141.3, 145.0, 155.4, 163.4 (d, *J* = 249.2 Hz). HRMS (ESI) m/z: $[M+H]^+$ calc. for $C_{19}H_{19}ClFN_6O_2S$ 449.0957; found 449.0963.

(*E*)-*N*-((5-((4-Nitrophenyl)amino)-1*H*-1,2,3-triazol-4-yl)(pyrrolidin-1-yl)methylene)methanesulfonamide (3y)

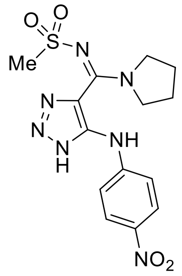

Prepared according to the general procedure from thioamide **1k** (100 mg, 0.31 mmol) and sulfonyl azide **2a** (270 mg, 2.23 mmol) for 31 h. Yellow solid, yield 45% (54 mg), mp 204–206 °C. ^{1}H NMR (400 MHz, DMSO-d_6) δ 15.01 (s, 1H, NH), 9.60 (s, 1H, NH), 8.16 (d, J = 9.2 Hz, 2H, CH_{Ar}), 7.56 (d, J = 9.1 Hz, 2H, CH_{Ar}), 3.57 (t, J = 6.0 Hz, 2H, CH_2), 3.15 (t, J = 6.2 Hz, 2H, CH_2), 2.86 (s, 3H, CH_3), 1.92–1.81 (m, 4H, CH_2)). ^{13}C NMR (100 MHz, DMSO-d_6): δ 23.7, 24.7, 42.8, 48.3, 114.7 (2C), 125.3 (2C), 128.4, 139.0, 143.5, 148.7, 154.8. HRMS (ESI) m/z: $[M+H]^+$ calc. for $C_{14}H_{18}N_7O_4S$ 380.1135; found 380.1134.

(*E*)-*N*-(Morpholino(5-(phenylamino)-1*H*-1,2,3-triazol-4-yl)methylene)benzenesulfonamide (3z)

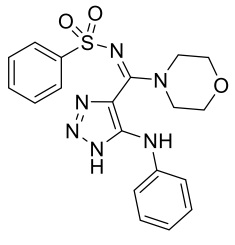

Prepared according to the general procedure from thioamide **1l** (90 mg, (0.31 mmol) and sulfonyl azide **2c** (400 mg, 2.18 mmol) for 26 h. White solid, yield 83% (107 mg), mp 163–165 °C. ^{1}H NMR (400 MHz, DMSO-d_6) δ 14.64 (br. s, 1H, NH), 8.36 (s, 1H, NH), 7.72 (d, J = 7.1 Hz, 2H, H_{Ar}), 7.54–7.45 (m, 3H, H_{Ar}), 7.35 (d, J = 7.9 Hz, 2H, CH_{Ar}), 7.21 (t, J = 7.7 Hz, 2H, CH_{Ar}), 6.83 (t, J = 7.2 Hz, 1H, CH_{Ar}), 3.57 (br. s, 4H, CH_2), 3.44 (br. s, 4H, CH_2). ^{13}C NMR (100 MHz, DMSO-d_6): δ 46.2 (2C), 65.2 (2C), 115.8 (2C), 119.5, 124.8, 125.7 (2C), 128.3 (2C), 128.5 (2C), 156.6, 143.8, 145.7, 142.3, 131.2. HRMS (ESI) m/z: $[M+H]^+$ calc. for $C_{19}H_{21}N_6O_3S$ 413.1390; found 413.1398.

(*E*)-4-Fluoro-*N*-(morpholino(5-((3-nitrophenyl)amino)-1*H*-1,2,3-triazol-4-yl)methylene)benzenesulfonamide (3aa)

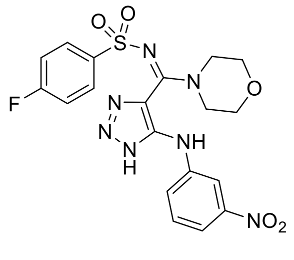

Prepared according to the general procedure from thioamide **1j** (73 mg, 0.22 mmol) and sulfonyl azide **2f** (308 mg, 1.53 mmol) for 17.5 h. White solid, yield 61%, (63 mg), mp 223–225 °C. ^{1}H NMR (600 MHz, DMSO-d_6) δ 15.00 (br. s, 1H, NH), 9.43 (s, 1H, NH), 8.15 (d, J = 8.0 Hz, 2H, CH_{Ar}), 7.67–7.69 (m, 2H, CH_{Ar}), 7.52 (d, J = 8.0 Hz, 2H, CH_{Ar}), 7.24–7.27 (m, 2H, CH_{Ar}), 3.50–3.59 (m, 8H, CH_2). ^{13}C NMR (150 MHz, DMSO-d_6) δ 45.6, 47.4, 65.4 (2C), 109.5, 113.7, 115.4 (d, J = 21.8 Hz, 2C), 121.7, 124.7, 127.7 (d, J = 8.5 Hz, 2C), 129.9, 140.0, 143.4, 145.0, 148.4, 156.4, 163.4 (d, J = 252.7 Hz). HRMS (ESI) m/z: $[M+H]^+$ calc. for $C_{19}H_{19}FN_7O_5S$ 476.1147; found 476.1150.

General procedure for the preparation of N-sulfonylamidines ***3ab, 3ac***

Method A. A mixture of thioamide **1m** (1.0 equiv) and sulfonyl azide **2a,c** (5.0 equiv**)** was dissolved in 1-propanol (2 mL). The reaction mixture was stirred under reflux for 8–11.5 h. The obtained solution was cooled and the solvent was evaporated under reduced pressure. The crude mixture was purified by flash chromatography on silica gel (eluent – hexane/EtOAc 3:7 → EtOAc) to afford *N*-sulfonyl amidines **3ab, 3ac**.

(*E*)-2-Amino-*N'*-(methylsulfonyl)thiazole-4-carboximidamide (3ab)

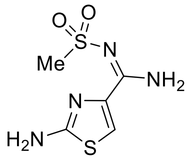

Prepared according to the general procedure from thioamide **1m** (100 mg, 0.63 mmol) and sulfonyl azide **2a** (579 mg, 4.78 mmol) for 11.5 h. White solid, yield 96% (198 mg), mp 199–201 °C. ^{1}H NMR (400 MHz, DMSO-d_6) δ 7.93 (s, 2H, NH_2), 7.38 (s, 1H, $CH_{thiaz.}$), 7.23 (s, 2H, NH_2), 2.95 (s, 3H, CH_3). ^{13}C NMR (100 MHz, DMSO-d_6): δ 41.4, 113.8, 143.8, 155.9, 168.4. HRMS (ESI) m/z: $[M+H]^+$ calc. for $C_5H_9N_4O_2S_2$ 221.0161; found 221.0171.

Method B. A mixture of thioamide **1m** (100 mg, 0.63 mmol, 1.0 equiv) and sulfonyl azide **2a** (191 mg, 1.58 mmol, 2.5 equiv) was heated at 100 °C in an oil bath for 8 h. The reaction mass darkens over time. The crude mixture was purified by flash chromatography on silica gel (eluent – hexane/EtOAc 3:7 → EtOAc) to obtain amidine **3ab** in 41% (85 mg) yield.

(*E*)-2-Amino-*N'*-(phenylsulfonyl)thiazole-4-carboximidamide (3ac)

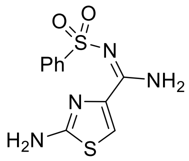

Prepared according to the general procedure from thioamide **1m** (150 mg, 0.94 mmol) and sulfonyl azide **2c** (579 mg, 3.16 mmol) for 6 h. White solid, yield 77% (862 mg), mp 235–237 °C. ^{1}H NMR (400 MHz, DMSO-d_6) δ 8.17 (s, 1H, NH_2), 8.09 (s, 1H, NH_2), 7.88 (d, J = 7.5, 2H, CH_{Ar}), 7.62–7.53 (m, 3H, CH_{Ar}), 7.43 (s, 1H, $CH_{thiaz.}$), 7.24 (s, 2H, NH_2).

^{13}C NMR (100 MHz, DMSO-d_6): δ 114.7, 125.9, 128.9, 132.1, 142.6, 143.6, 156.2, 168.4. HRMS (ESI) m/z: $[M+H]^+$ calc. for $C_{10}H_{11}N_4O_2S_2$ 283.0318; found 283.0332.

General procedure for the preparation of N-sulfonylamidines ***3ad-3af***

Method A. A mixture of thioamide **1n** (1.0 equiv) and sulfonyl azide **2a,c,f** (2.5 equiv) was heated at 100 °C for 22 h. The reaction mixture was cooled, and a mixture of chloroform and acetonitrile (1:1) was added. The crude mixture was heated after dissolution and concentrated under reduced pressure. The obtained residue was separated by flash chromatography on silica gel (eluent – hexane/EtOAc 7:3 → 4:6 (**3ae,3af**) or hexane/EtOAc 5:5 → 3:7 (**3ad**) to afford *N*-sulfonylamidines **3ad-3af**. The isolated *N*-sulfonylamidines **3ad-3af** were washed with cold ethanol additionally. The precipitate was collected by filtration and dried in a vacuum desiccator over P_4O_{10}. Amidine **3ad** was dissolved in a minimal volume of DMSO and water was added to the solution. DCM was added to the resulting solution and amidine was extracted (3 × 5 mL).The organic layer was collected, washed with water (2 × 10 mL) and concentrated *in vacuum*. The resulting amidine **3ad** was dried in a vacuum desiccator over P_4O_{10}.

(*Z*)-5-Methyl-*N'*-(methylsulfonyl)-3-phenylisoxazole-4-carboximidamide (3ad)

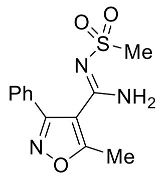

Prepared according to the general procedure from thioamide **1n** (100 mg, 0.46 mmol) and sulfonyl azide **2a** (141 mg, 1.16 mmol) for 22 h. Colorless semisolid, yield 73% (64 mg). ^1H NMR (400 MHz, DMSO-d_6) δ 8.75 (s, 1H, NH), 8.27 (s, 1H, NH), 7.66 (dd, J = 6.4, 2.8 Hz, 2H, CH_{Ar}), 7.53–7.51 (m, 3H, CH_{Ar}), 2.86 (s, 3H, CH_3), 2.53 (s, 3H, CH_3). ^{13}C NMR (100 MHz, DMSO-d_6): δ 11.8, 41.4, 111.4, 127.8 (2C), 127.9 (2C), 128.8, 130.1, 157.1, 160.1, 170.1. HRMS (ESI) m/z: $[M+H]^+$ calc. for $C_{12}H_{14}N_3O_3S$ 280.0750; found 280.0762.

Method B. A mixture of thioamide **1n** (100 mg, 0.46 mmol, 1.0 equiv) and sulfonyl azide **2a** (198 mg, 2.3 mmol, 5 equiv) was dissolved in 1-propanol-1 (3 mL). The reaction mixture was stirred under reflux for 22 h. The obtained solution was cooled and the solvent was evaporated under reduced pressure. The residue was separated by flash chromatography on silica gel (eluent – hexane/EtOAc 5:5 → 3:7) to obtain amidine **3ad** in 50% (44 mg) yield.

(*Z*)-5-Methyl-3-phenyl-*N'*-(phenylsulfonyl)isoxazole-4-carboximidamide (3ae)

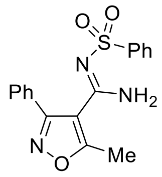

Prepared according to the general procedure from thioamide **1n** (83 mg, 0.38 mmol) and sulfonyl azide **2c** (174 mg, 0.95 mmol) for 22 h. White solid, yield 49% (64 mg), mp 205–207 °C. ^{1}H NMR (400 MHz, DMSO-*d*$_6$) *δ* 8.94 (s, 1H, NH), 8.55 (s, 1H, NH), 7.75 (d, *J* = 7.5 Hz, 2H, CH_{Ar}), 7.64 (t, *J* = 7.3 Hz, 1H, CH_{Ar}), 7.54 (t, *J* = 7.4 Hz, 2H, CH_{Ar}), 7.47–7.42 (m, 3H, CH_{Ar}), 7.33 (t, *J* = 7.5 Hz, 2H, CH_{Ar}), 2.41 (s, 3H, CH_3). ^{13}C NMR (100 MHz, DMSO-*d*$_6$): *δ* 11.6, 111.2, 126.2 (2C), 127.5 (2C), 127.7, 128.6, 128.9 (2C), 129.9 (2C), 132.3, 141.8, 157.7, 159.8, 169.9. HRMS (ESI) m/z: $[M+H]^+$ calc. for $C_{17}H_{16}N_3O_3S$ 342.0907; found 342.0920.

(*Z*)-*N*'-((4-Fluorophenyl)sulfonyl)-5-methyl-3-phenylisoxazole-4-carboximidamide (3af)

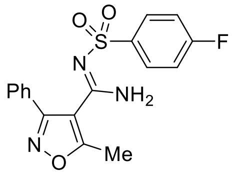

Prepared according to the general procedure from thioamide **1n** (150 mg, 0.69 mmol) and sulfonyl azide **2f** (346 mg, 1.72 mmol) for 22 h. White solid, yield 76% (188 mg), mp 207–209 °C. ^{1}H NMR (400 MHz, DMSO-*d*$_6$) *δ* 9.00 (br. s, 1H, NH), 8.60 (br. s, 1H, NH), 7.79 (dd, *J* = 8.5, 5.4 Hz, 2H, CH_{Ar}), 7.48–7.43 (m, 3H, CH_{Ar}), 7.35 (t, *J* = 7.8 Hz, 4H, CH_{Ar}), 2.44 (s, 3H, CH_3). ^{13}C NMR (100 MHz, DMSO-*d*$_6$): *δ* 11.6, 111.1, 115.9 (d, *J* = 22.6 Hz, 2C), 127.4 (2C), 127.7, 128.6 (2C), 129.2 (d, *J* = 9.4 Hz, 2C), 129.9, 138.3 (d, *J* = 2.9 Hz), 157.8, 159.9, 164.0 (d, *J* = 250.5 Hz), 170.0. HRMS (ESI) m/z: $[M+H]^+$ calc. for $C_{17}H_{15}FN_3O_3S$ 360.0813; found 360.0826.

(*Z*)-5-Methyl-*N*'-((4-nitrophenyl)sulfonyl)-3-phenylisoxazole-4-carboximidamide (3ag)

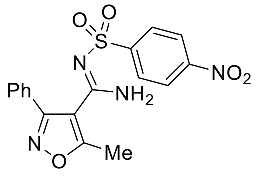

Prepared according to the general procedure from thioamide **1n** (100 mg, 0.46 mmol) and sulfonyl azide **2g** (261 mg, 1.14 mmol) for 22 h. White solid, yield 66% (117 mg), mp 195–197 °C. ^{1}H NMR (400 MHz, DMSO-*d*$_6$) *δ* 9.17 (s, 1H, NH), 8.83 (s, 1H, NH), 8.28 (d, *J* = 8.8 Hz, 2H, CH_{Ar}), 7.90 (d, *J* = 8.8 Hz, 2H, CH_{Ar}), 7.44–7.32 (m, 5H, CH_{Ar}), 2.45 (s, 3H, CH_3). ^{13}C NMR (100 MHz, DMSO-*d*$_6$): *δ* 11.6, 110.8, 124.1 (2C), 127.3 (2C), 127.5 (2C), 127.6, 128.5 (2C), 129.8, 147.3, 149.3, 158.4, 159.8, 170.0. EIMS (m/z): 386 $[M]^+$ (47), 321 (36), 200 (20), 143 (37), 77 (100). Anal. Calcd for $C_{17}H_{14}N_4O_5S$: C, 52.85; H, 3.65; N, 14.50; found: C, 52.64; H, 3.93; N, 14.37.

General procedure for the preparation of N-sulfonylamidines ***3ah*****,*****3ai***

A mixture of thioamide **1o** (1.0 equiv) and the sulfonyl azide **2a,c** (5.0 equiv**)** was dissolved in 1-butanol (4 mL). The reaction mixture was stirred under reflux for 28 h. The

obtained solution was cooled and 1-butanol was evaporated under reduced pressure. The crude mixture was purified by flash chromatography on silica gel (eluent – hexane/EtOAc 8:2 → 6:4) to afford *N*-sulfonyl amidines **3ah,3ai**. The isolated products **3ah,3ai** were washed with cold ethanol additionally. The precipitate was collected by filtration and dried in a vacuum desiccator over P_4O_{10}.

(*Z*)-5-Methyl-*N'*-(methylsulfonyl)-*N*,3-diphenylisoxazole-4-carboximidamide (3ah)

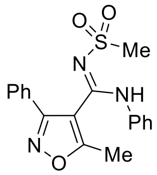

Prepared according to the general procedure from thioamide **1o** (129 mg, 0.44 mmol) and azide **2a** (270 mg, 2.20 mmol) for 28 h. White solid, yield 45% (70 mg), mp 169–171 °C. ^{1}H NMR (400 MHz, CD_3OD) δ 7.78 (t, *J* = 6.8 Hz, 2H, CH_{Ar}), 7.61 (d, *J* = 7.9 Hz, 2H, CH_{Ar}), 7.45–7.36 (m, 5H, CH_{Ar}), 7.21 (t, *J* = 7.0 Hz, 2H, CH_{Ar}), 2.97 (s, 3H, CH_3), 2.56 (s, 3H, CH_3). ^{13}C NMR (100 MHz, DMSO-d_6): δ 11.9, 42.9, 110.2, 121.6 (2C), 125.4, 127.2 (2C), 128.1, 128.8 (2C), 128.9 (2C), 130.1, 137.9, 153.4, 159.7, 169.0. HRMS (ESI) m/z: $[M+H]^+$ calc. for $C_{18}H_{18}N_3O_3S$ 356.1063; found 356.1078.

(*Z*)-5-Methyl-*N*,3-diphenyl-*N'*-(phenylsulfonyl)isoxazole-4-carboximidamide (3ai)

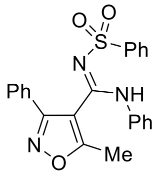

Prepared according to the general procedure from thioamide **1o** (135 mg, 0.46 mmol) and azide **2c** (420 mg, 2.30 mmol) for 28 h. White solid, yield 38% (103 mg), mp 158–160 °C. ^{1}H NMR (400 MHz, DMSO-d_6) δ 10.93 (s, 1H, NH), 7.66–7.45 (m, 12H, CH_{Ar}), 7.37 (t, *J* = 7.7 Hz, 2H, CH_{Ar}), 7.20 (t, *J* = 7.3 Hz, 1H, CH_{Ar}), 2.36 (s, 3H, CH_3). ^{13}C NMR (100 MHz, DMSO-d_6): δ 11.6, 109.8, 121.8 (2C), 125.8, 126.0 (2C), 127.0 (2C), 128.0, 128.8, 128.9 (2C), 129.0 (4C), 130.1, 132.2, 142.4, 154.2, 159.6, 169.0. HRMS (ESI) m/z: $[M+H]^+$ calc. for $C_{23}H_{20}N_3O_3S$ 418.1220; found 418.1237.

*General procedure for the preparation of bis-N-sulfonylamidines **3aj-3an***

Method A. A mixture of thioamide **1p** (1.0 equiv) and the corresponding sulfonyl azide **2a,c-f** (2.5 equiv) was heated at 100 °C in anoil bath for 8.5–10.5 h. The reaction mixture was cooled, and a mixture of chloroform and acetonitrile (1:1) was added. The crude mixture was heated until dissolution and concentrated under reduced pressure, and the obtained residue was separated by flash chromatography on silica gel (eluent – hexane/EtOAc 5:5 → 2:8) to afford *N*-sulfonylamidines **3aj-3an**.

(2Z,6Z)-*N*'2,*N*'6-Bis(methylsulfonyl)pyridine-2,6-bis(carboximidamide)

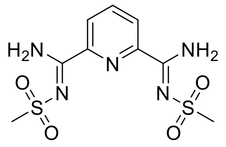

Prepared according to the general procedure from thioamide **1p** (100 mg, 0.51 mmol) and sulfonyl azide **2a** (156 mg, 1.27 mmol) for 10.5 h. White solid, yield 77% (125 mg), mp 263–265 °C. ^{1}H NMR (400 MHz, DMSO-*d*$_6$) δ 9.75 (s, 2H, NH_2), 8.37 (d, *J* = 7.9 Hz, 2H, $CH_{pyrid.}$), 8.31 (s, 2H, NH_2), 8.19 (t, *J* = 7.9 Hz, 1H, $CH_{pyrid.}$), 3.07 (s, 6H, CH_3). ^{13}C NMR (100 MHz, DMSO-*d*$_6$): δ 41.4 (2C), 125.8 (2C), 139.7, 148.5 (2C), 157.5 (2C). EIMS (m/z): EIMS (m/z): 319 [M]$^+$ (18), 225 (100), 130 (72). Anal. Calcd for $C_9H_{13}N_5O_4S_2$: C, 33.85; H, 4.10; N, 21.93; found: C, 34.85; H, 3.86; N, 22.09.

(2*Z*,6*Z*)-*N*'2,*N*'6-Bis(phenylsulfonyl)pyridine-2,6-bis(carboximidamide) (3ak)

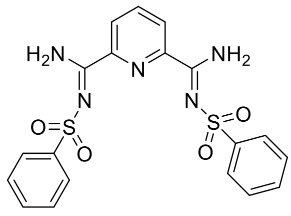

Prepared according to the general procedure from thioamide **1p** (100 mg, 0.51 mmol) and sulfonyl azide **2c** (232 mg, 1.27 mmol) for 8.5 h. White solid, yield 84% (190 mg), mp 186–188 °C. ^{1}H NMR (400 MHz, DMSO-*d*$_6$) δ 9.91 (s, 2H, NH_2), 8.56 (s, 2H, NH_2), 8.32 (d, *J* = 7.9 Hz, 2H, $CH_{pyrid.}$), 8.12 (t, *J* = 7.9 Hz, 1H, $CH_{pyrid.}$), 7.98 (d, *J* = 7.5 Hz, 4H, CH_{Ar}), 7.64–7.55 (m, 6H, CH_{Ar}). ^{13}C NMR (100 MHz, DMSO-*d*$_6$): δ 126.1 (4C), 126.2 (2C), 129.0 (4C), 132.4 (2C), 139.9, 142.2 (2C), 148.3 (2C), 157.9 (2C).HRMS (ESI) m/z: [M+H]$^+$ calc. for $C_{19}H_{18}N_5O_4S_2$ 444.0795; found 444.0813.

Method B. A mixture of thioamide **1p** (100 mg, 0.51 mmol, 1.0 equiv) and sulfonyl azide **2c** (232 mg, 1.27 mmol, 4.0 equiv) was heated at reflux in 1-propanol (2 mL) for 11 h. Solvent was evaporated under reduced pressure. The crude mixture was purified by flash chromatography on silica gel (eluent – hexane/EtOAc 5:5 → 2:8) to obtain *N*-sulfonylamidine **3ak** in 84% (190 mg) yield.

(2*Z*,6*Z*)-*N*'2,*N*'6-Ditosylpyridine-2,6-bis(carboximidamide) (3al)

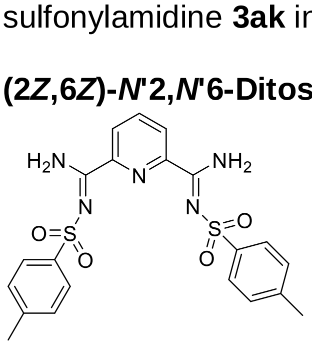

Prepared according to the general procedure from thioamide **1p** (150 mg, 0.76 mmol) and sulfonyl azide **2d** (378 mg, 1.92 mmol) for 10.5 h. White solid, yield 90% (236 mg), mp 258–260 °C (decomp.). ^{1}H NMR (400 MHz, DMSO-*d*$_6$) δ 9.85 (s, 2H, NH_2), 8.49 (s,

2H, NH_2), 8.29 (d, J = 7.9 Hz, 2H, $CH_{pyrid.}$), 8.11 (t, J = 7.9 Hz, 1H, $CH_{pyrid.}$), 7.86 (d, J = 8.2 Hz, 4H, CH_{Ar}), 7.36 (d, J = 8.1 Hz, 4H, CH_{Ar}), 2.35 (s, 6H, CH_3). ^{13}C NMR (100 MHz, DMSO-d_6): δ20.9 (2C), 126.1 (2C), 126.2 (4C), 129.4 (4C), 139.3 (2C), 139.8, 142.6 (2C), 148.4 (2C), 157.7 (2C). HRMS (ESI) m/z: $[M+H]^+$ calc. for $C_{21}H_{22}N_5O_4S_2$ 472.1108; found 472.1125.

(2Z,6Z)-*N*'2,*N*'6-Bis((4-methoxyphenyl)sulfonyl)pyridine-2,6-bis(carboximidamide)

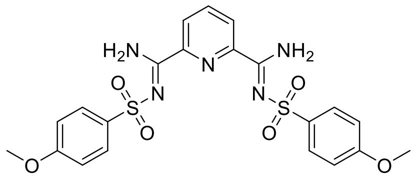

Prepared according to the general procedure from thioamide **1p** (100 mg, 0.51 mmol) and sulfonyl azide **2e** (273 mg, 1.27 mmol) for 10.5 h. White solid, yield 71% (181 mg), mp 222–224 °C. ^{1}H NMR (400 MHz, DMSO-d_6) δ 9.81 (s, 2H, NH_2), 8.45 (s, 2H, NH_2), 8.29 (d, J = 7.9 Hz, 2H, $CH_{pyrid.}$), 8.11 (t, J = 7.9 Hz, 1H, $CH_{pyrid.}$), 7.91 (d, J = 8.8 Hz, 4H, CH_{Ar}), 7.07 (d, J = 8.8 Hz, 4H, CH_{Ar}), 3.81 (s, 6H, CH_3). ^{13}C NMR (100 MHz, DMSO-d_6): δ 55.6 (2C), 114.1 (2C), 126.0 (4C), 128.3 (4C), 134.0 (2C), 139.8, 148.4 (2C), 157.5 (2C), 162.2 (2C). EIMS (m/z): 252 (39), 251 (46), 171 (53), 77 (100). Anal. Calcd for $C_{21}H_{21}N_5O_6S_2$: C, 50.09; H, 4.20; N, 13.91; found: C, 50.37; H, 4.05; N, 13.72.

(2Z,6Z)-*N*'^{2},*N*'^{6}-Bis((4-fluorophenyl)sulfonyl)pyridine-2,6-bis(carboximidamide) (3an)

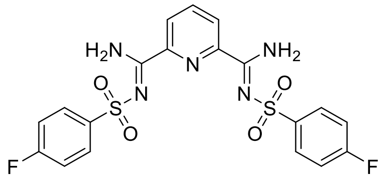

Prepared according to the general procedure from thioamide **1p** (100 mg, 0.51 mmol) and sulfonyl azide **2f** (255 mg, 1.27 mmol) for 10.5 h. White solid, yield 82% (199 mg), mp 173–175 °C. ^{1}H NMR (400 MHz, DMSO-d_6) δ 9.92 (s, 2H, NH_2), 8.57 (s, 2H, NH_2), 8.32 (d, J = 7.8 Hz, 2H, $CH_{pyrid.}$), 8.15–8.03 (m, 5H, $CH_{pyrid.}$ + CH_{Ar}), 7.40 (t, J = 8.6 Hz, 4H, CH_{Ar}). ^{13}C NMR (100 MHz, DMSO-d_6): δ 116.1 (d, J = 22.6 Hz, 4C), 126.2 (2C), 129.1 (d, J = 9.4 Hz, 4C), 138.6 (d, J = 3.0 Hz, 2C), 139.9, 148.3 (2C), 157.9 (2C), 164.1 (d, J = 250.7 Hz, 2C). EIMS (m/z): 239 (41), 159 (30), 111 (90), 95 (100). Anal. Calcd for $C_{19}H_{15}F_2N_5O_4S_2$: C, 47.60; H, 3.15; N, 14.61; found: C, 47.27; H, 3.44; N, 13.89.

General procedures for the preparation 1H-1,2,3-triazole-4-carboxamides ***4c,d***

Sodium 4-acetyl-1-phenyl-1*H*-1,2,3-triazol-5-olate (**5**) (1.0 equiv.) was suspended in 1-propanol (15 mL) and the aproapriate amine hydrochloride (1.1–1.2 equiv.) was added. The reaction mixture was stirred at reflux for 12 h and concentrated *in vacuo*. After cooling the precipitate was filtered off and washed with cold ethanol and water and dried in a vacuum desiccator over P_4O_{10}.

1-Butyl-5-methyl-*N*-phenyl-1*H*-1,2,3-triazole-4-carboxamide (4c)

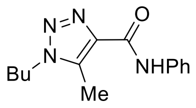

Prepared according to the general procedure from sodium 4-acetyl-1-phenyl-1*H*-1,2,3-triazol-5-olate (**5**) (5.00 g, 30.65 mmol) and *n*-butylamine hydrochloride (2.92 g, 26.64 mmol). White solid, yield 64% (3.692 g), mp 103–104 °C. ^{1}H NMR (400 MHz, DMSO-d_6) δ 10.29 (s, 1 H, NH), 7.83 (d, J = 8.0 Hz, 2H, H_{Ar}), 7.32 (t, J = 8.0 Hz, 2H, H_{Ar}), 7.08 (t, J = 8.0 Hz, 1H, H_{Ar}), 4.35 (t, J =7.1 Hz, 2H, CH_2), 2.58 (s, 1H, CH_3), 1.82–1.74 (m, 2H, CH_2), 1.34–1.25 (m, 2H, CH_2), 0.91 (t, J =7.3 Hz, 3H, CH_3). ^{13}C NMR (100 MHz, DMSO-d_6): δ 8.3, 13.3, 19.1, 31.0, 47.0, 120.3 (2C), 123.5, 128.5 (2C), 136.6, 138.0, 138.7, 159.7. HRMS (ESI) m/z: $[M+H]^+$ calcd. for $C_{14}H_{19}N_4O$ 259.1553; found 259.1563.

1-Decyl-5-methyl-*N*-phenyl-1*H*-1,2,3-triazole-4-carboxamide (4d)

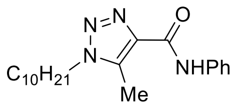

Prepared according to the general procedure from sodium 4-acetyl-1-phenyl-1*H*-1,2,3-triazol-5-olate (**5**) (5.00 g, 30.65 mmol) and *n*-decylamine hydrochloride (3.786 g, 19.54 mmol). White solid, yield 42% (2.585 g), mp 65–67 °C. ^{1}H NMR (400 MHz, $CDCl_3$): δ 9.00 (s, 1H, NH), 7.68 (d, J = 7.9 Hz, 2H, CH_{Ar}), 7.35 (t, J = 7.8 Hz, 2H, CH_{Ar}), 7.12 (t, J =7.4 Hz, 1H, CH_{Ar}), 4.27 (t, J =7.3 Hz, 2H, CH_2), 2.65 (s, 3H, CH_3), 1.85–1.91 (m, 2H, CH_2), 1.25–1.34 (m, 14H, $7CH_2$), 0.87 (t, J = 6.6 Hz, 3H, CH_3). ^{13}C NMR (100 MHz, $CDCl_3$): δ 8.9, 14.2, 22.8, 26.6, 29.1, 29.3, 29.5, 29.6, 29.8, 31.9, 48.1, 119.9 (2C), 124.3, 129.1 (2C), 136.3, 137.9, 138.5, 159.6. HRMS (ESI) m/z: $[M+H]^+$ calcd. for $C_{20}H_{31}N_4O$ 343.2492; found 343.2505.

Table 1. Crystal data and structure refinement

Compound	3e	3t	3ag
formula	$C_{18}H_{19}N_5O_3S$	$C_{19}H_{19}N_7O_5S \cdot C_4H_8O_2$	$C_{17}H_{14}N_4O_5S$
FW	385.44	545.58	386.38
crystal system	Triclinic	Triclinic	Monoclinic
space group	P^{i}	P^{i}	$P2_1/n$
a/Å	7.1372(6)	9.4172(8)	10.210 (6)
b/Å	7.2212(5)	11.4155(10)	7.242 (6)
c/Å	18.9343(12)	13.0409(9)	26.864 (15)
α/deg	89.548(6)	97.171(6)	90.00
β/deg	85.809(5)	96.461(6)	97.45(5)
γ/deg	69.902(7)	109.787(8)	90.00
$V/Å^3$	913.83(12)	1290.72(18)	1970 (2)
Z	2	2	4
$D_c/g\ cm^{-3}$	1.401	1.404	1.303
μ/mm^{-1}	0.21	0.18	1.77
$R[F^2 > 2\sigma(F^2)]$	0.058	0.063	0.046
$wR(F^2)$	0.145	0.222	0.132
GOF	1.006	1.008	1.009

References

1. Naito, T; Nakagawa, S.; Okumura, J.; Konishi, M.; Kawaguchi, H. *J. Antibiot*. **1965**, 18, 145–157.
2. Fusco, Q. *Gazz. Chim. Ital*. **1937**, 67, 589–599.
3. Evstratova, M. I.; Prokopov, A. A.; Ivanova, I. L.; Markova, I. G.; Tubina, I. S.; Anisimova, O. S.; Skvirskaya, N. V.; Kan, I. I.; Suvorov, B. V.; Yakhontov, L. N. *Pharm. Chem. J.* **1985**, 19, 126–129 [*Khim.-Farm. Zhurn*. **1985**, 19, 196–199]
4. Sanghvi, Y.; Larson, S.; Robins, R.; Revankar, G.; Gupta, P.; George, R. D.; Dalley, N. K. *J. Heterocycl, Chem.* **1988**, 25, 623–633.
5. Khazhieva, I.; Glukhareva, T.; El'tsov, O.; Morzherin, Yu.; Minin, A.; Pozdina, V.; Ulitko, M. *Pharm. Chem. J*. **2015**, 49, 296–300.
6. Ilkin, V. G.; Dianova, L. N.; Bakulev, V. A.; Berseneva, V. S.; Saveliev, D. A.; Beryozkina, T. V. *Chem. Heterocycl. Compd*. **2020** (accepted for publications).
7. Berseneva, V. S; Morzherin, Y. Y.; Dehaen, W.; Luyten, I.; Bakulev, V. A. *Tetrahedron*, **2001**, 57, 2179–2184.
8. Baker, A. T.; Singh, P.; Vignevich, V. *Austral. J. Chem*. **1991**, 44, 1041–1048.
9. Kosterina, M. F.; Morzherin, Yu. Yu.; Tkachev, A. V.; Rybalova, T. V.; Gatilov, Yu. V.; Bakulev, V. A. *Russ. Chem. Bull.* **2002**, 51, 653–658.
10. Kang, T.; Kim, H.; Kim, J. C.; Chang, S. *Chem. Commun*. **2014**, 50, 12073–12075.
11. Lima-Neto, R. G.; Cavalcante, N. N. M.; Srivastava, R. M.; Mendonca Jr., F. J. B.; Wanderley, A. G.; Neves, R. P.; Dos Anjos, J. V. *Molecules* **2012**, 17, 5882–5892.
