# Supplementary material for: Regioselective synthesis of heterocyclic N-sulfonyl amidines from heteroaromatic thioamides and sulfonyl azides
Source: Beilstein J Org Chem. 2020 Dec 1;16:2937–47. doi: 10.3762/bjoc.16.243 (PMC7722631; doi:10.3762/bjoc.16.243)
Supplement: File 2 — Copies of NMR spectra of all new compounds. [file Beilstein_J_Org_Chem-16-2937-s002.pdf]

BEILSTEIN JOURNAL OF ORGANIC CHEMISTRY

Supporting Information

for

Regioselective synthesis of heterocyclic *N*-sulfonyl amidines from heteroaromatic thioamides and sulfonyl azides

Vladimir Ilkin, Vera Berseneva, Tetyana Beryozkina, Tatiana Glukhareva, Lidia Dianova, Wim Dehaen, Eugenia Seliverstova and Vasiliy Bakulev

Beilstein J. Org. Chem. **2020,** *16,* 2937–2947. doi:10.3762/bjoc.16.243

Copies of NMR spectra of all new compounds

License and Terms: This is a supporting information file under the terms of the Creative Commons Attribution License (https://creativecommons.org/licenses/by/4.0). Please note that the reuse, redistribution and reproduction in particular requires that the author(s) and source are credited and that individual graphics may be subject to special legal provisions.
The license is subject to the *Beilstein Journal of Organic Chemistry* terms and conditions: (https://www.beilstein-journals.org/bjoc/terms)

NMR spectra

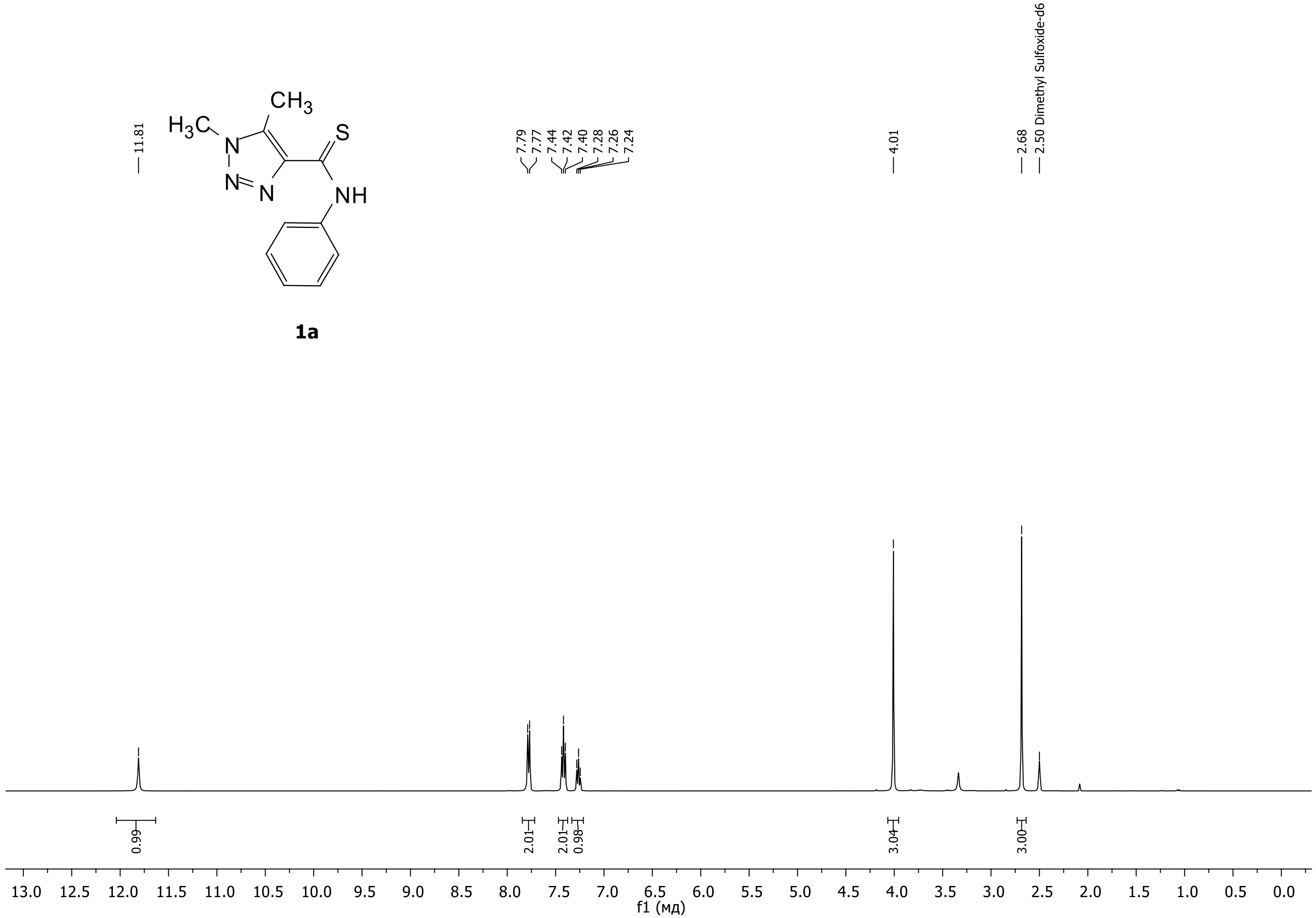

The ^{1}H NMR (400 MHz, DMSO-d_6) spectrum of compound **1a**.

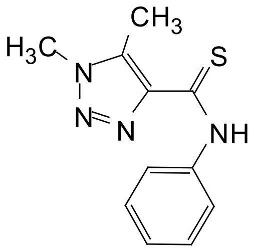

1a

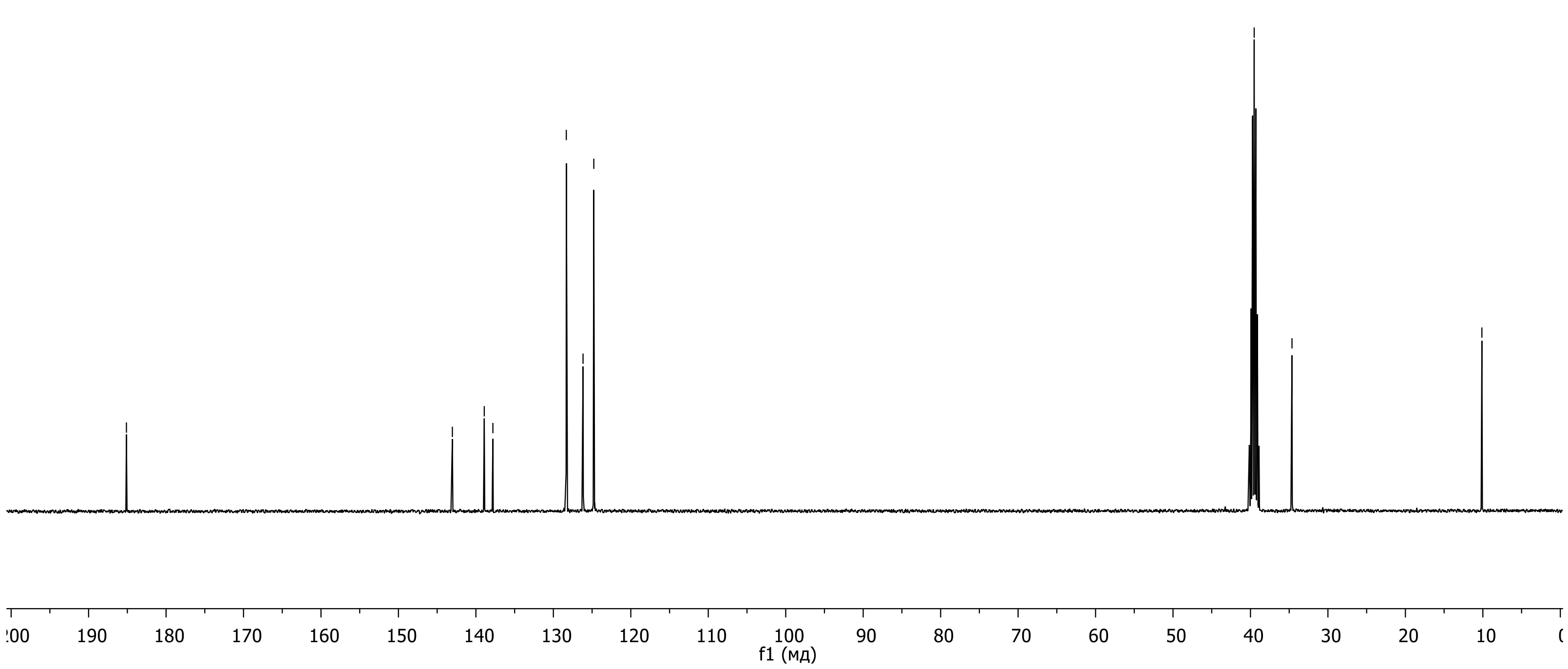

The ^{13}C NMR (100 MHz, DMSO-d_6) spectrum of compound **1a**.

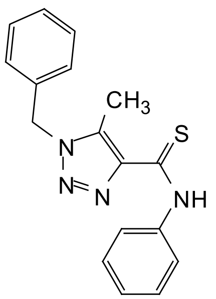

1b

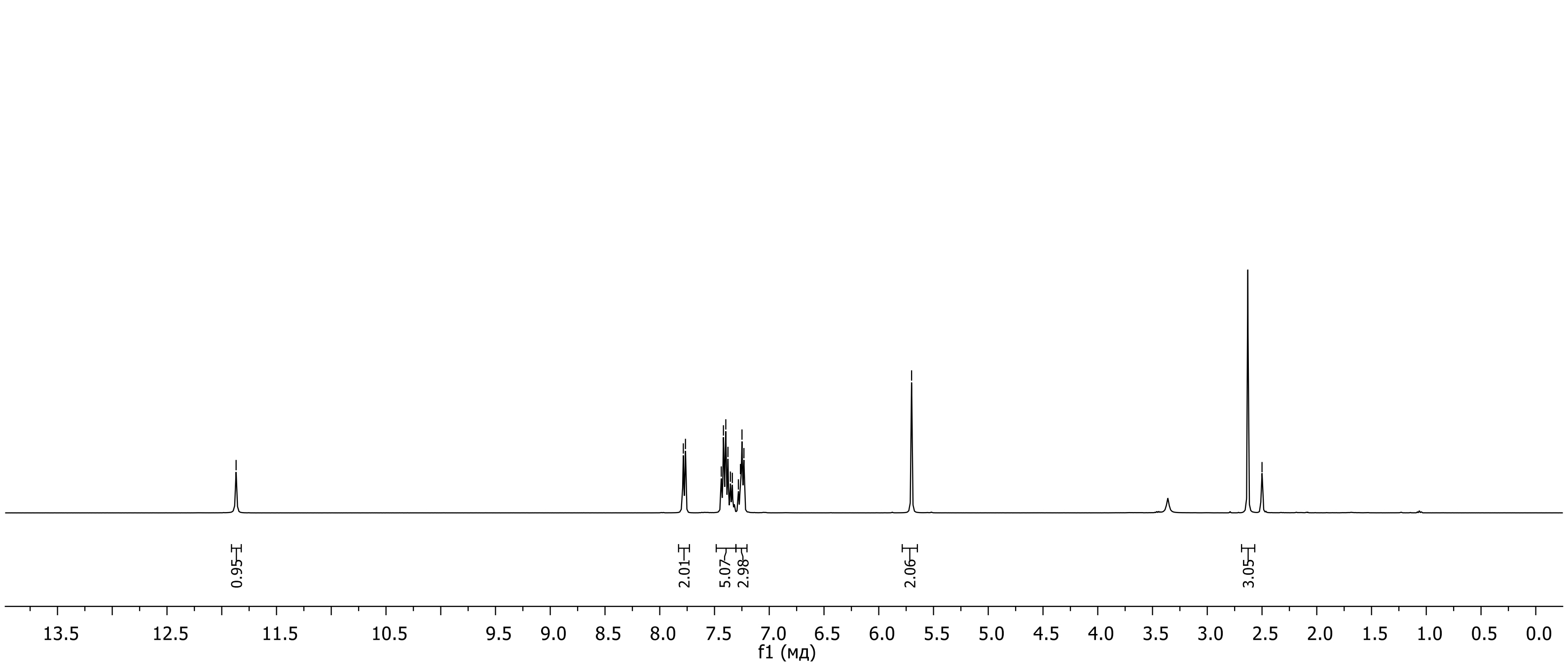

The ^1H NMR (400 MHz, DMSO-d_6) spectrum of compound **1b**.

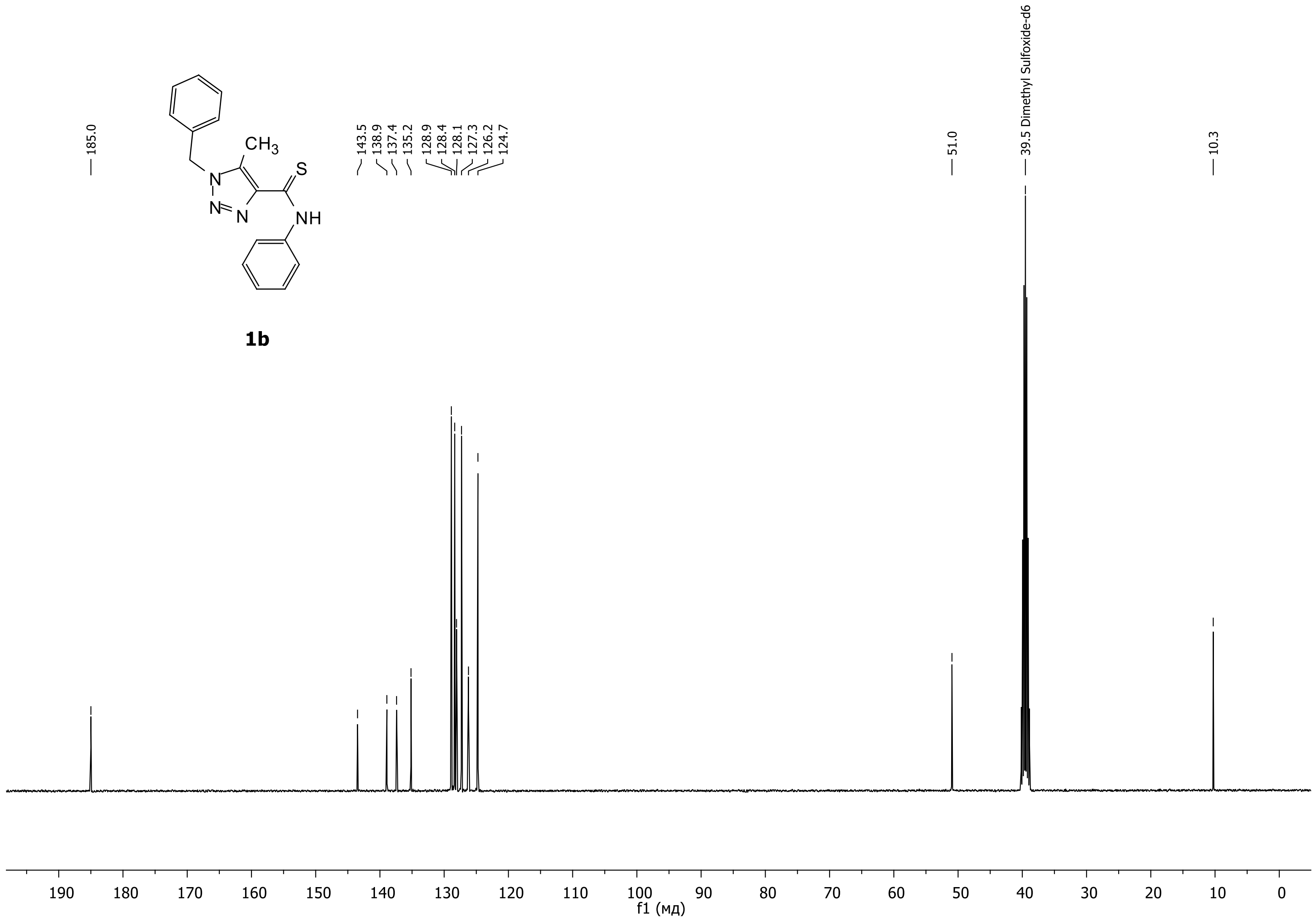

The ^{13}C NMR (100 MHz, DMSO-d_6) spectrum of compound **1b**.

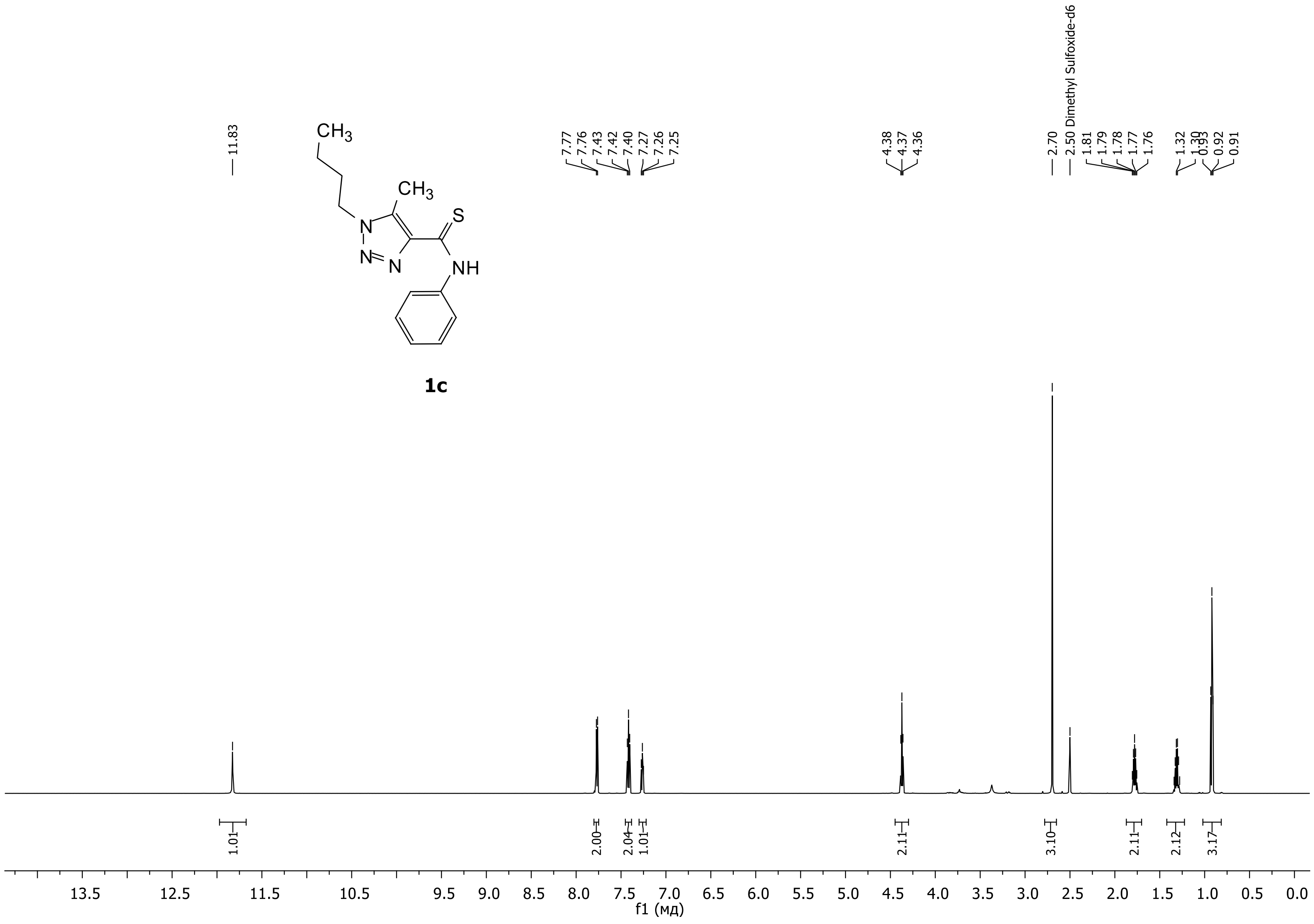

The ^{1}H NMR (400 MHz, DMSO-d_6) spectrum of compound **1c**.

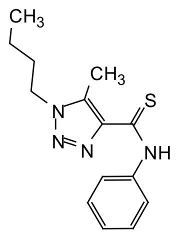

1c

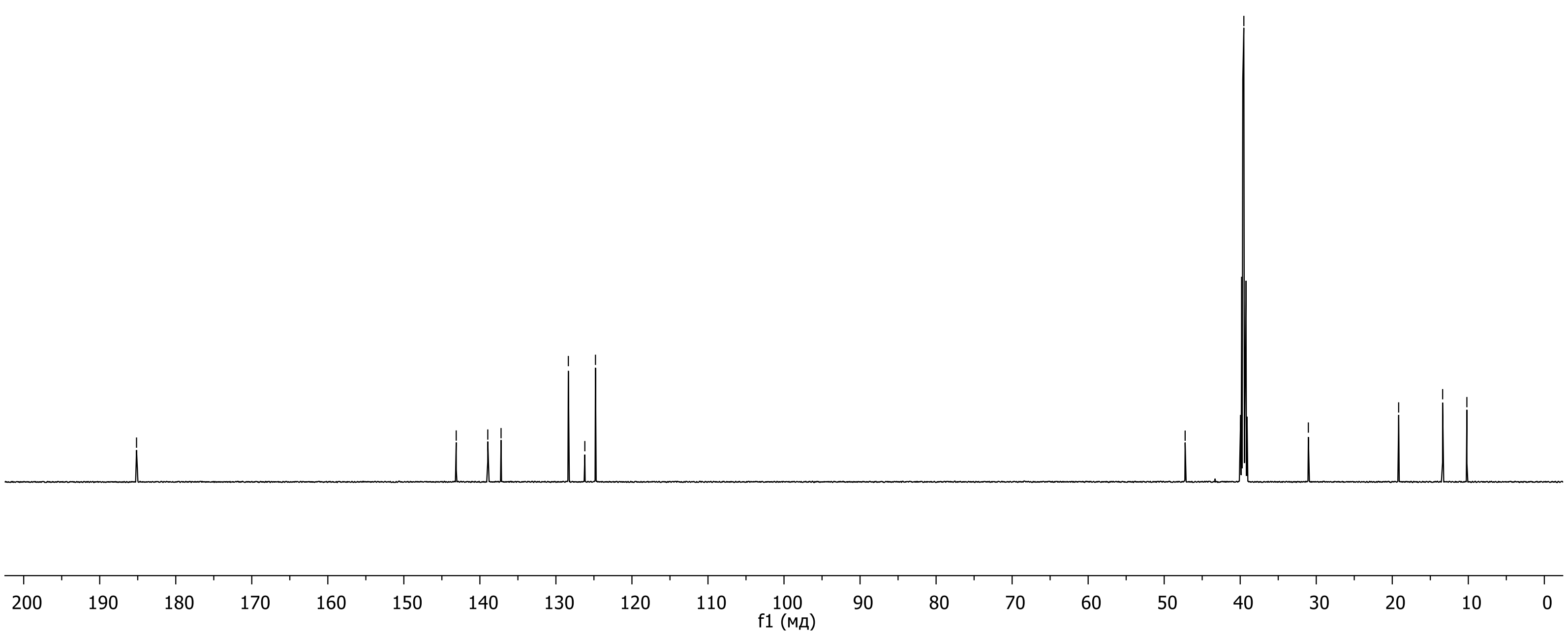

The ^{13}C NMR (100 MHz, DMSO-d_6) spectrum of compound **1c**.

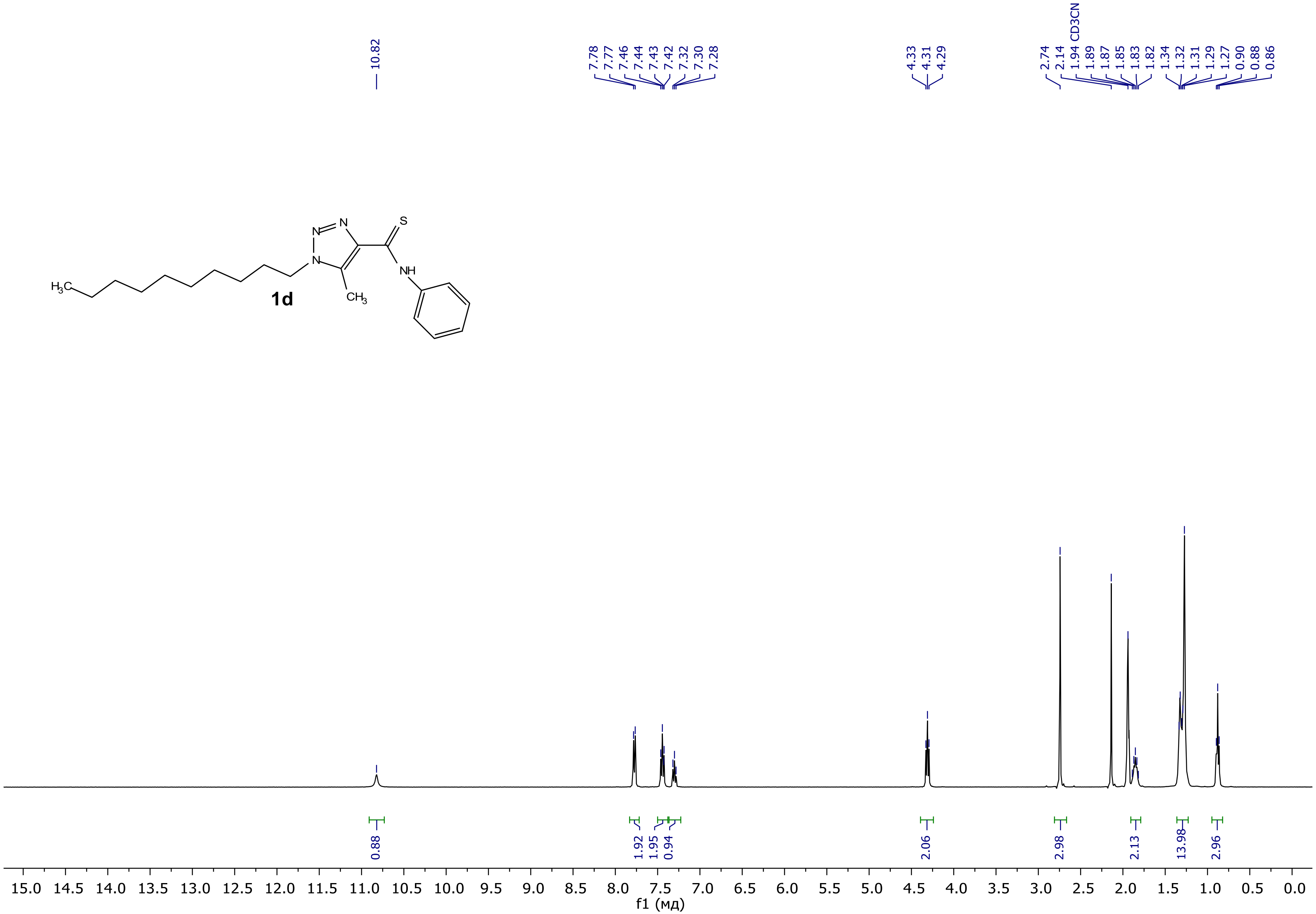

The ^{1}H NMR (400 MHz, CD_3CN) spectrum of compound **1d**. 7

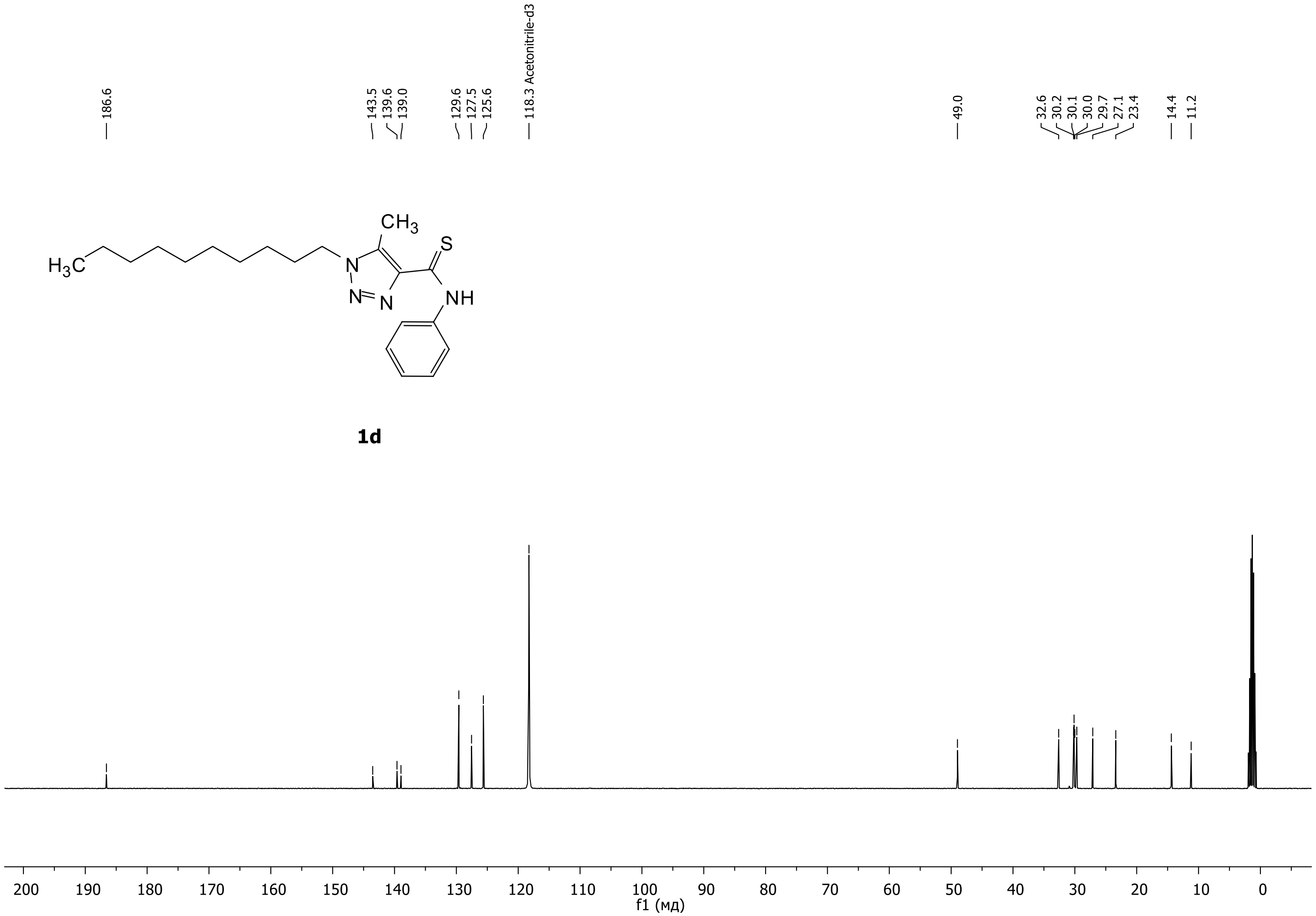

The ^{13}C NMR (100 MHz, CD_3CN) spectrum of compound **1d**.

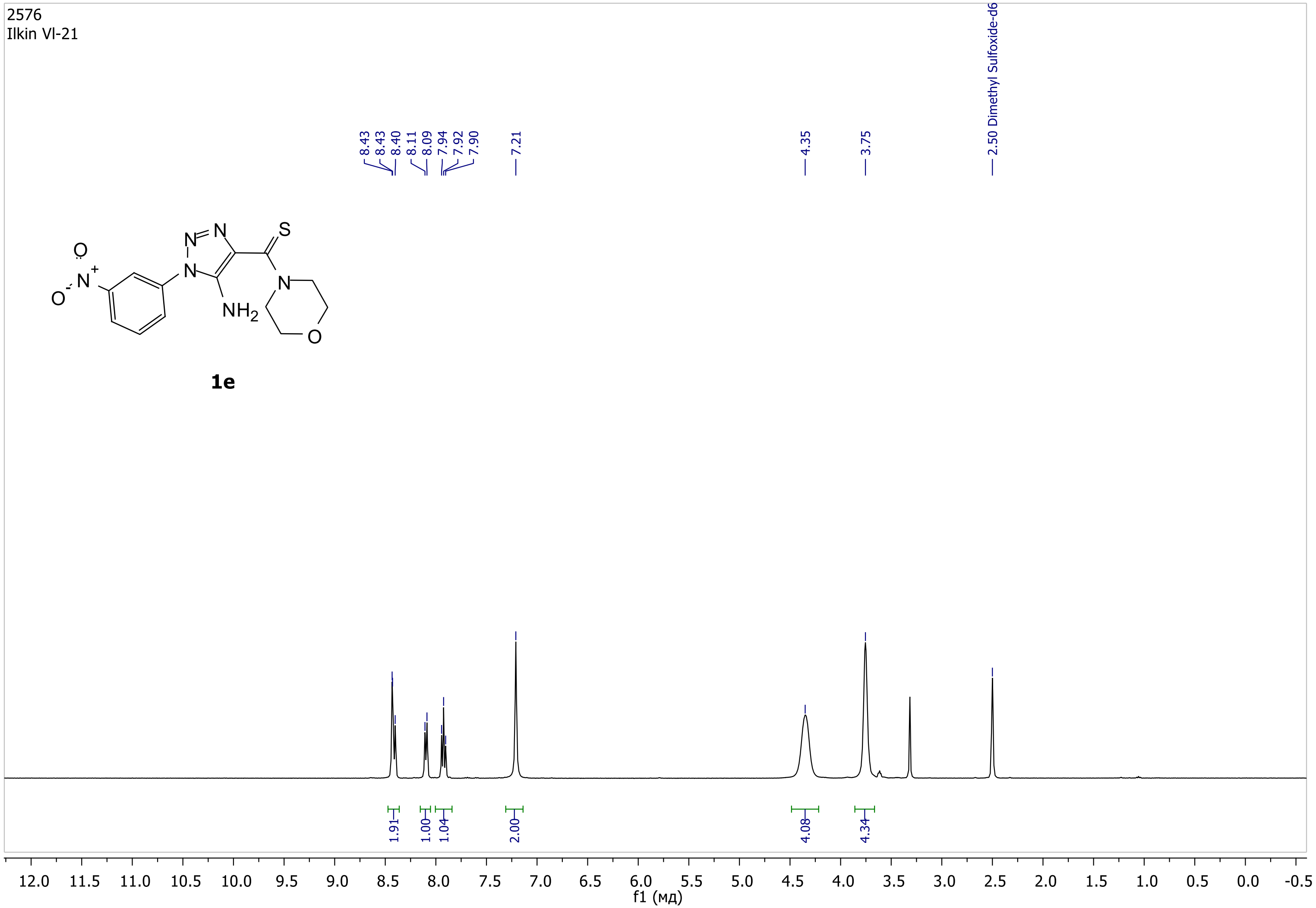

The ^{1}H NMR (400 MHz, DMSO-d_6) spectrum of compound **1e**.

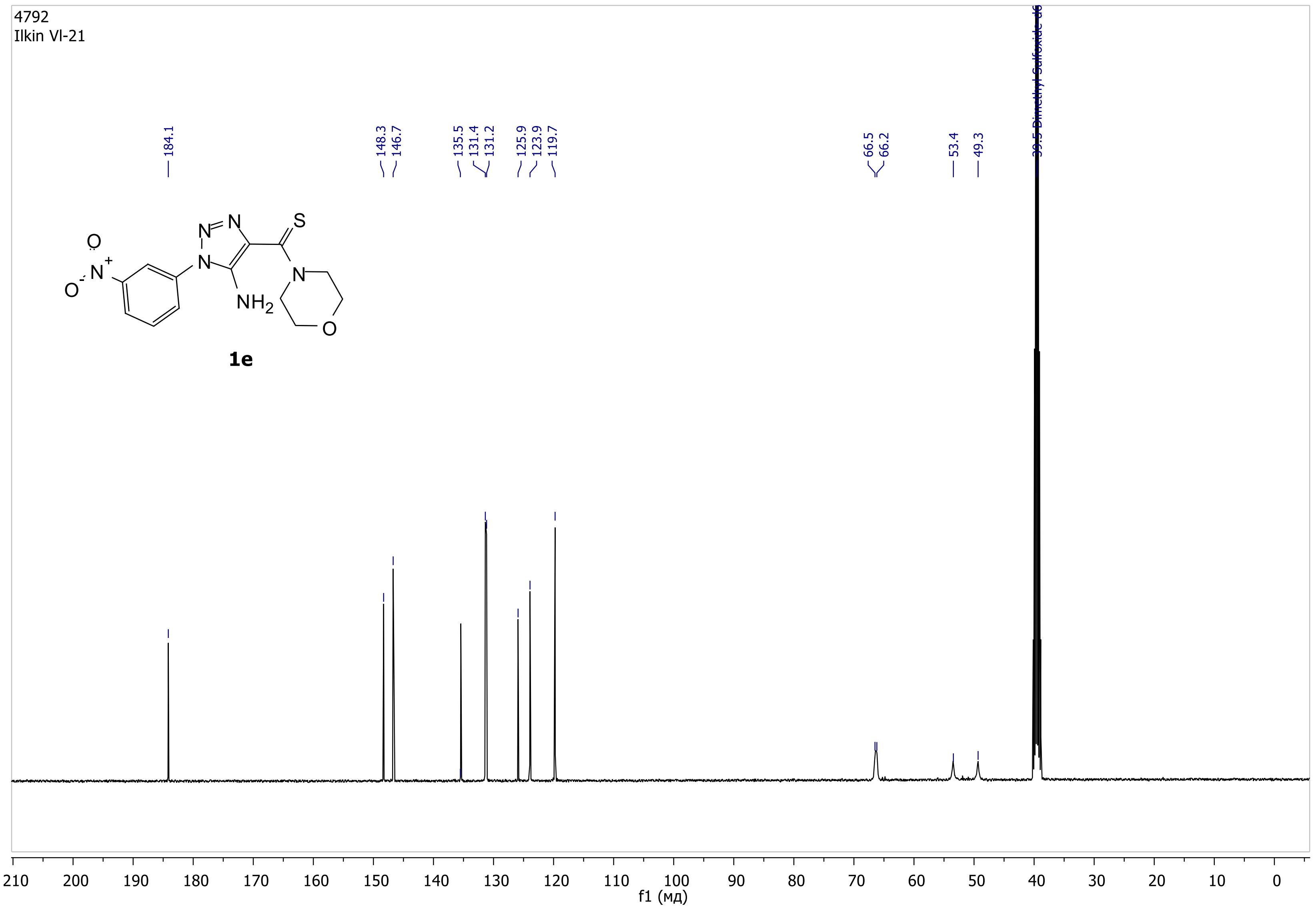

The ^{13}C NMR (100 MHz, DMSO-d_6) spectrum of compound **1e**.

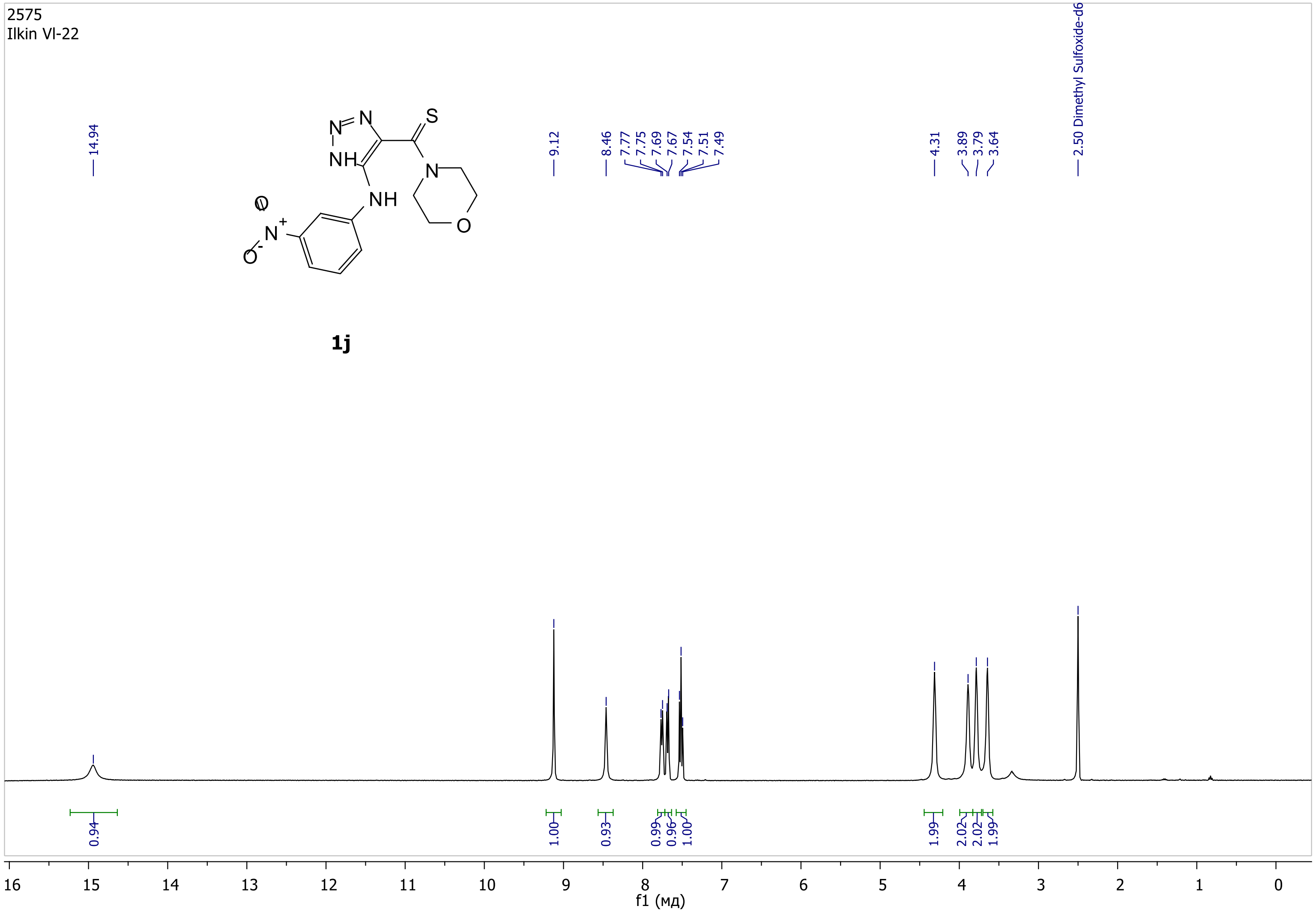

The ^{1}H NMR (400 MHz, DMSO-d_6) spectrum of compound **1j**.

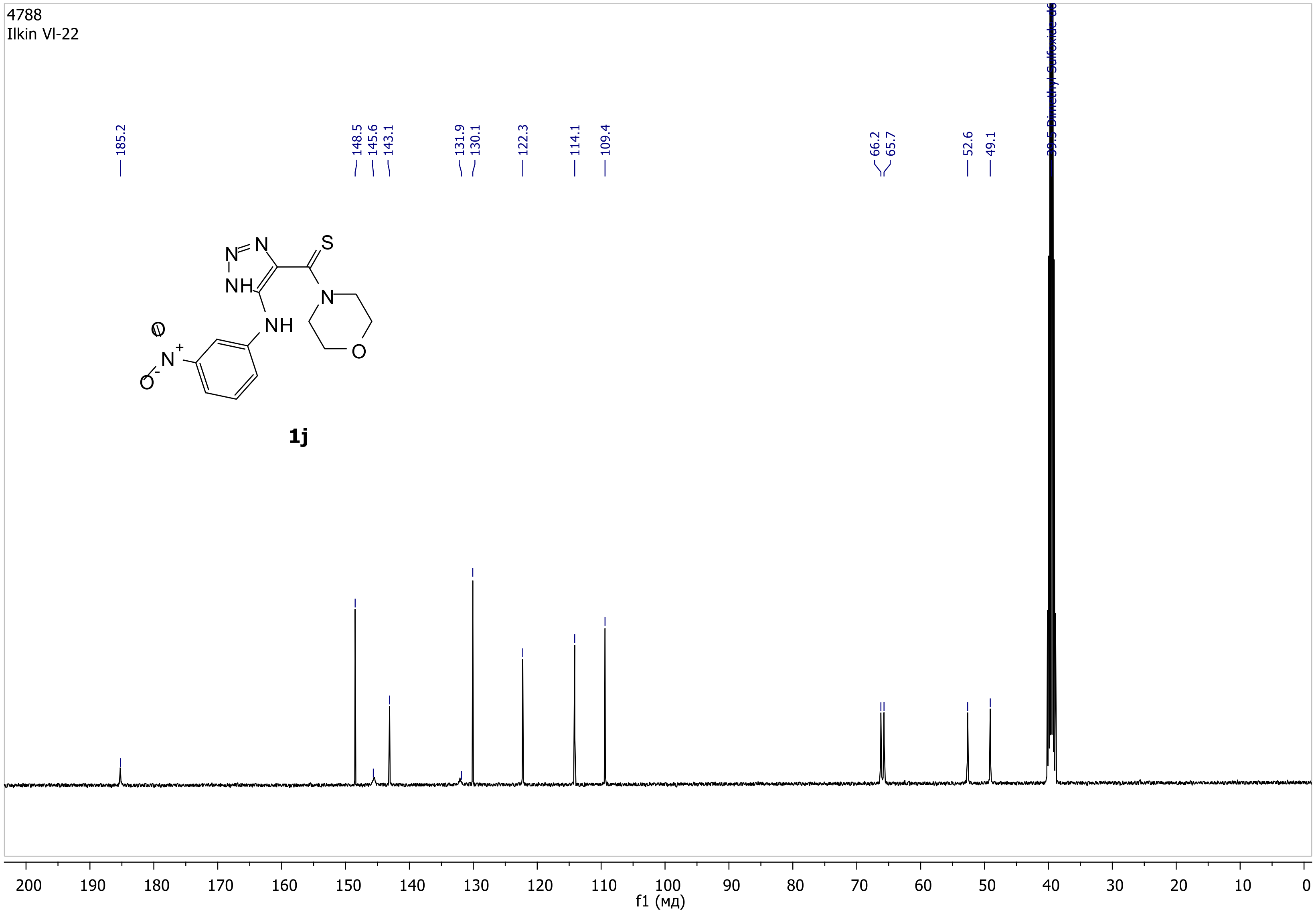

The ^{13}C NMR (100 MHz, DMSO-d_6) spectrum of compound **1j**.

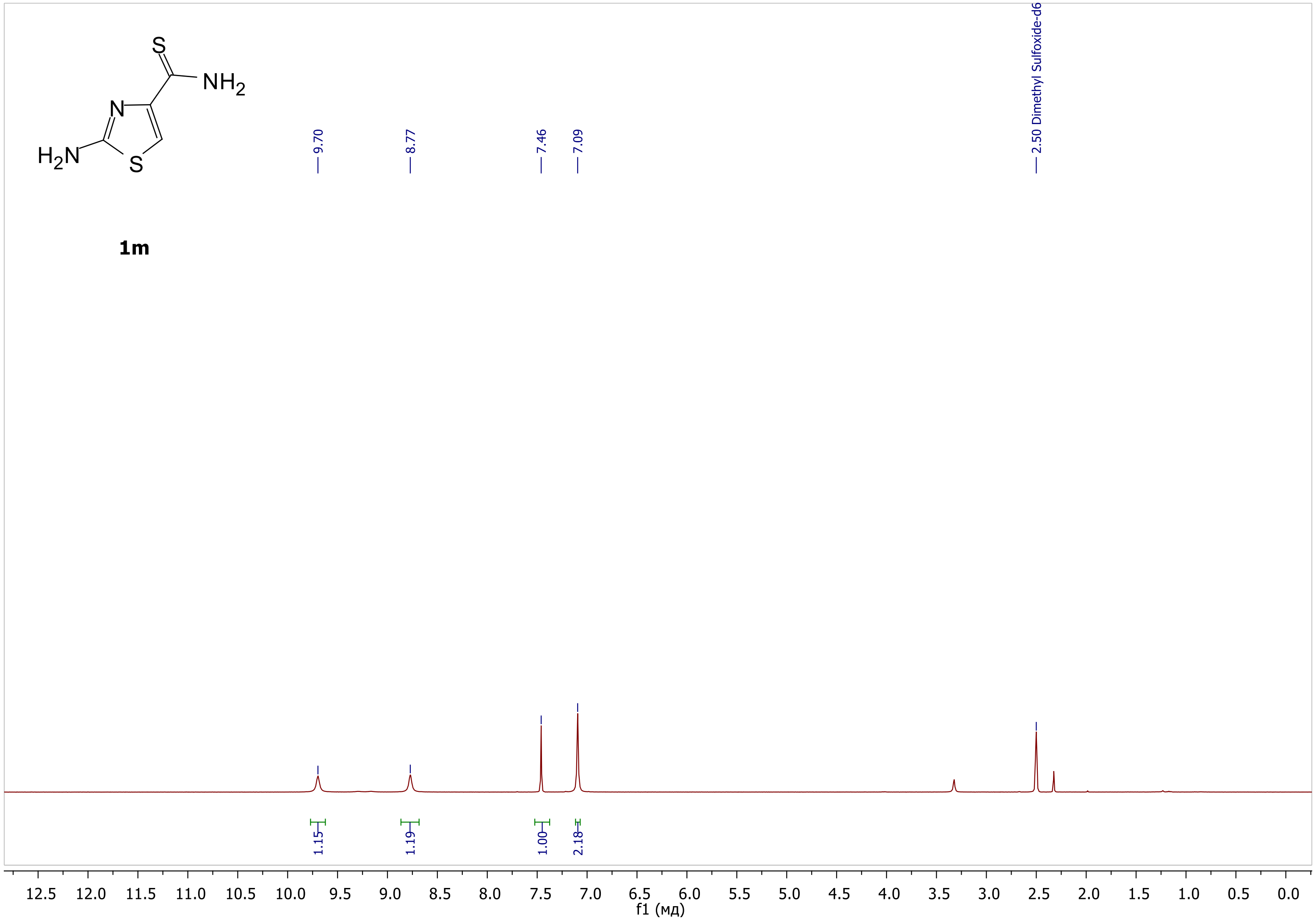

The ^{1}H NMR (400 MHz, DMSO-d_6) spectrum of compound **1m**.

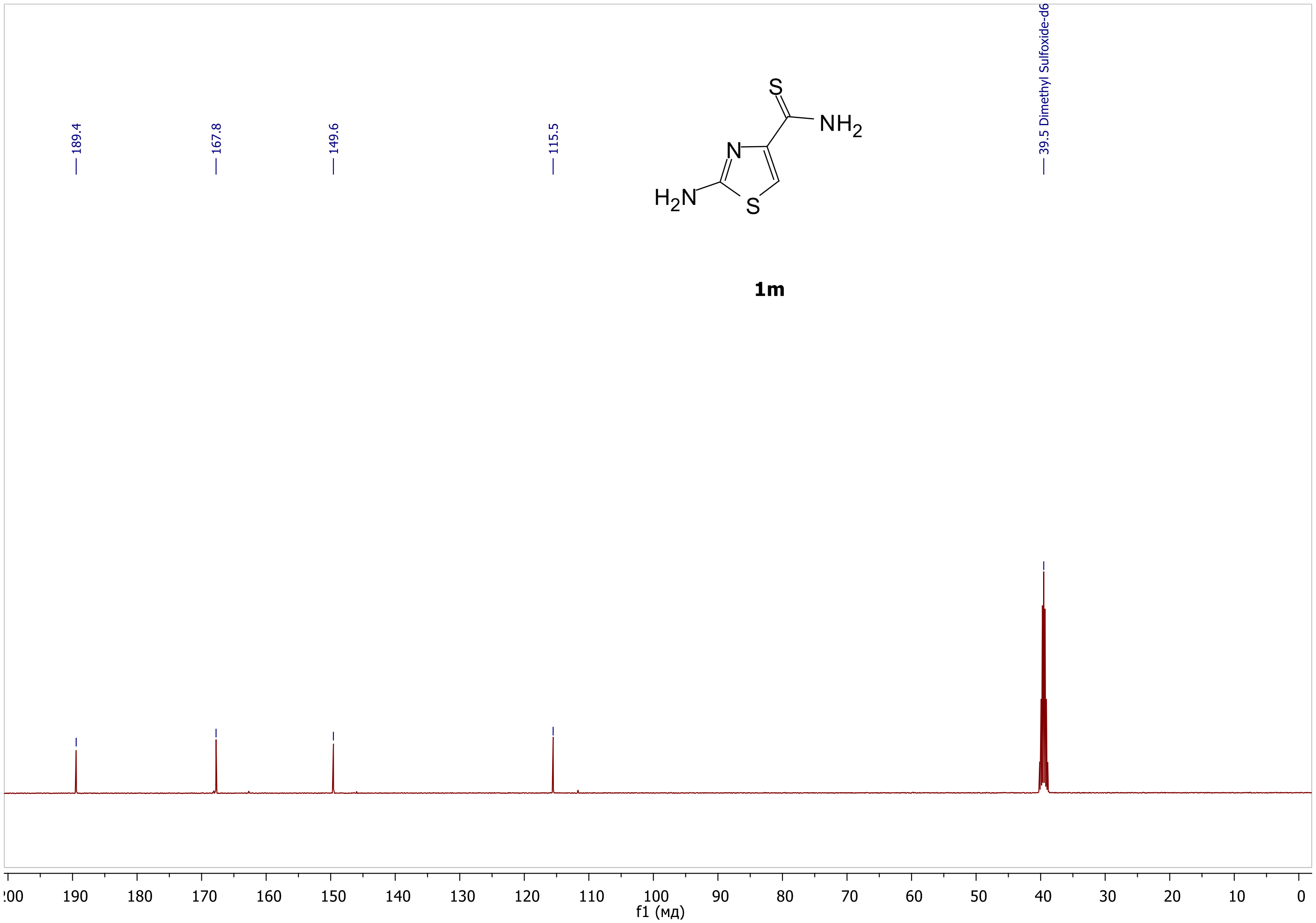

The ^{13}C NMR (100 MHz, DMSO-d_6) spectrum of compound **1m**.

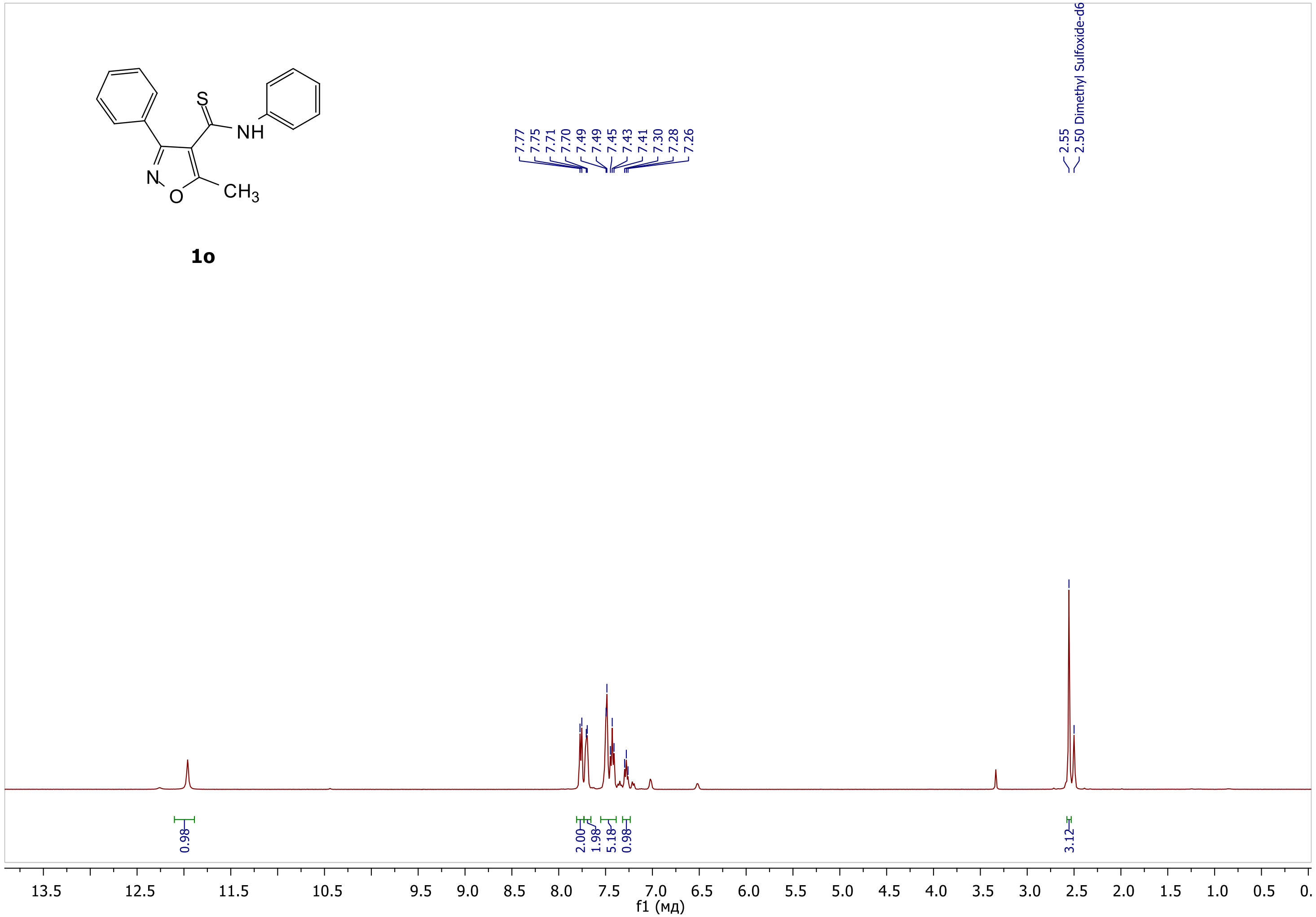

The ^{1}H NMR (400 MHz, DMSO-d_6) spectrum of compound **1o**.

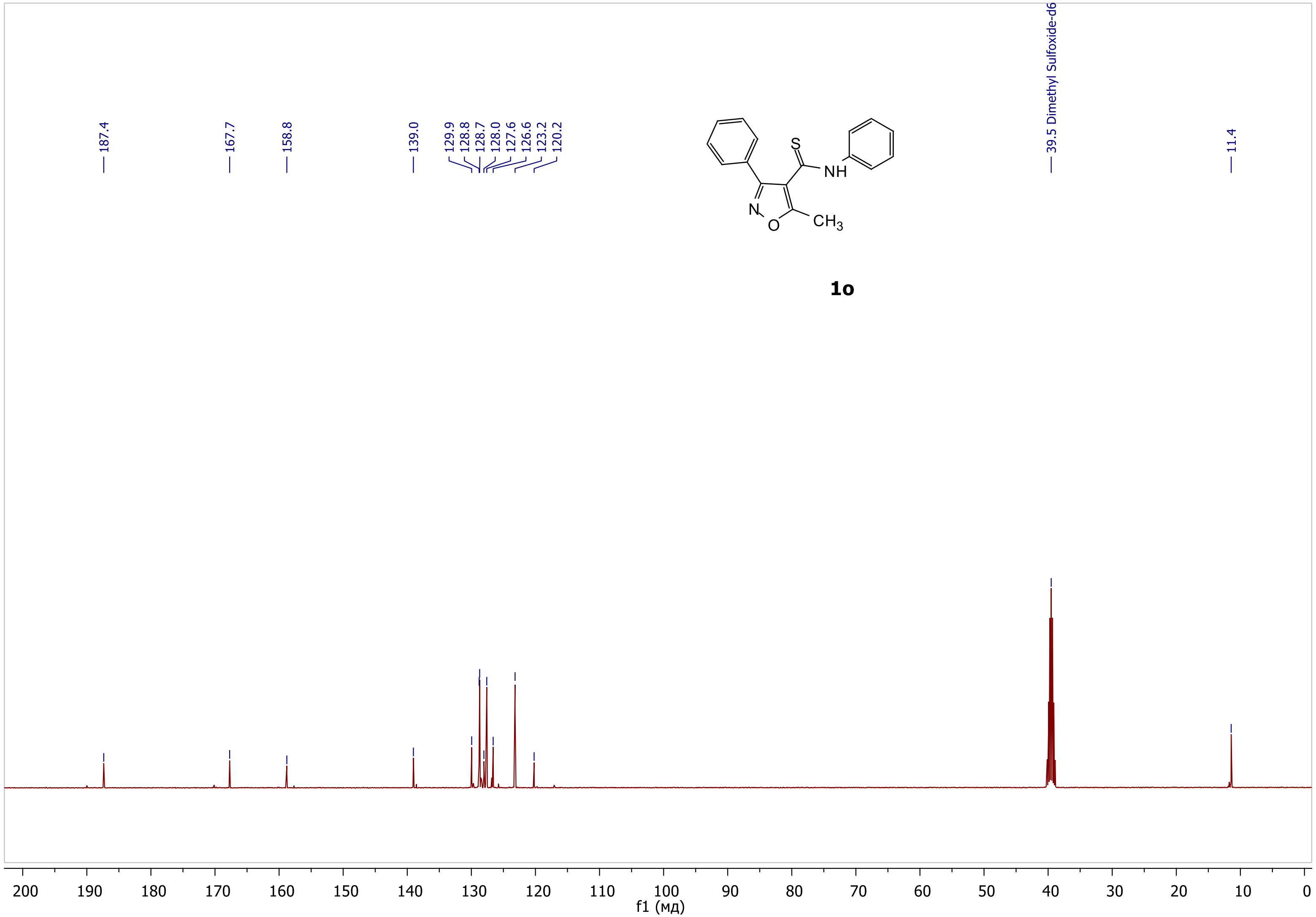

The ^{13}C NMR (100 MHz, DMSO-d_6) spectrum of compound **1o** .

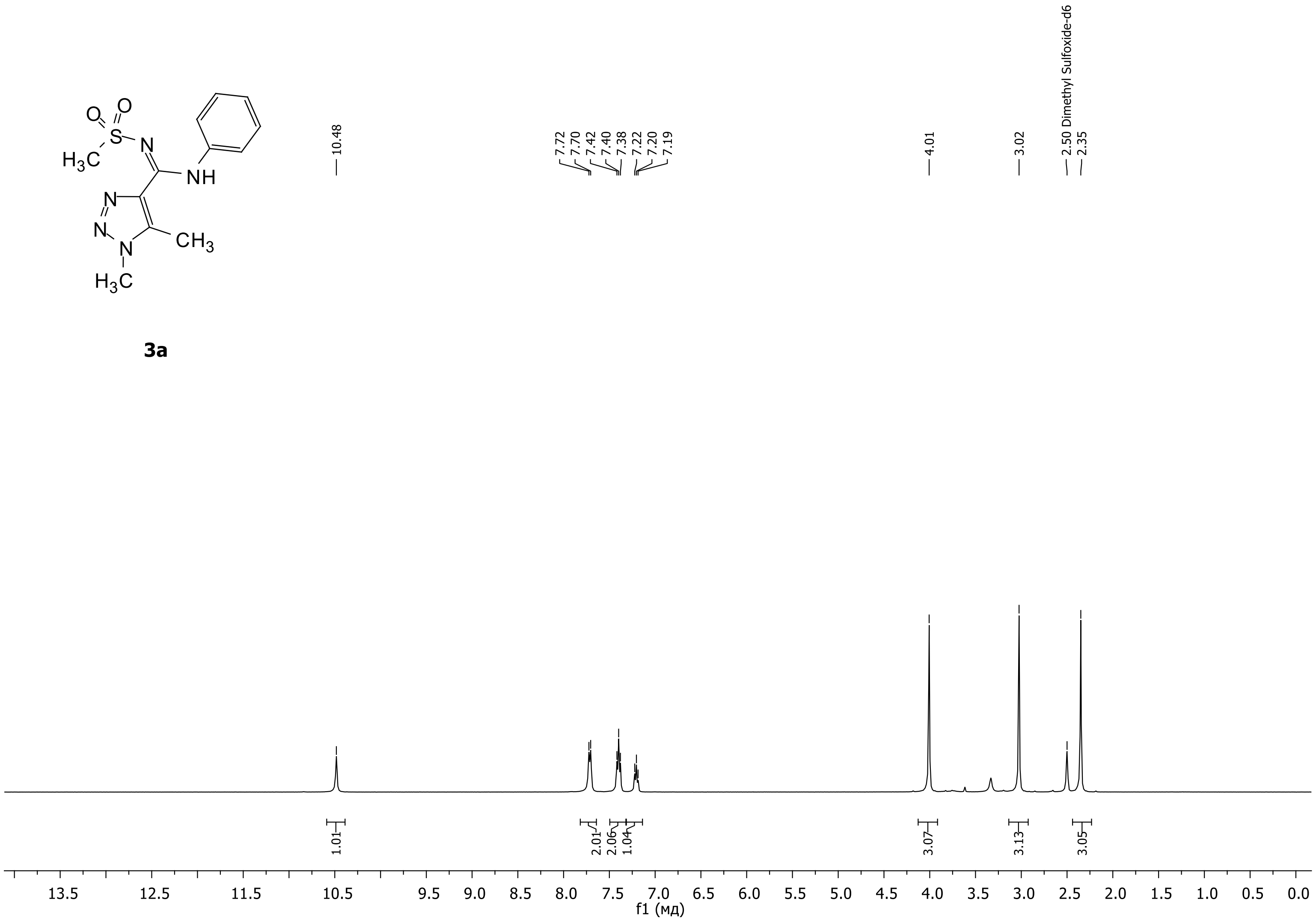

The ^{1}H NMR (400 MHz, DMSO-d_6) spectrum of compound **3a**.

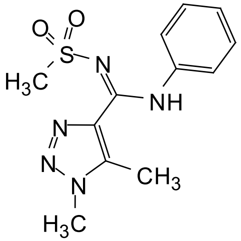

3a

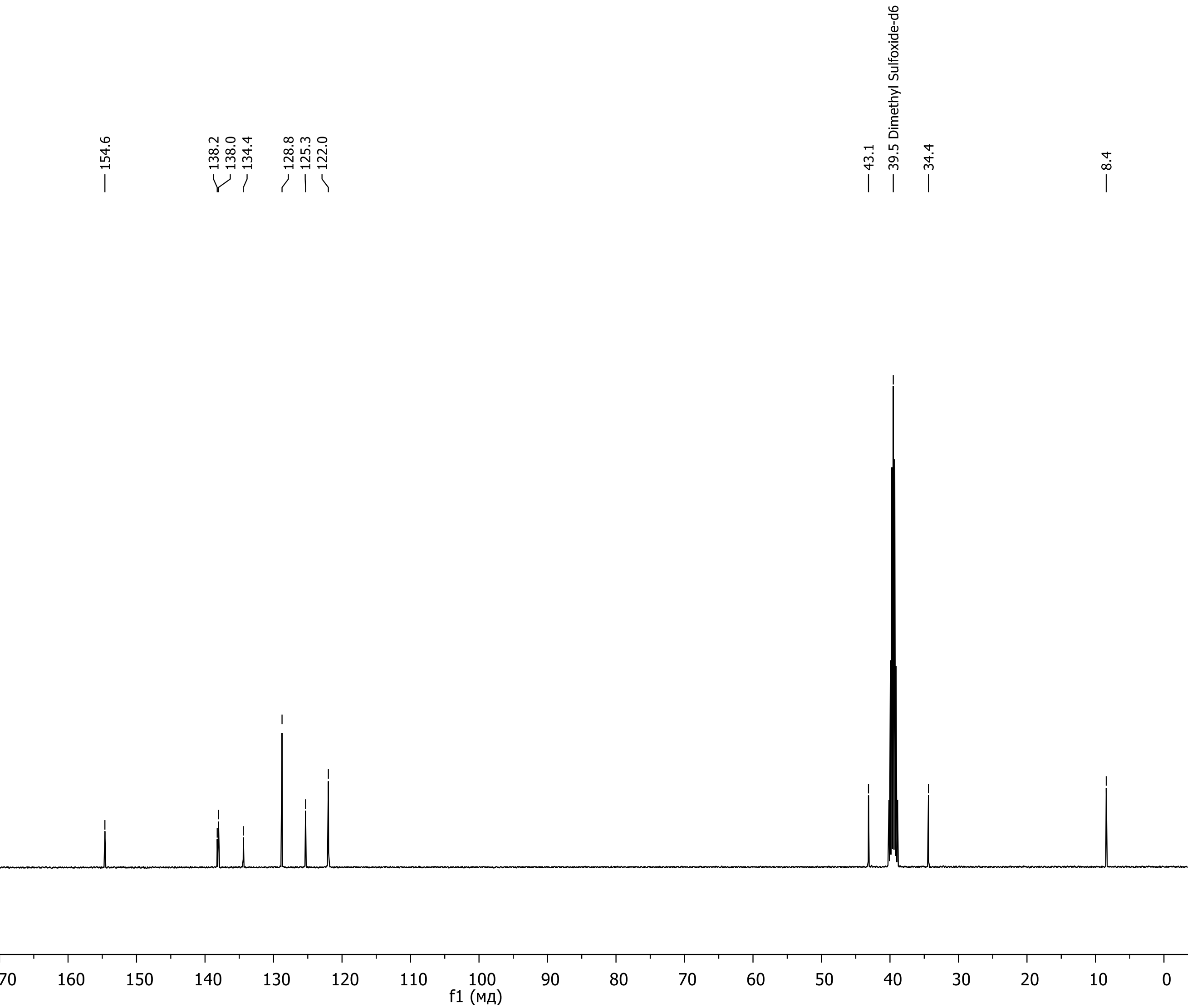

The ^{13}C NMR (100 MHz, DMSO-d_6) spectrum of compound **3a**.

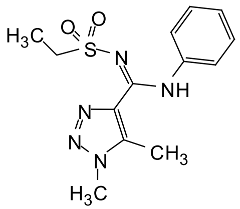

3b

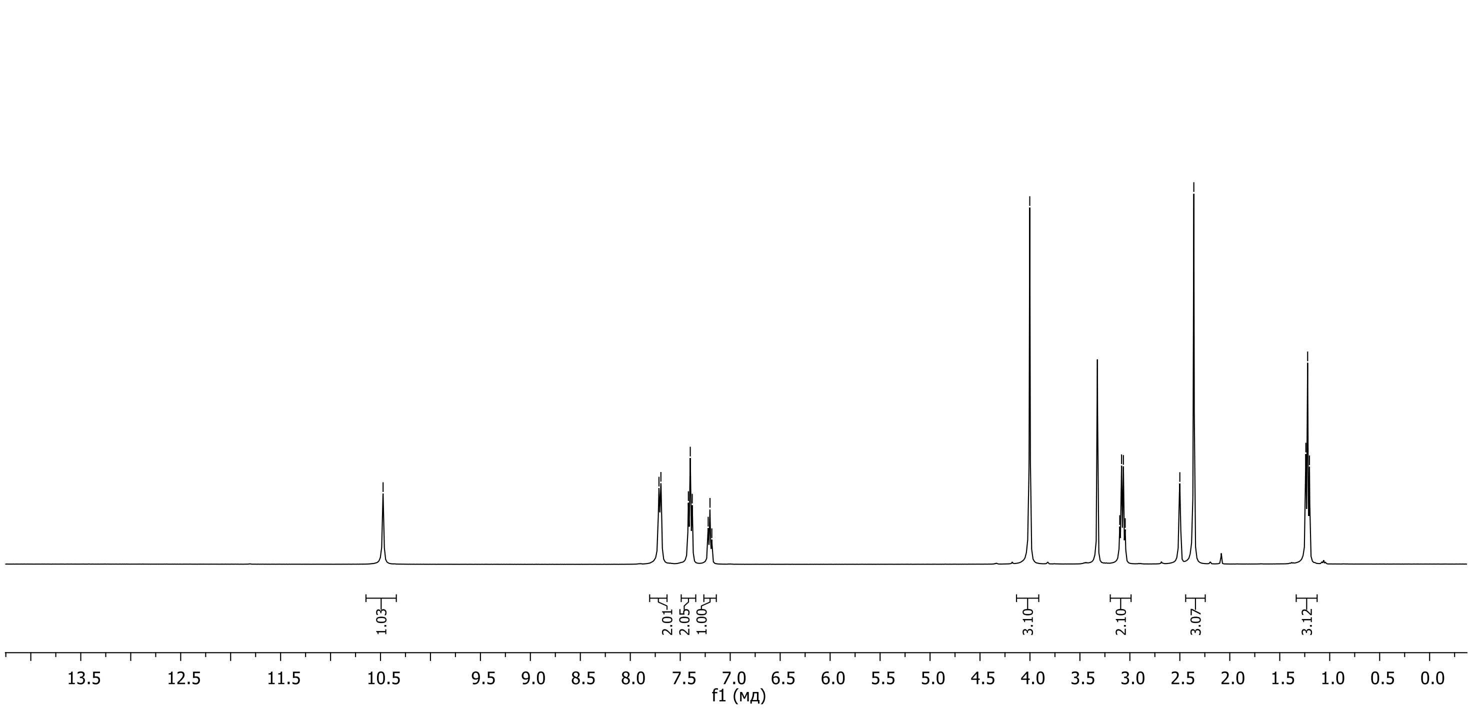

The ^{1}H NMR (400 MHz, DMSO-d_6) spectrum of compound **3b**.

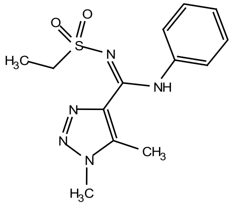

3b

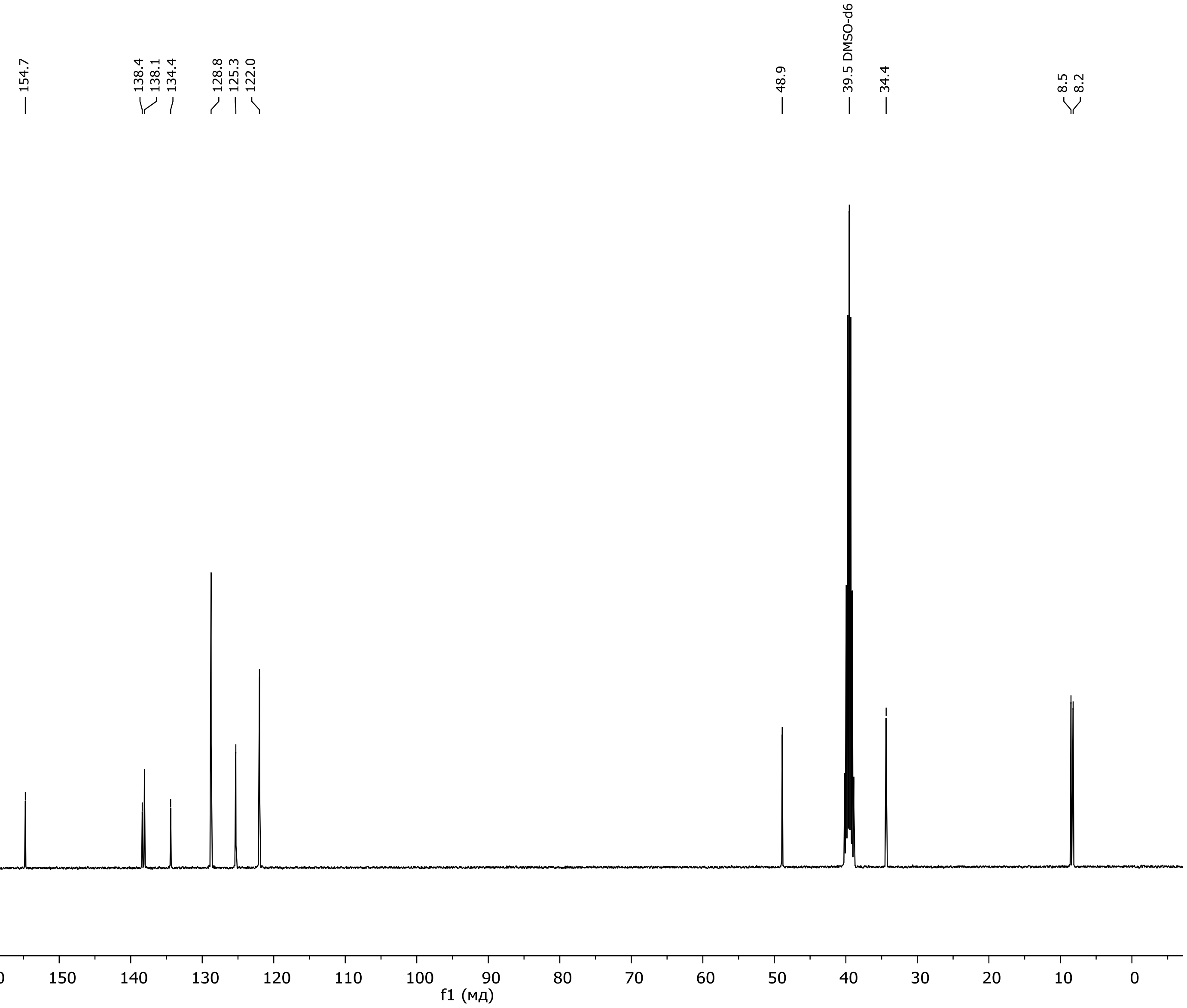

The ^{13}C NMR (100 MHz, DMSO-d_6) spectrum of compound **3b**.

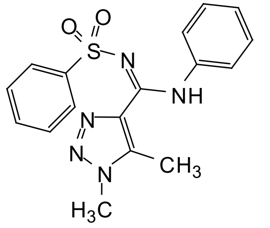

3c

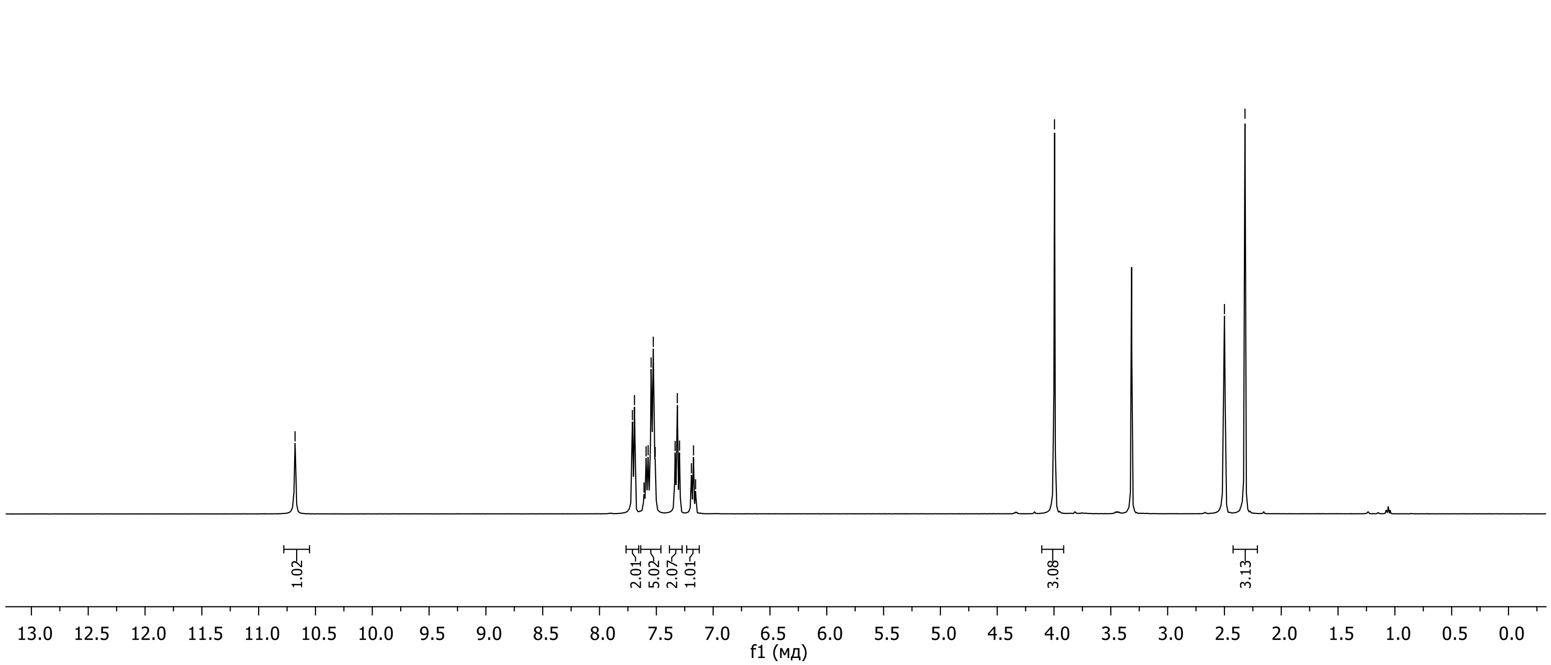

The ^{1}H NMR (400 MHz, DMSO-d_6) spectrum of compound **3c**.

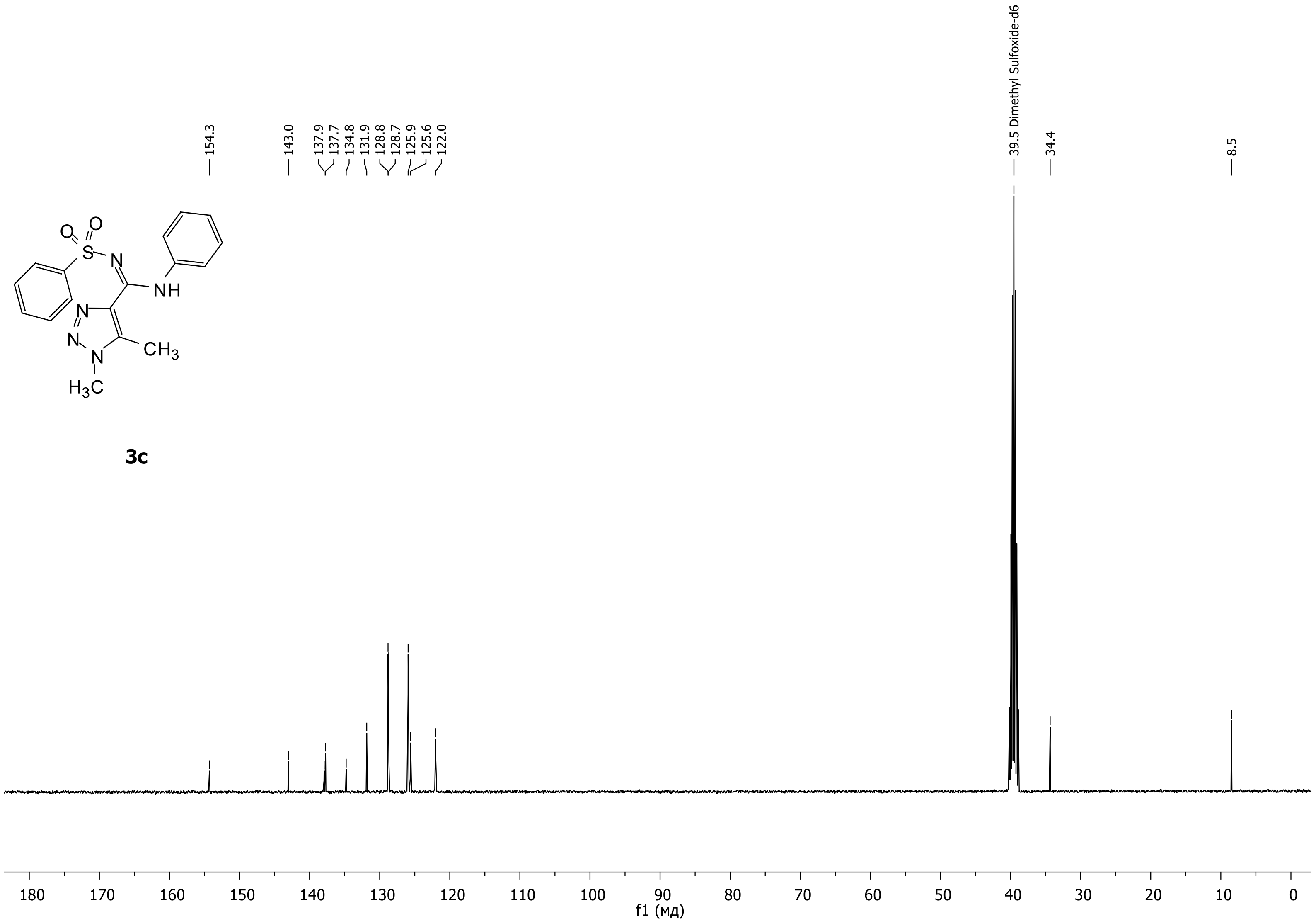

The ^{13}C NMR (100 MHz, DMSO-d_6) spectrum of compound **3c**.

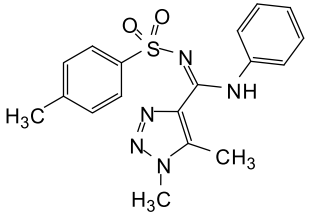

3d

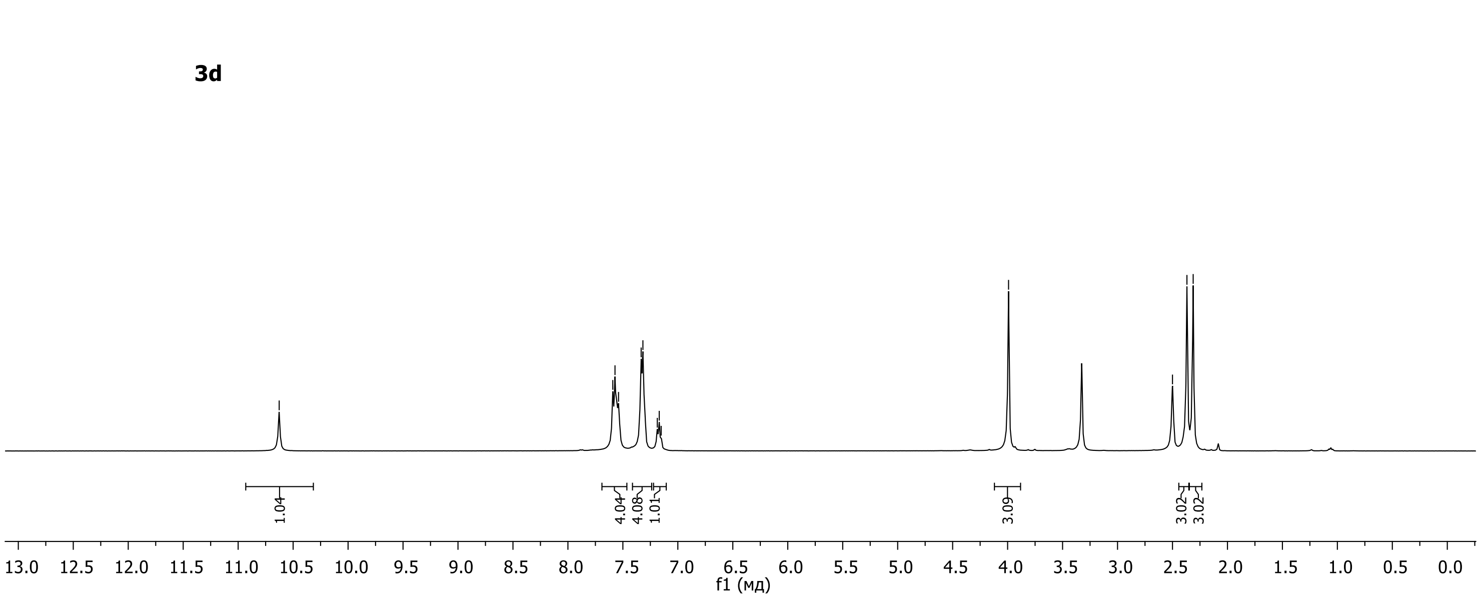

The ^{1}H NMR (400 MHz, DMSO-d_6) spectrum of compound **3d**.

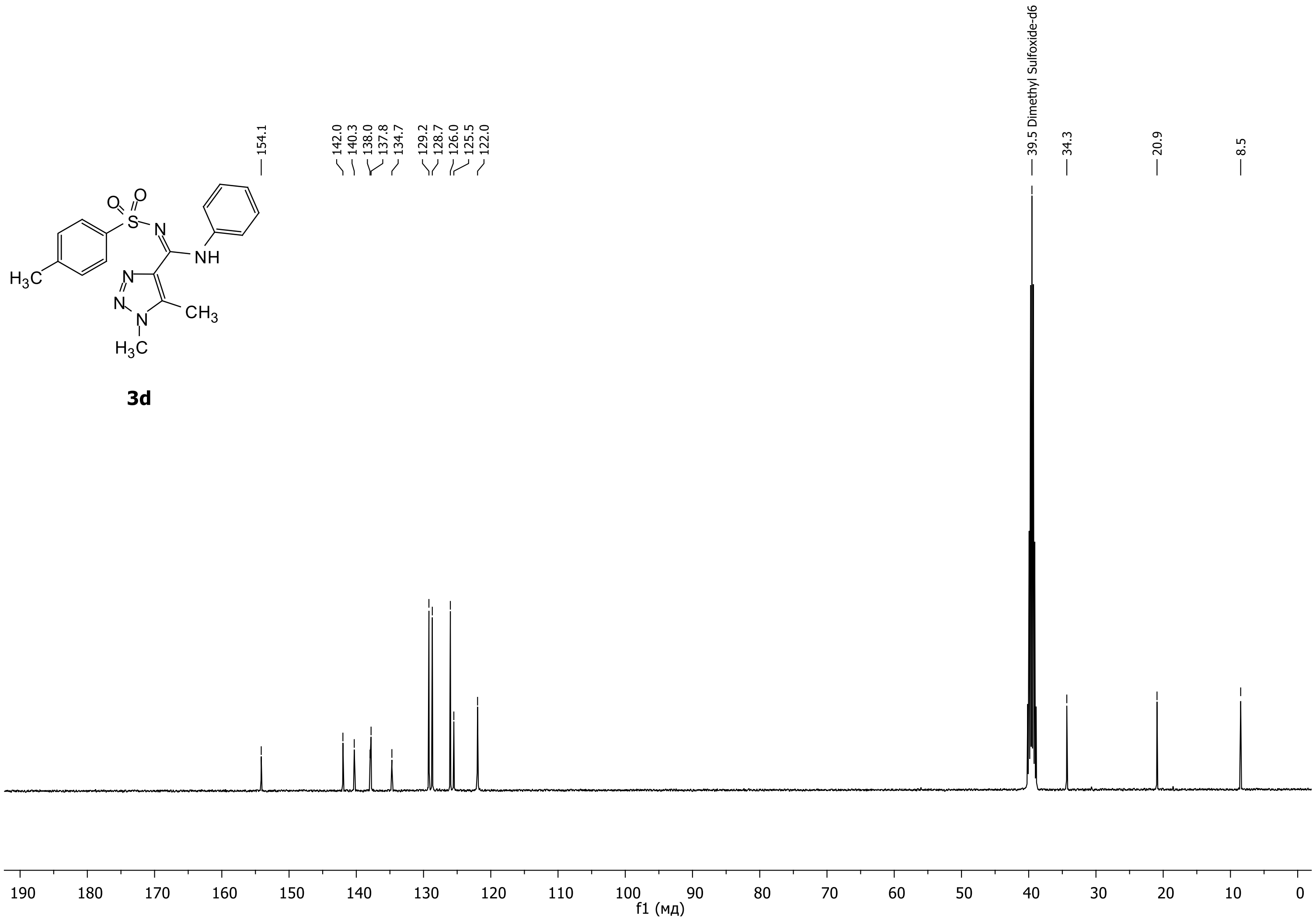

The ^{13}C NMR (100 MHz, DMSO-d_6) spectrum of compound **3d**.

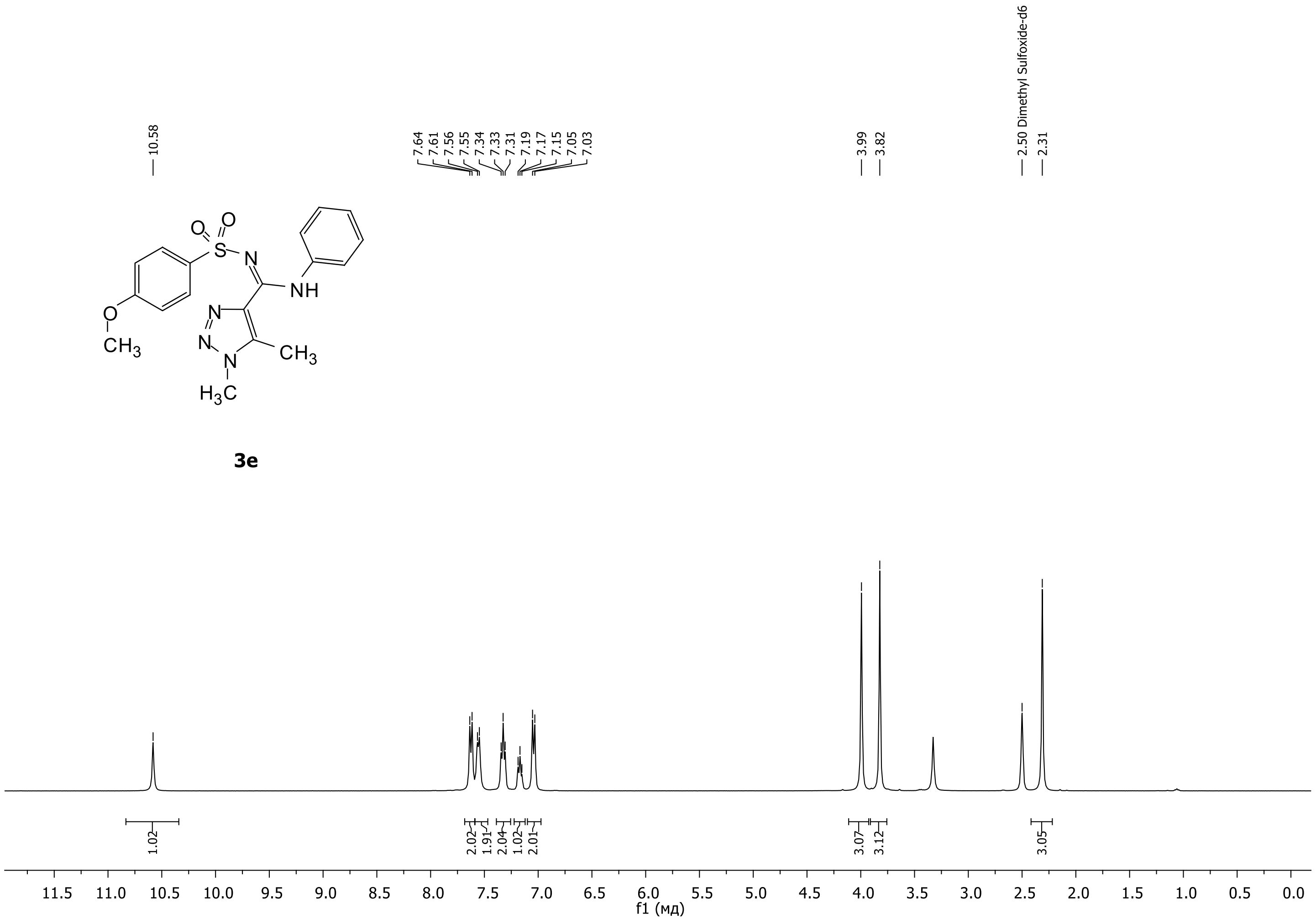

The ^{1}H NMR (400 MHz, DMSO-d_6) spectrum of compound **3e**.

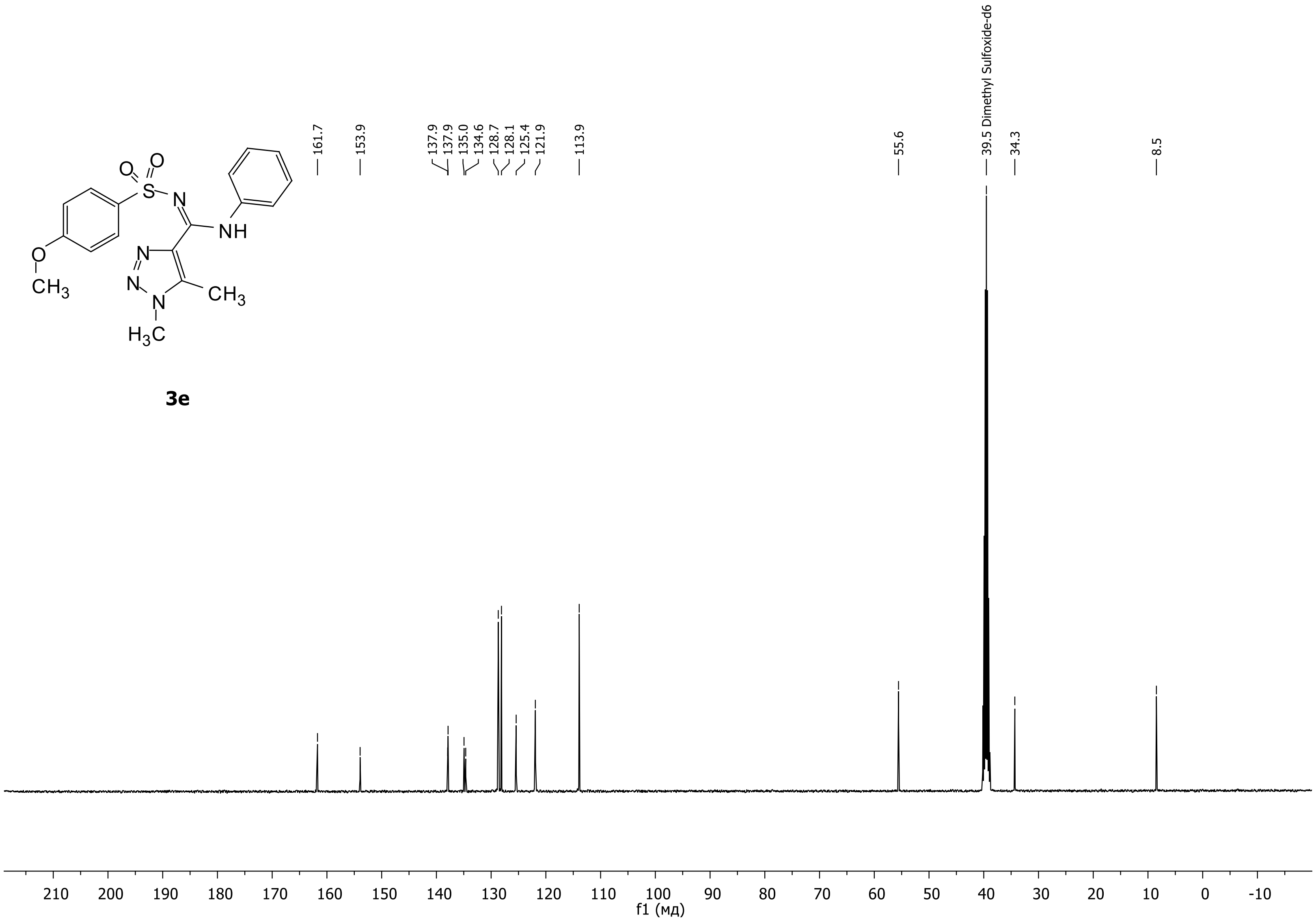

The ^{13}C NMR (100 MHz, DMSO-d_6) spectrum of compound **3e**.

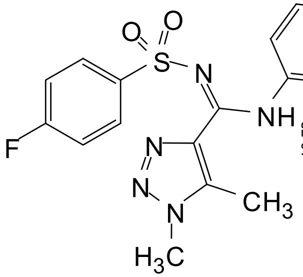

3f

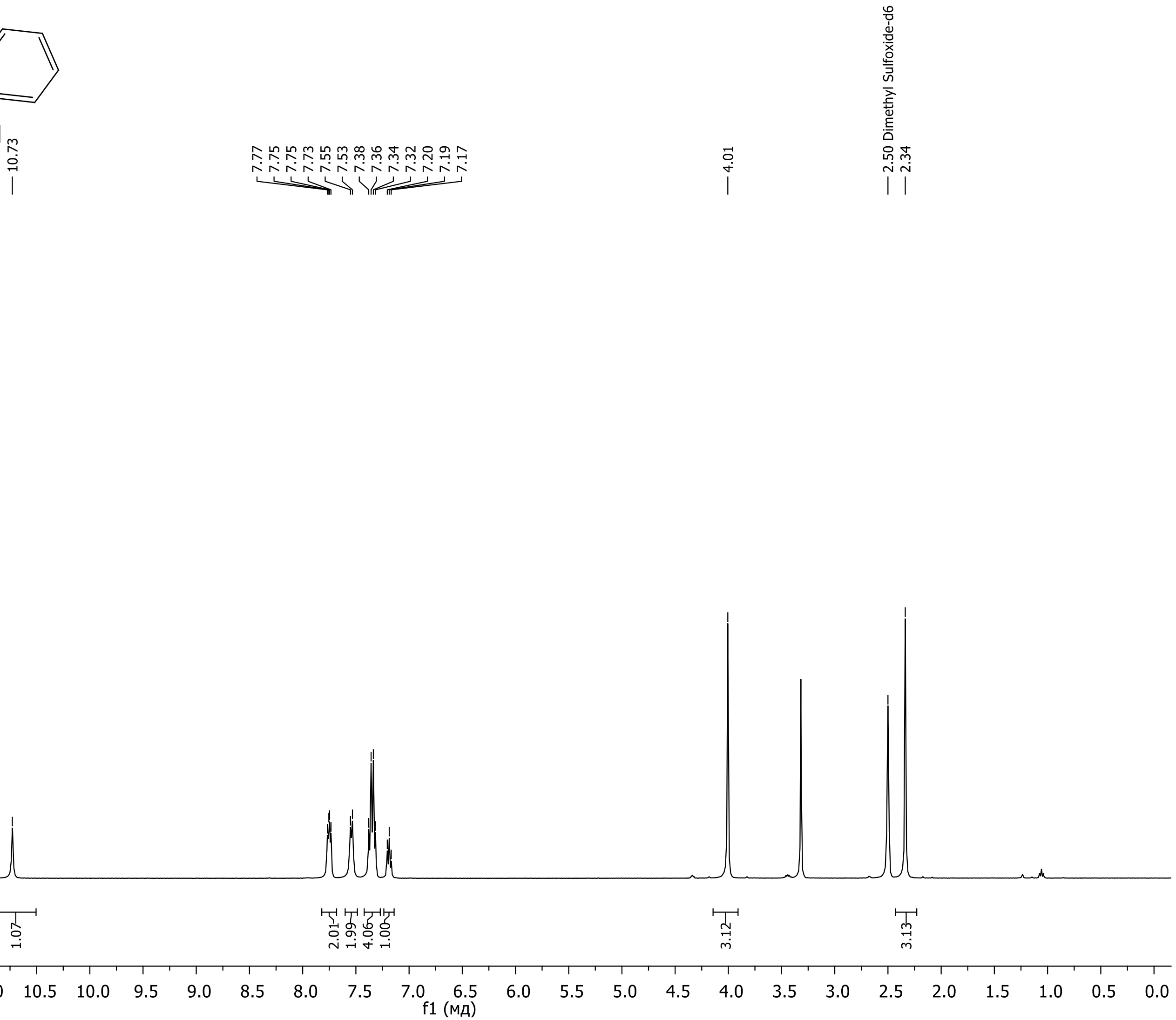

The ^{1}H NMR (400 MHz, DMSO-d_6) spectrum of compound **3f**.

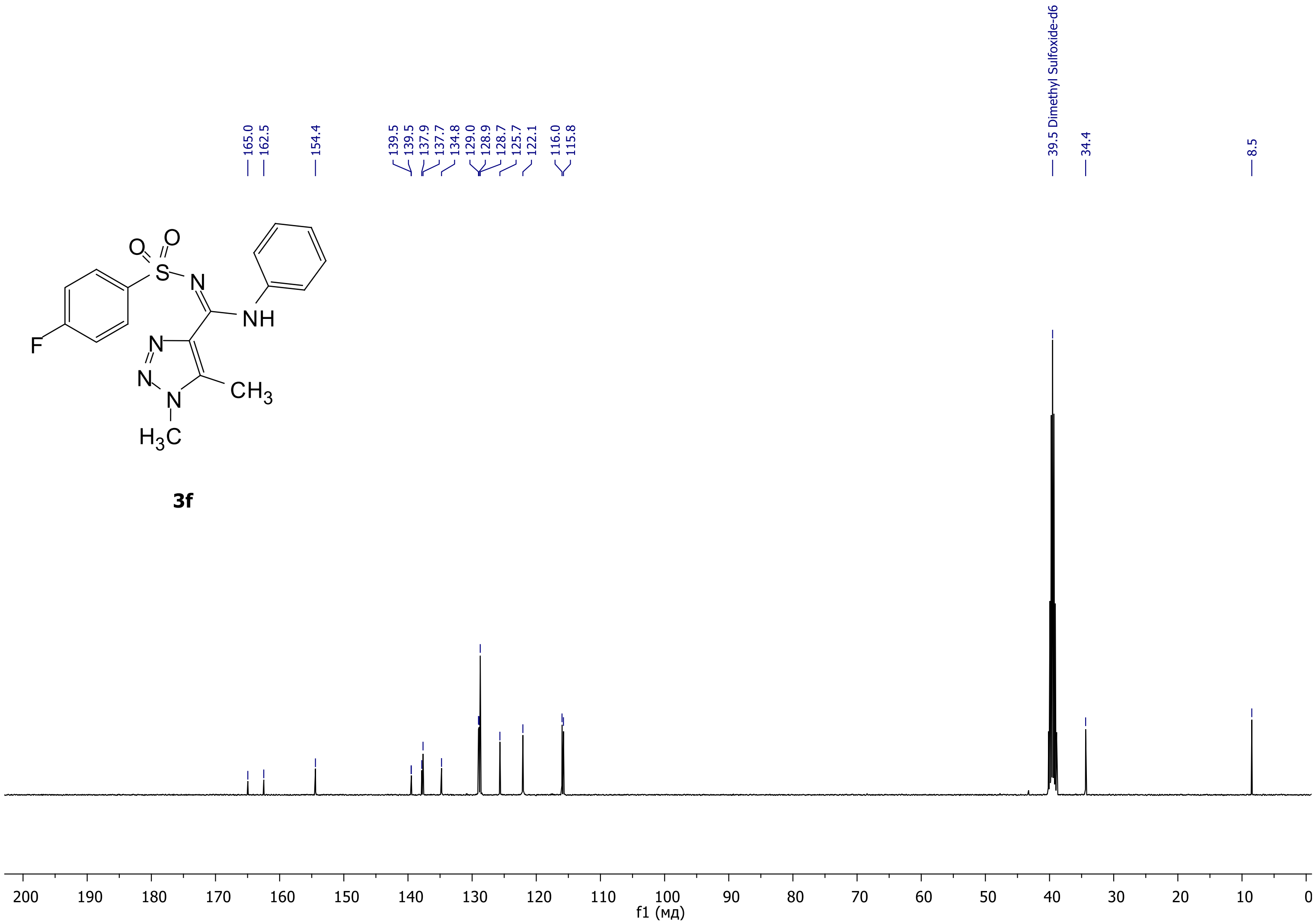

The ^{13}C NMR (100 MHz, DMSO-d_6) spectrum of compound **3f**.

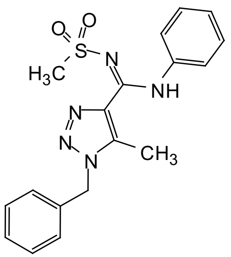

3g

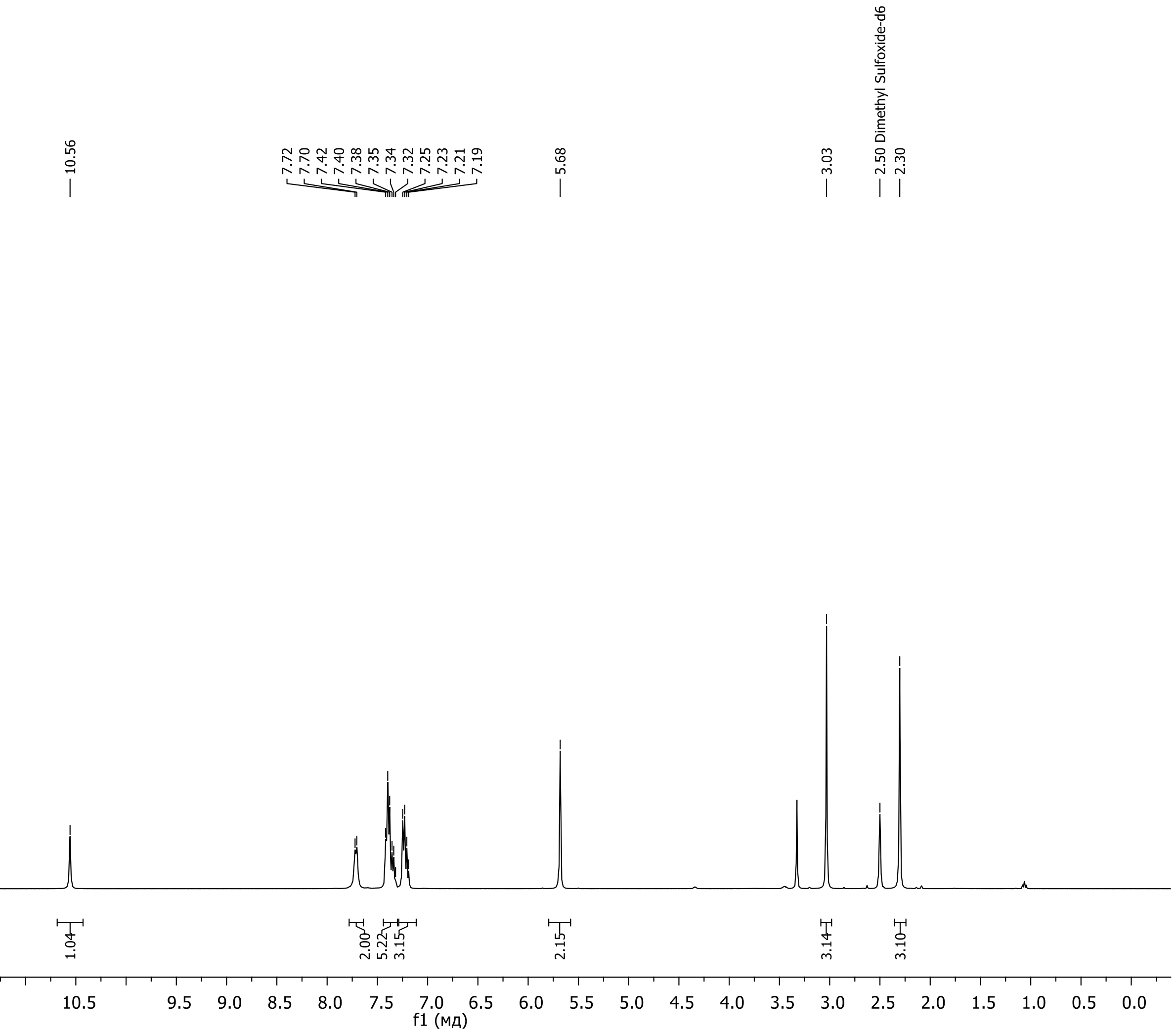

The ^{1}H NMR (400 MHz, DMSO-d_6) spectrum of compound **3g**.

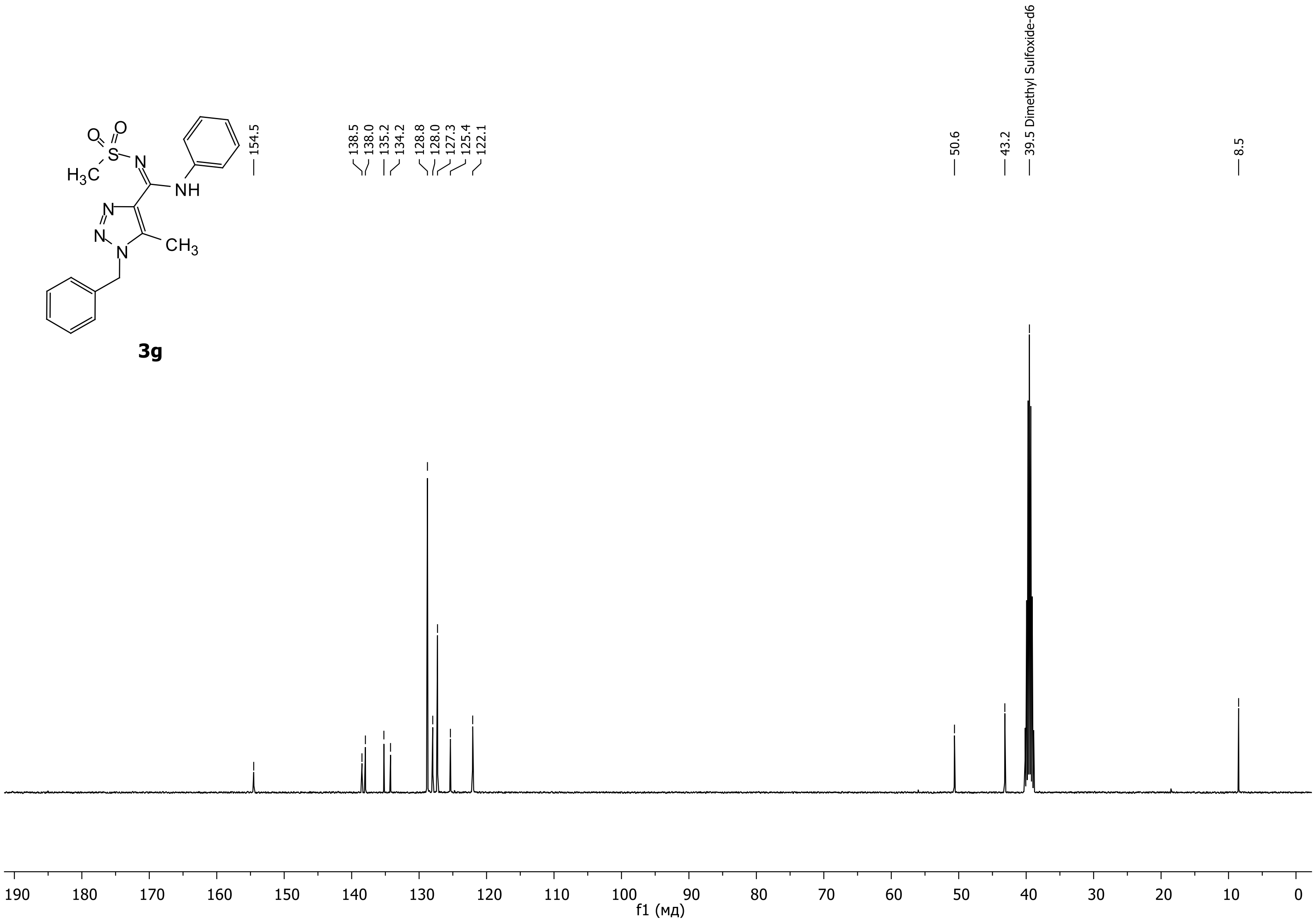

The ^{13}C NMR (100 MHz, DMSO-d_6) spectrum of compound **3g**.

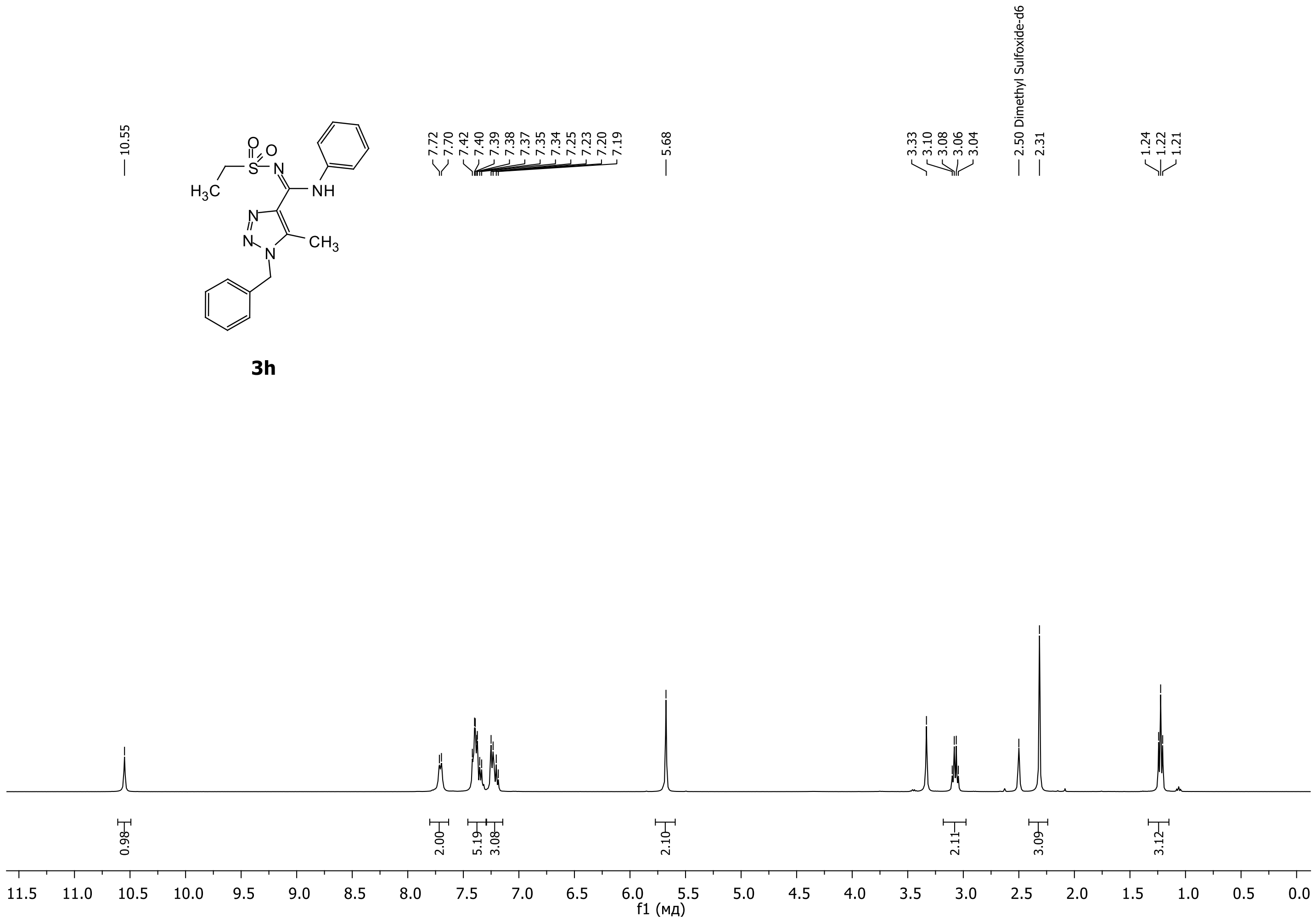

The ^{1}H NMR (400 MHz, DMSO-d_6) spectrum of compound **3h**.

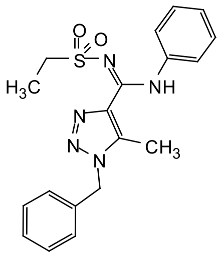

3h

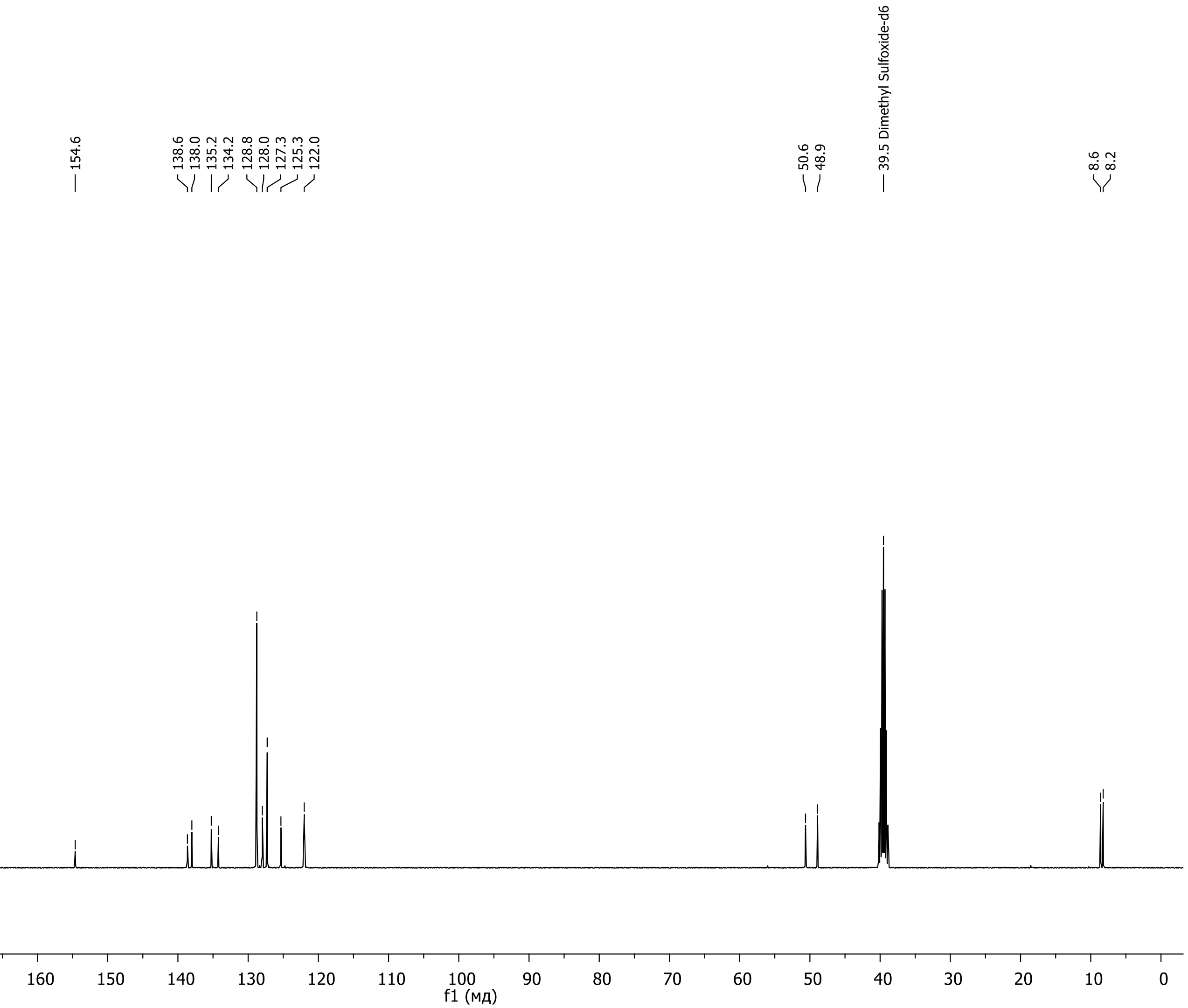

The ^{13}C NMR (100 MHz, DMSO-d_6) spectrum of compound **3h**.

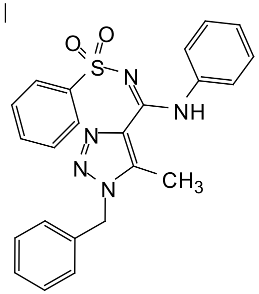

3i

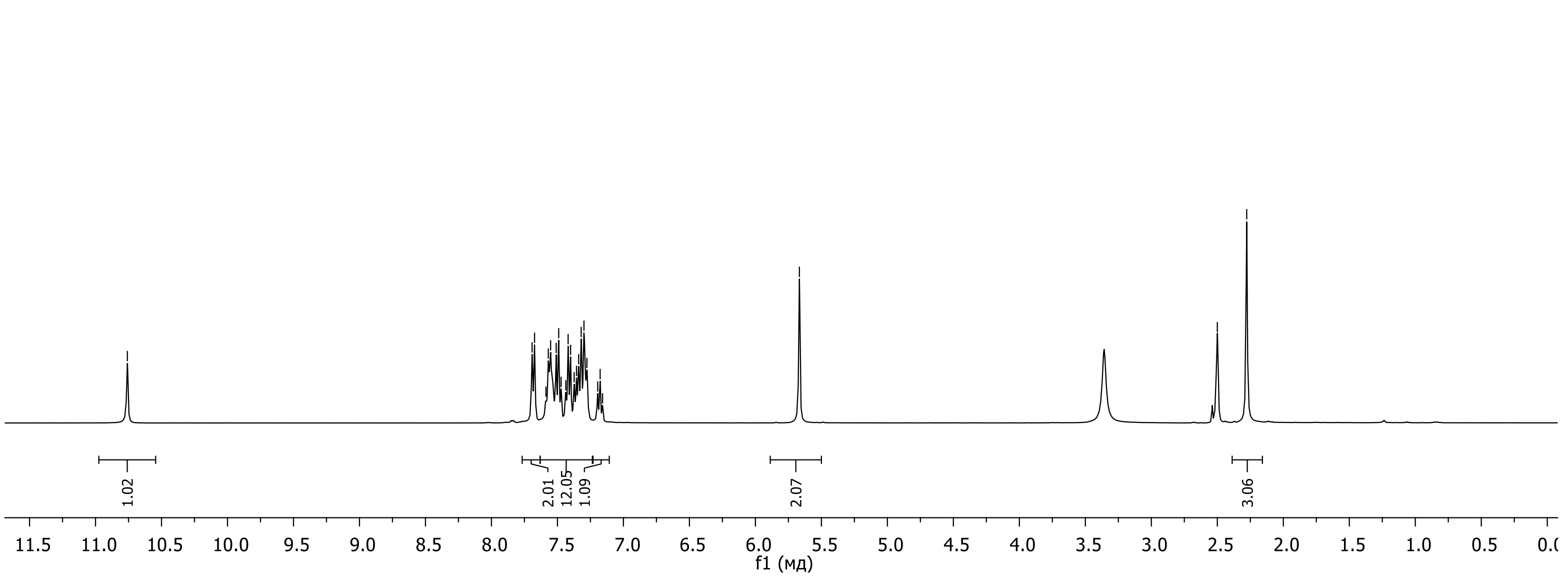

The ^{1}H NMR (400 MHz, DMSO-d_6) spectrum of compound **3i**.

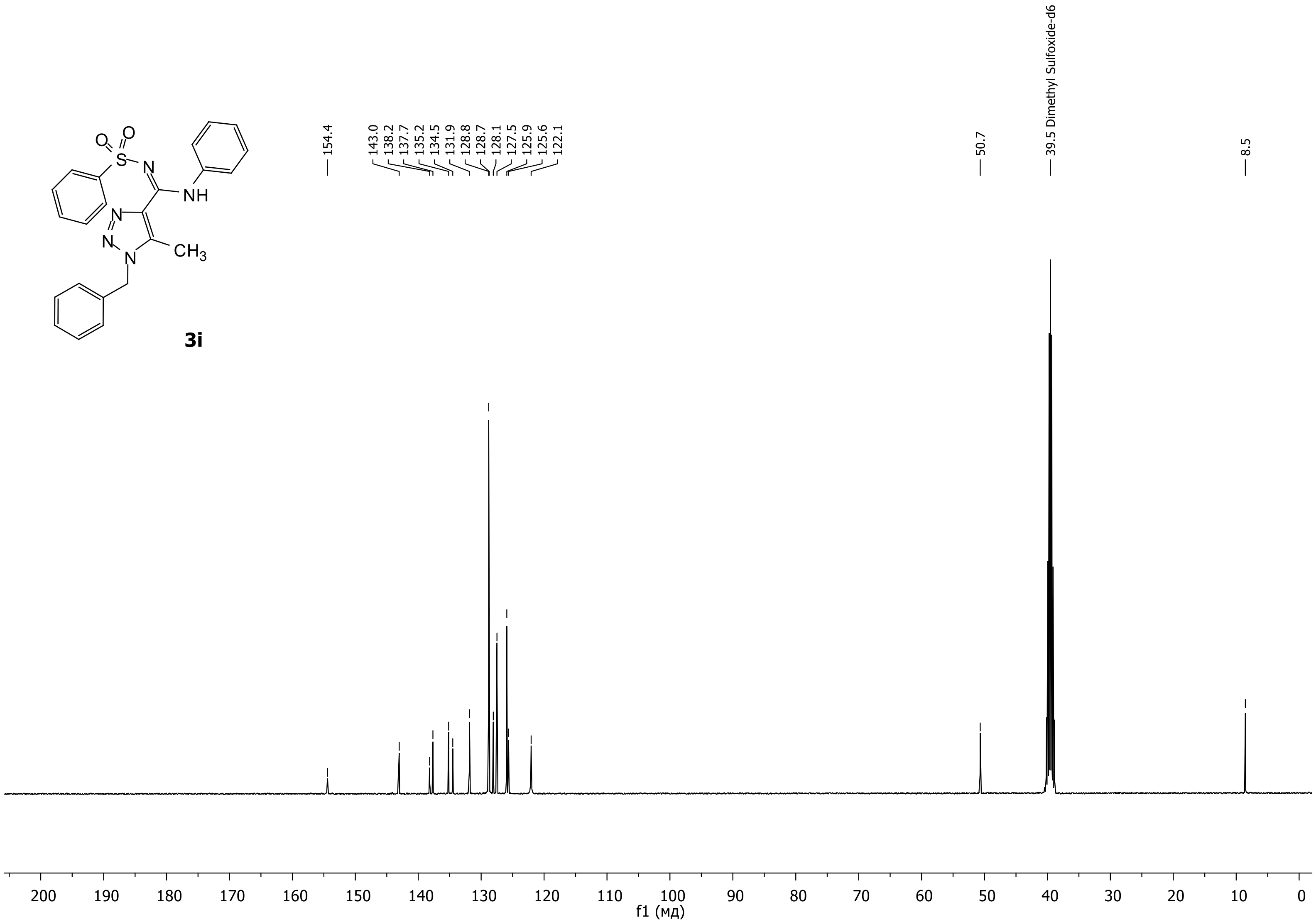

The ^{13}C NMR (100 MHz, DMSO-d_6) spectrum of compound **3i**.

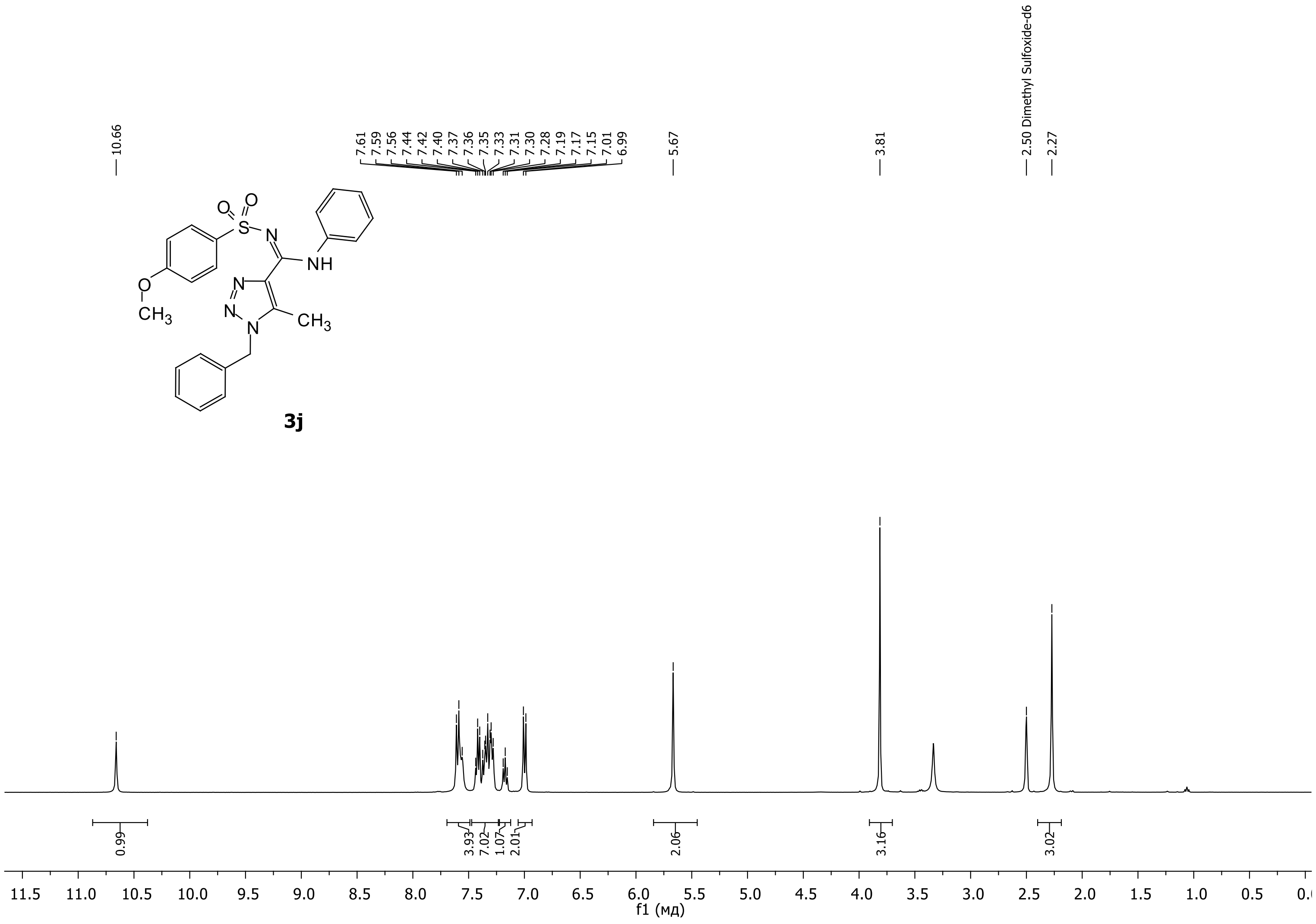

The ^{1}H NMR (400 MHz, DMSO-d_6) spectrum of compound **3j**.

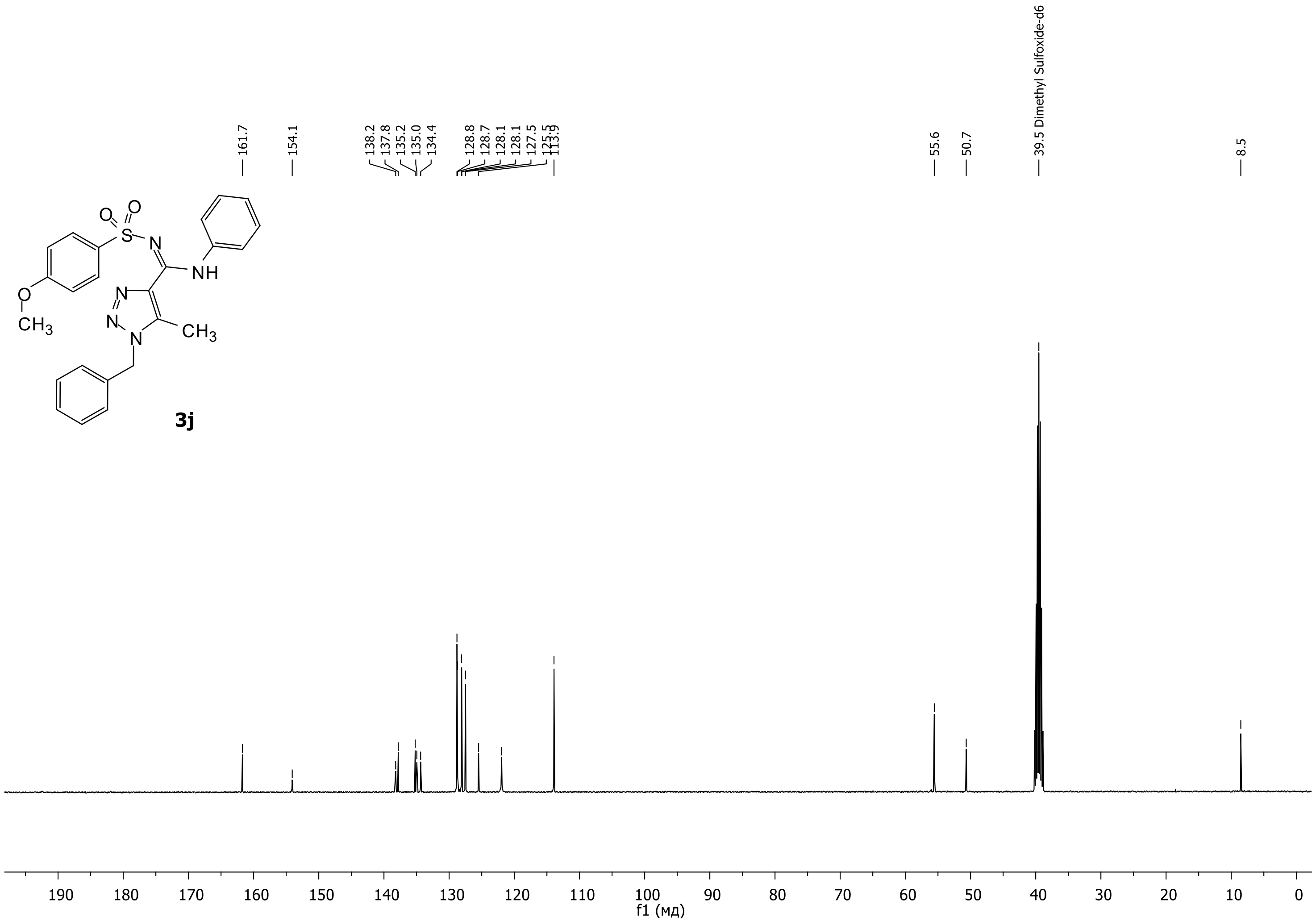

The ^{13}C NMR (100 MHz, DMSO-d_6) spectrum of compound **3j**.

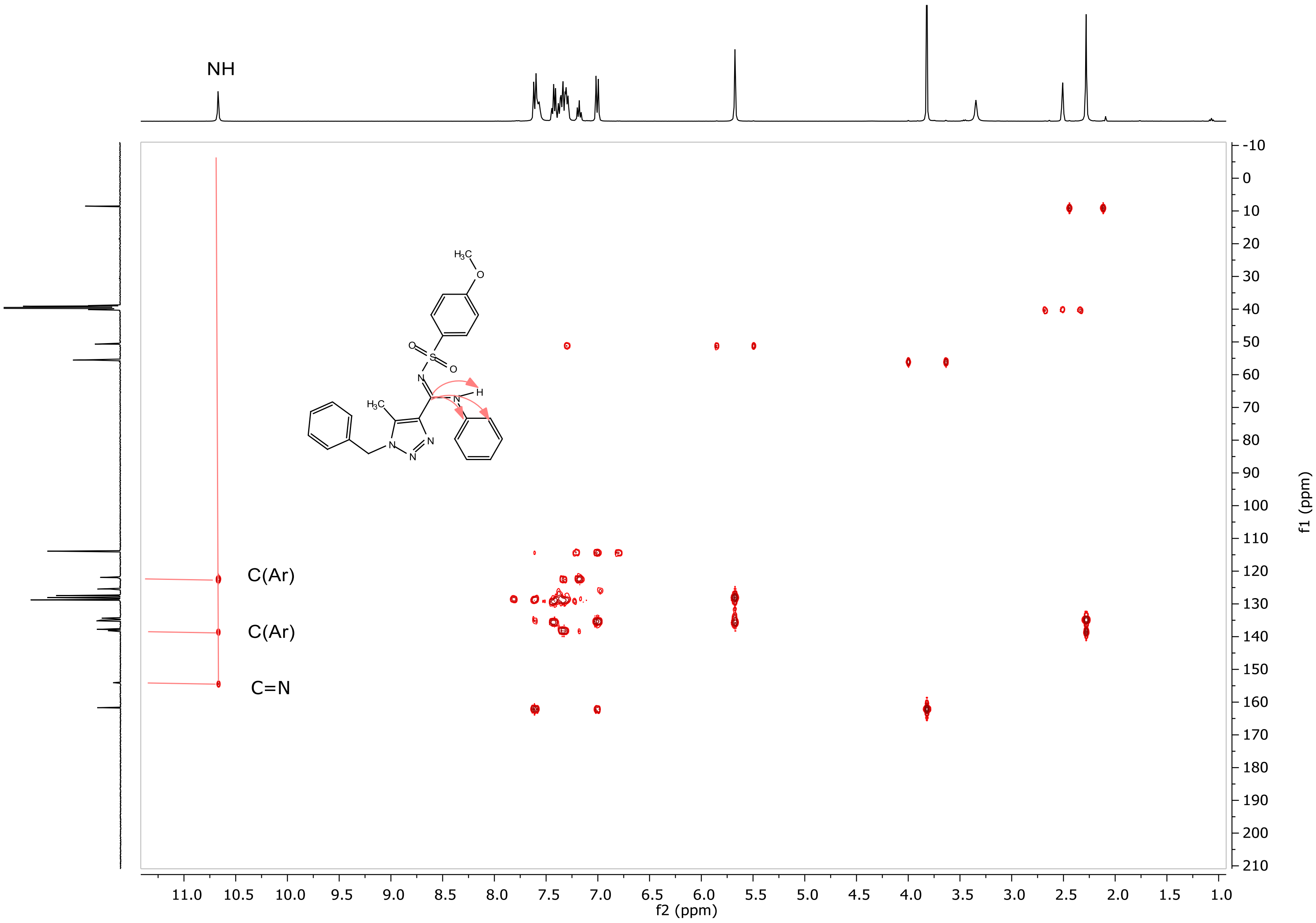

The HMBC NMR spectrum of compound **3j**.

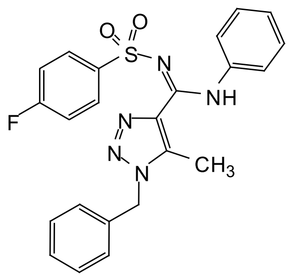

3k

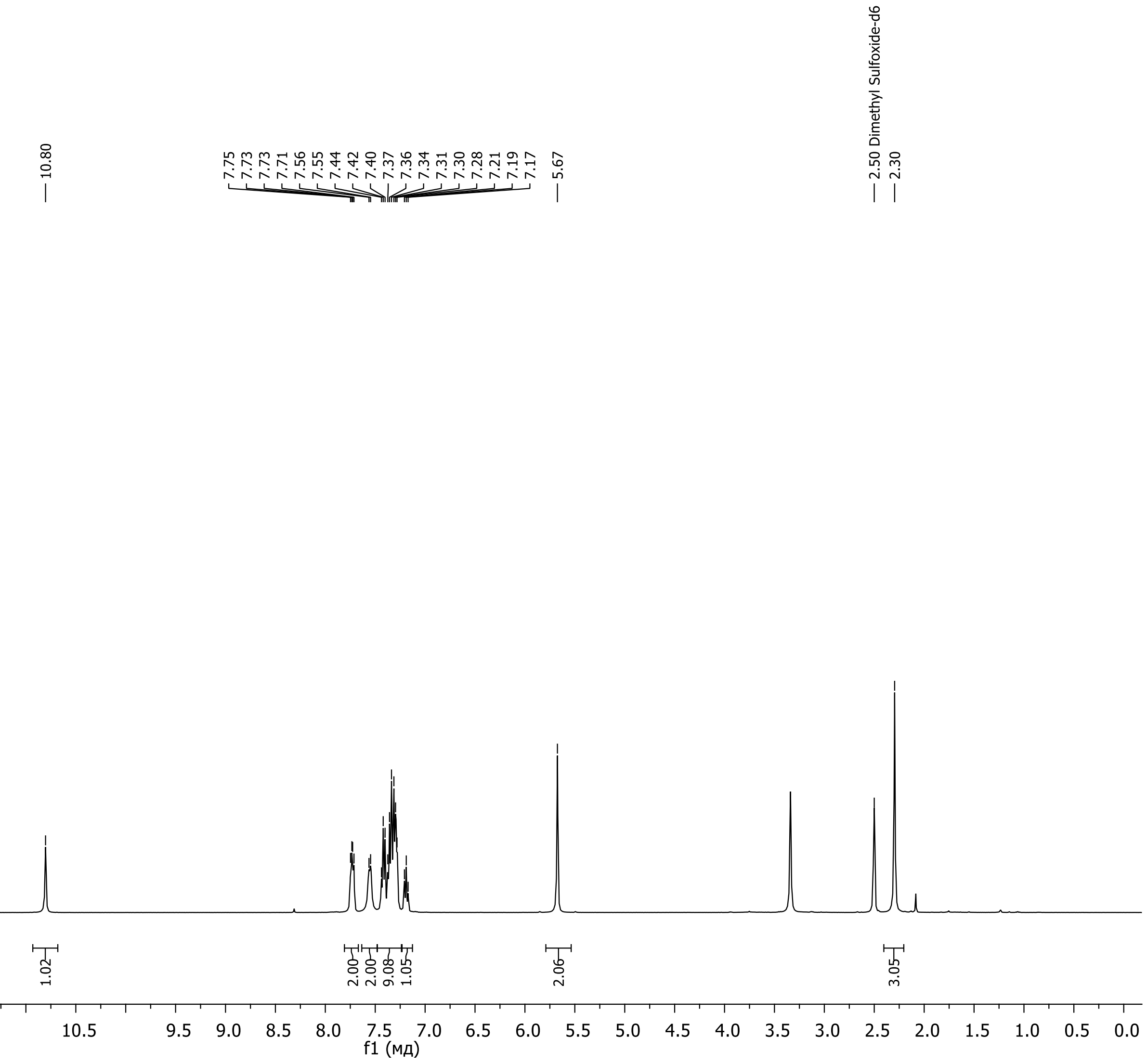

The ^{1}H NMR (400 MHz, DMSO-d_6) spectrum of compound **3k**.

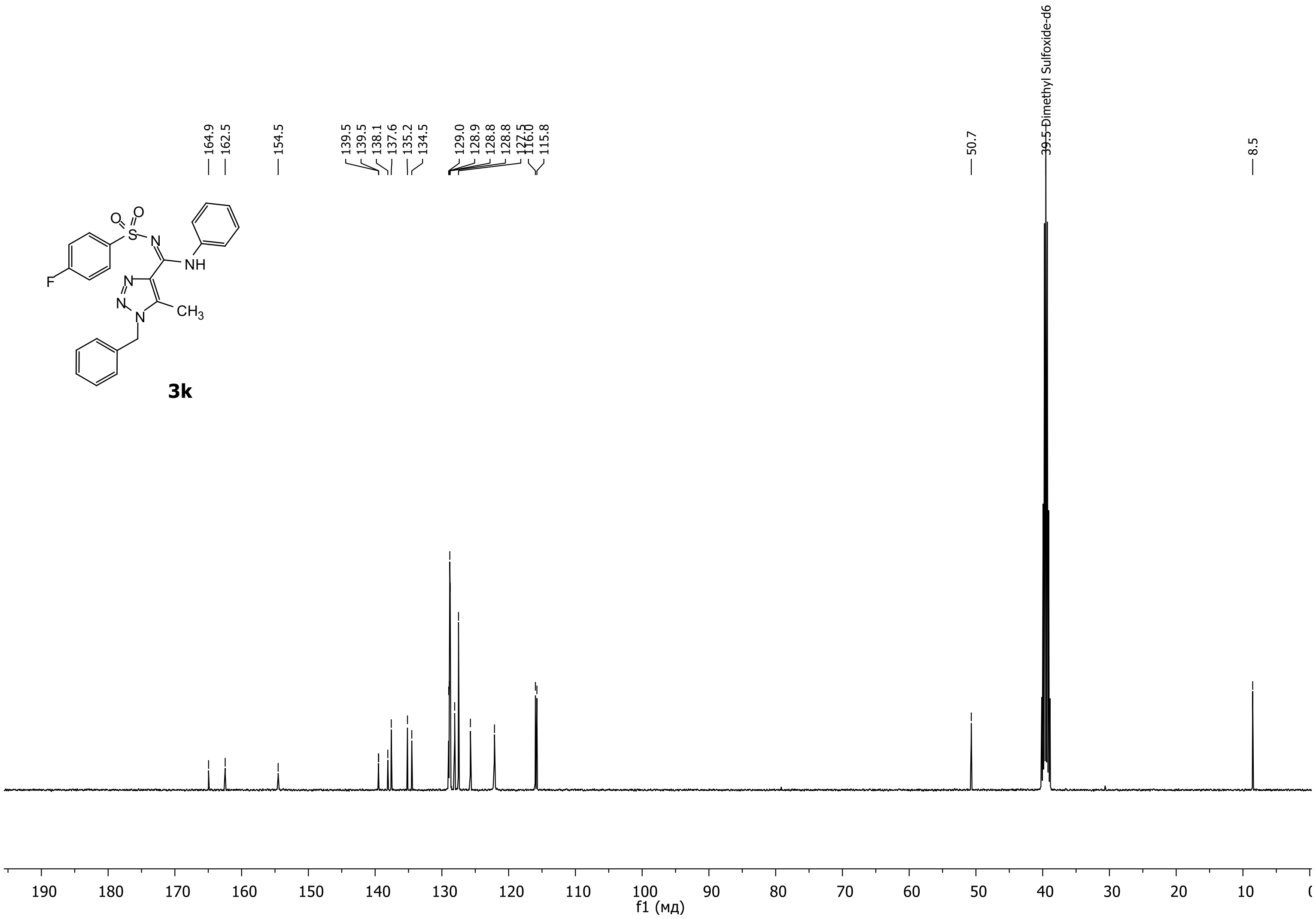

The ^{13}C NMR (100 MHz, DMSO-d_6) spectrum of compound **3k**.

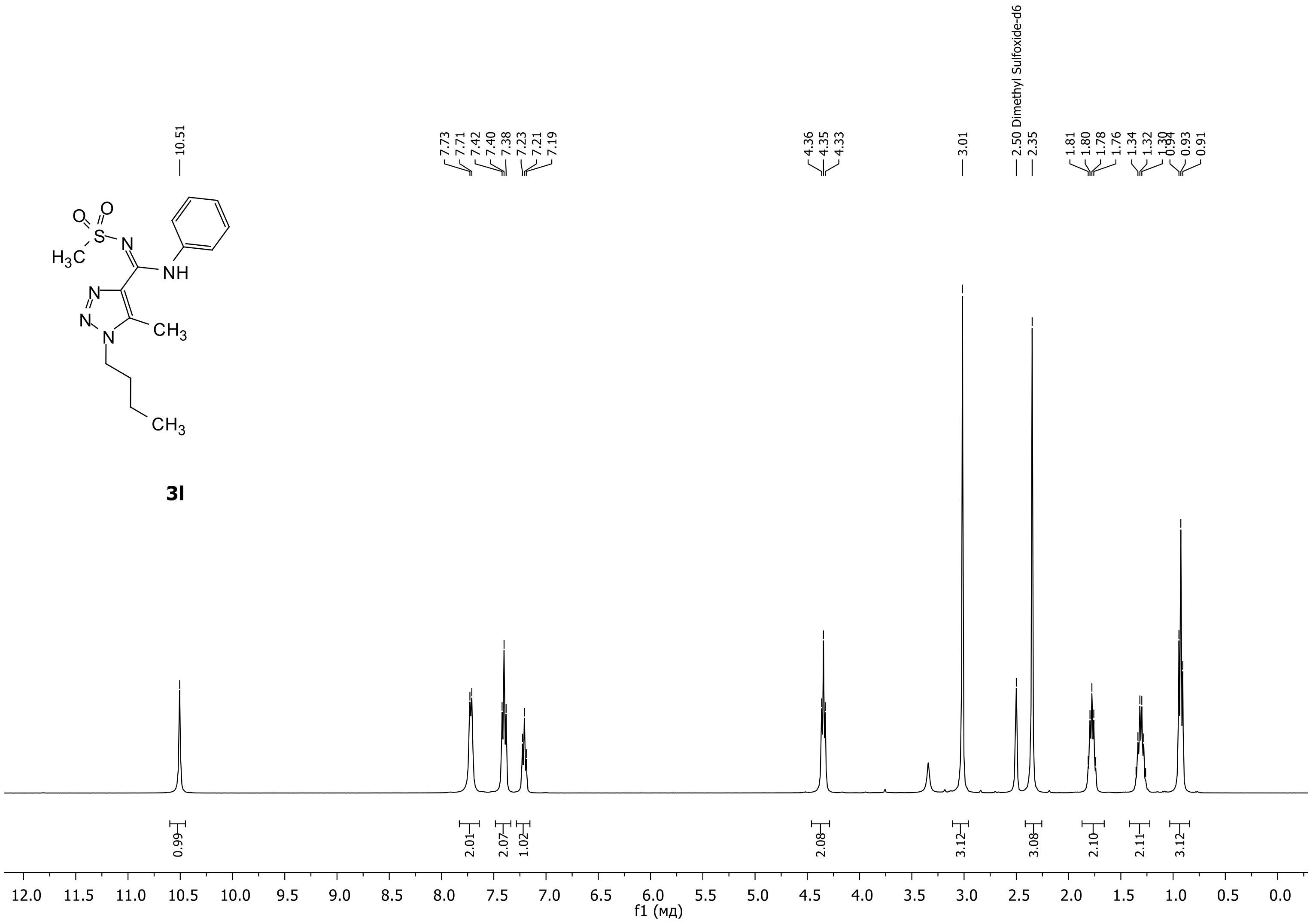

The ^{1}H NMR (400 MHz, DMSO-d_6) spectrum of compound **3l**.

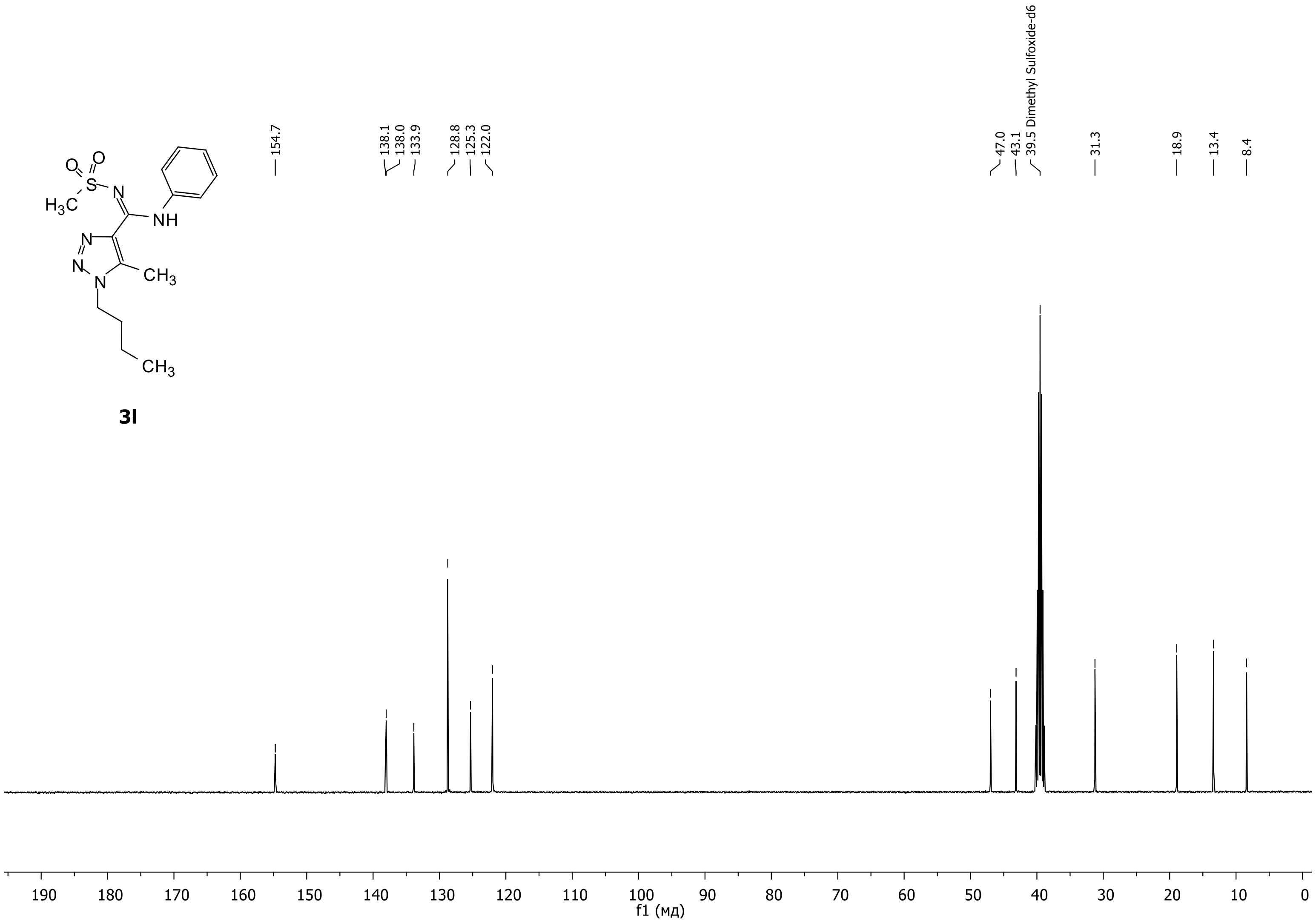

The ^{13}C NMR (100 MHz, DMSO-d_6) spectrum of compound **3l**.

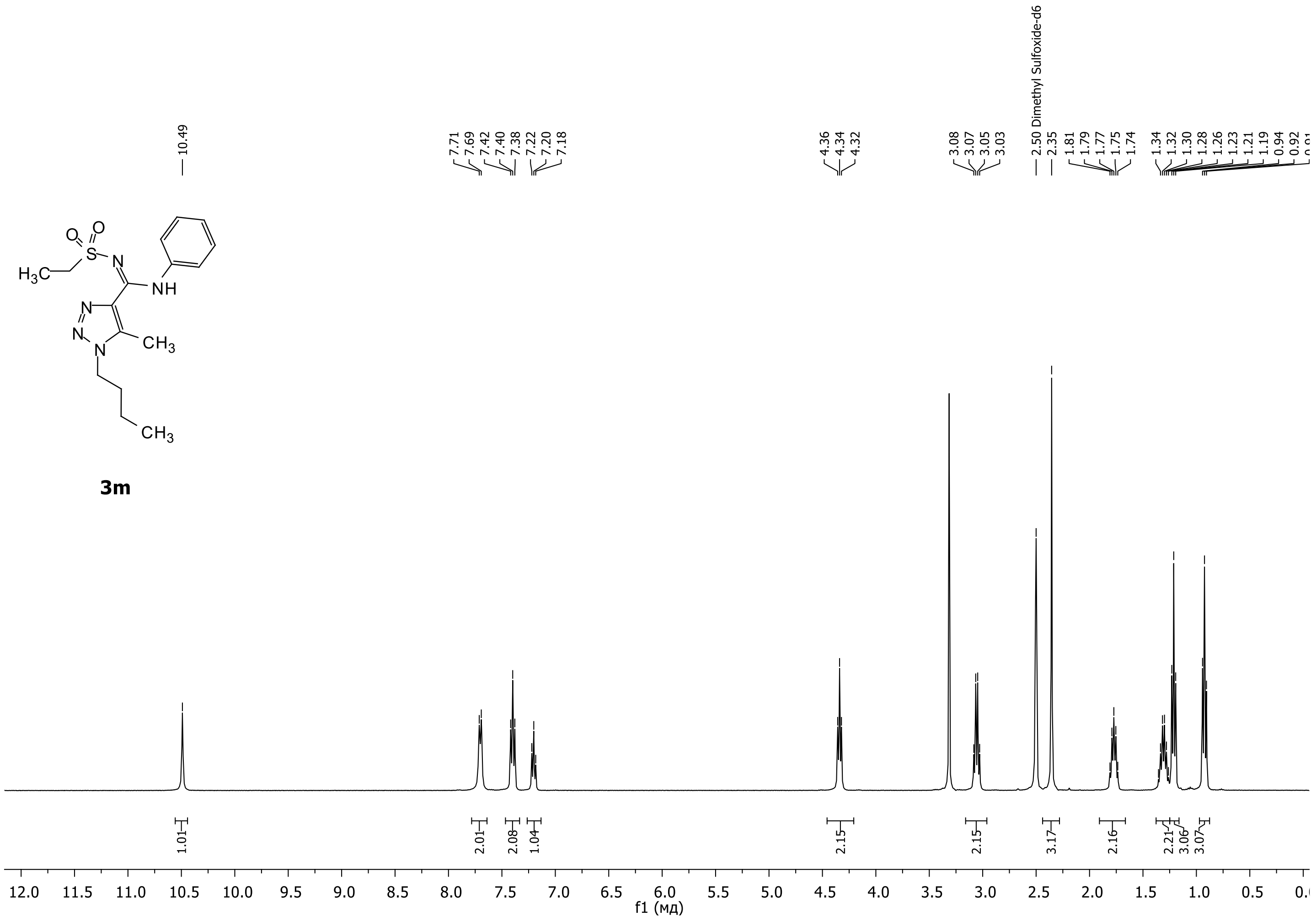

The ^{1}H NMR (400 MHz, DMSO-d_6) spectrum of compound **3m**.

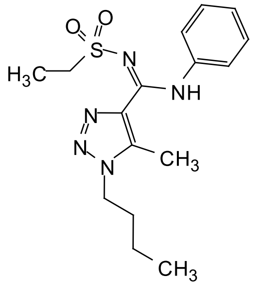

3m

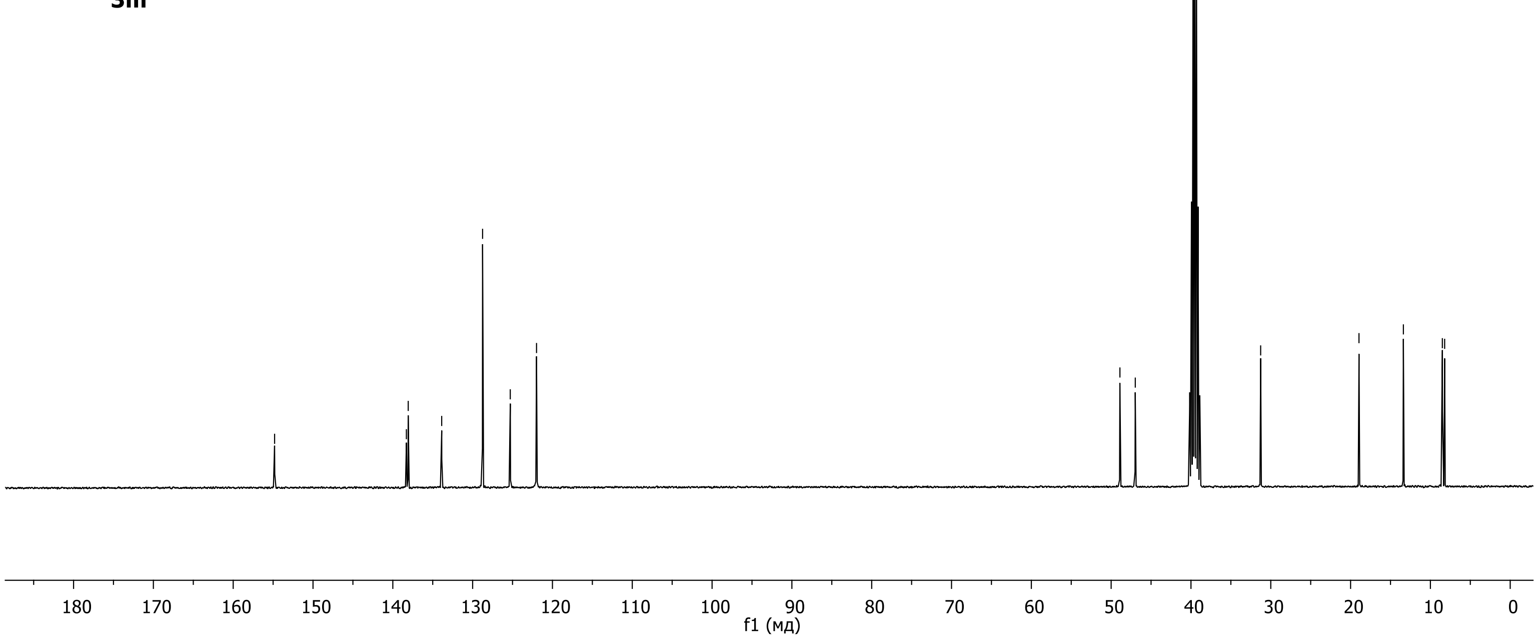

The ^{13}C NMR (100 MHz, DMSO-d_6) spectrum of compound **3m**.

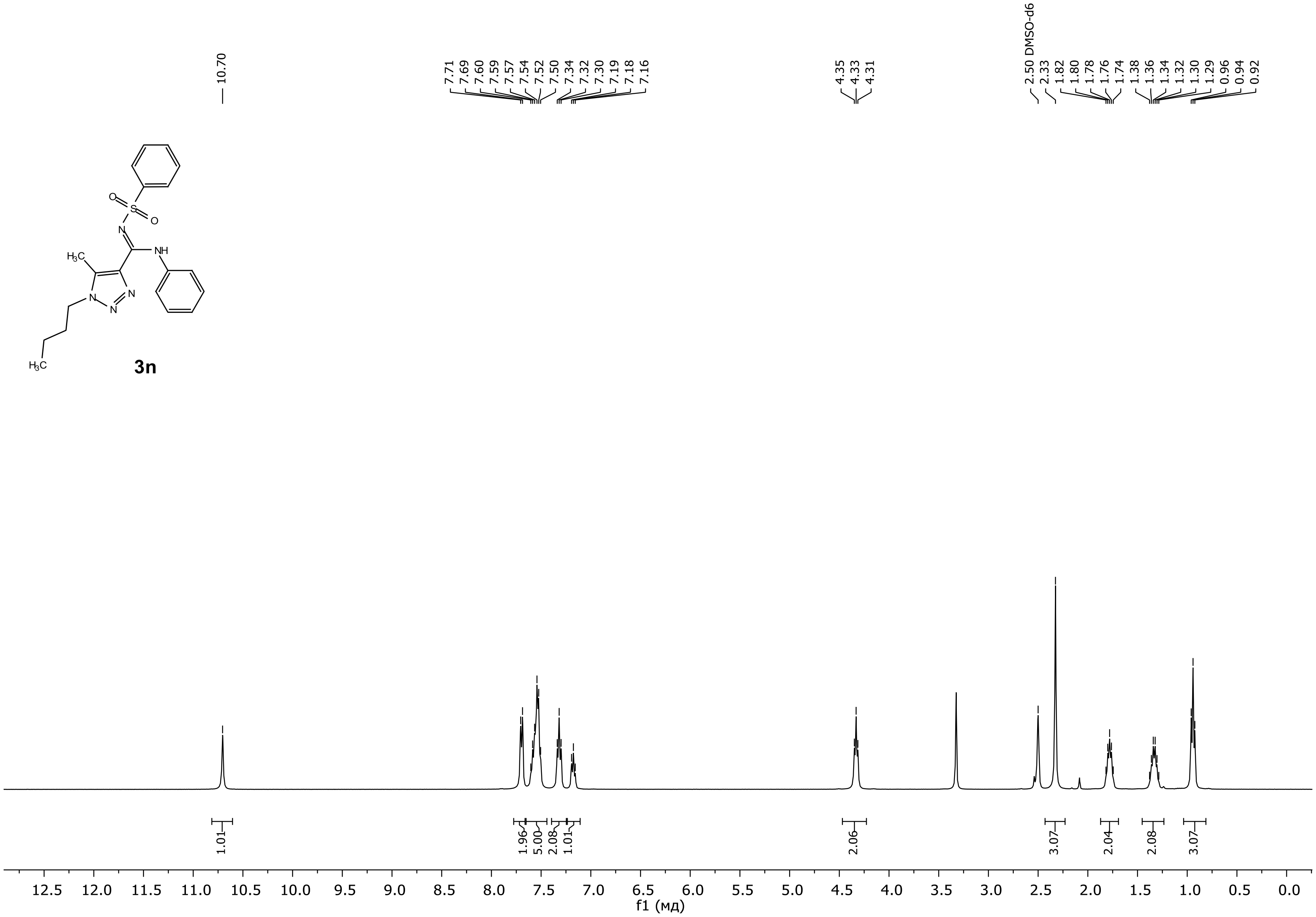

The ^{1}H NMR (400 MHz, DMSO-d_6) spectrum of compound **3n**.

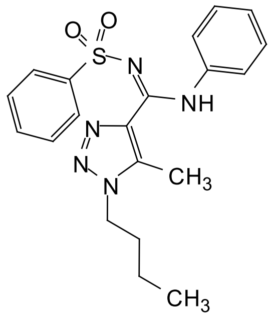

3n

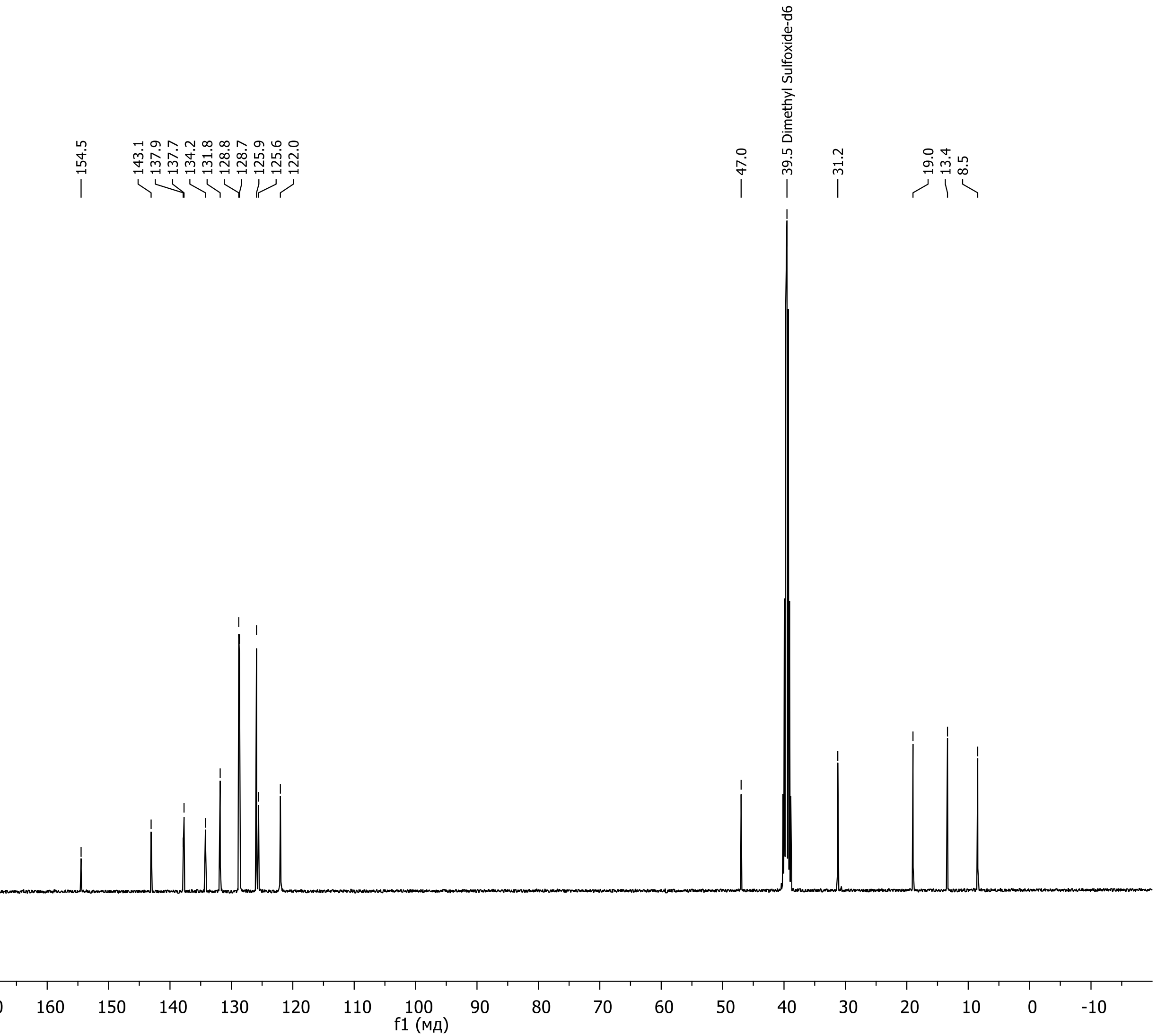

The ^{13}C NMR (100 MHz, DMSO-d_6) spectrum of compound **3n**.

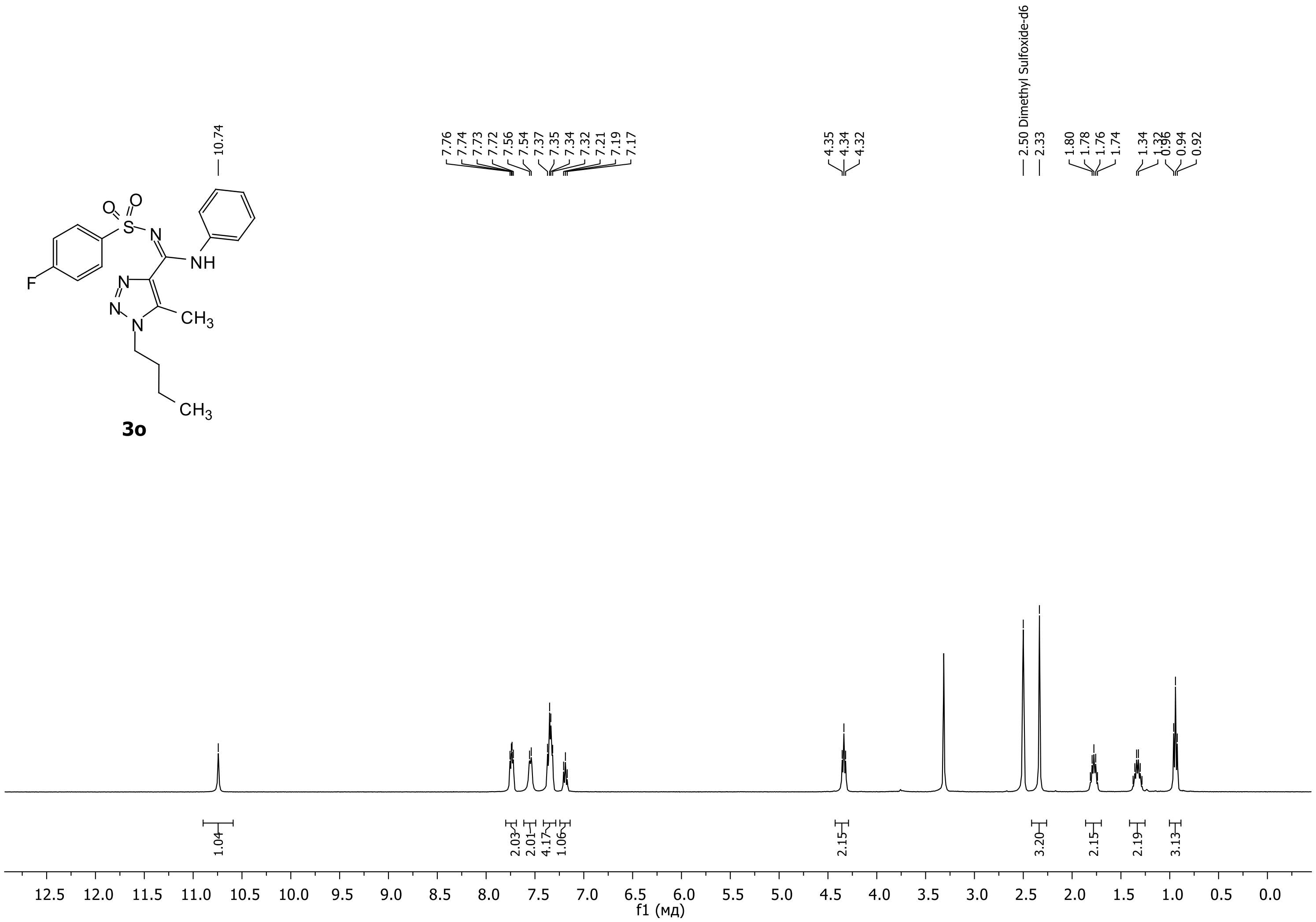

The ^{1}H NMR (400 MHz, DMSO-d_6) spectrum of compound **3o**.

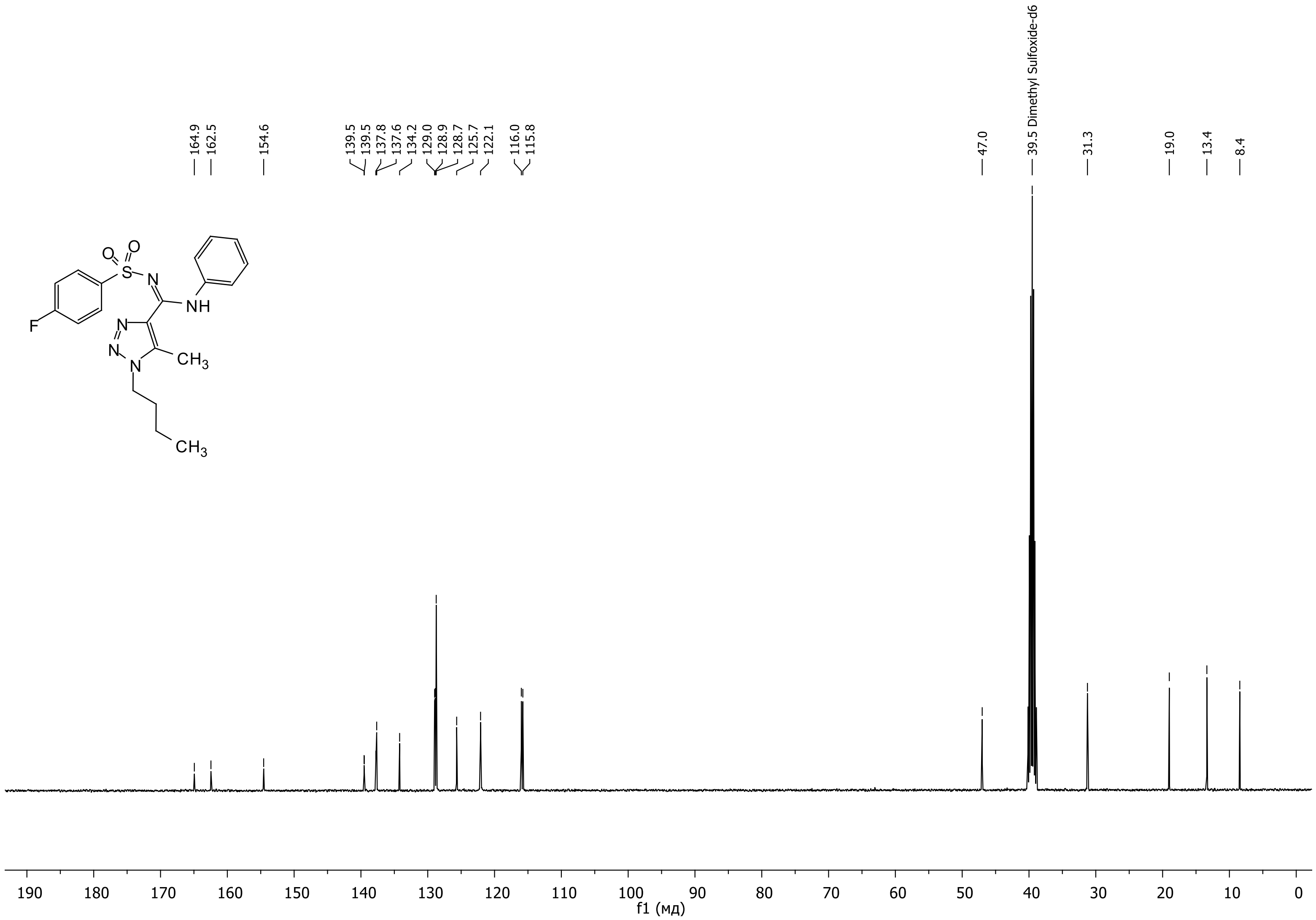

The ^{13}C NMR (100 MHz, DMSO-d_6) spectrum of compound **3o**.

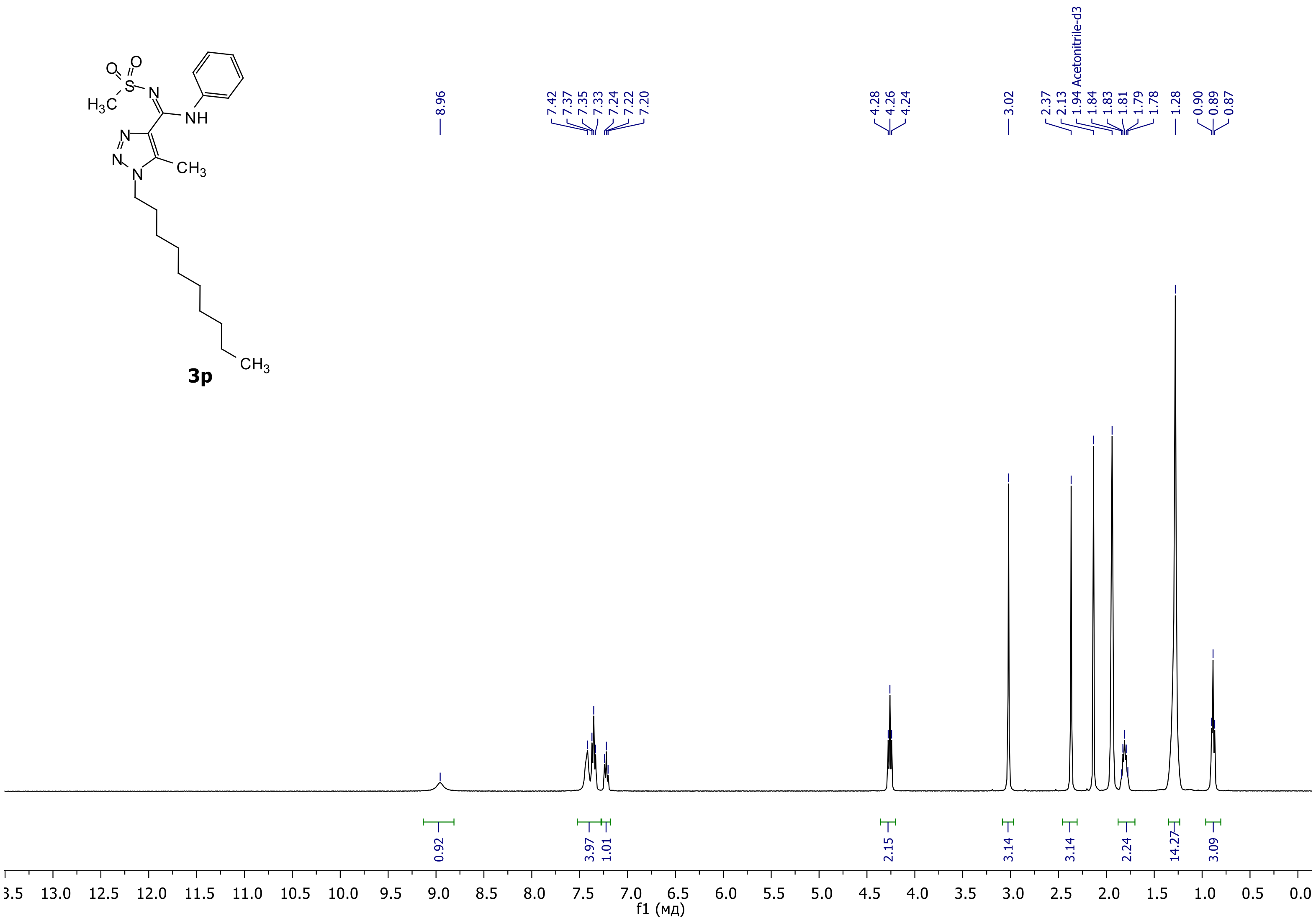

The ^{1}H NMR (400 MHz, CD_3CN, 40 $^{\circ}C$) spectrum of compound **3p**.

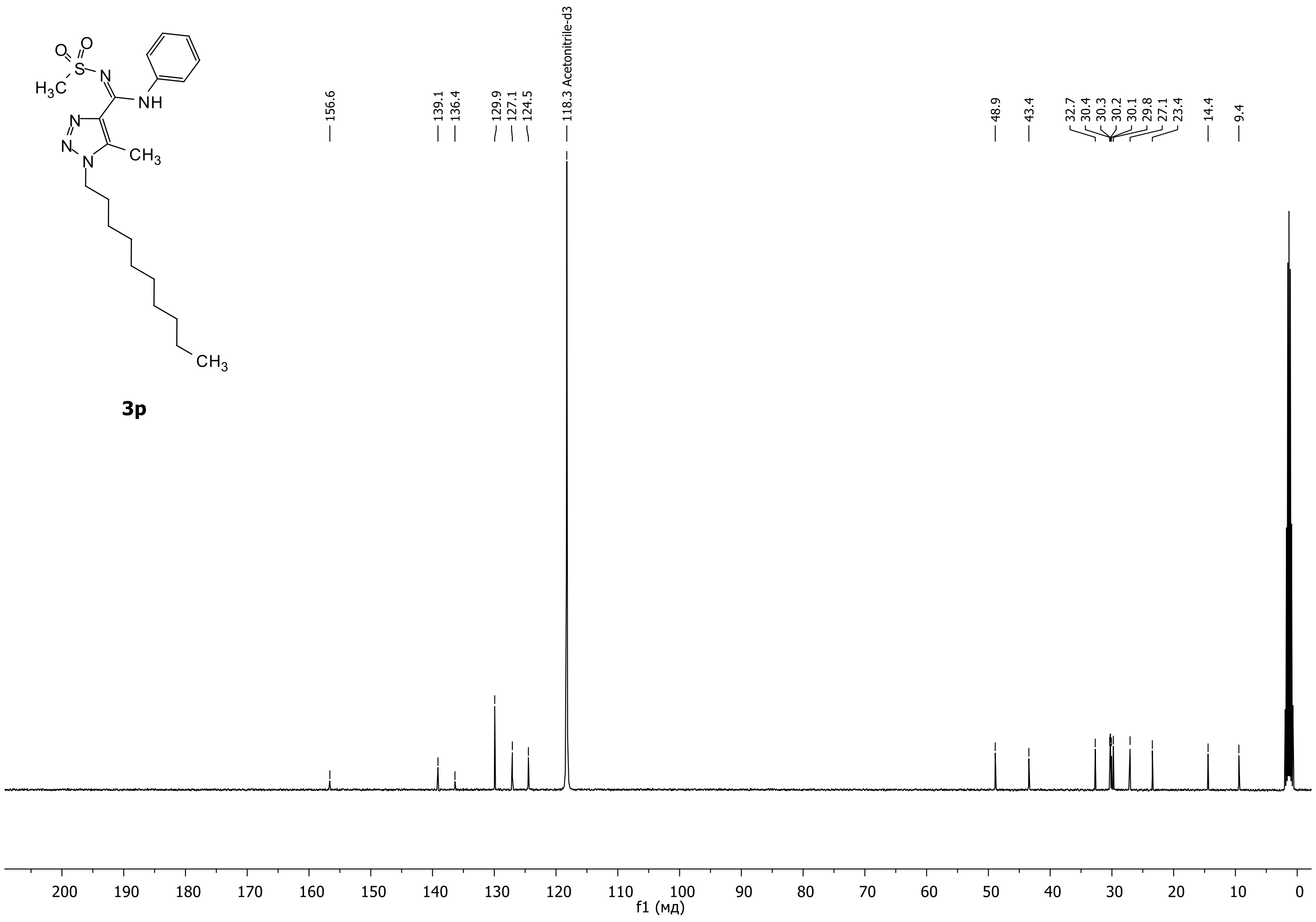

The ^{13}C NMR (100 MHz, CD_3CN, 40 °C) spectrum of compound **3p**.

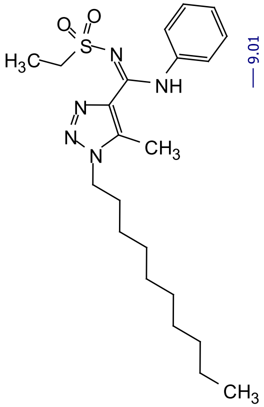

3q

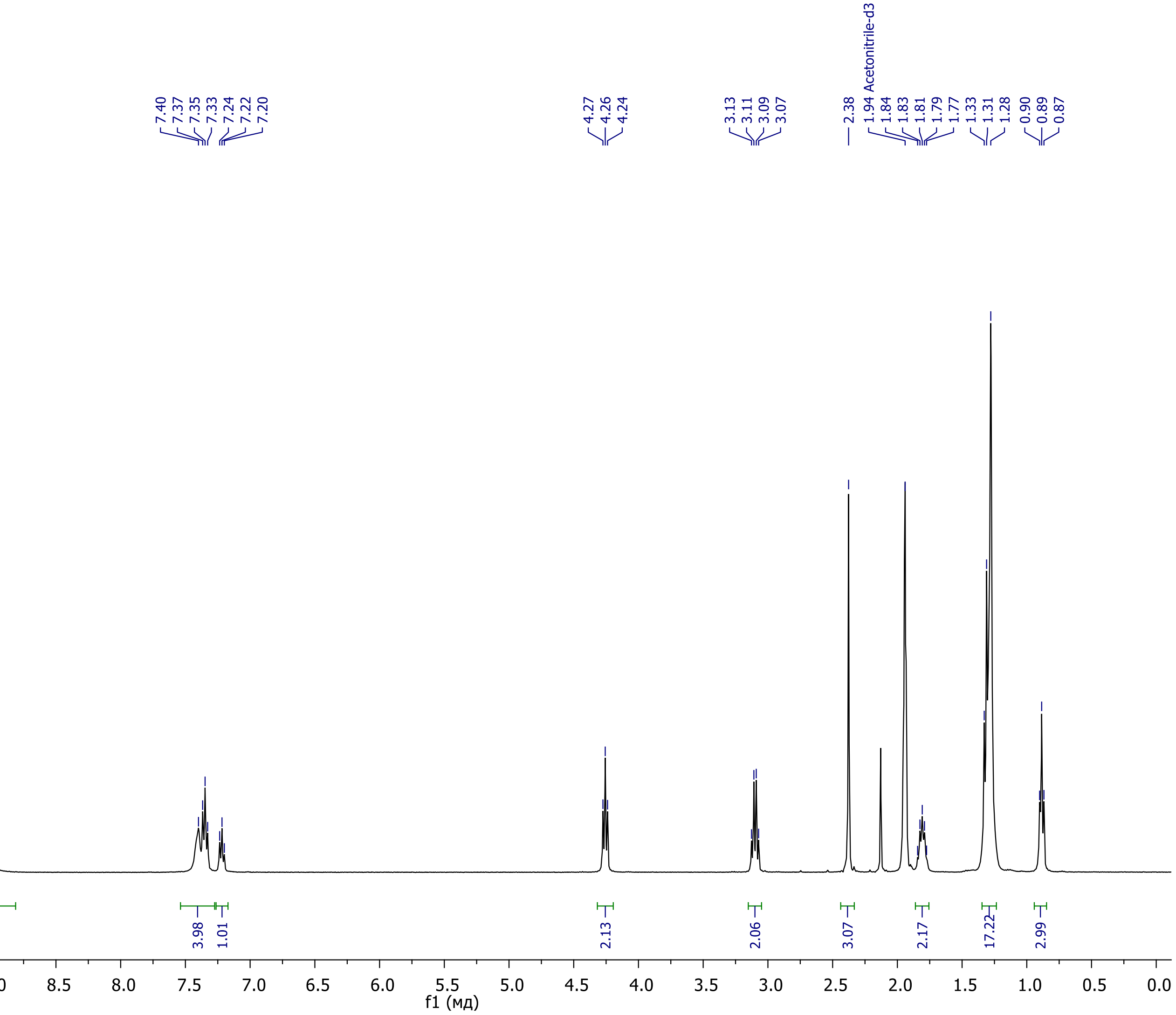

The ^{1}H NMR (400 MHz, CD_3CN) spectrum of compound **3q**.

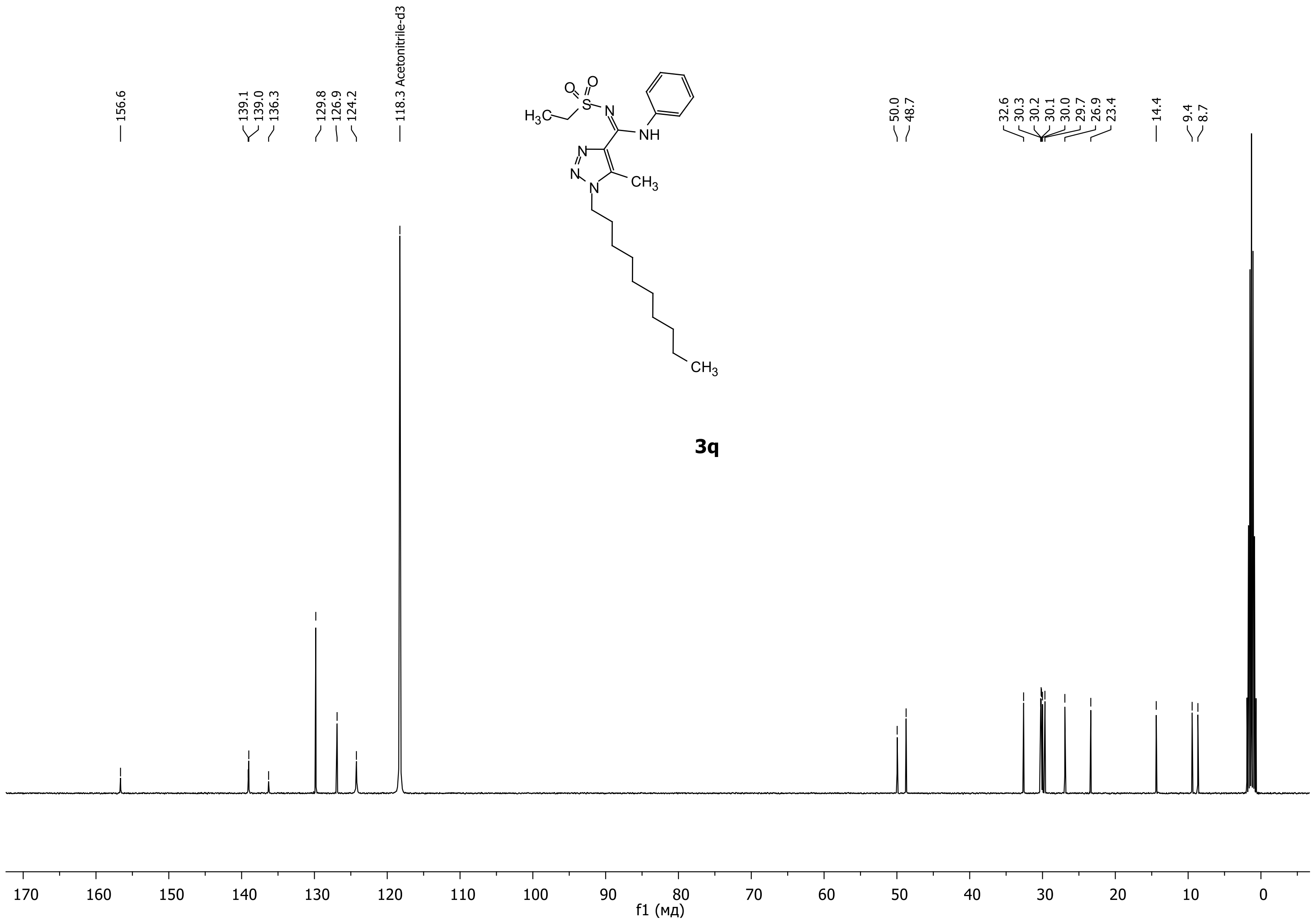

The ^{13}C NMR (100 MHz, CD_3CN) spectrum of compound **3q**.

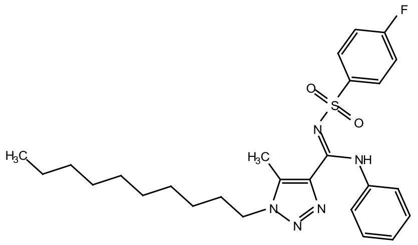

3r

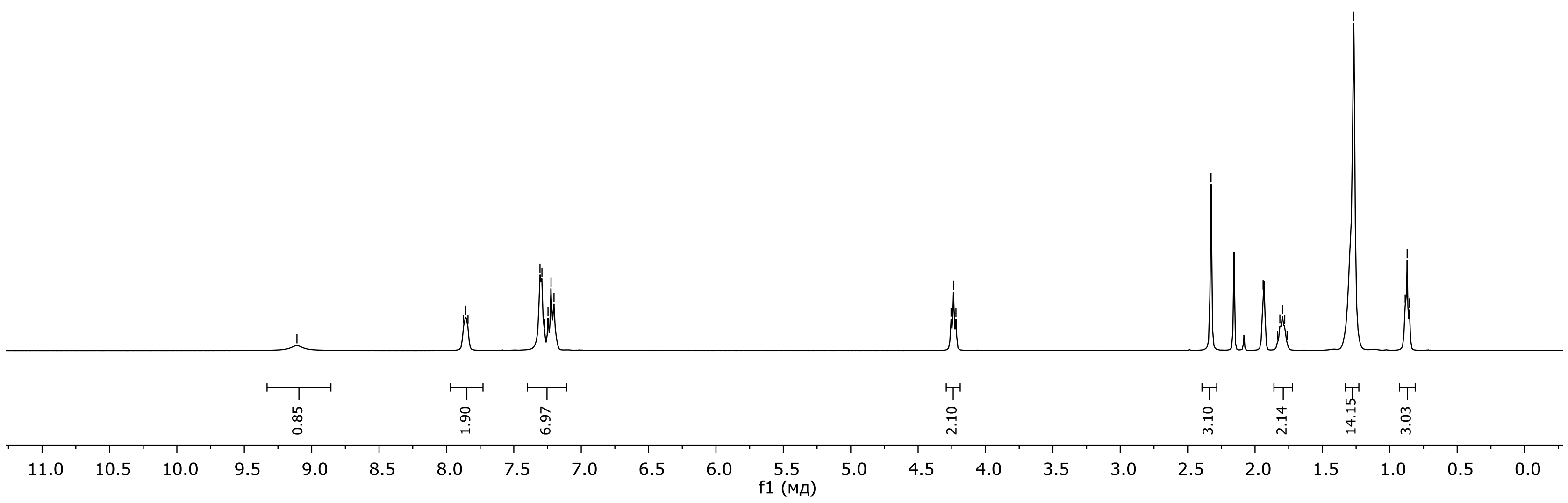

The ^{1}H NMR (400 MHz, CD_3CN) spectrum of compound **3r**.

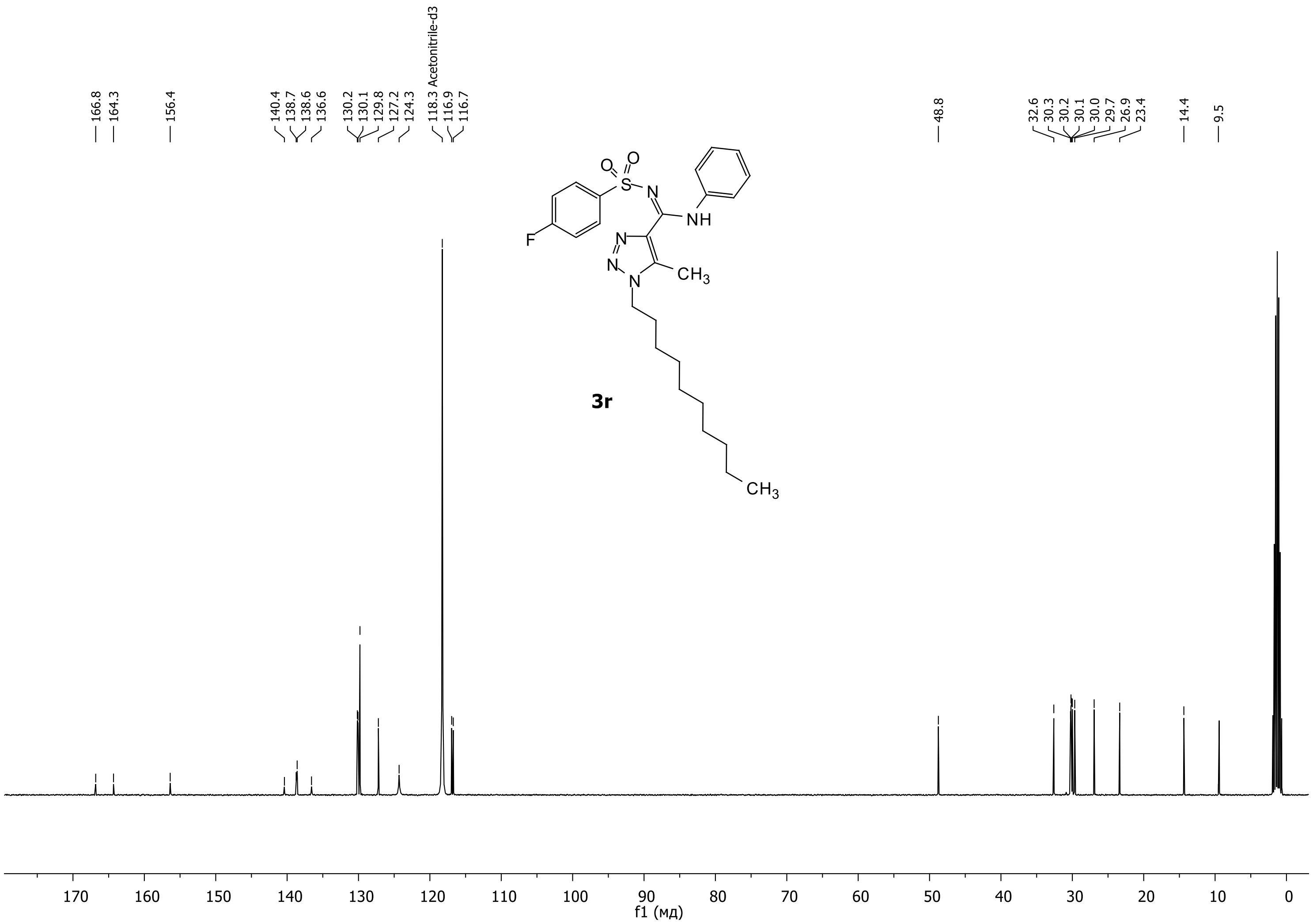

The ^{13}C NMR (100 MHz, CD_3CN) spectrum of compound **3r**.

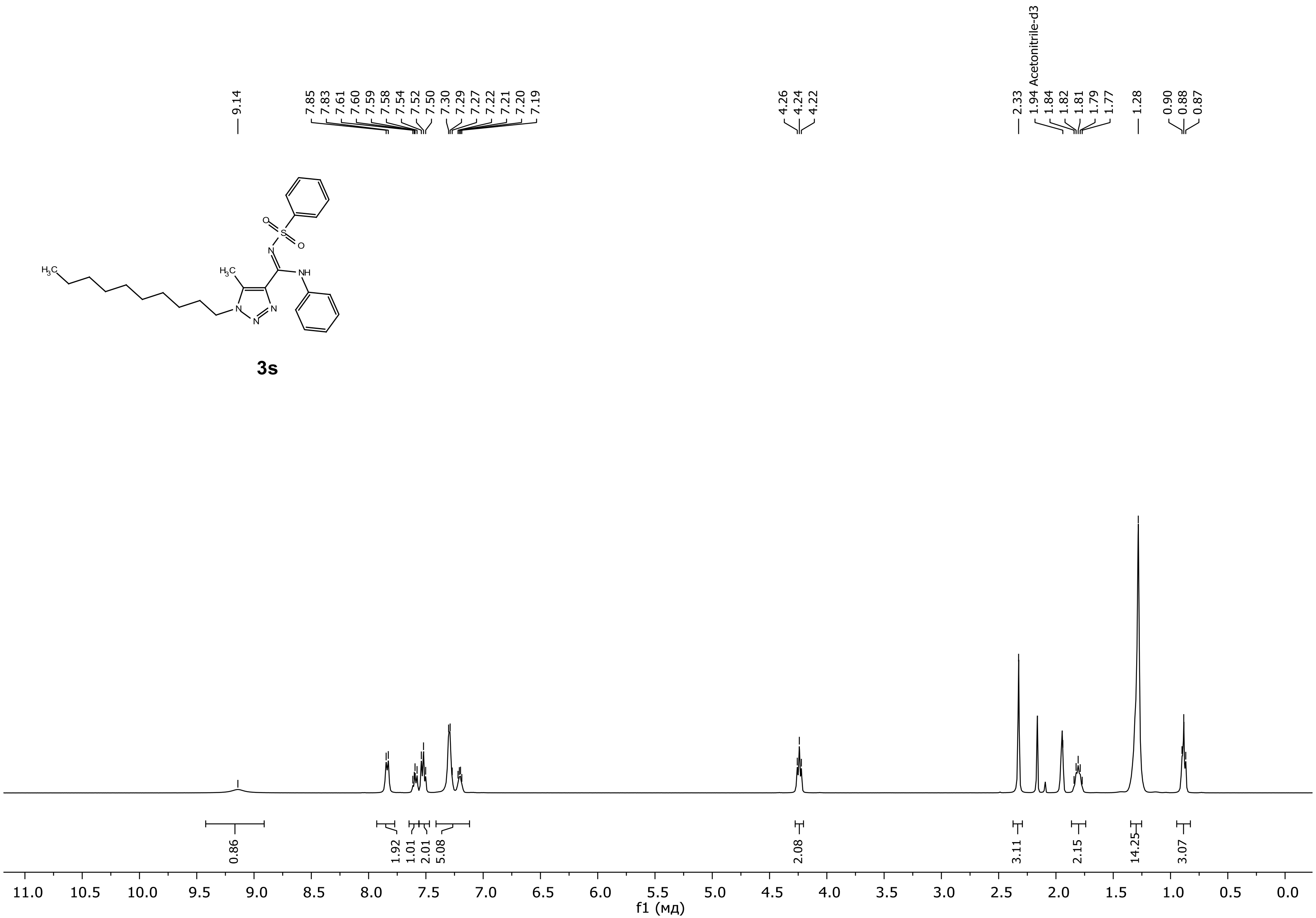

The ^{1}H NMR (400 MHz, CD_3CN) spectrum of compound **3s**.

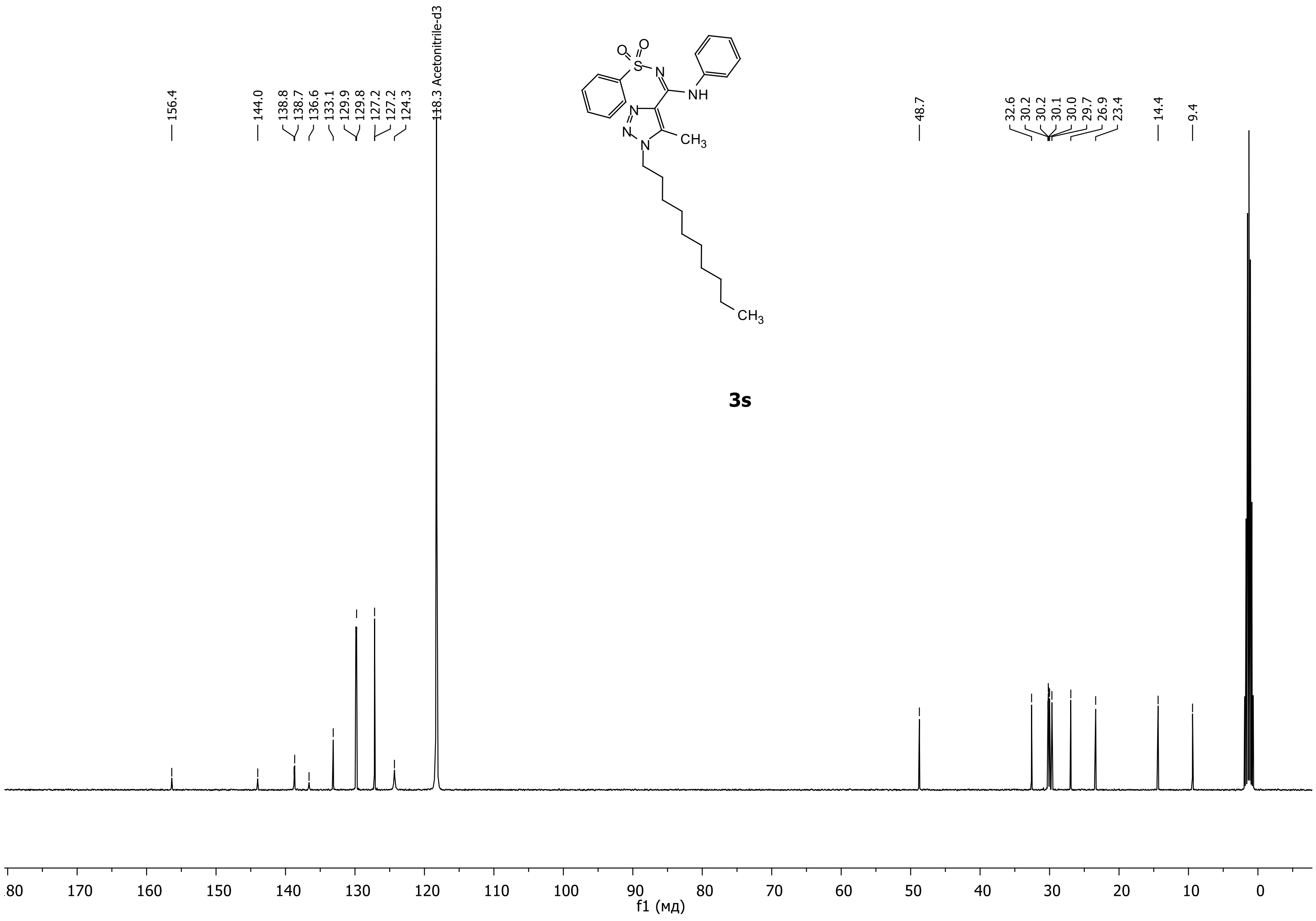

The ^{13}C NMR (100 MHz, CD_3CN) spectrum of compound **3s**.

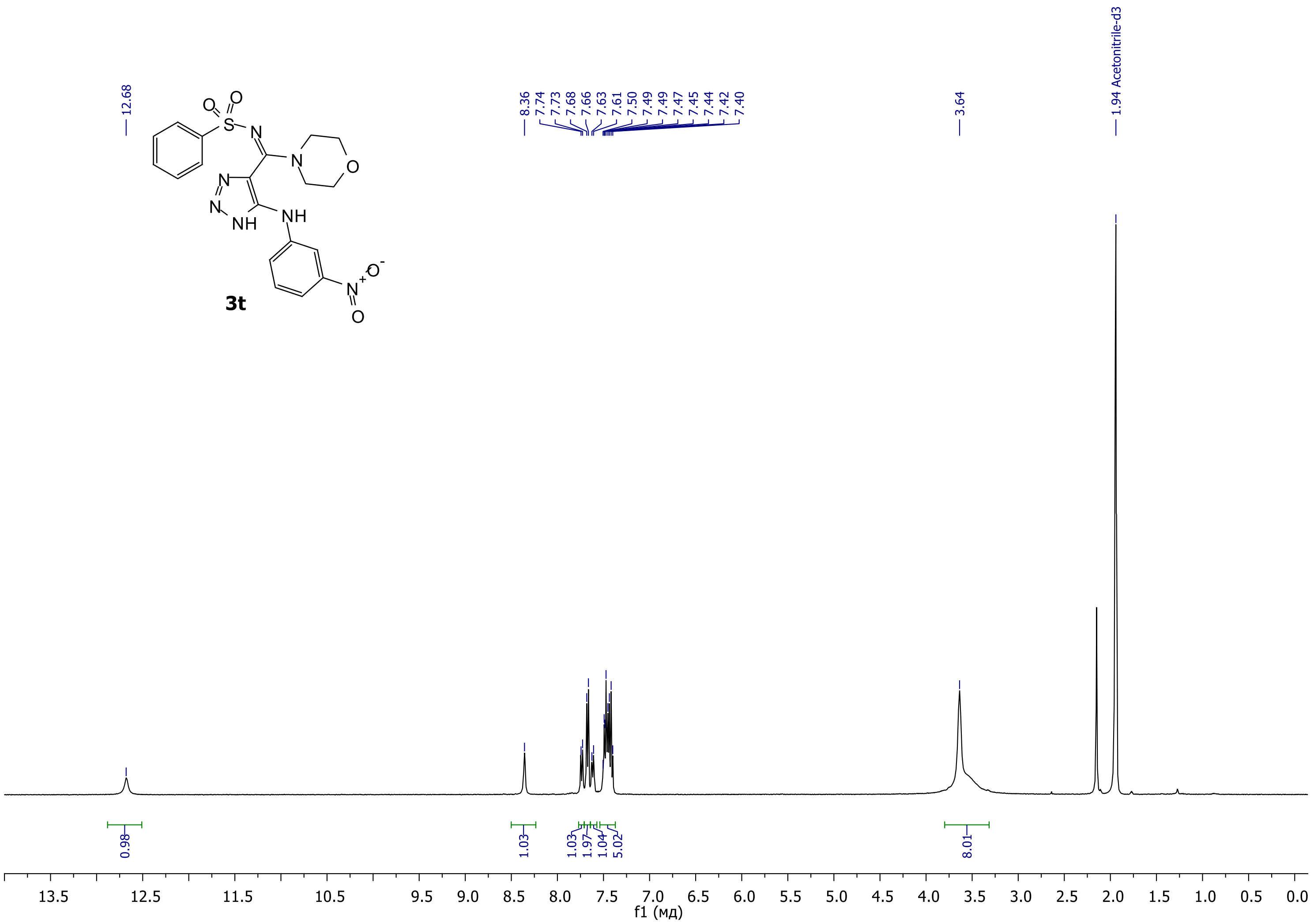

The ^{1}H NMR (400 MHz, CD_3CN) spectrum of compound **3t**.

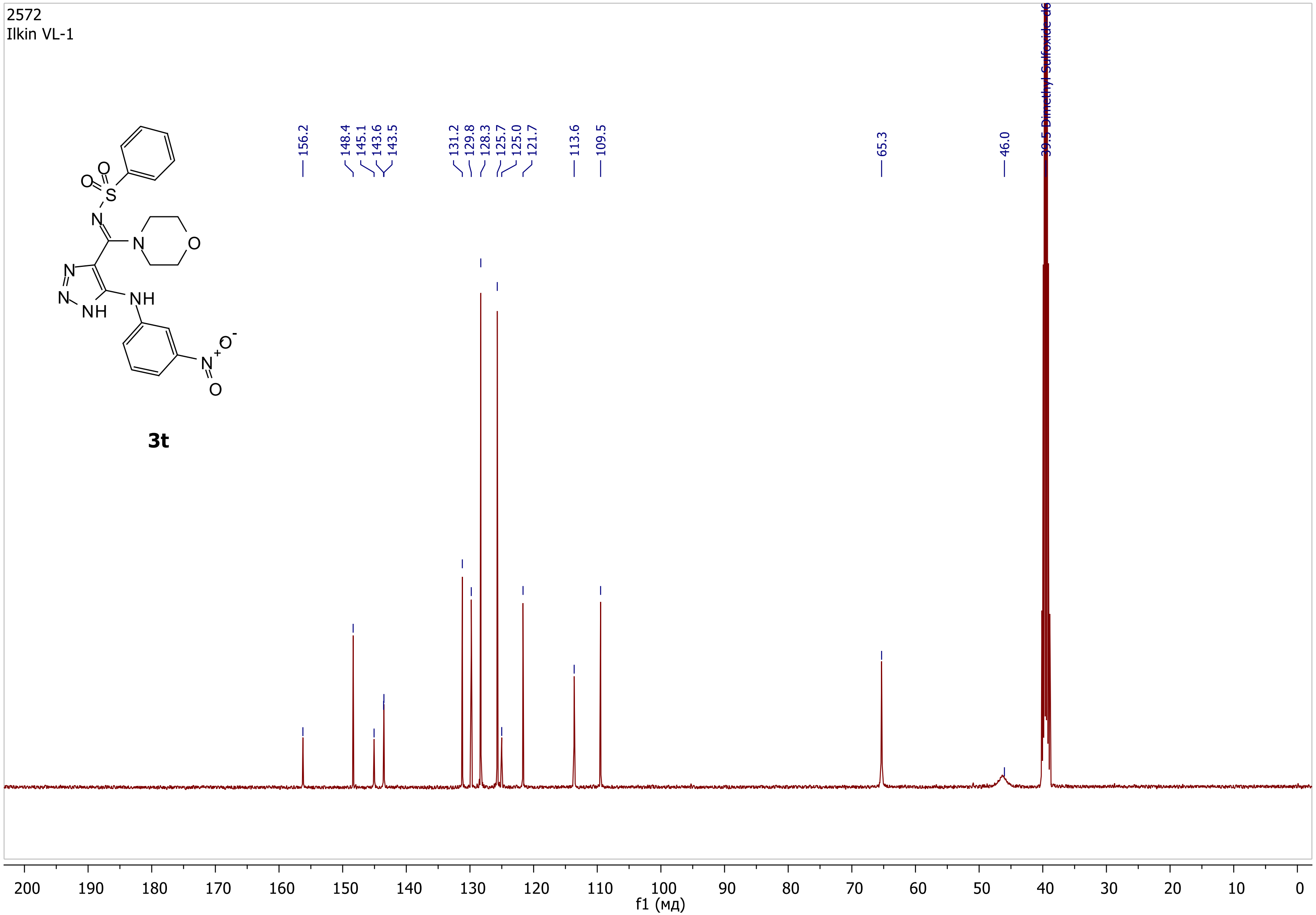

The ^{13}C NMR (100 MHz, DMSO-d_6, 50 °C) spectrum of compound **3t**.

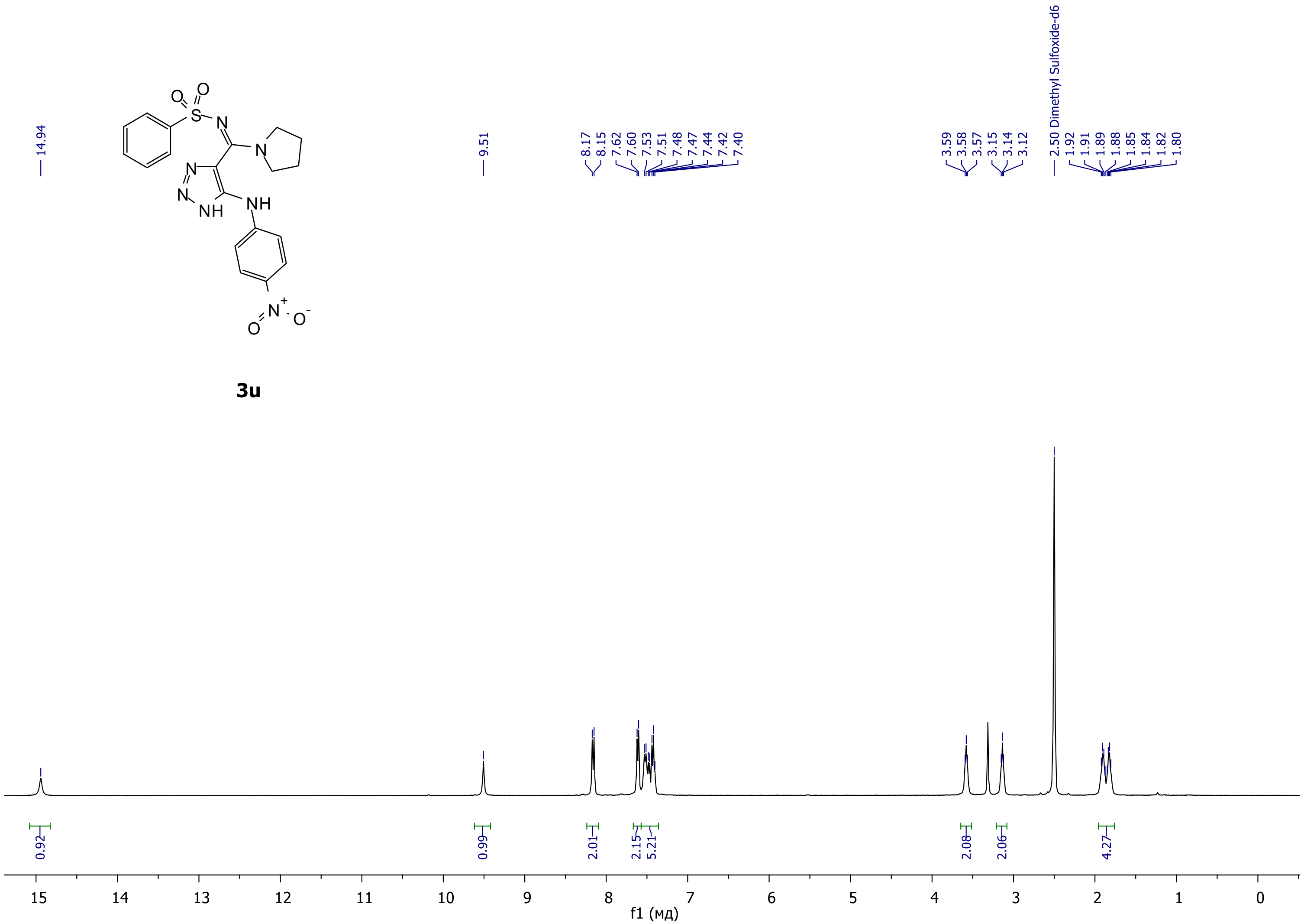

The ^{1}H NMR (400 MHz, DMSO-d_6) spectrum of compound **3u**.

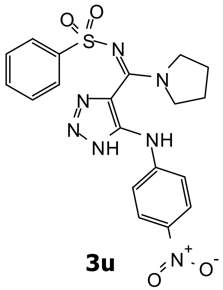

3u

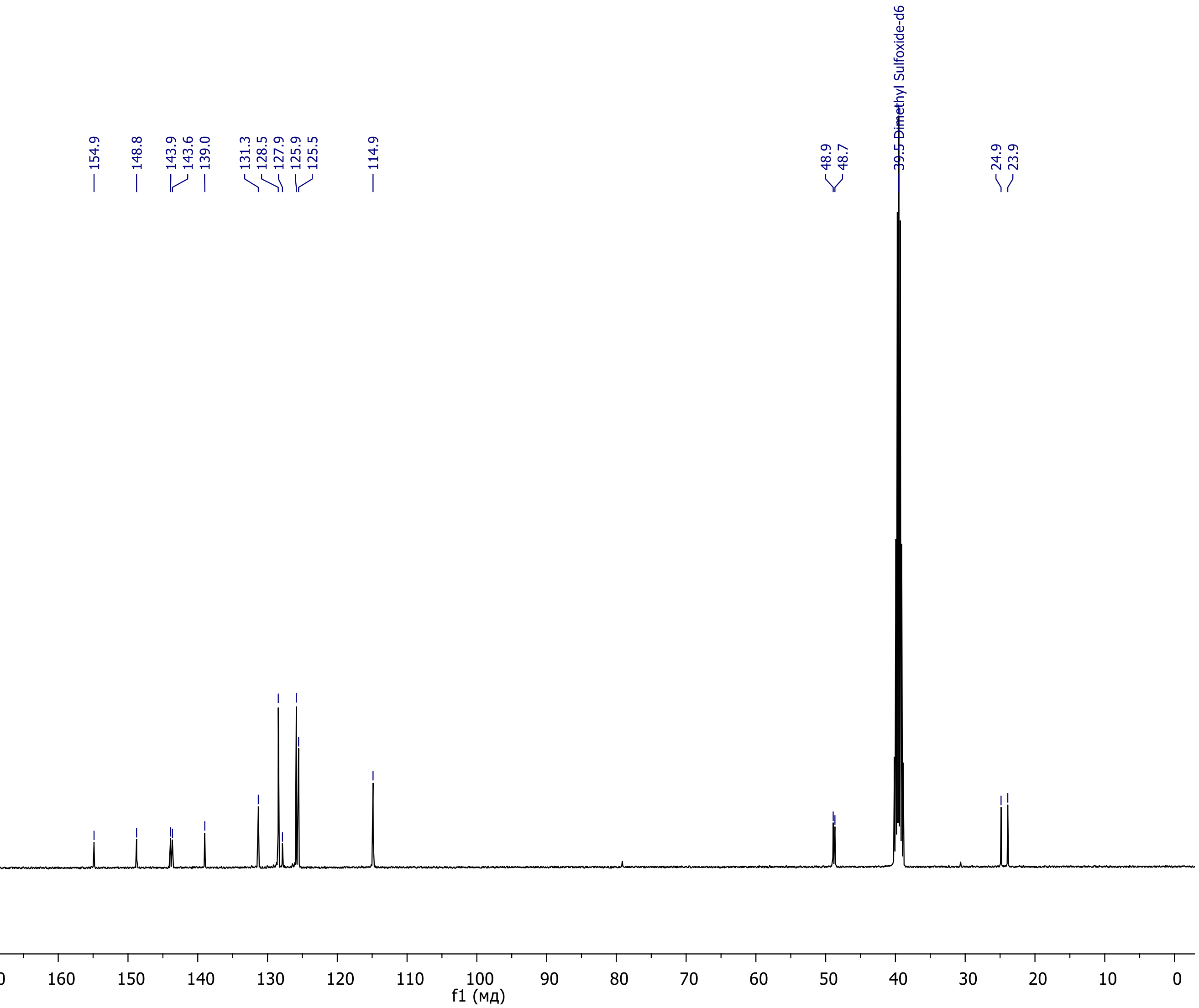

The ^{13}C NMR (100 MHz, DMSO-d_6) spectrum of compound **3u**.

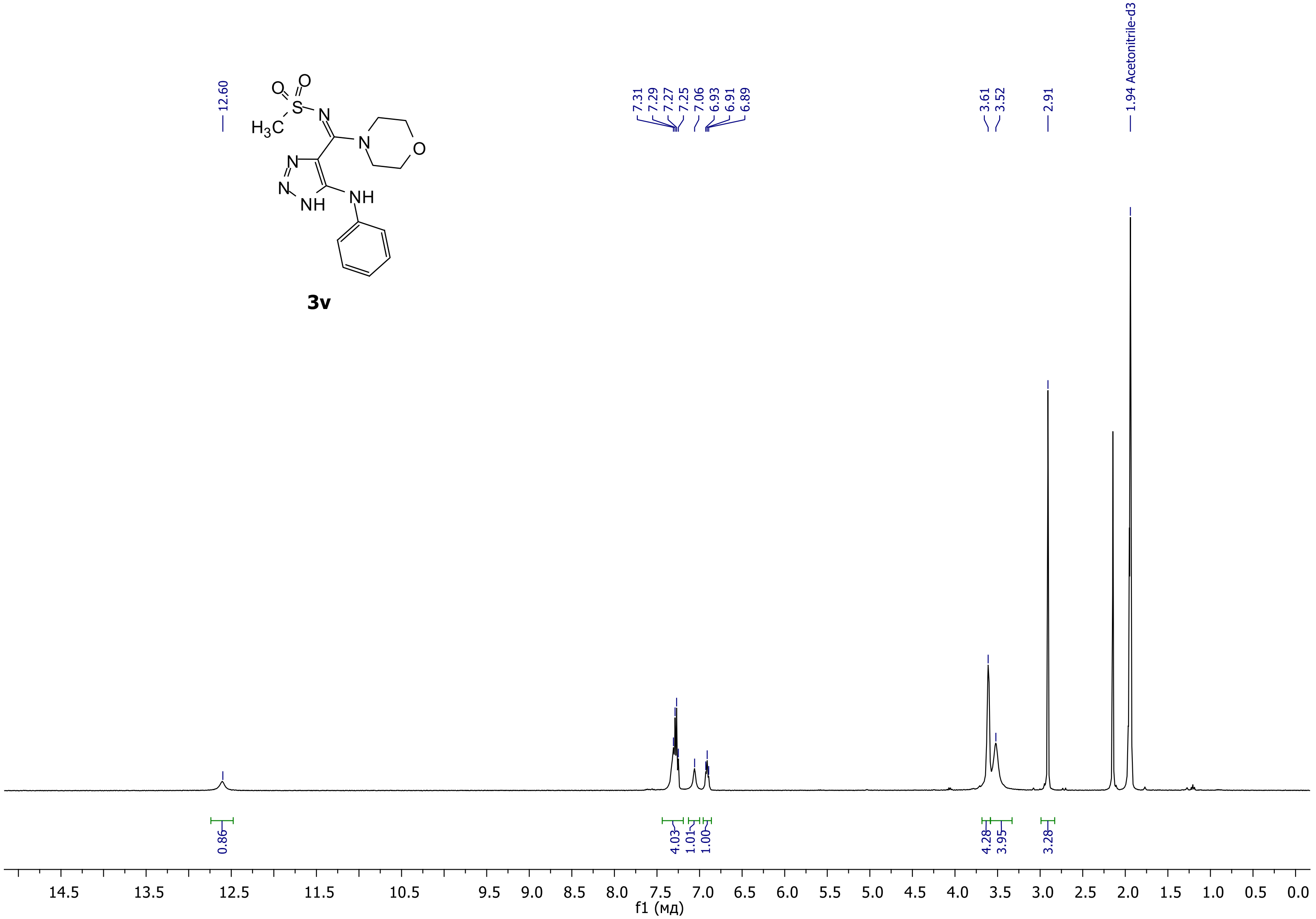

The ^{1}H NMR (400 MHz, CD_3CN) spectrum of compound **3v**.

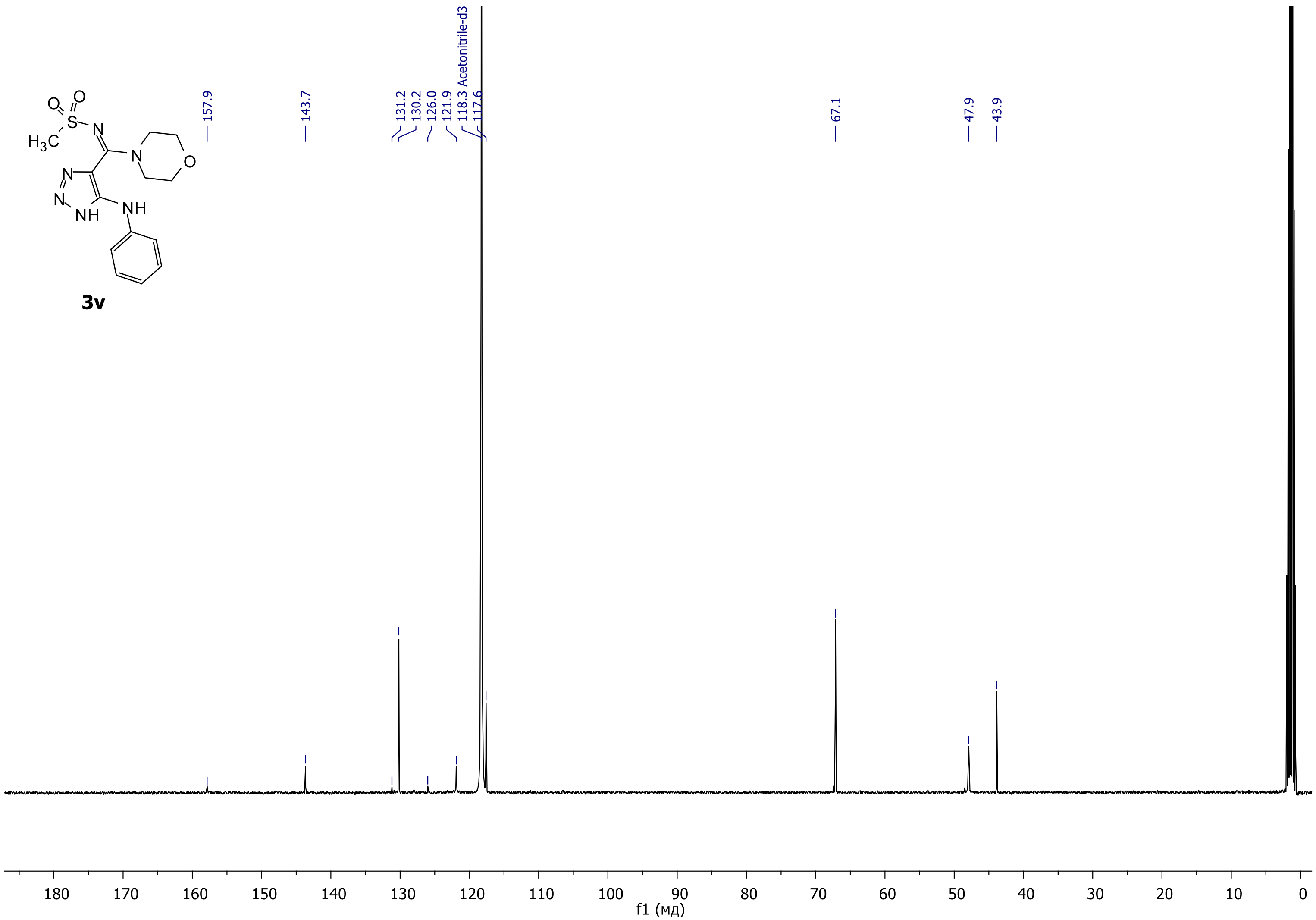

The ^{13}C NMR (100 MHz, CD_3CN, 50 °C) spectrum of compound **3v**.

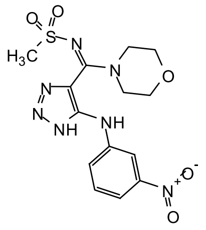

3w

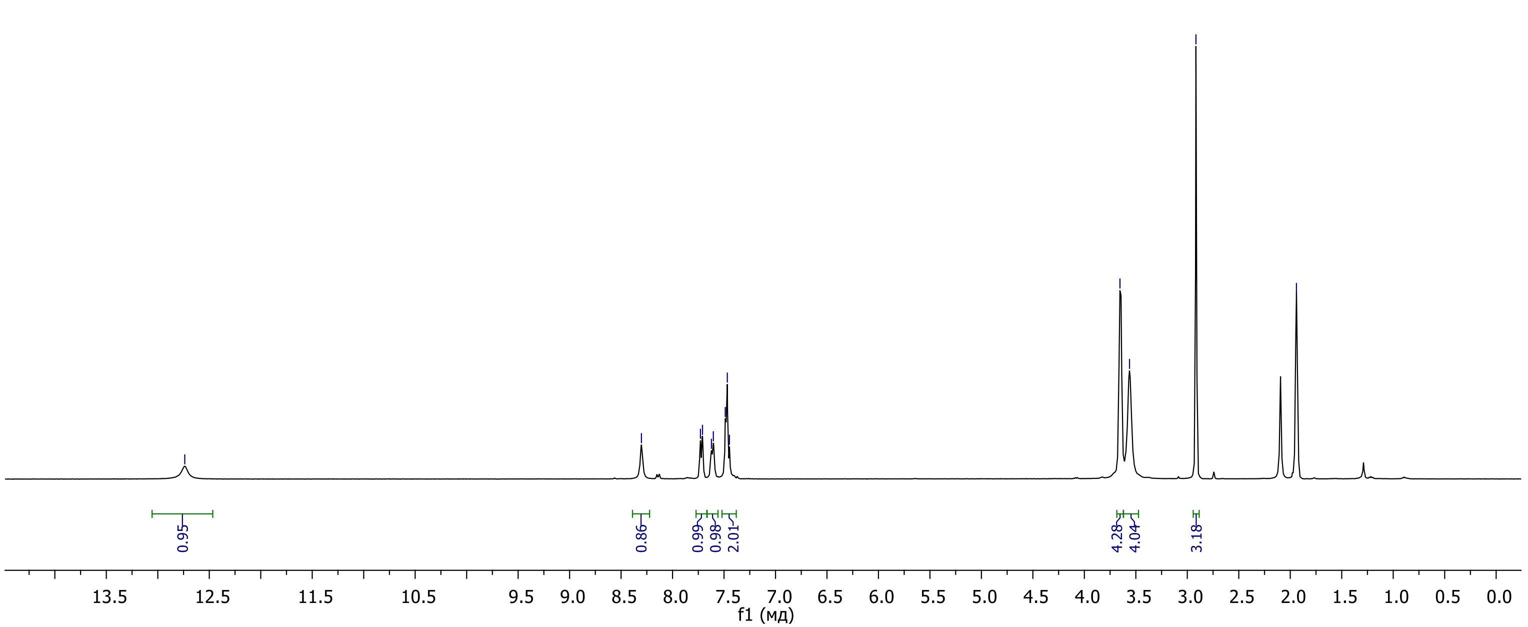

The ^{1}H NMR (400 MHz, CD_3CN) spectrum of compound **3w**.

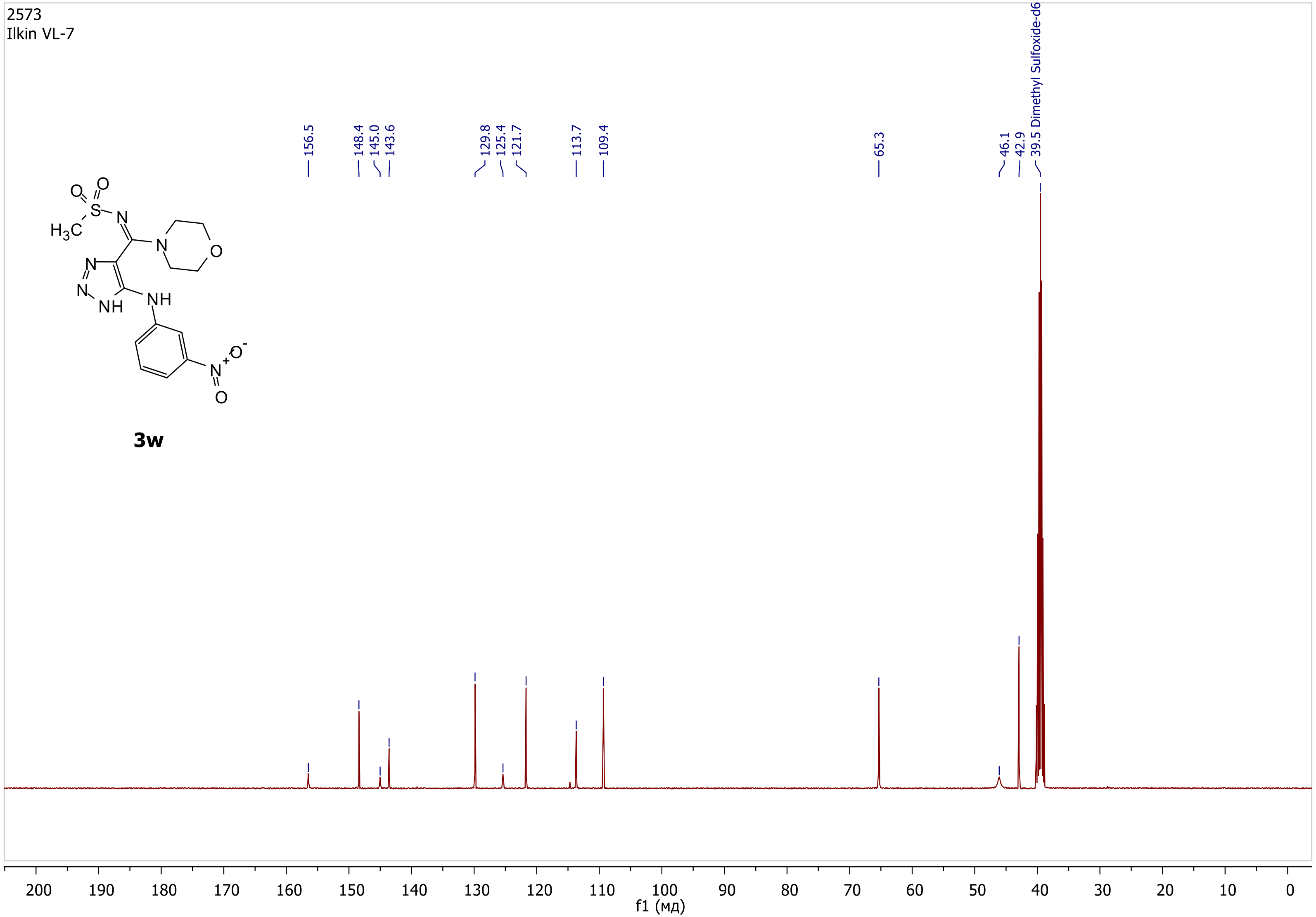

The ^{13}C NMR (100 MHz, DMSO-d_6, 50 °C) spectrum of compound **3w**.

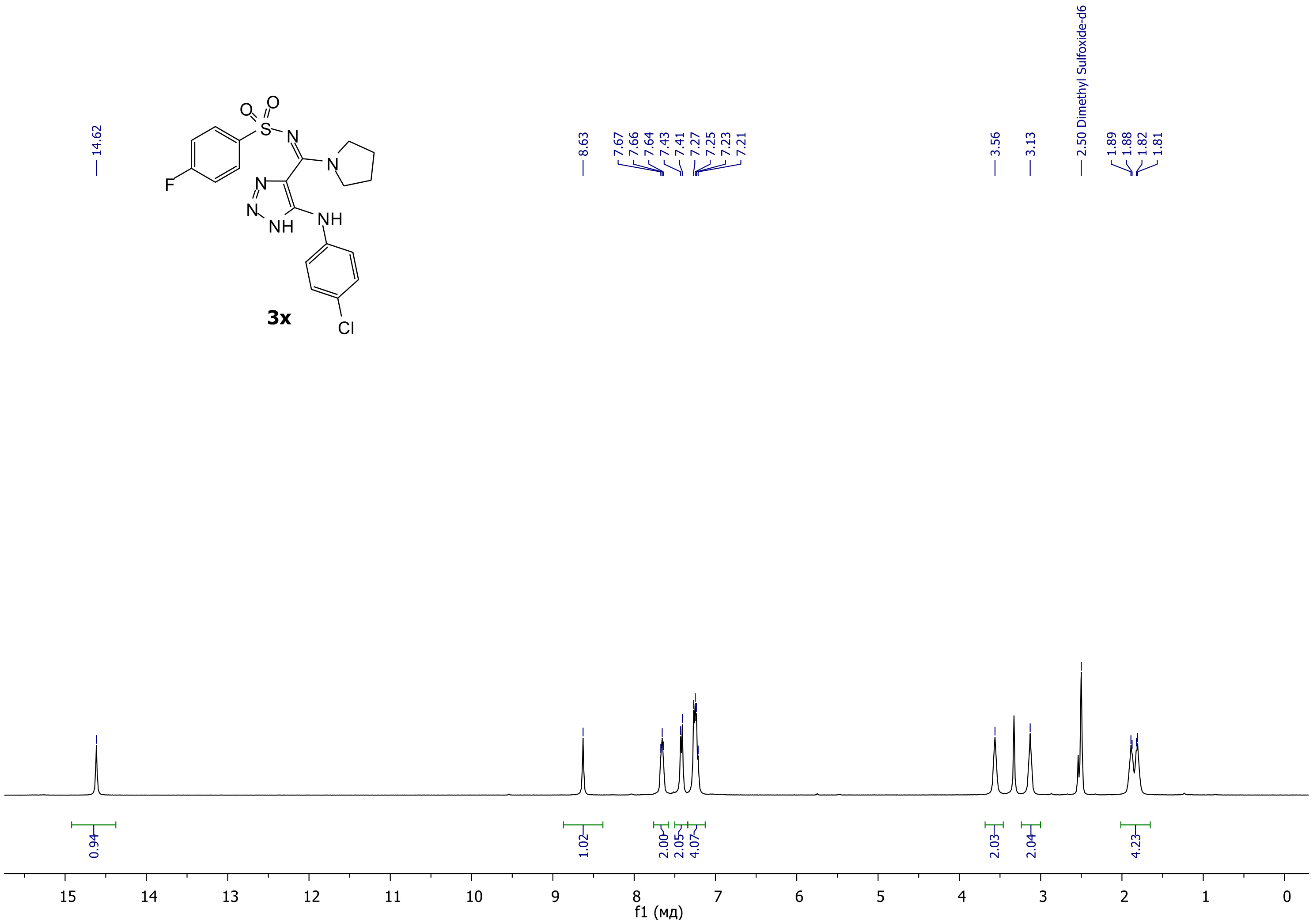

The ^{1}H NMR (400 MHz, DMSO-d_6) spectrum of compound **3x**.

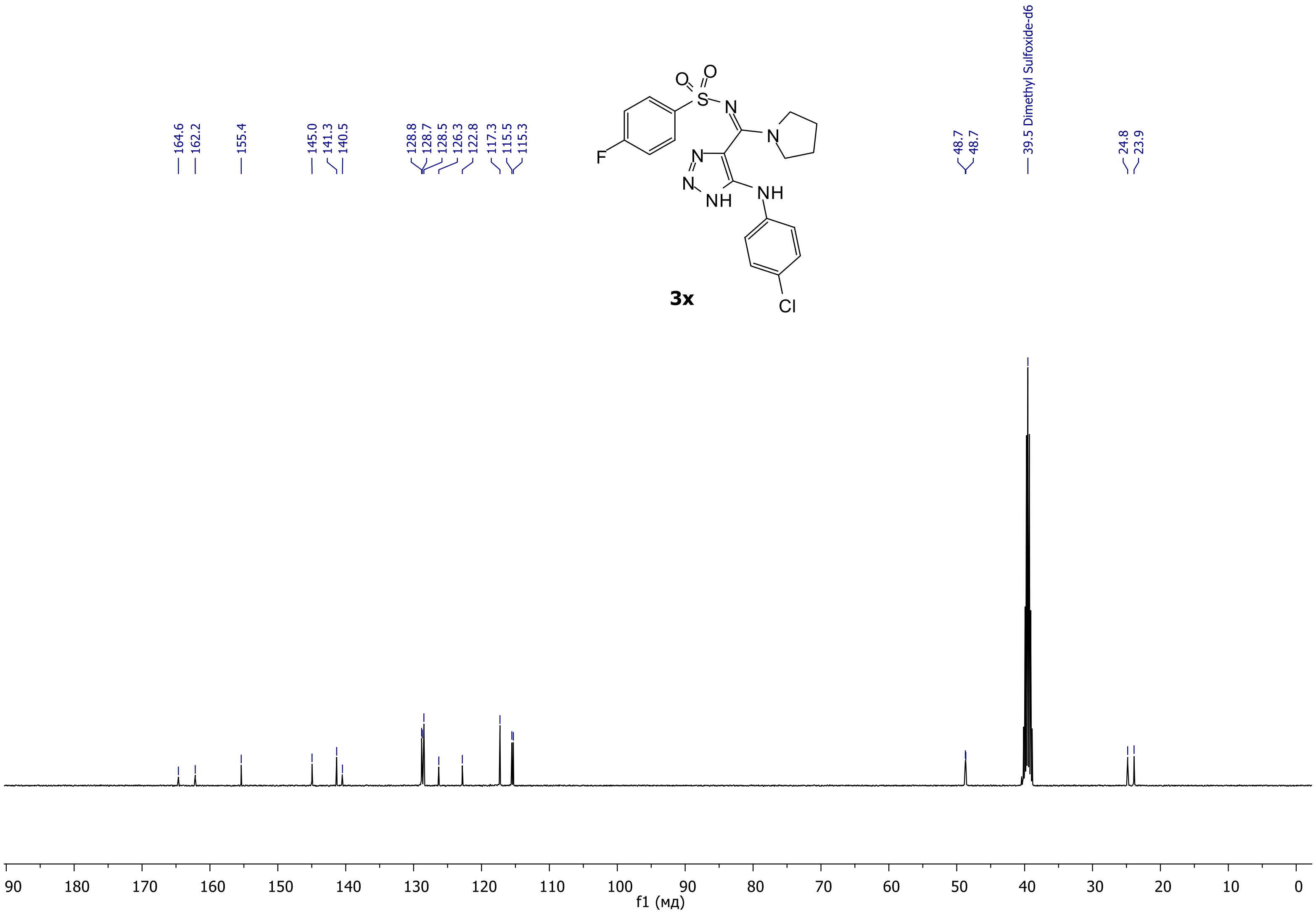

The ^{13}C NMR (100 MHz, DMSO-d_6) spectrum of compound **3x**.

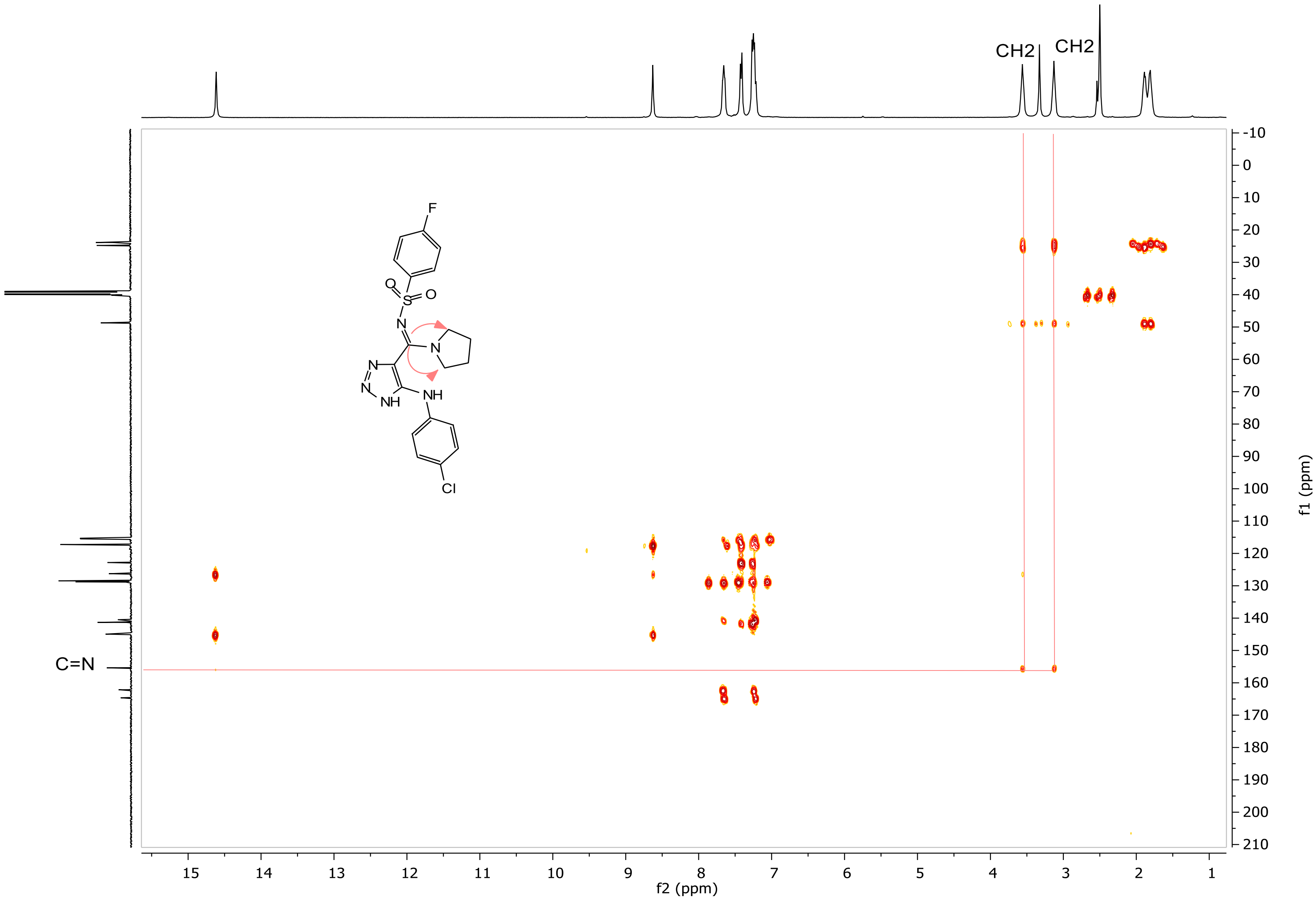

The HMBC NMR spectrum of compound **3x**.

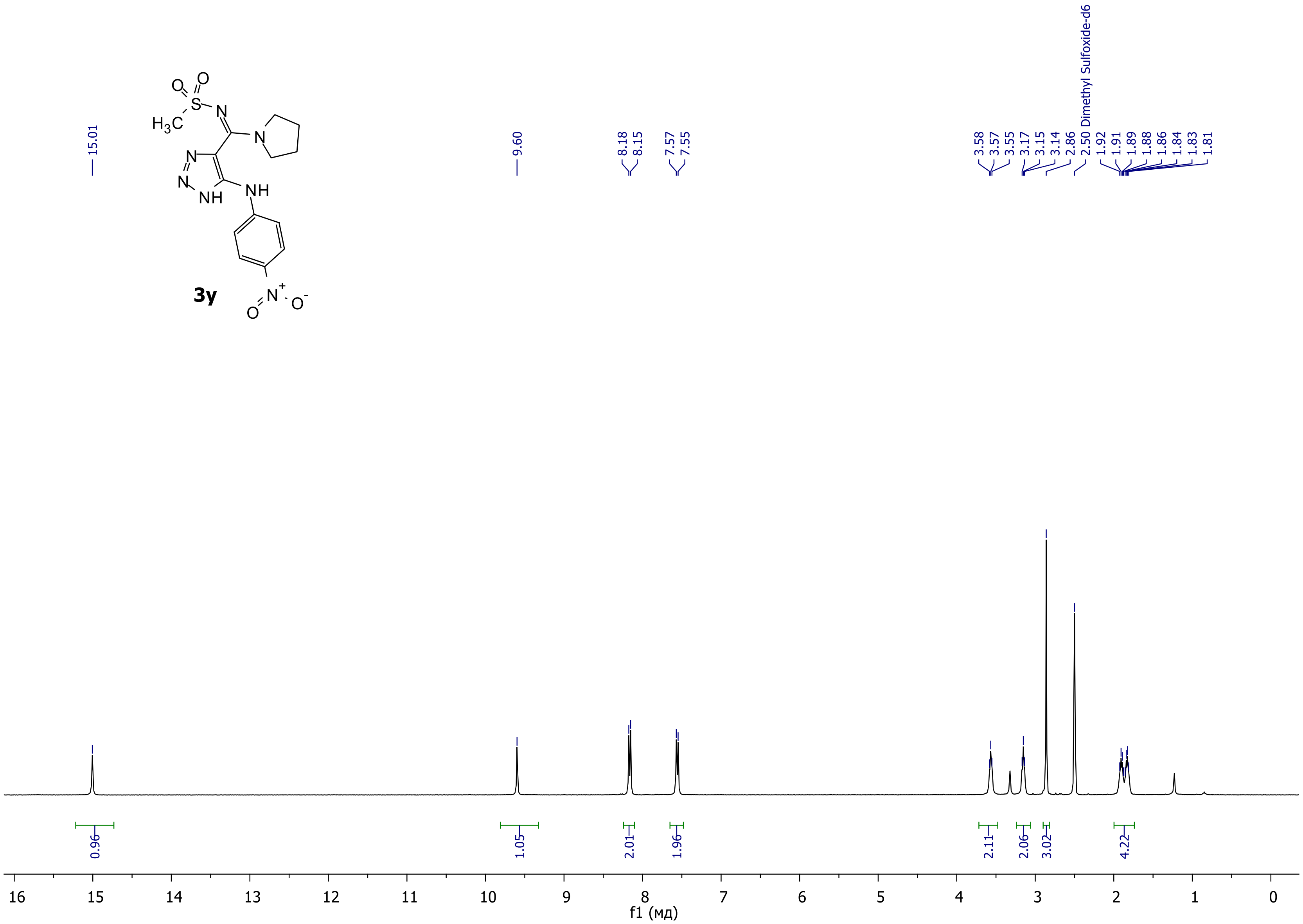

The ^{1}H NMR (400 MHz, DMSO-d_6) spectrum of compound **3y**.

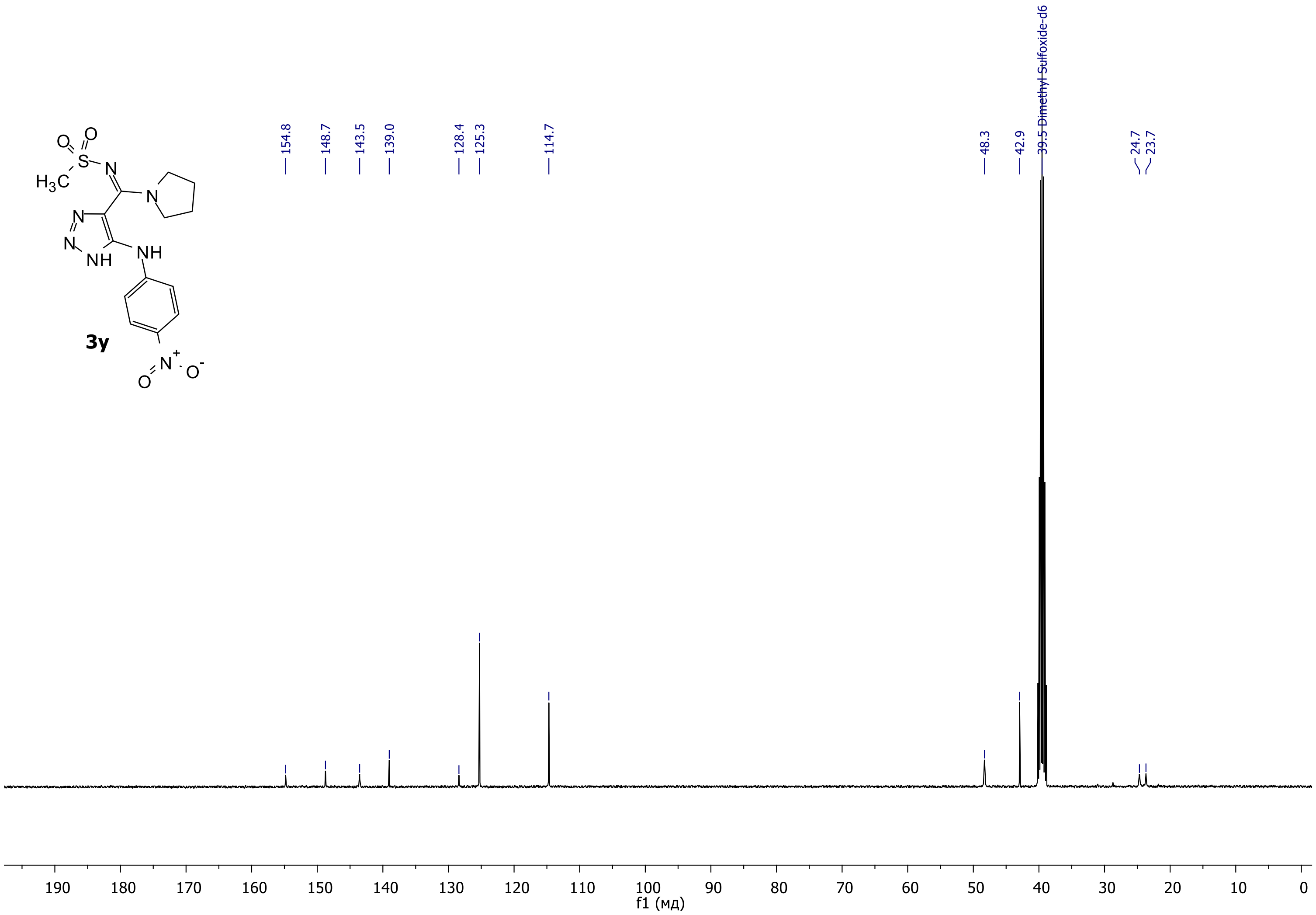

The ^{13}C NMR (100 MHz, DMSO-d_6) spectrum of compound **3y**.

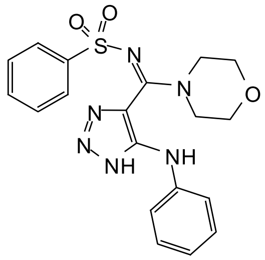

3z

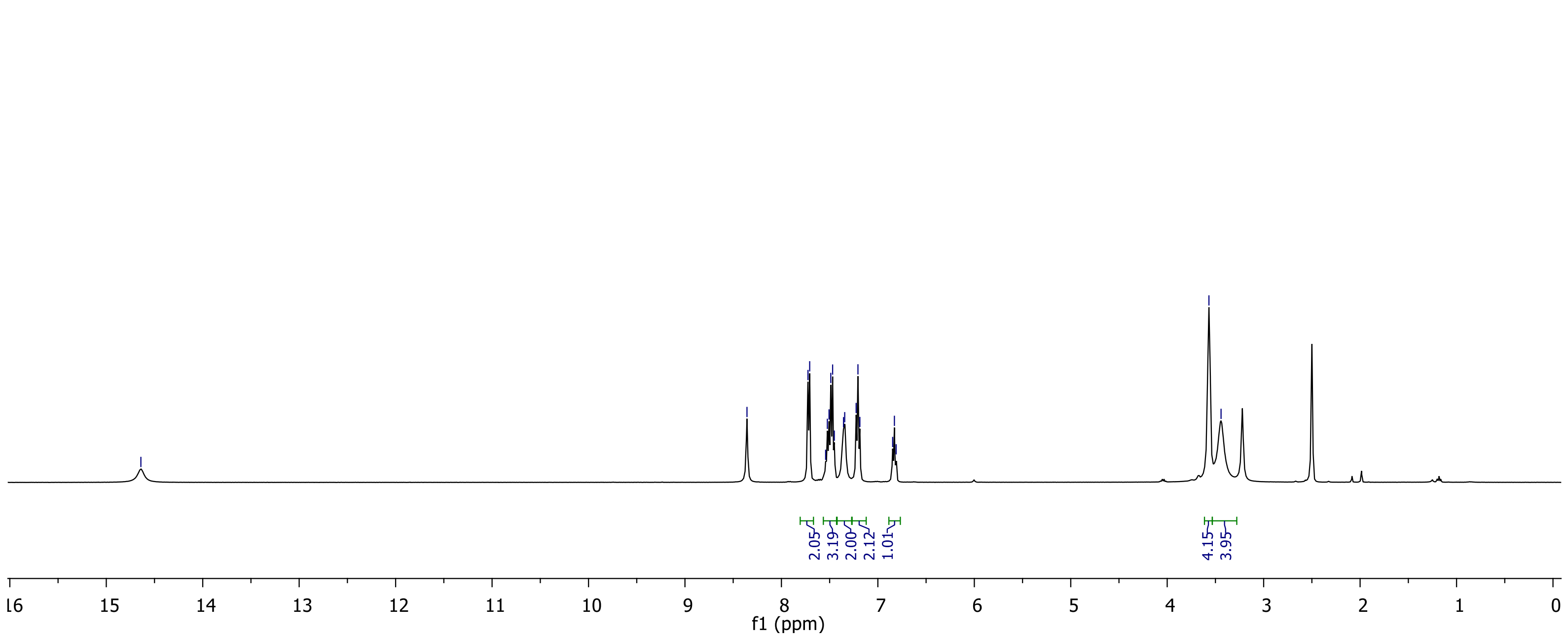

The ^{1}H NMR (400 MHz, DMSO-d_6) spectrum of compound **3z**.

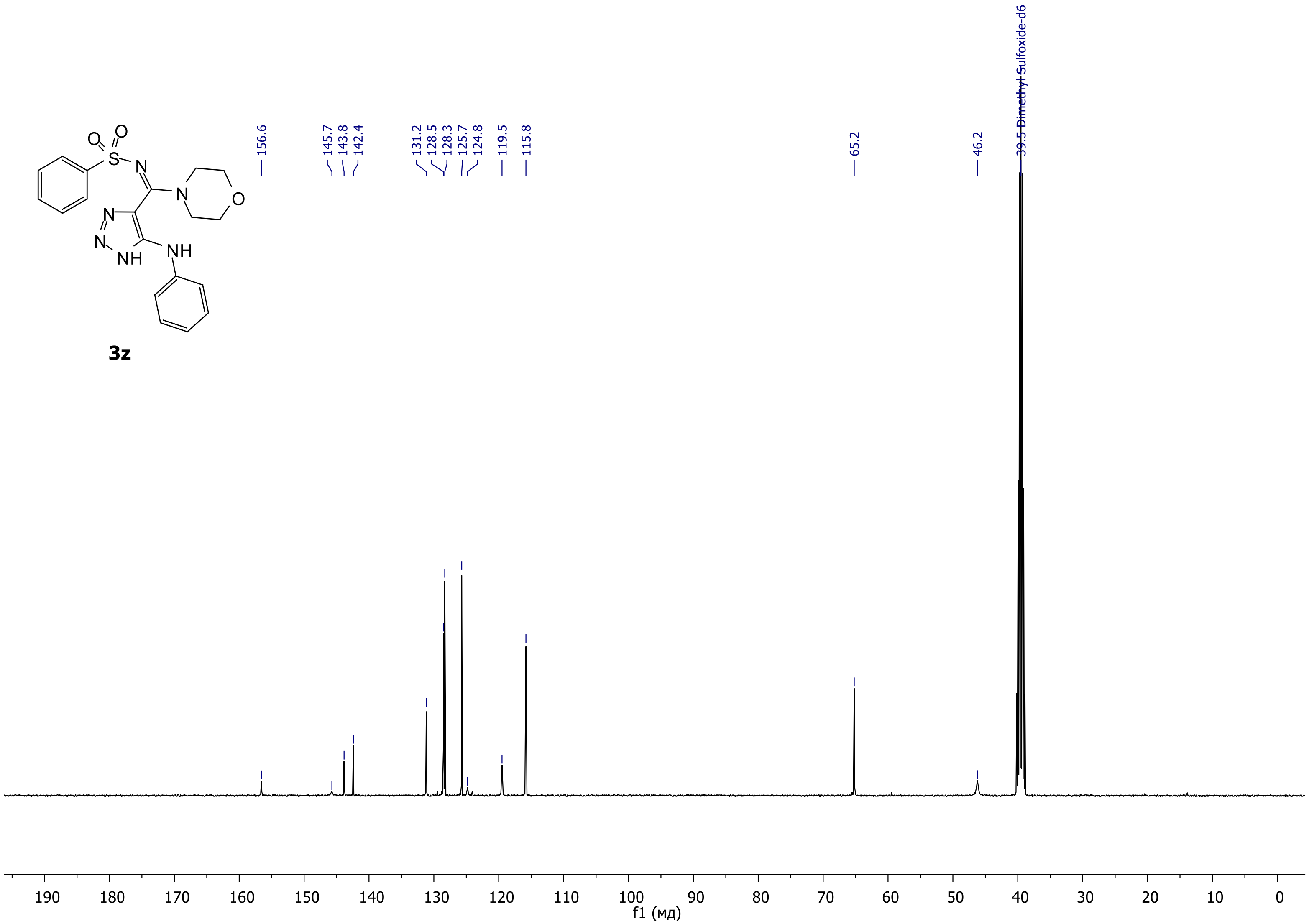

The ^{13}C NMR (100 MHz, DMSO-d_6, 50 °C) spectrum of compound **3z**.

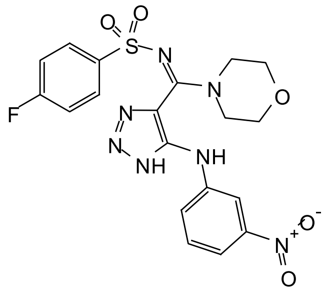

3aa

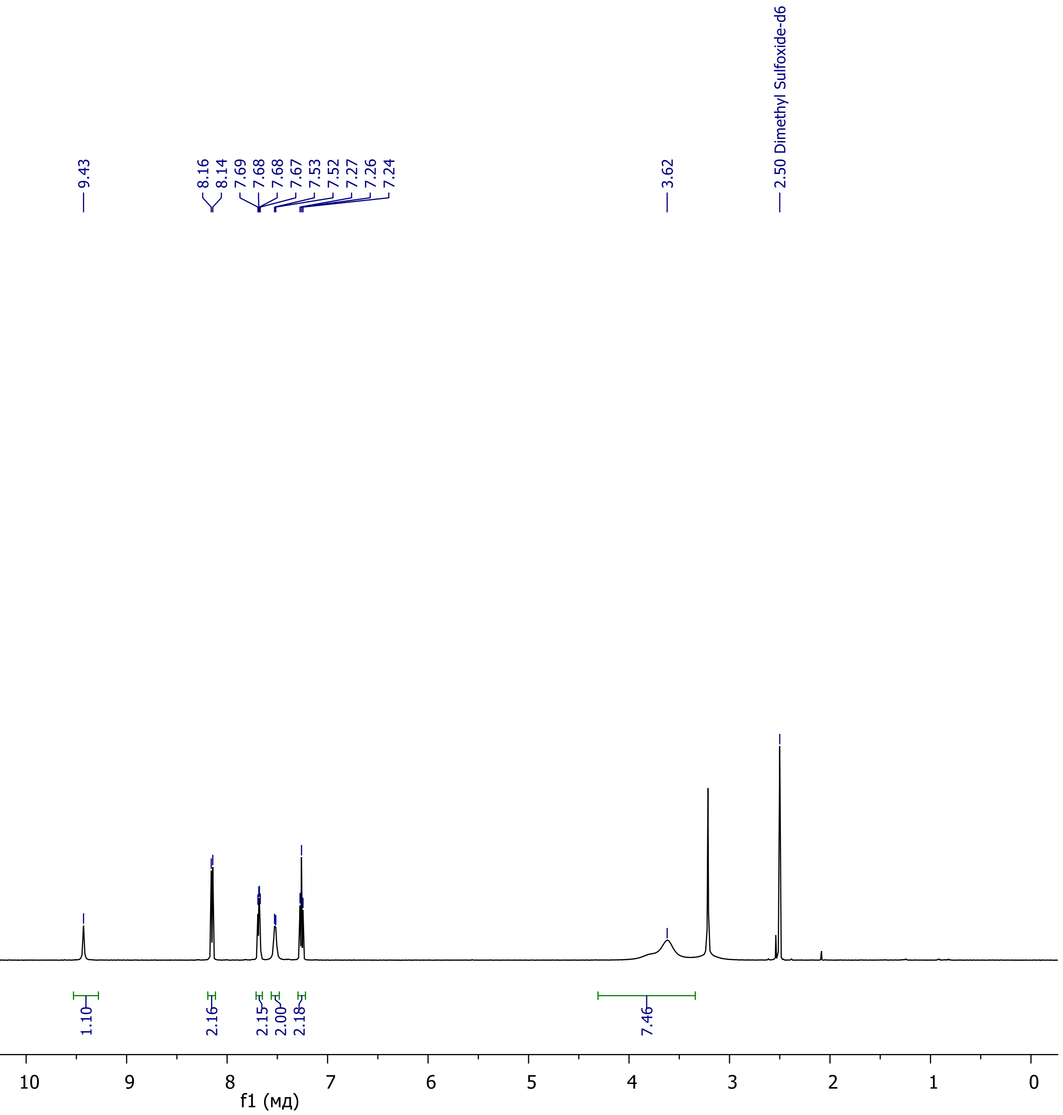

The ^{1}H NMR (400 MHz, DMSO-d_6) spectrum of compound **3aa**.

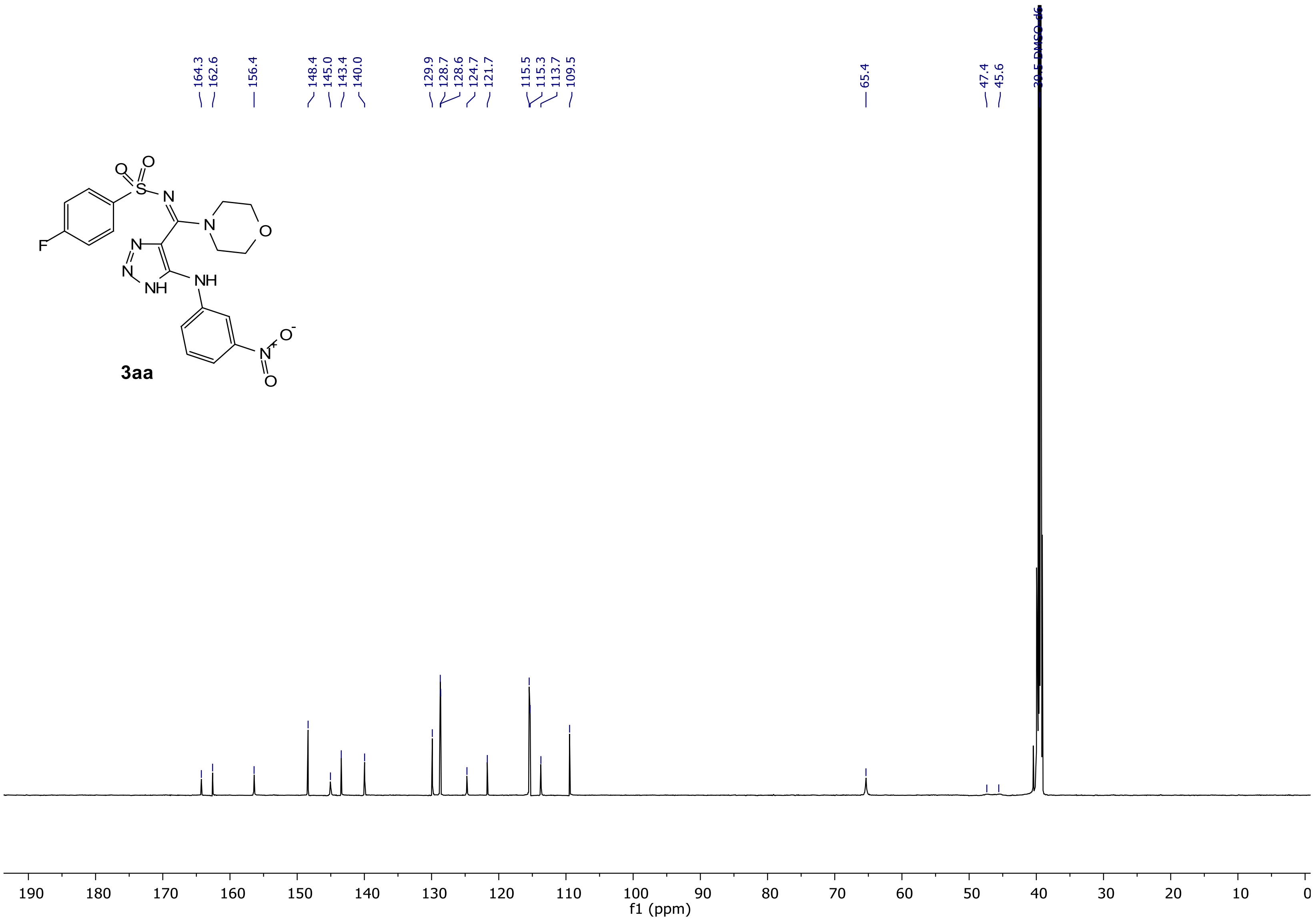

The ^{13}C NMR (150 MHz, DMSO-d_6) spectrum of compound **3aa**.

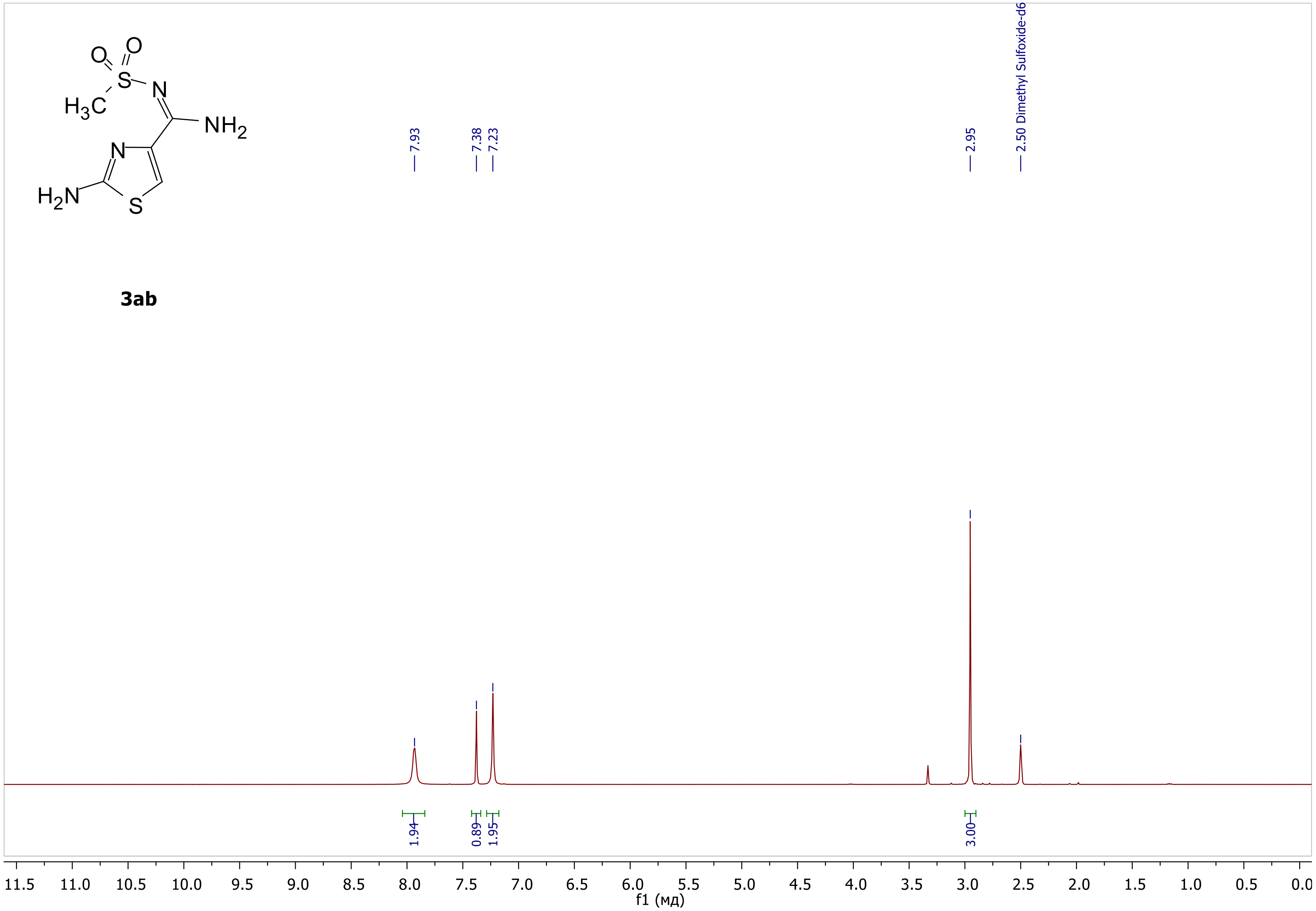

The ^{1}H NMR (400 MHz, DMSO-d_6) spectrum of compound **3ab**.

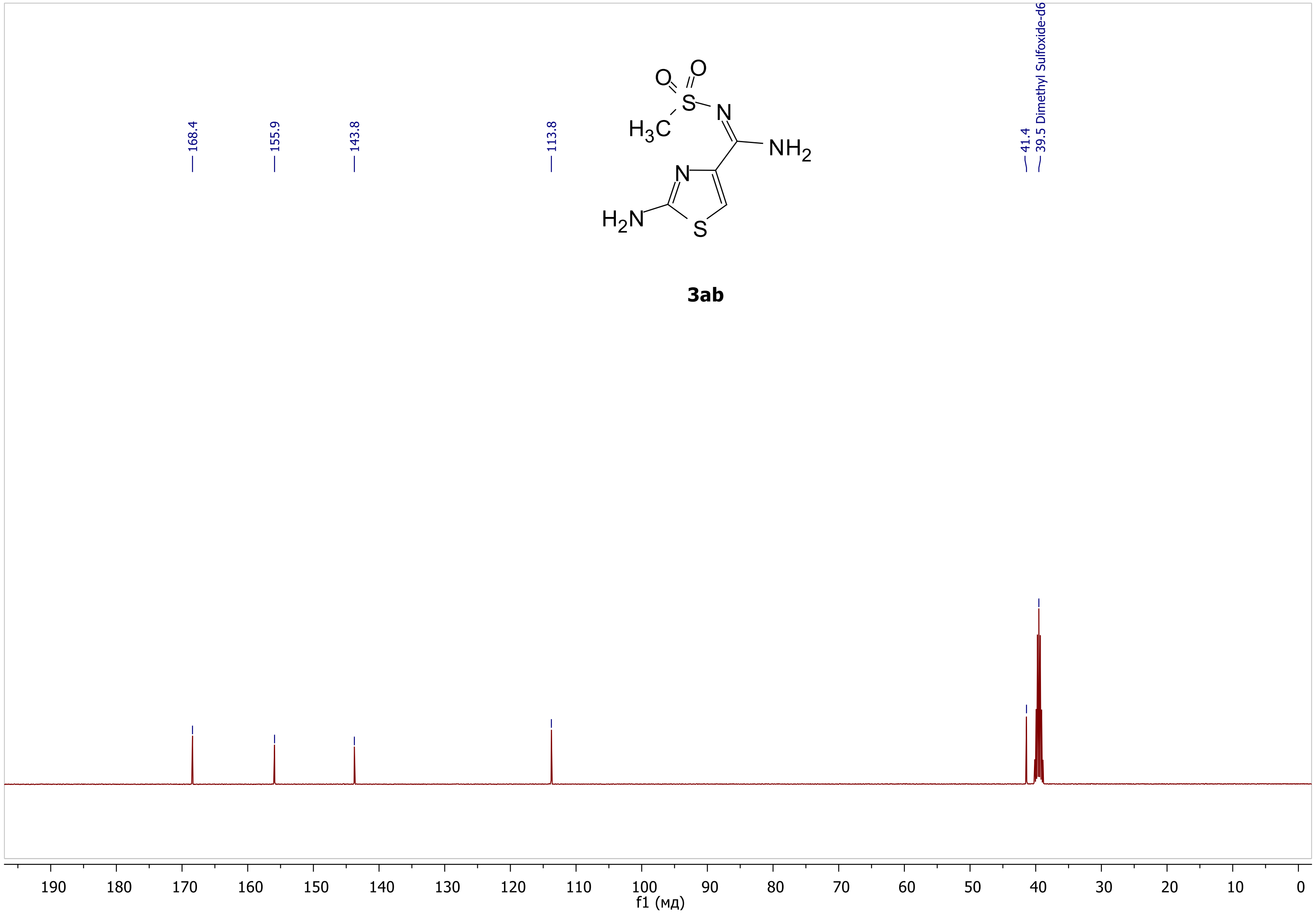

The ^{13}C NMR (100 MHz, DMSO-d_6) spectrum of compound **3ab**.

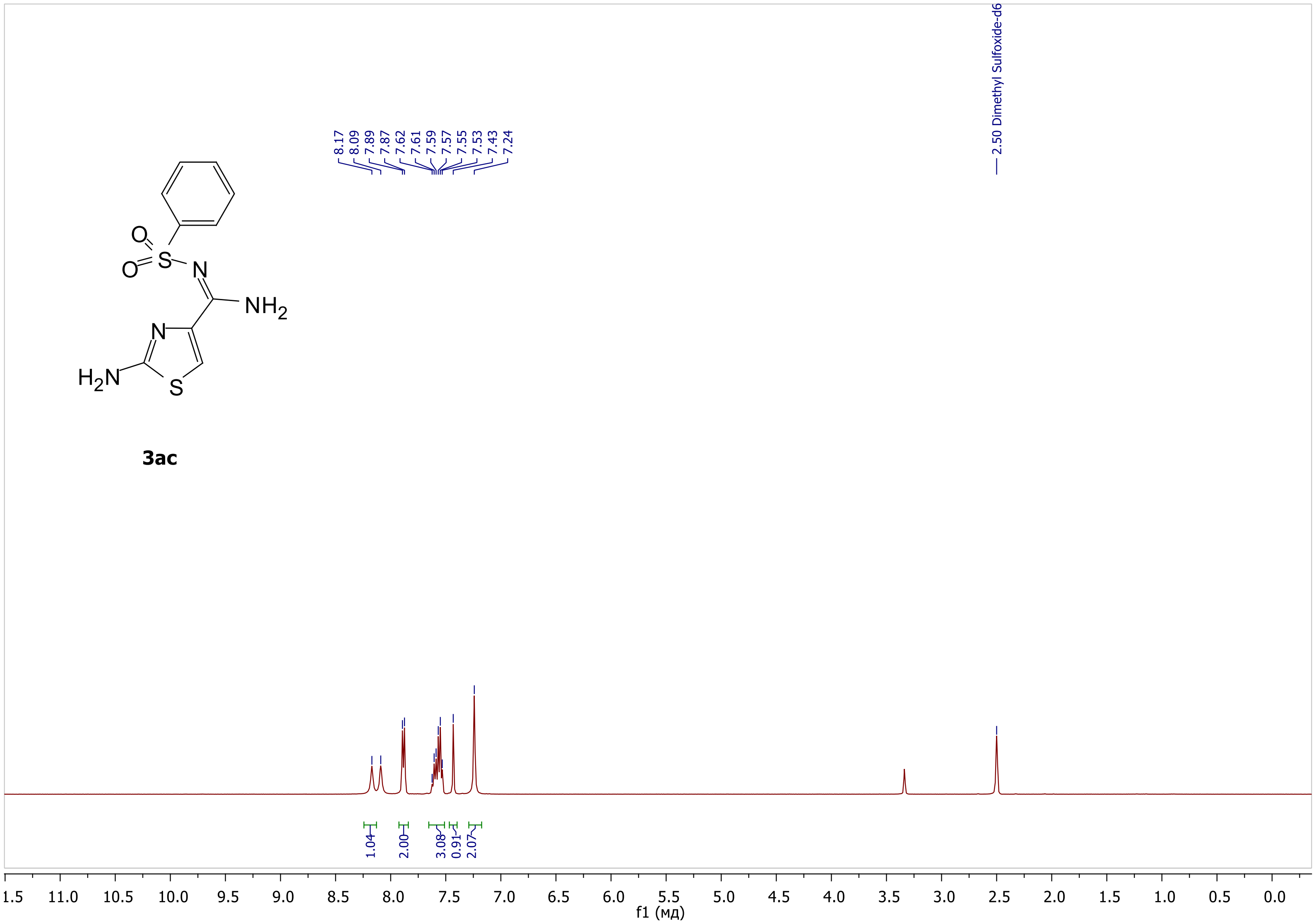

The ^{1}H NMR (400 MHz, DMSO-d_6) spectrum of compound **3ac**.

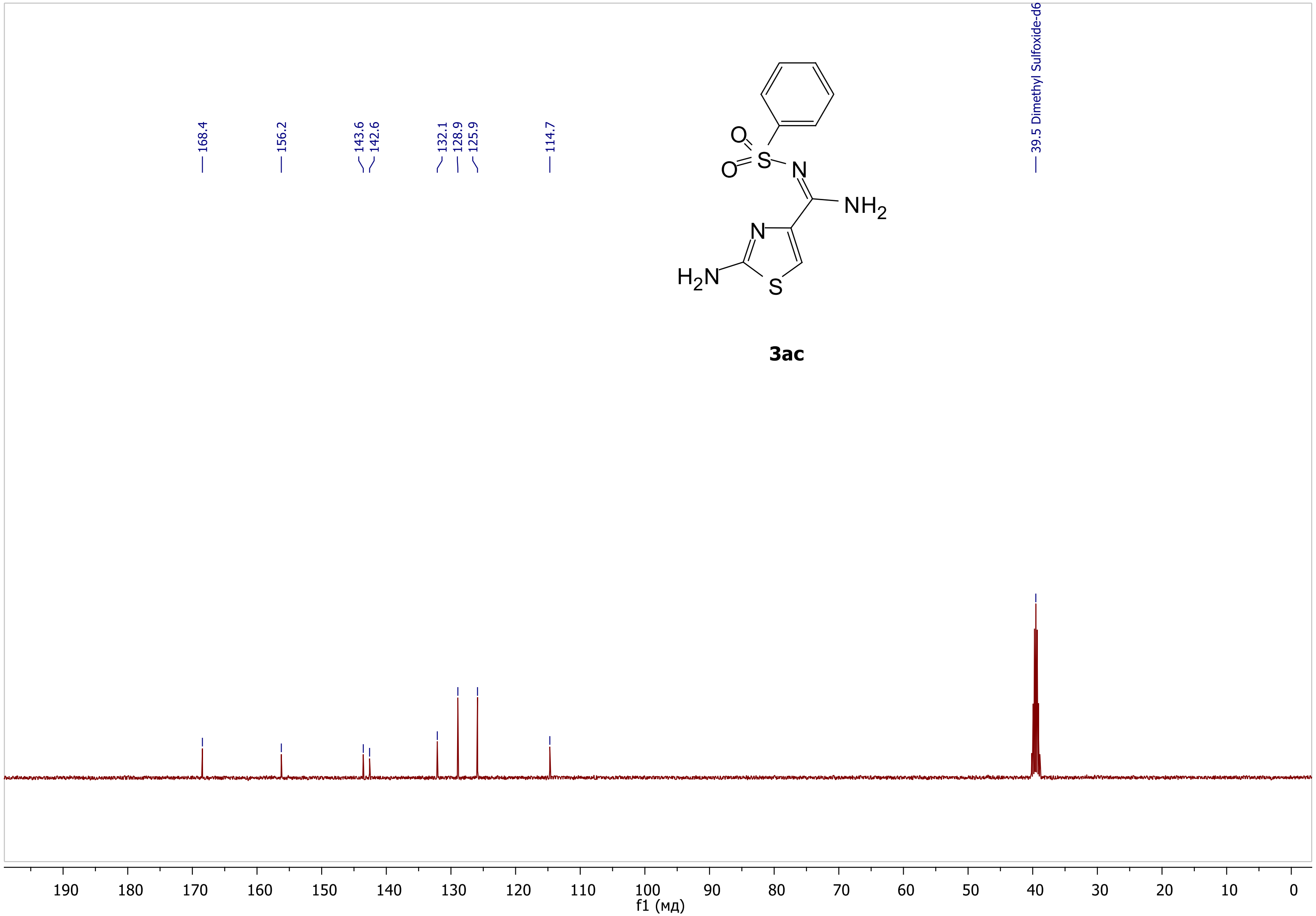

The ^{13}C NMR (100 MHz, DMSO-d_6) spectrum of compound **3ac**.

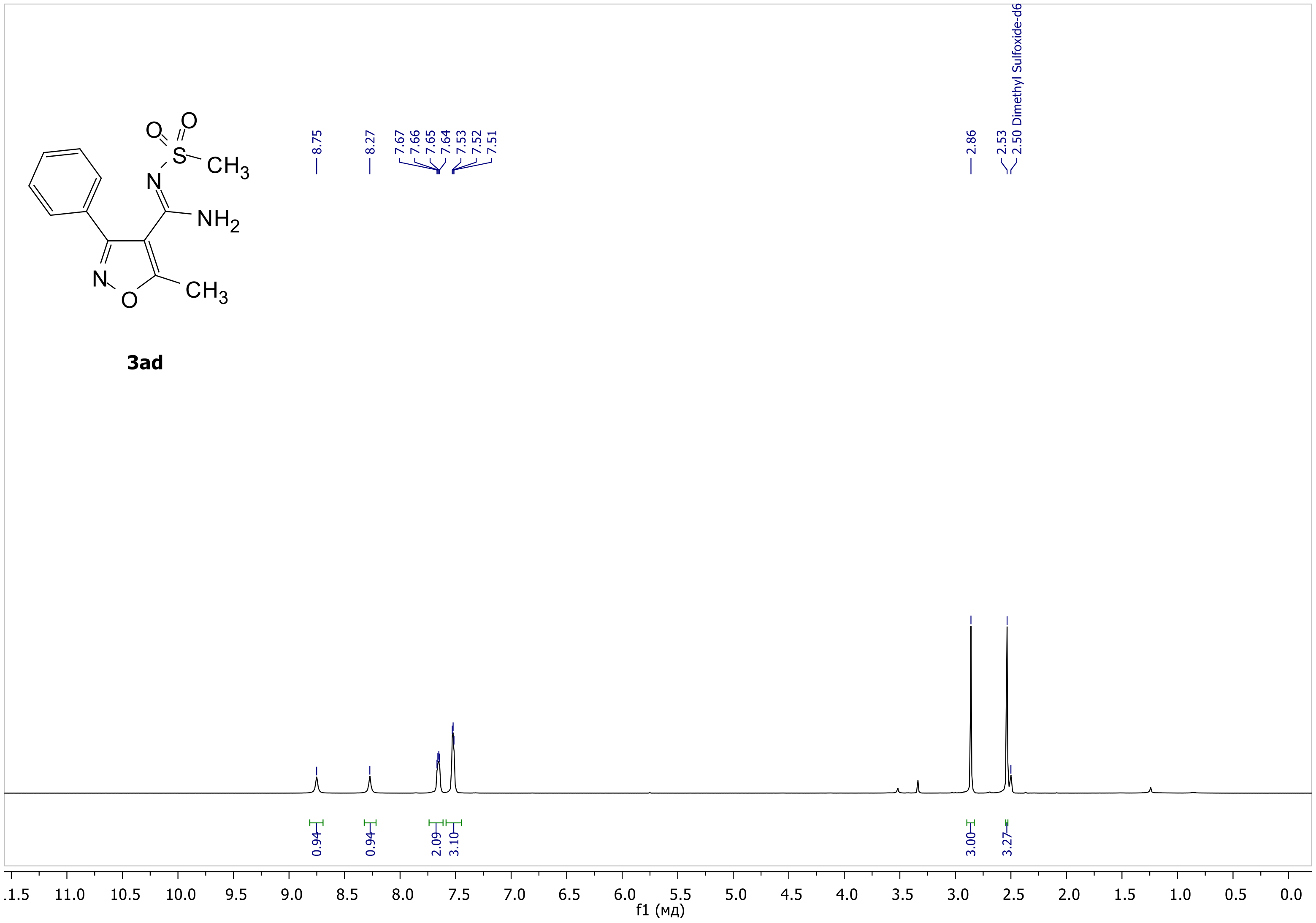

The ^{1}H NMR (400 MHz, DMSO-d_6) spectrum of compound **3ad**.

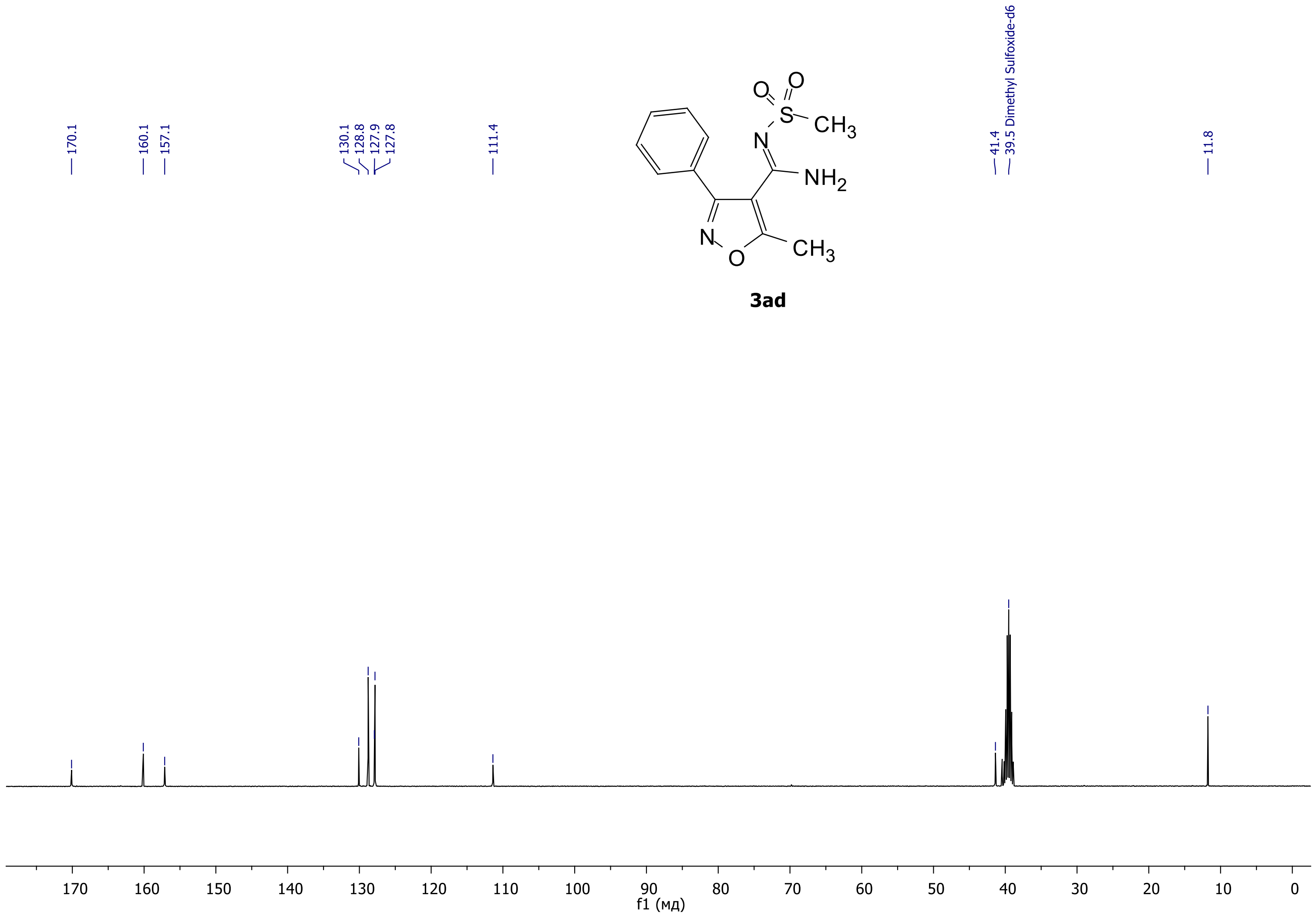

The ^{13}C NMR (100 MHz, DMSO-d_6) spectrum of compound **3ad**.

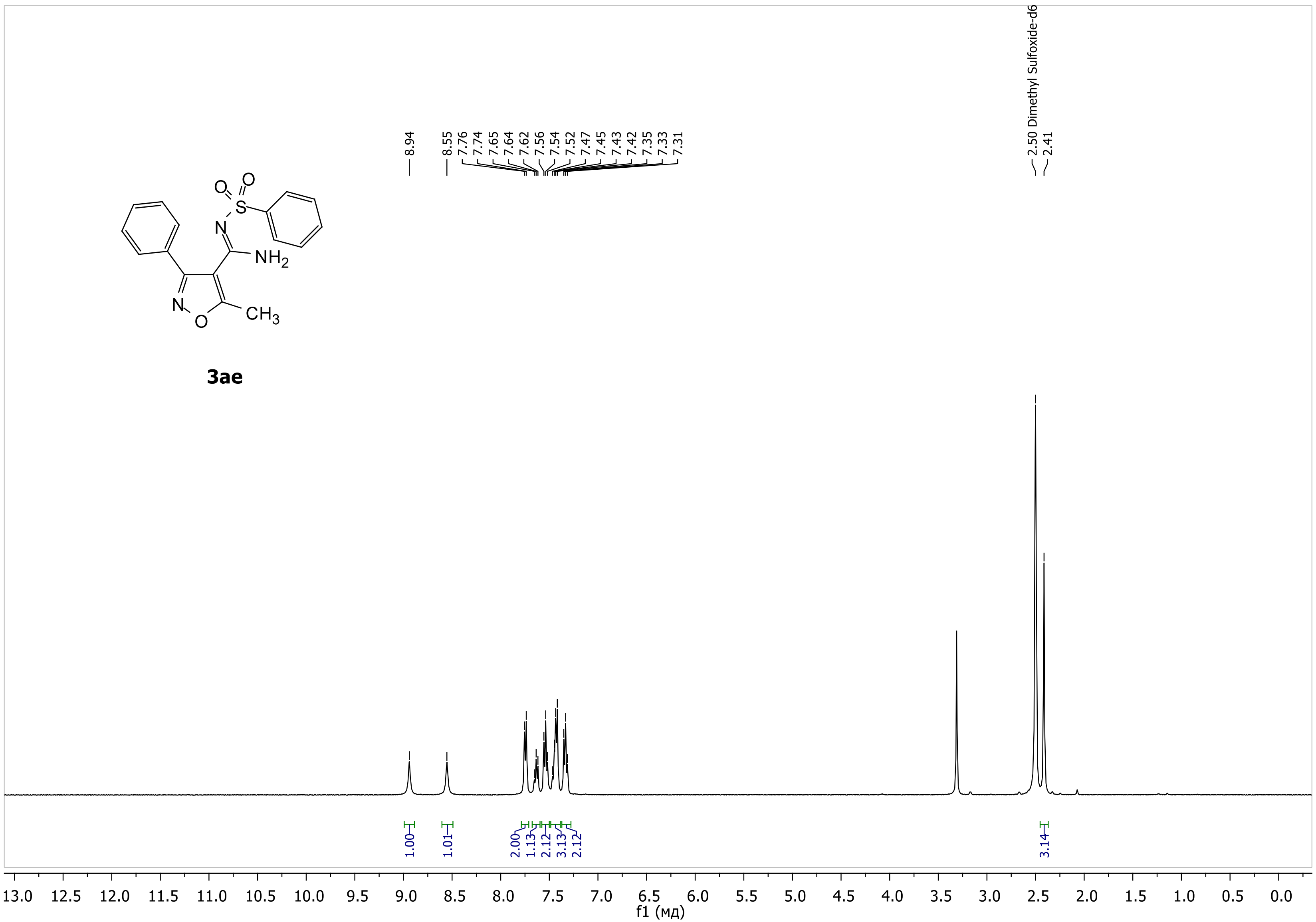

The ^{1}H NMR (400 MHz, DMSO-d_6) spectrum of compound **3ae**.

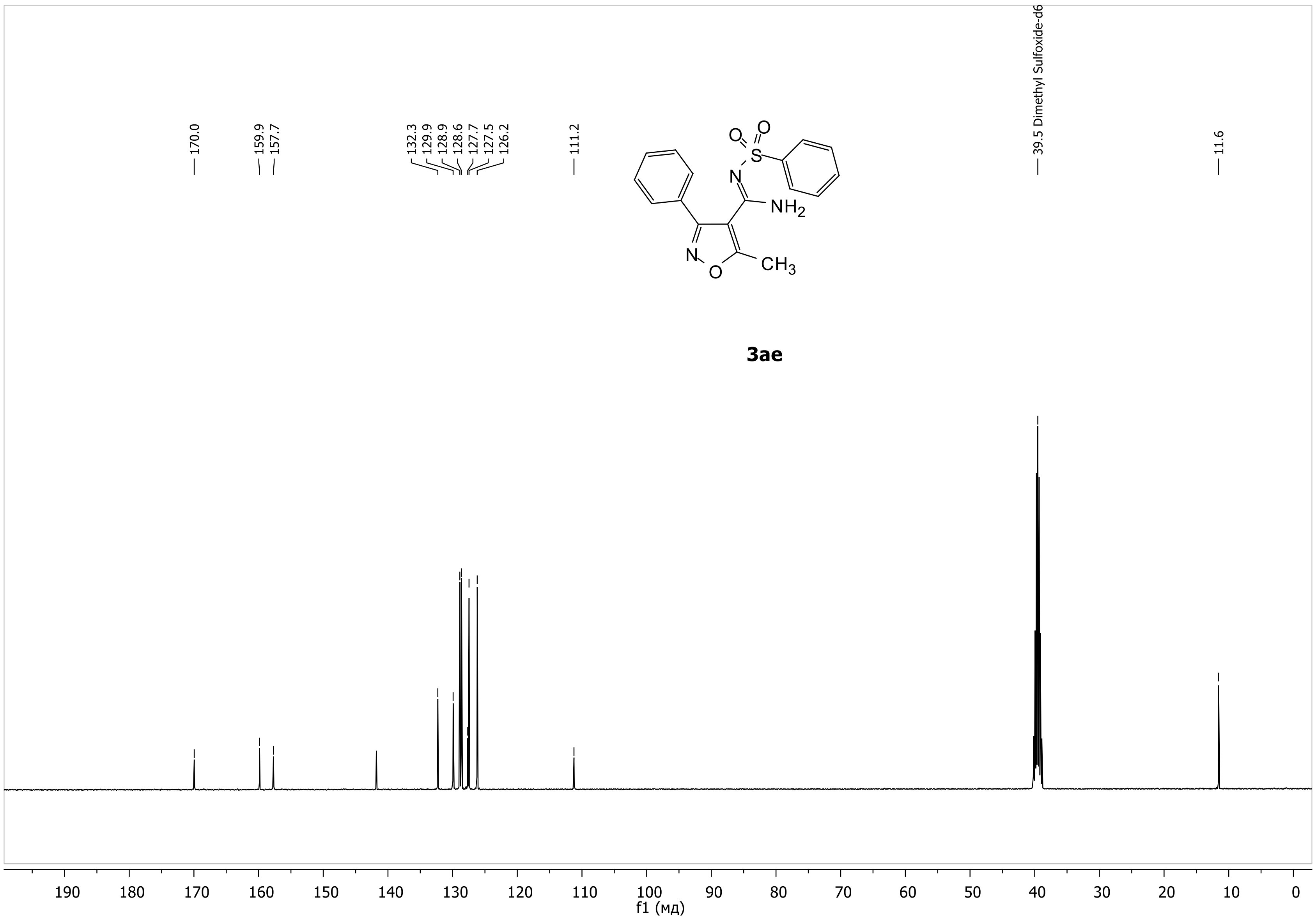

The ^{13}C NMR (100 MHz, DMSO-d_6) spectrum of compound **3ae**.

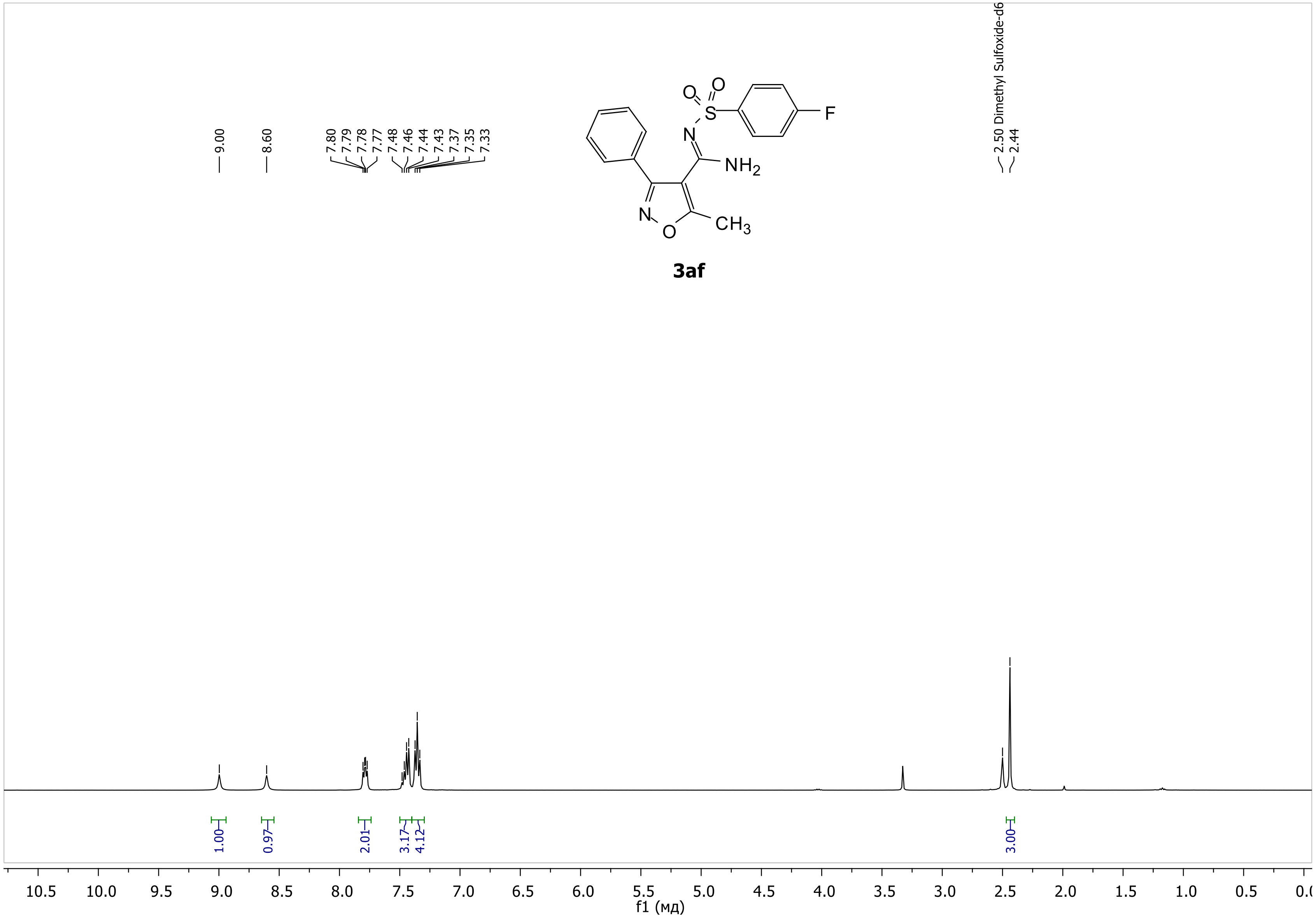

The ^{1}H NMR (400 MHz, DMSO-d_6) spectrum of compound **3af**.

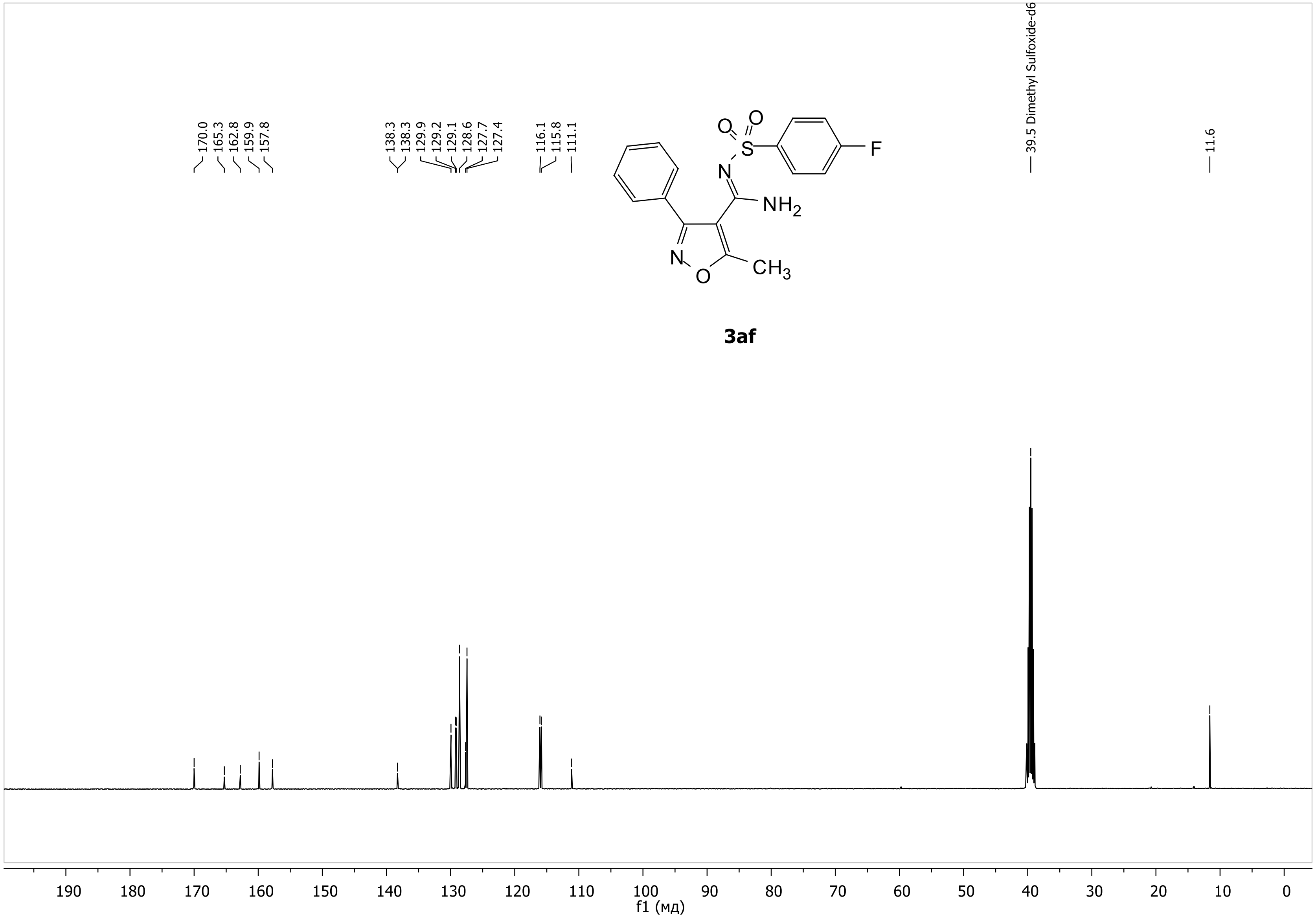

The ^{13}C NMR (100 MHz, DMSO-d_6) spectrum of compound **3af**.

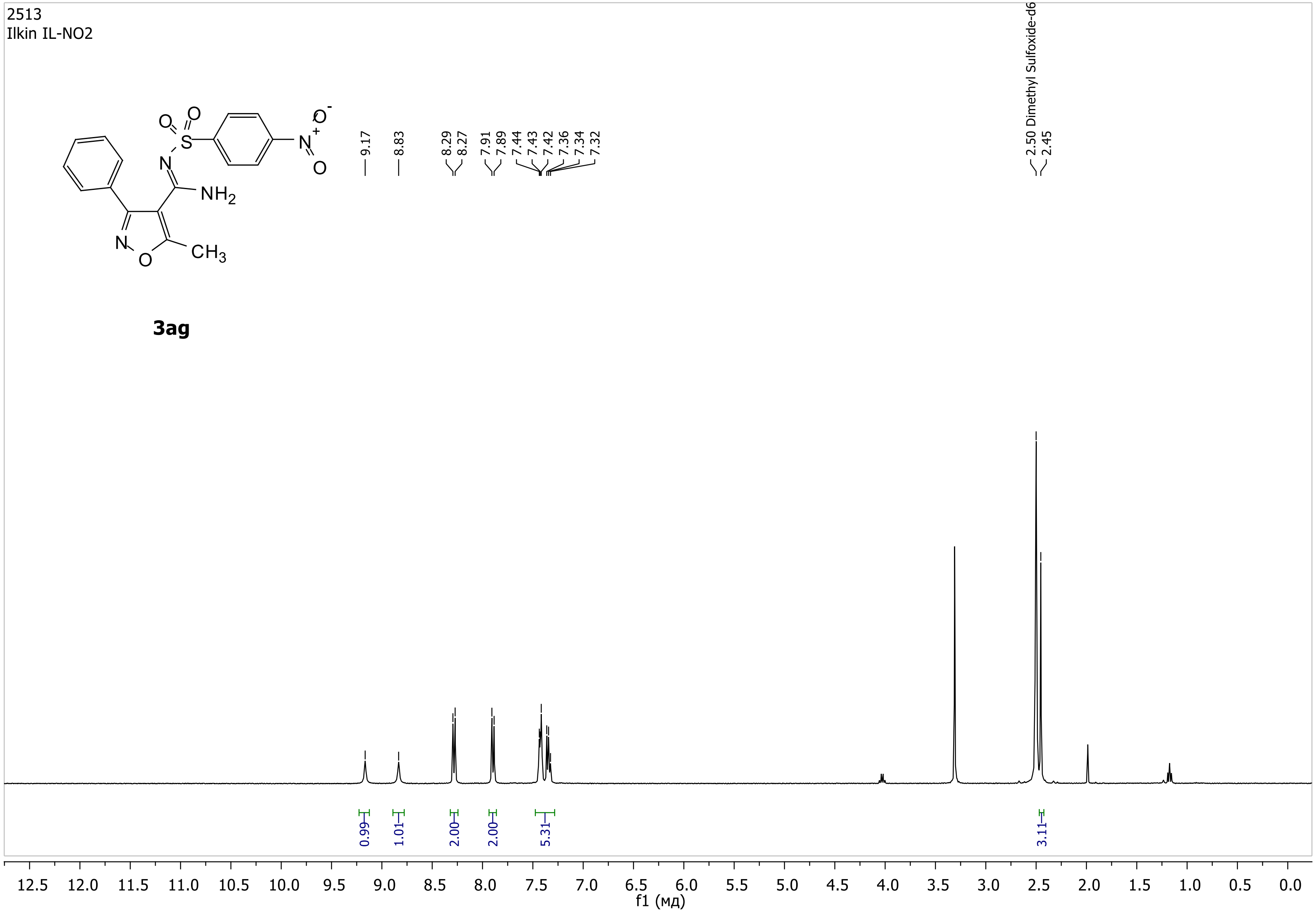

The ^{1}H NMR (400 MHz, DMSO-d_6) spectrum of compound **3ag**.

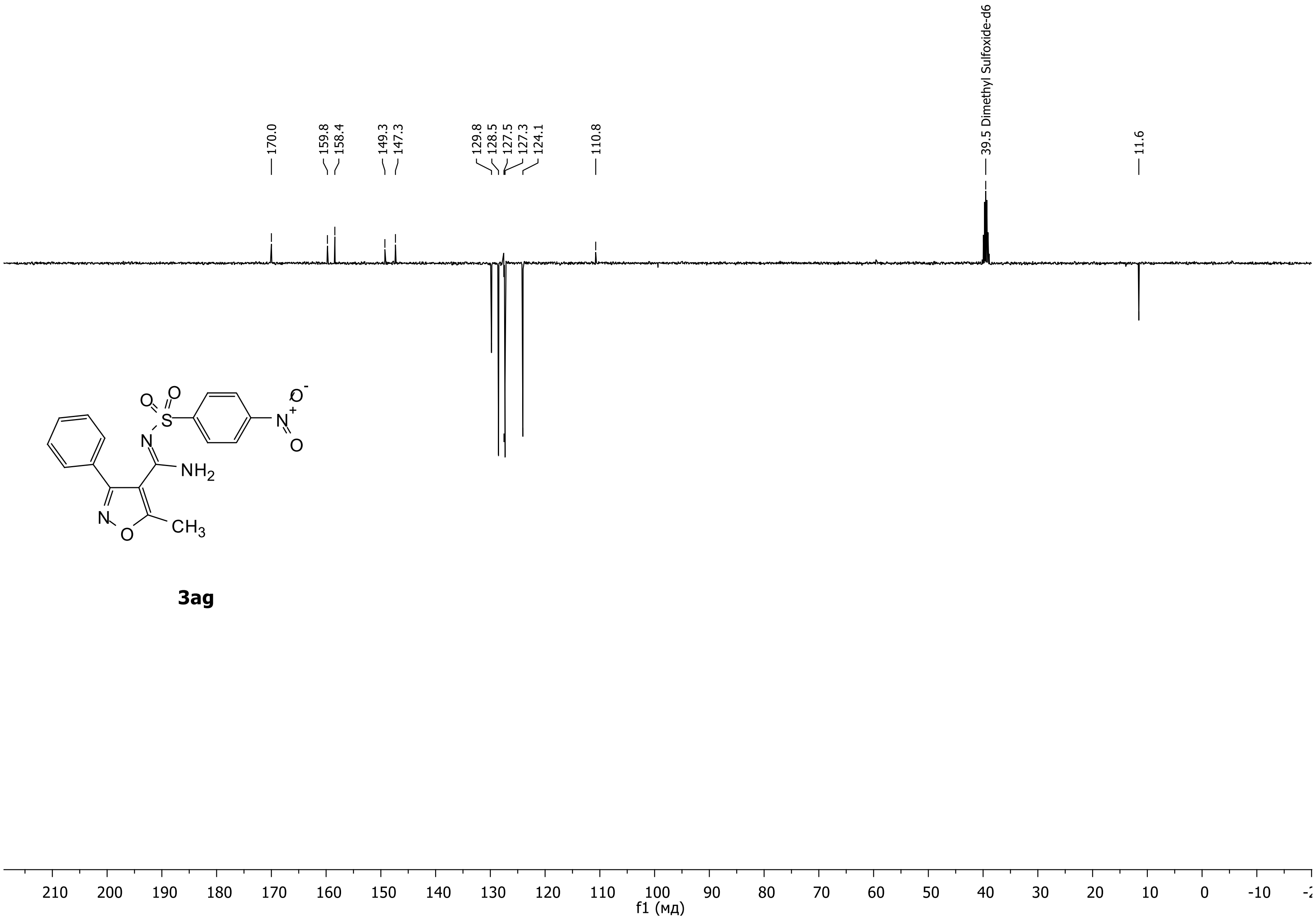

3ag

The ^{13}C NMR (100 MHz, DMSO-d_6) spectrum of compound **3ag**.

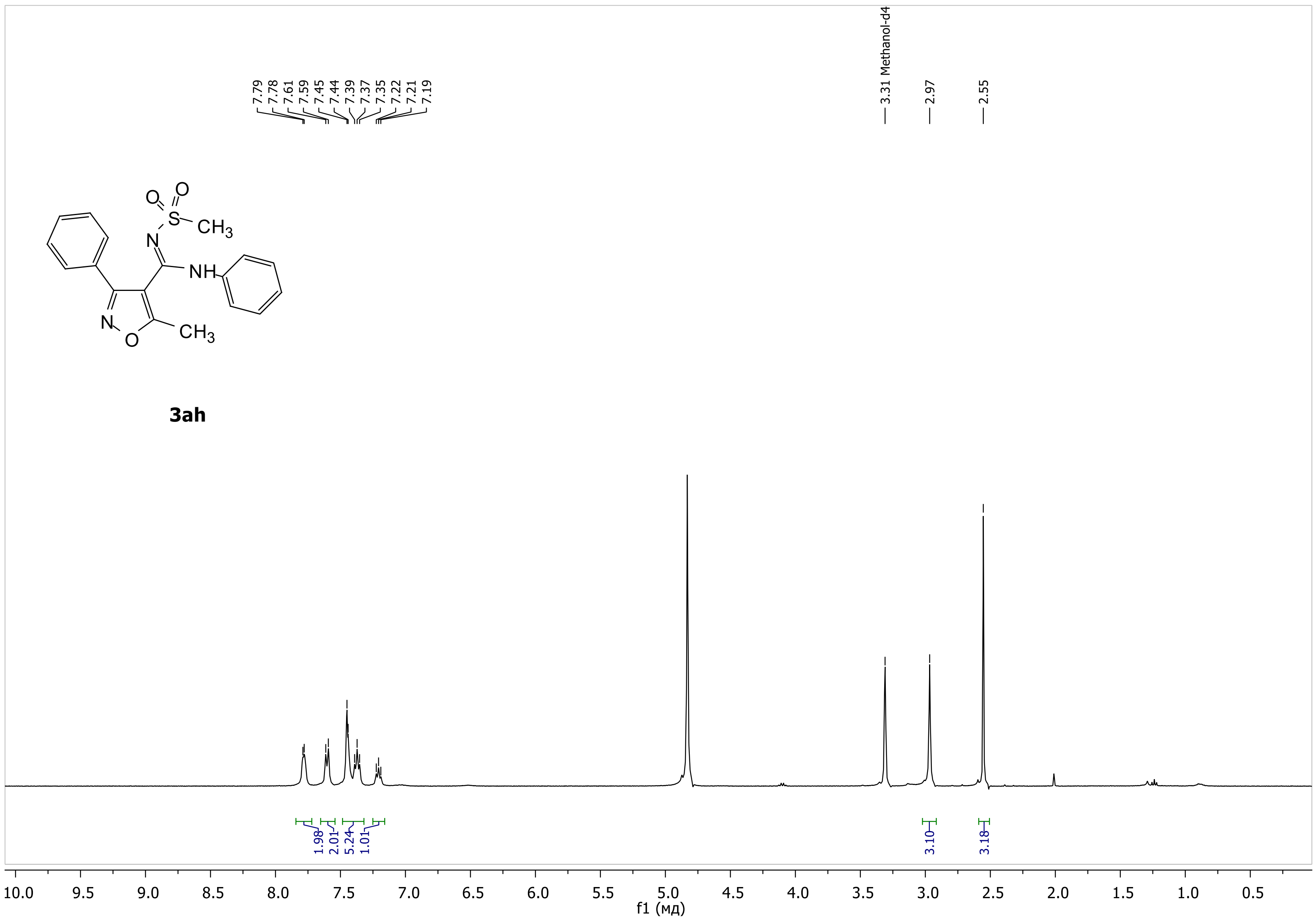

The ^{1}H NMR (400 MHz, CD_3OD) spectrum of compound **3ah**.

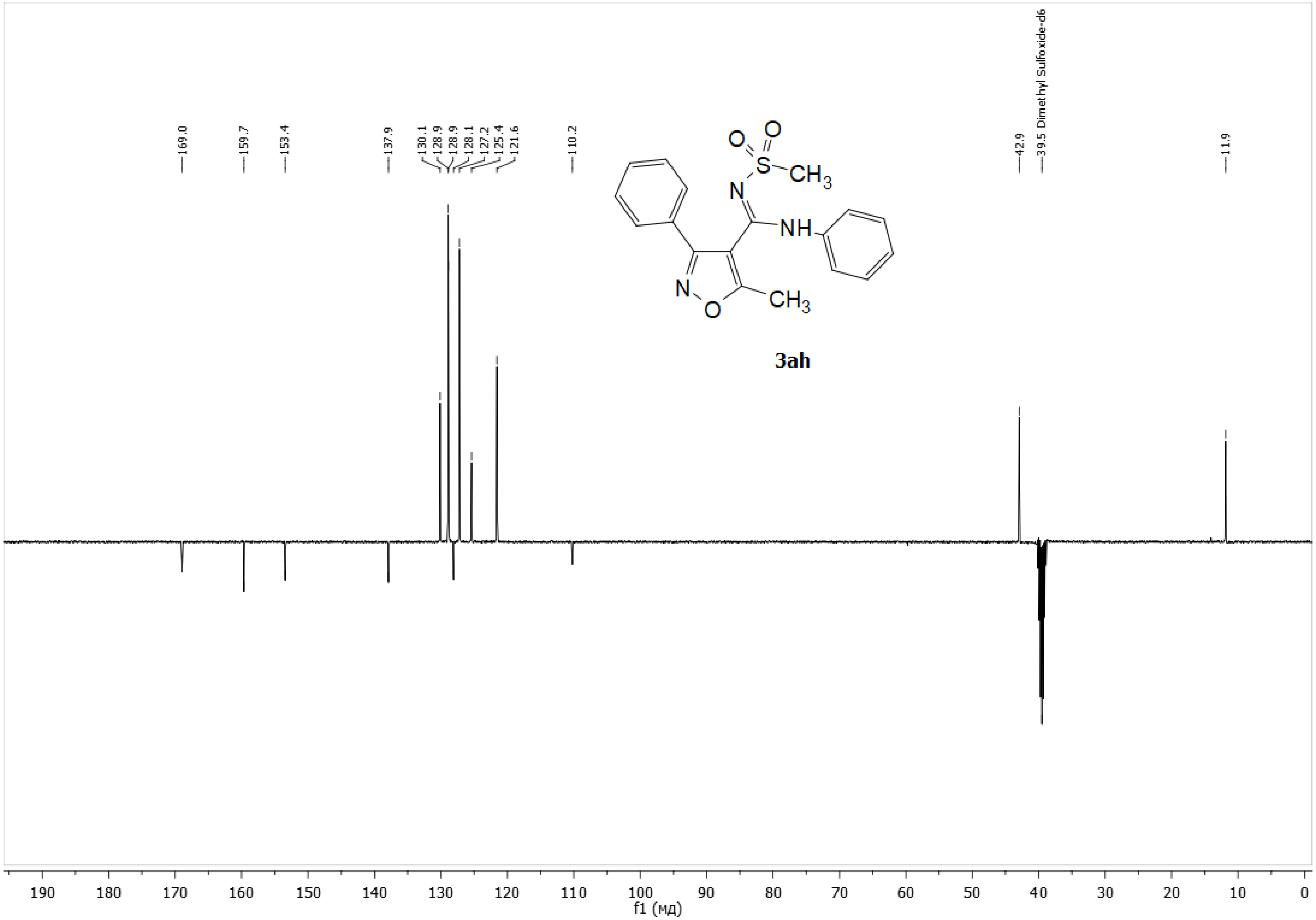

The ^{13}C NMR (100 MHz, DMSO-d_6) spectrum of compound **3ah**.

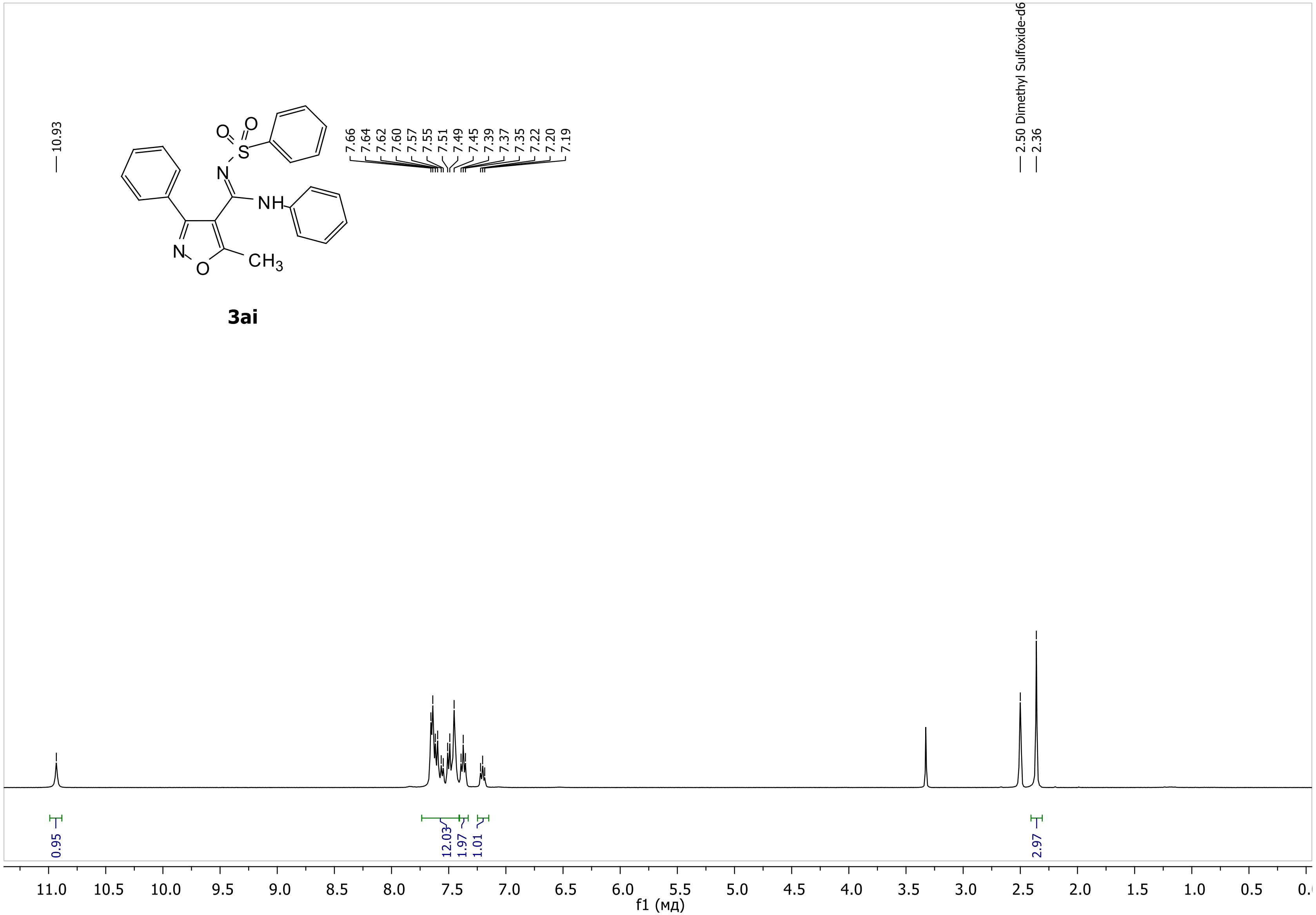

The ^{1}H NMR (400 MHz, DMSO-d_6) spectrum of compound **3ai**.

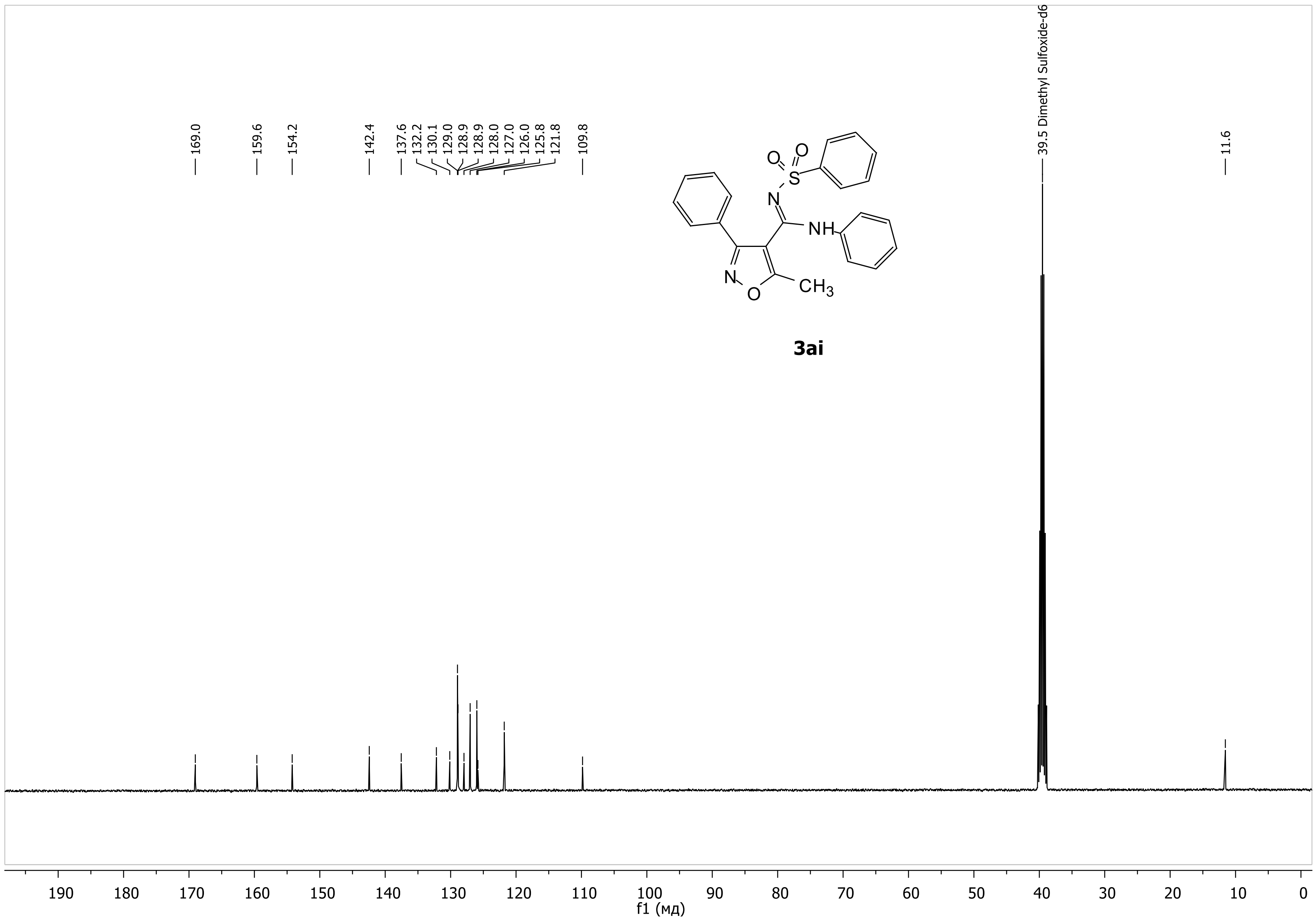

The ^{13}C NMR (100 MHz, DMSO-d_6) spectrum of compound **3ai**.

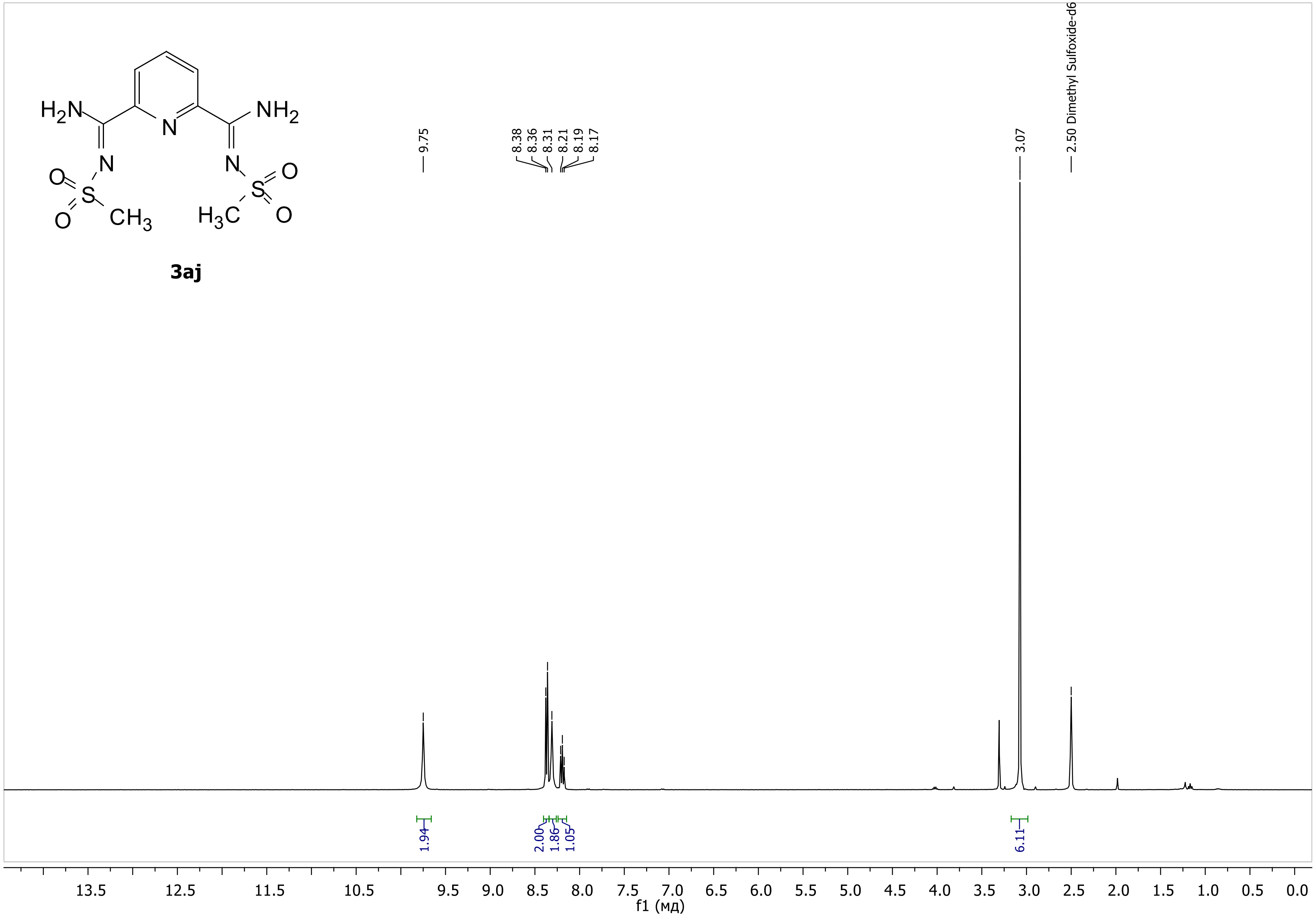

The ^{1}H NMR (400 MHz, DMSO-d_6) spectrum of compound **3aj**.

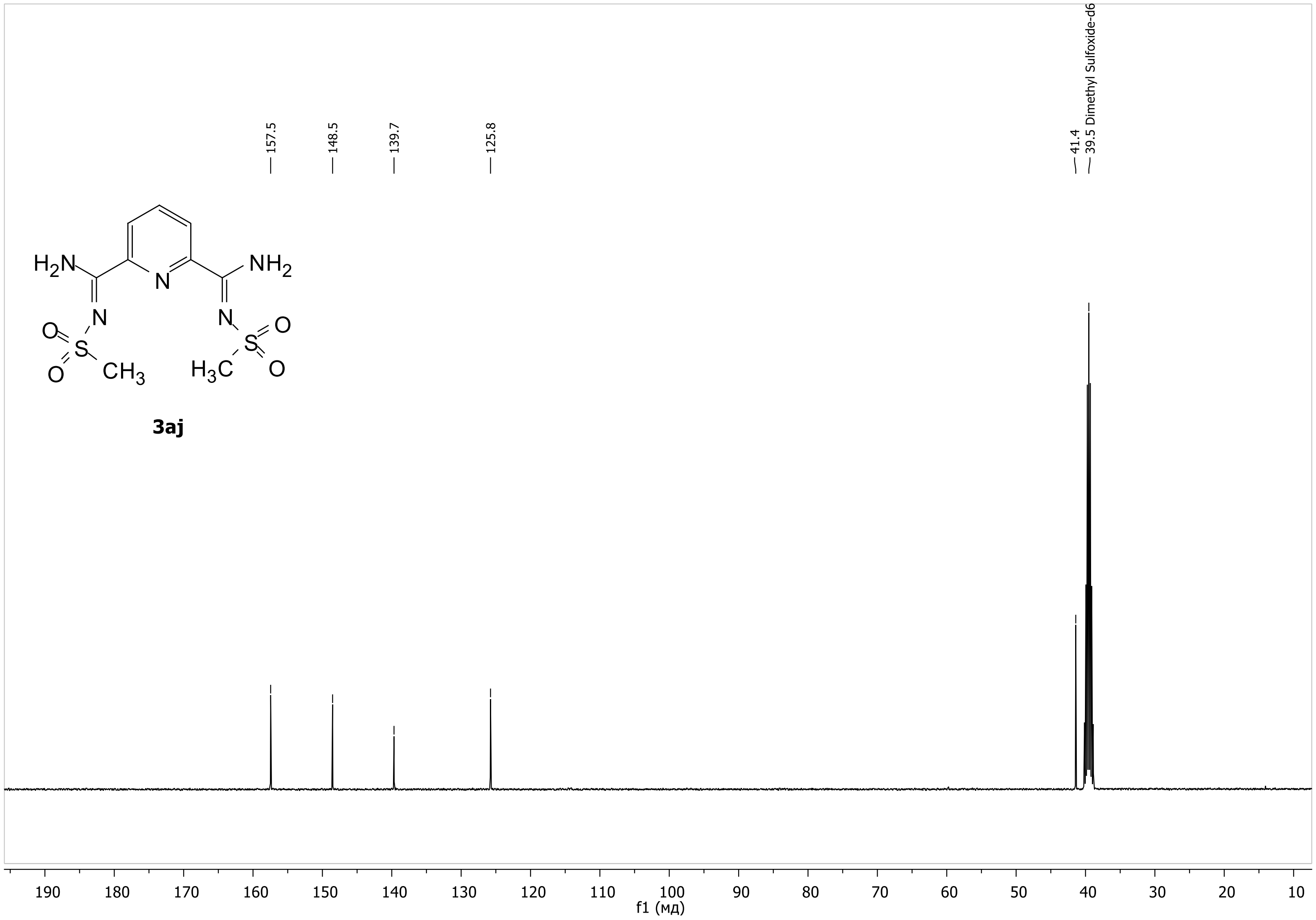

The ^{13}C NMR (100 MHz, DMSO-d_6) spectrum of compound **3aj**.

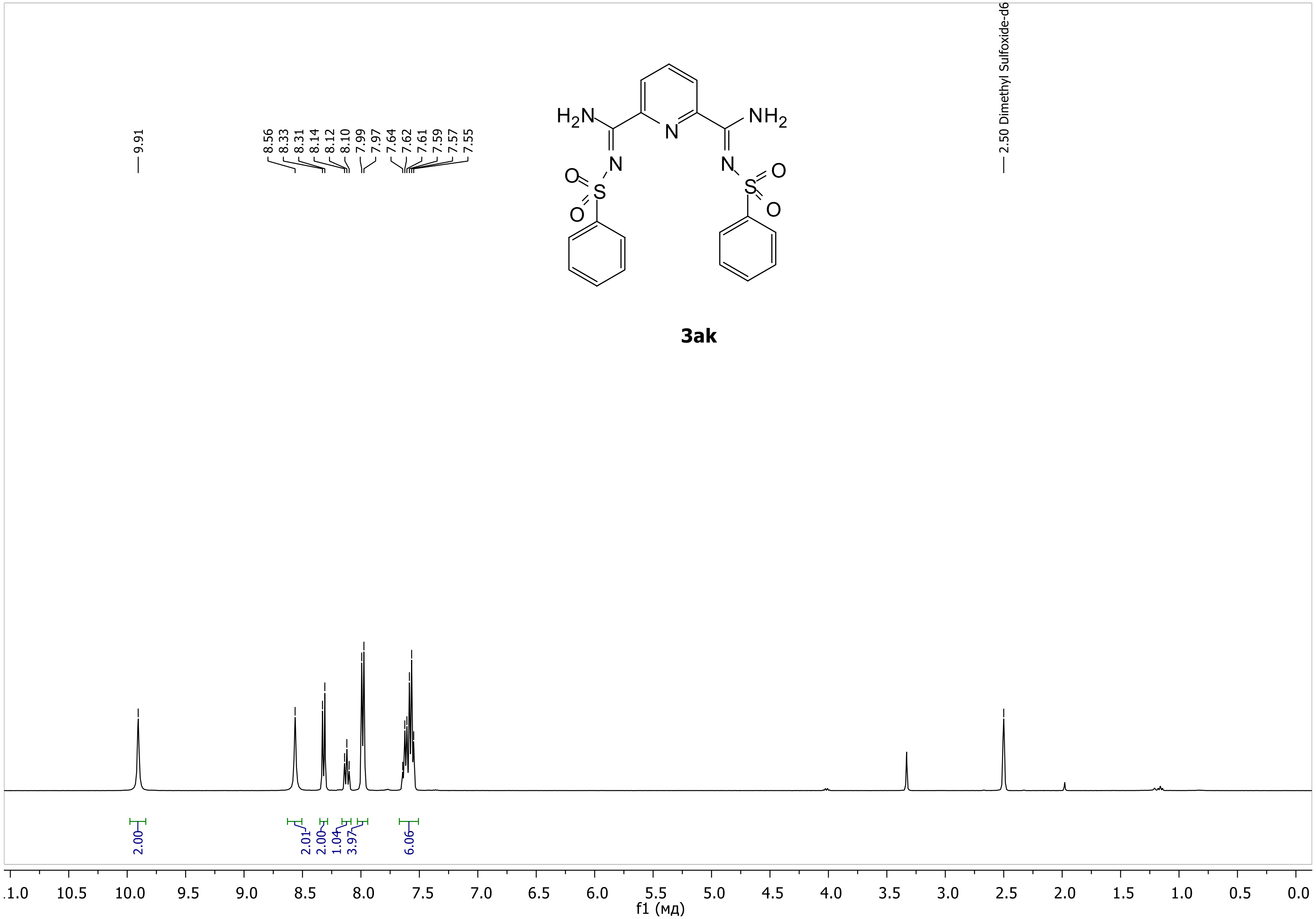

The ^{1}H NMR (400 MHz, DMSO-d_6) spectrum of compound **3ak**.

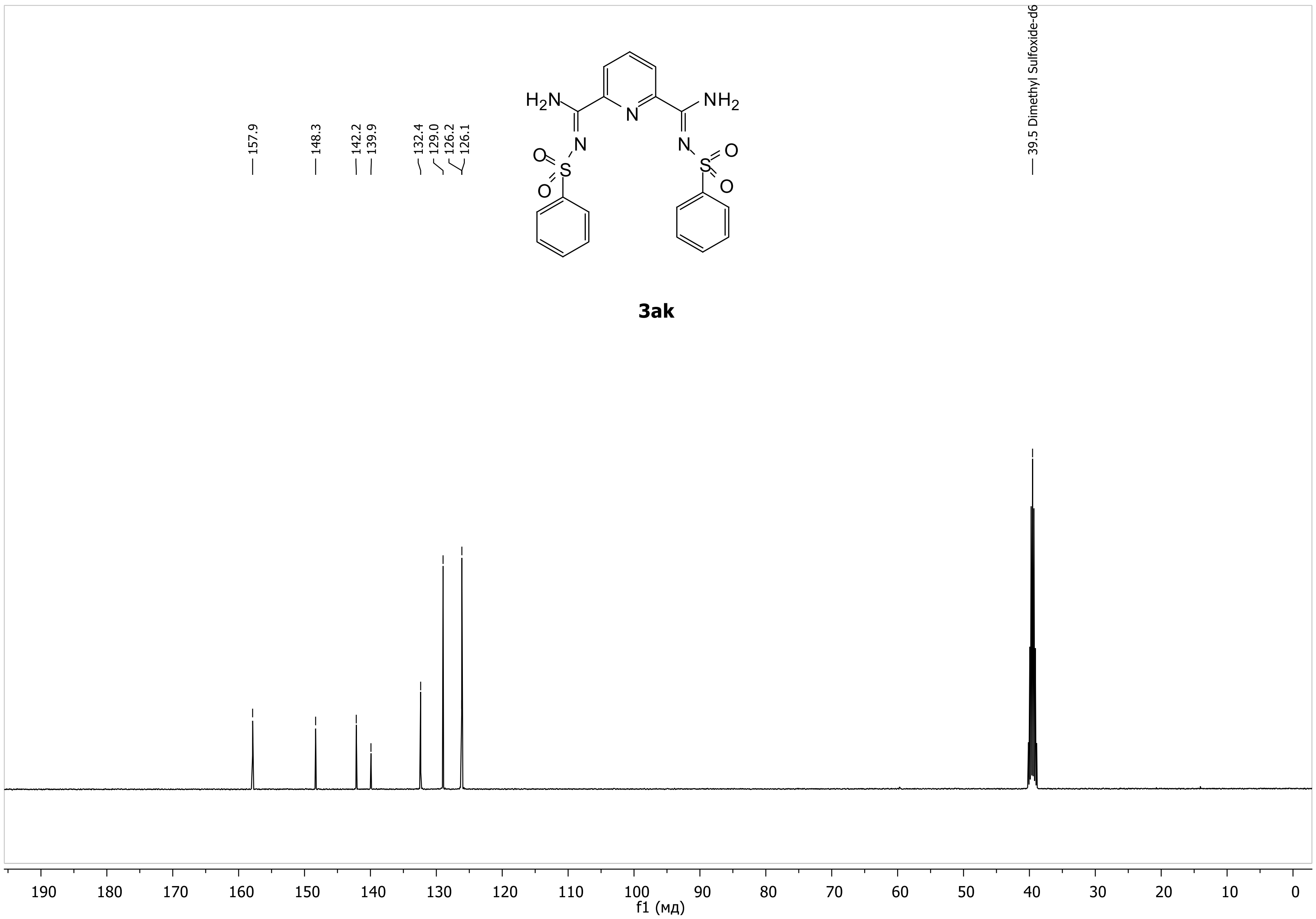

The ^{13}C NMR (100 MHz, DMSO-d_6) spectrum of compound **3ak**.

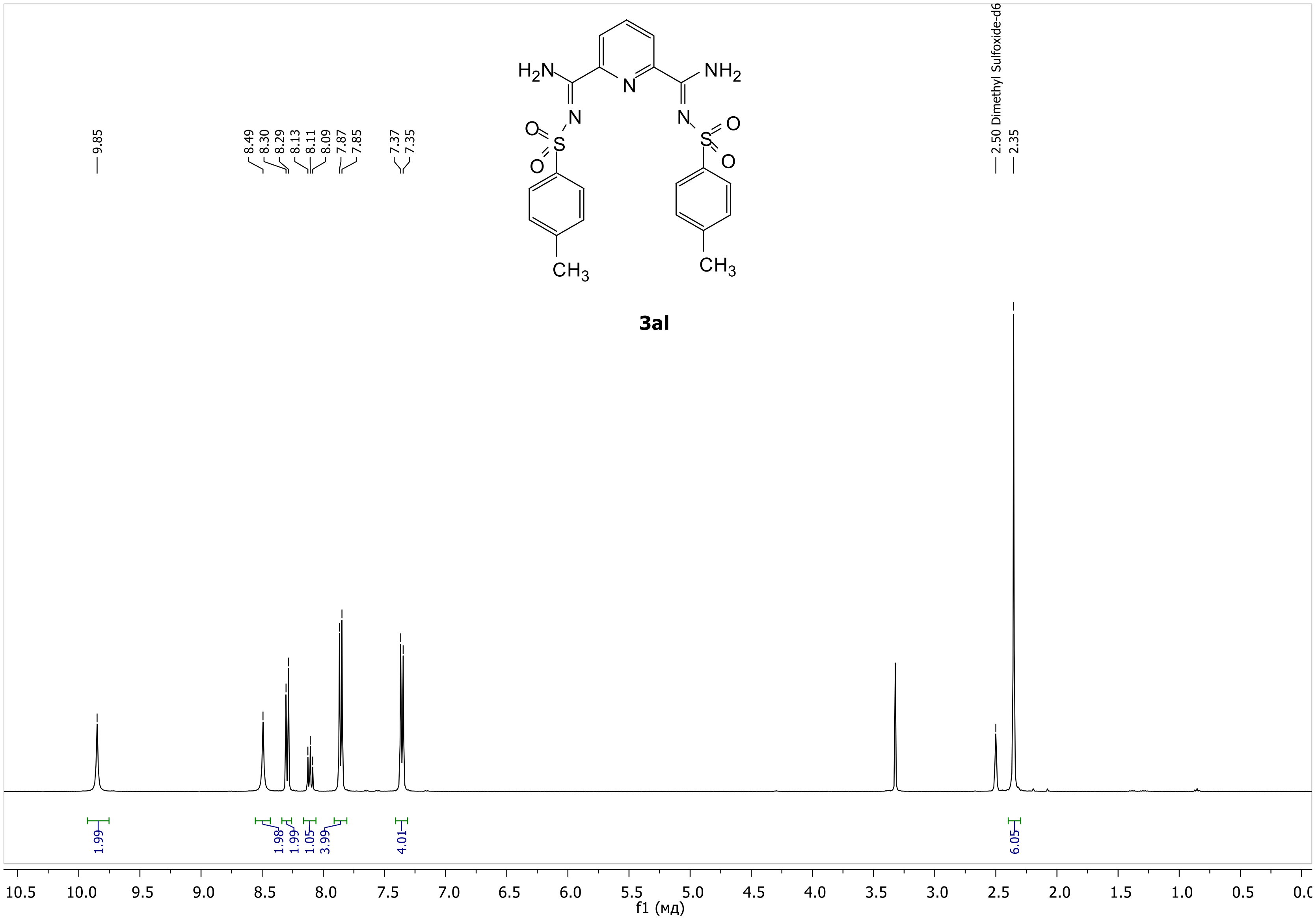

The ^{1}H NMR (400 MHz, DMSO-d$_6$) spectrum of compound **3al**.

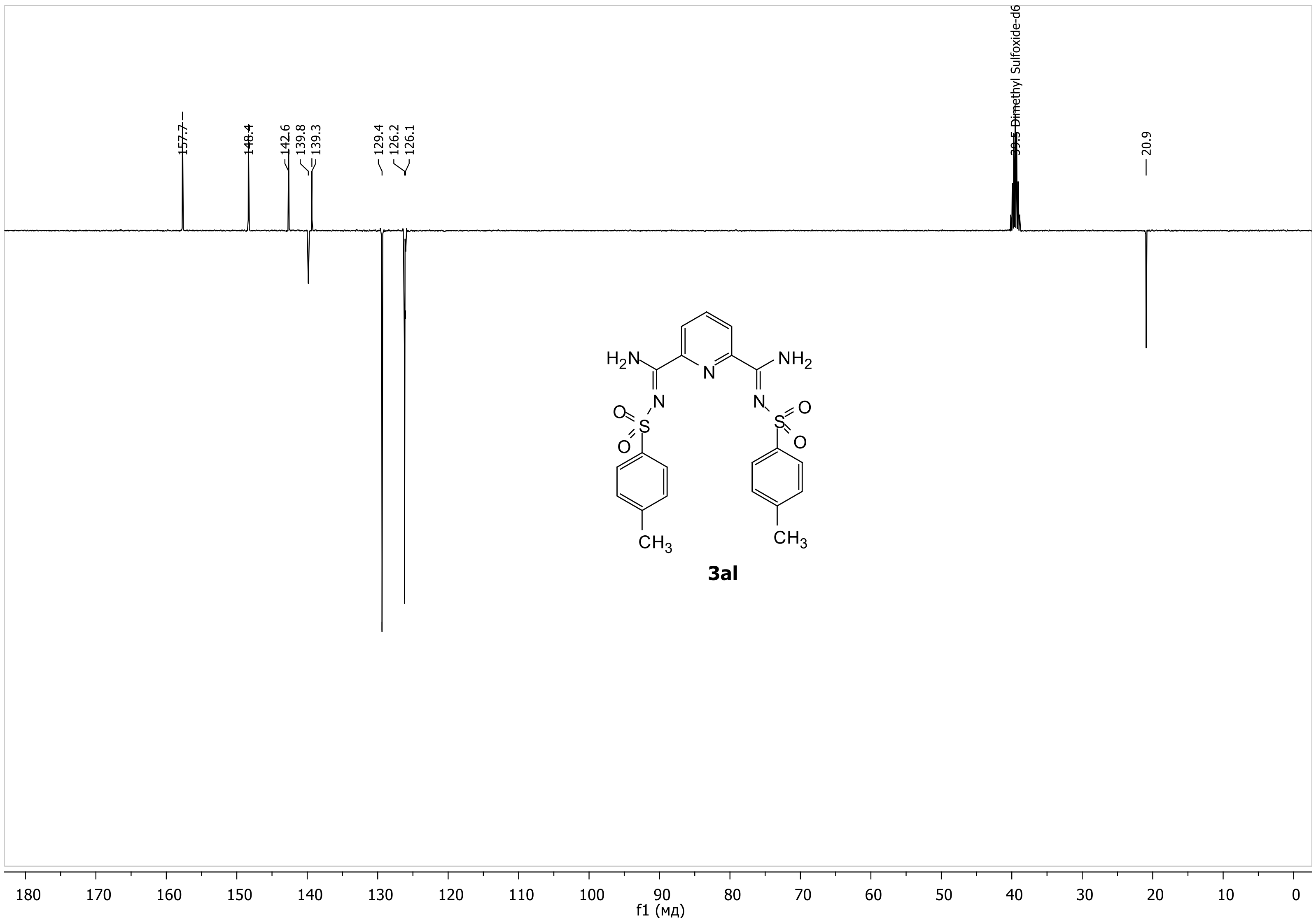

The ^{13}C NMR (100 MHz, DMSO-d_6) spectrum of compound **3al**.

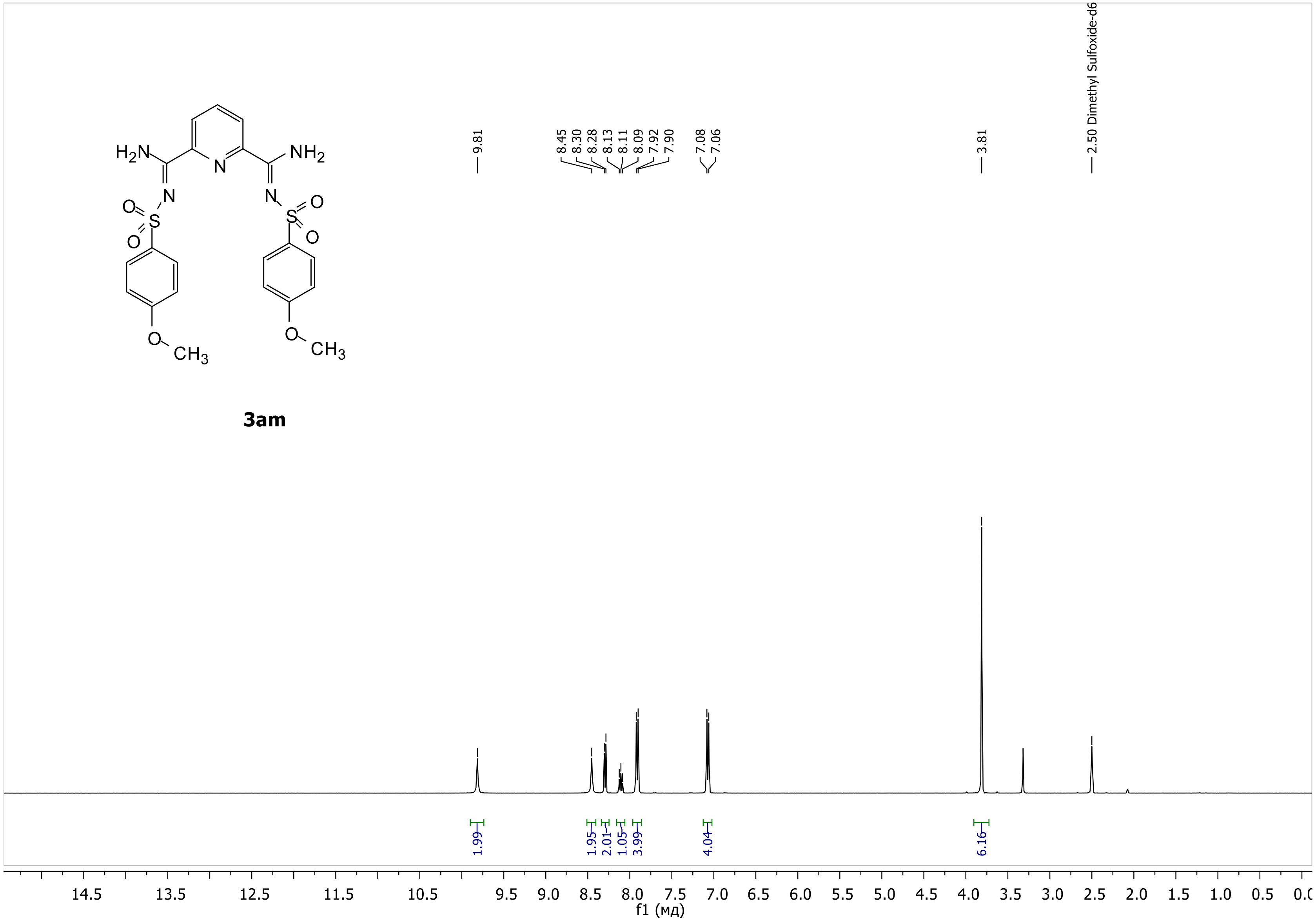

The ^{1}H NMR (400 MHz, DMSO-d_6) spectrum of compound **3am**.

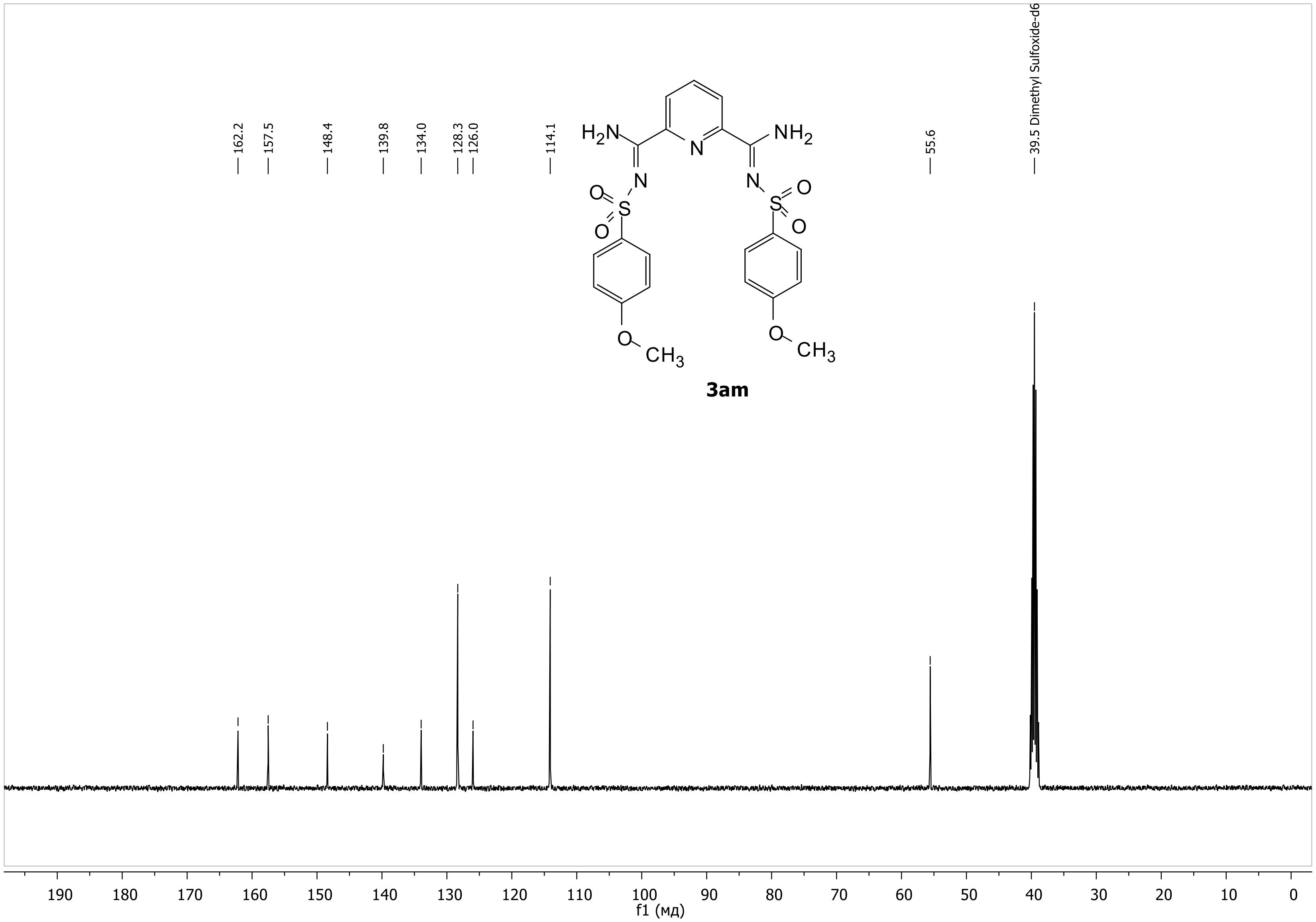

The ^{13}C NMR (100 MHz, DMSO-d_6) spectrum of compound **3am**.

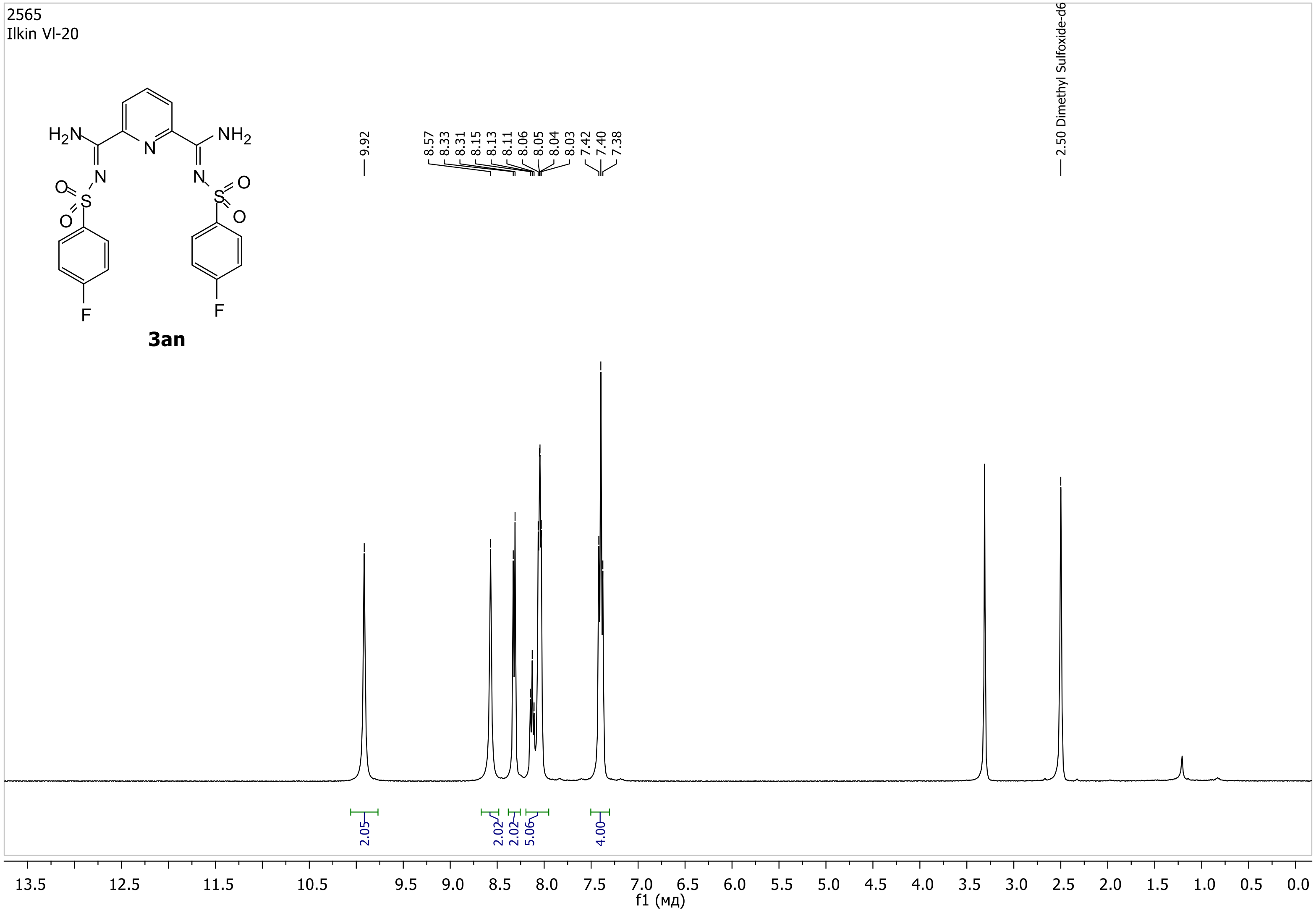

The ^{1}H NMR (400 MHz, DMSO-d_6) spectrum of compound **3an**.

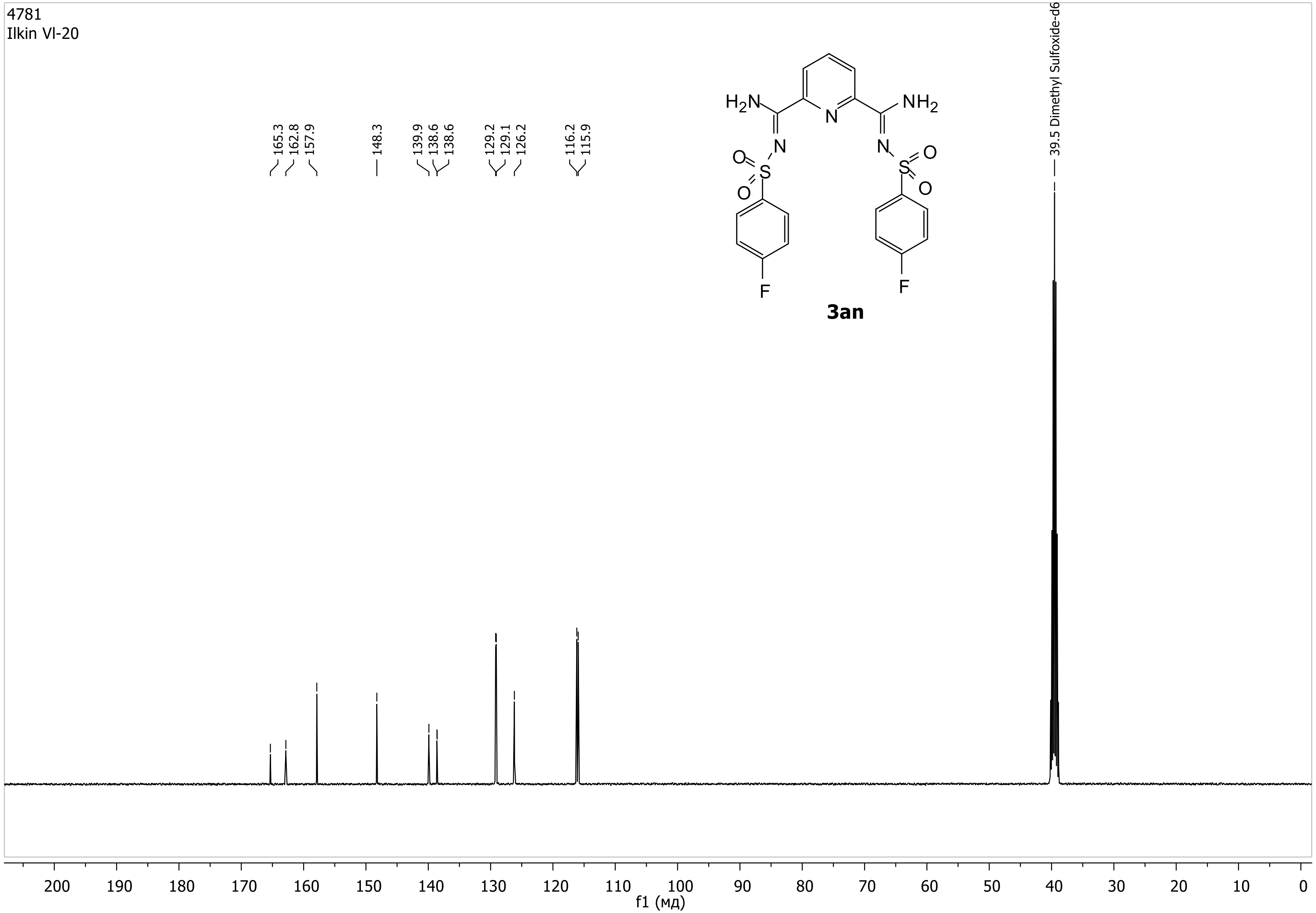

The ^{13}C NMR (100 MHz, DMSO-d_6) spectrum of compound **3an**.

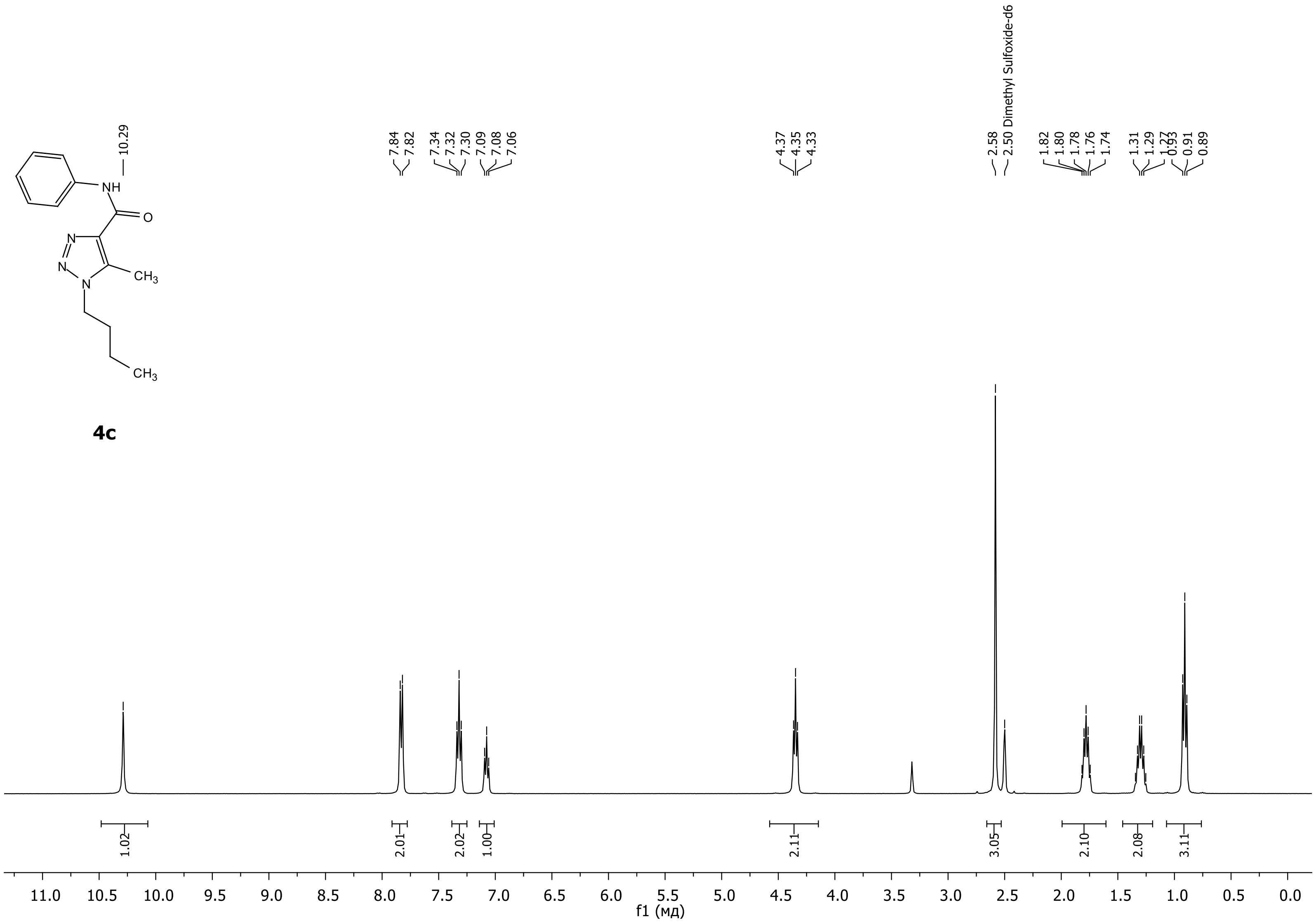

The ^{1}H NMR (400 MHz, DMSO-d_6) spectrum of compound **4c**.

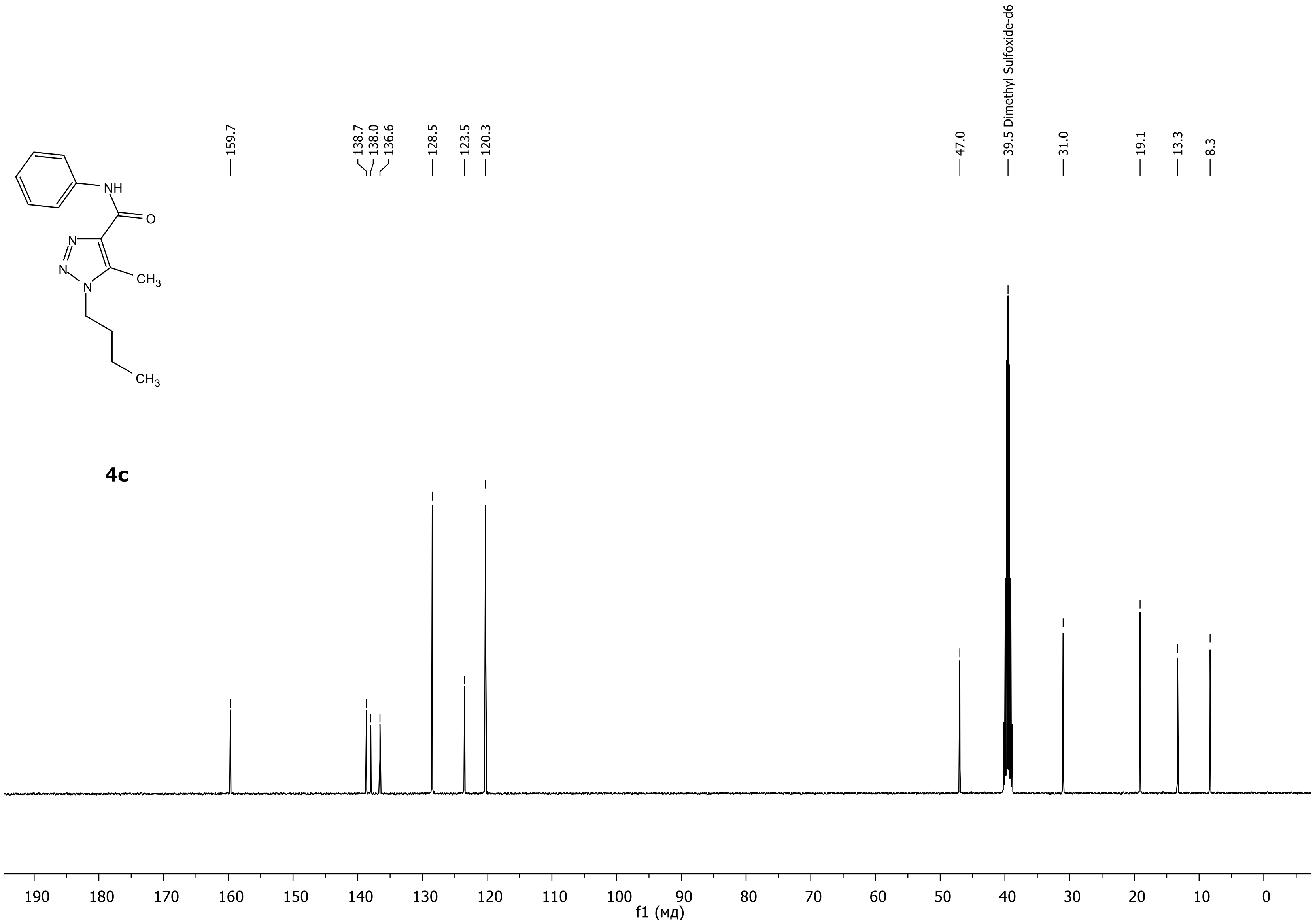

The ^{13}C NMR (100 MHz, DMSO-d_6) spectrum of compound **4c**.

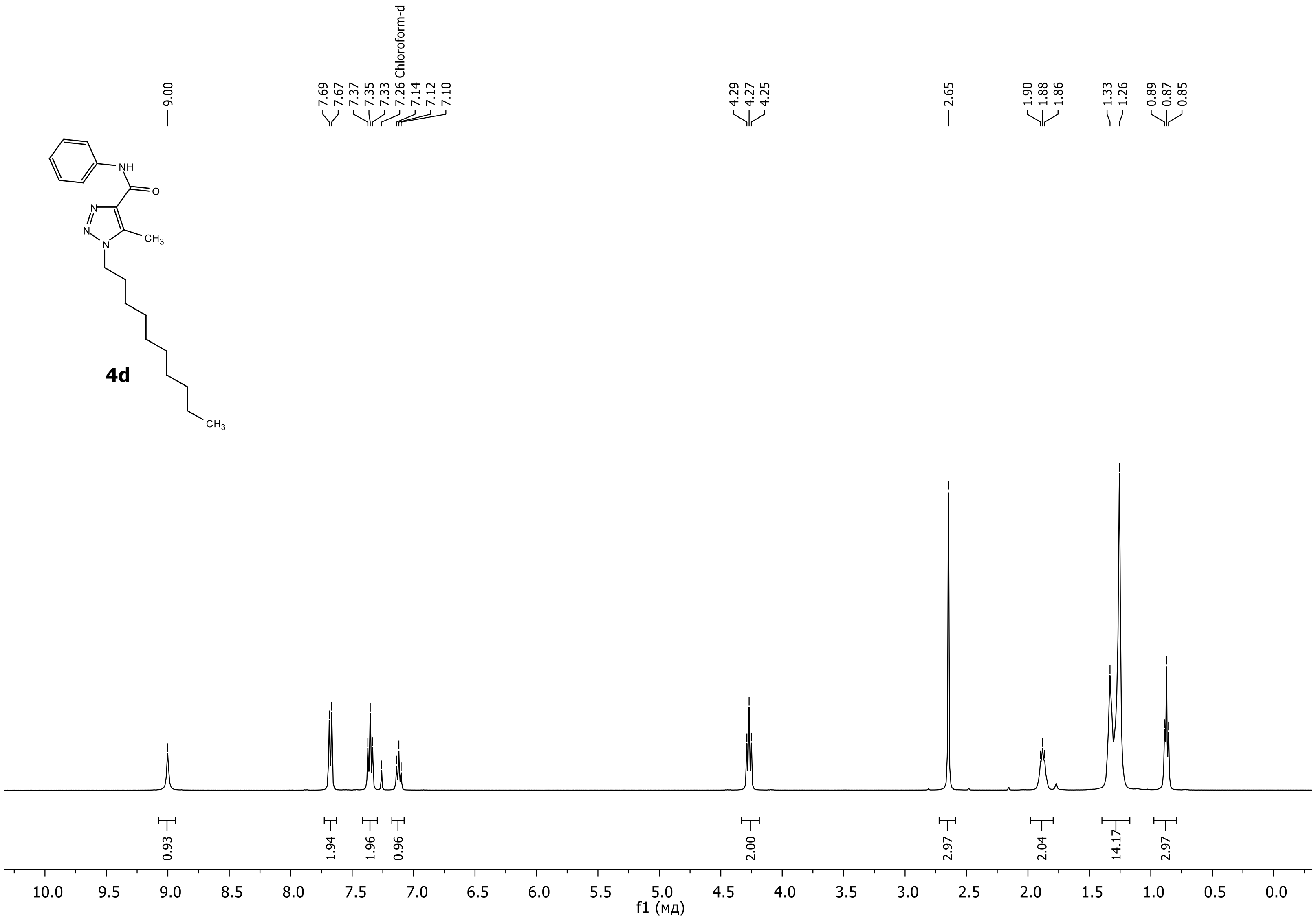

The ^{1}H NMR (400 MHz, $CDCl_3$) spectrum of compound **4d**.

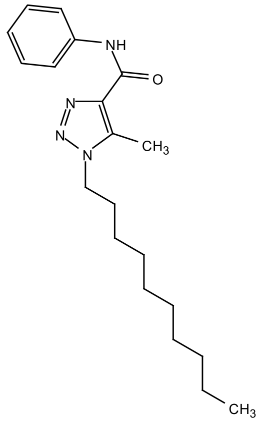

4d

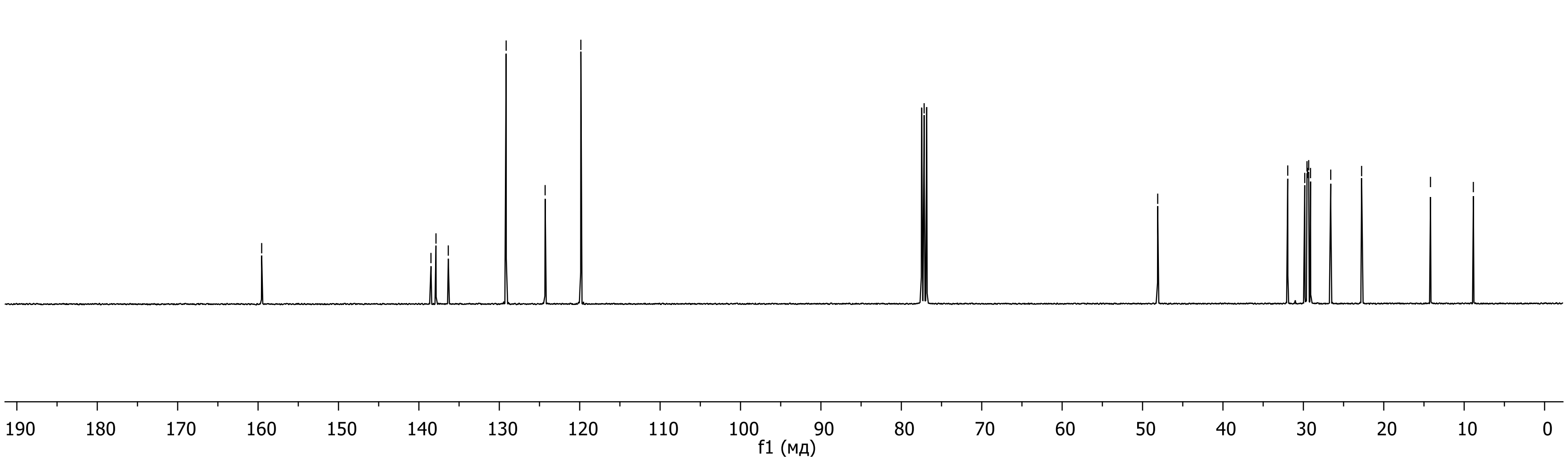

The ^{13}C NMR (100 MHz, $CDCl_3$) spectrum of compound **4d**.
